# Identification and Characterization of Chemosensory Receptors in the Pheromone Gland-Ovipositor of *Spodoptera frugiperda* (J. E. Smith)

**DOI:** 10.3390/insects13050481

**Published:** 2022-05-21

**Authors:** Ya-Lan Sun, Jun-Feng Dong, Hai-Bo Yang, Ding-Xu Li, Cai-Hong Tian

**Affiliations:** 1 College of Horticulture and Plant Protection, Henan University of Science and Technology, Luoyang 471000, China; yalansun@haust.edu.cn (Y.-L.S.); hbyang@haust.edu.cn (H.-B.Y.); ldigxu@163.com (D.-X.L.); 2 Institute of Plant Protection, Henan Academy of Agricultural Sciences, Zhengzhou 450002, China

**Keywords:** odorant receptor, gustatory receptor, ionotropic receptor, pheromone gland-ovipositor, transcriptome, real-time quantitative PCR, *Spodoptera frugiperda*

## Abstract

**Simple Summary:**

Chemical cues are generally thought to be primarily detected by the cephalic organ antennae, maxillary palps, and proboscises in insects. Although several recent studies have reported the chemosensory roles of ovipositors in some moth species, the expression of chemosensory receptors and their functions in the ovipositor remain largely unknown. Here, we systematically analyzed the pheromone gland-ovipositor (PG-OV) transcriptome of the fall armyworm, *Spodoptera frugiperda* (Lepidoptera: Noctuidae). A total of 26 candidate chemosensory receptor genes were revealed, including 12 odorant receptors (ORs), 4 gustatory receptors (GRs), and 10 ionotropic receptors (IRs). Specific genes including pheromone receptors, ORco, CO_2_ receptors, sugar receptors, and IR co-receptors were identified. Tissue expression profiling demonstrated that the annotated receptor genes were mainly expressed in the antennae (for ORs and IRs) or proboscis (for GRs), but two ORs, two GRs, and two IRs were also highly enriched in the PG-OV, with expression levels only slightly lower or even similar to those in the antennae/proboscis. This report provides the first large-scale description of chemosensory receptors in the PG-OV of *S. frugiperda*. It may inspire researchers to investigate how chemosensory receptors function in the ovipositor of *S. frugiperda*, as well as in the ovipositors of other moths.

**Abstract:**

Chemoreception by moth ovipositors has long been suggested, but underlying molecular mechanisms are mostly unknown. To reveal such chemosensory systems in the current study, we sequenced and assembled the pheromone gland-ovipositor (PG-OV) transcriptome of females of the fall armyworm, *Spodoptera frugiperda*, a pest of many crops. We annotated a total of 26 candidate chemosensory receptor genes, including 12 odorant receptors (ORs), 4 gustatory receptors (GRs), and 10 ionotropic receptors (IRs). The relatedness of these chemosensory receptors with those from other insect species was predicted by phylogenetic analyses, and specific genes, including pheromone receptors, ORco, CO_2_ receptors, sugar receptors, and IR co-receptors, were reported. Although real-time quantitative-PCR analyses of annotated genes revealed that OR and IR genes were mainly expressed in *S. frugiperda* antennae, two ORs and two IRs expressed in antennae were also highly expressed in the PG-OV. Similarly, GR genes were mainly expressed in the proboscis, but two were also highly expressed in the PG-OV. Our study provides the first large-scale description of chemosensory receptors in the PG-OV of *S. frugiperda* and provides a foundation for exploring the chemoreception mechanisms of PG-OV in *S. frugiperda* and in other moth species.

## 1. Introduction

The principal roles of adult insects are to find mating partners and habitats for the survival and reproduction of their offspring. To perform these functions, insects depend on chemosensory systems [1,2,3]. Insects mainly use antennae, and also other cephalic organs, such as maxillary palps and proboscis, to detect chemical cues from the outside environment [4,5]. Chemosensory sensilla scattered on these organs are hair-like structures innervated by the dendrites of chemosensory neurons [6,7]. Chemosensory receptors expressed on the dendritic membrane of chemosensory neurons mediate insect chemoreception. These receptors mostly belong to three families: the olfactory receptors (ORs), the gustatory receptors (GR), and the ionotropic receptors (IRs) [8].

Insect ORs and GRs, first identified in the *Drosophila melanogaster* genome [9,10,11,12,13], consist of 350 to 500 amino acids [14]. Genes in these two families possess the opposite membrane topology of G-protein-coupled receptors, with their N-termini internal to the cell and their C-termini external [15]. Insect functional ORs are demonstrated to be heterodimers that consist of a highly conserved protein called the odorant receptor co-receptor (ORco) and a ligand-specific ORx, which acts as non-selective ligand-gated ion channels [16,17]. Insect functional ORs have also been reported to be heterotetramers, based upon the structural data of Butterwick et al. [18]. ORs are broadly tuned to alcohols, aldehydes, ketones, and esters in the environment [19]. The number and protein sequences of ORs vary widely among insect orders [20,21,22]. Pheromone receptors (PRs), which represent a subclass of insect ORs, are proposed to be mainly involved in sex pheromone detections [23,24]. Members of the GR family, usually abundant in insect gustatory organs, detect nonvolatile compounds, including sugars, bitters, and plant secondary metabolites [25]. The GR family consists of several major subfamilies. One family mediates the perception of carbon dioxide [26], another senses the various sugars [27], and another specifically senses fructose [28,29]. While members of the “bitter taste” subfamily are supposed to perceive various bitter compounds as well as cuticular hydrocarbons [30]. Insect functional GRs can act independently or as heteromultimers, i.e., as ligand-gated ion channels [31]. Insect IRs have been identified in both the olfactory and gustatory organs. Genes in this family evolved from an ancient and highly conserved superfamily called ionotropic glutamate receptors (iGluRs) and share a similar structure and mechanism of action. The IRs consist of from 600 amino acids to 1000 amino acids. The architecture of IRs consists of two extracellular ligand-binding domains and three transmembrane domains [32]. Genes in the IR family, which have been well studied in *D*. *melanogaster*, function in the sensing of different odorants, including aldehydes, ammonia, acids, salts, and also humidity and temperature [33,34,35,36]. Sequence analyses and expression pattern studies split the IR family into two classes: “antennal IRs”, which are primarily expressed in the antennae and are mainly involved in olfaction, and “divergent IRs”, which are generally expressed in many tissues across the insect body and in some cases are responsible for taste sensing. In addition, IR co-receptor branches (including IR76b, IR25a, and IR8a) have also been reported. Similar to ORs, variable IR partners form heteromeric complexes with one or more co-receptors to perform their physiological functions [37,38].

Although detection of odorants in insects has been almost exclusively attributed to cephalic organs and especially to antennae, this inference has been challenged by a number of studies. For example, a pheromone-binding protein (PBP2) and a sex pheromone-specific OR (HR13) were detected in the female ovipositor of *Heliothis viresence* [39]. Studies on the ovipositor of *Manduca sexta* reported that a group of sensilla exhibited responses to a variety of host plant volatiles, and transcripts of ORco, IR25a, and IR8a were detected [40]. The OR31 of *Helicoverpa assulta*, which was co-expressed with ORco in ovipositor sensilla, was recently found to be involved in the detection of the host plant volatile *Z*-3-hexenyl butyrate [41]. These studies suggest a possible role of chemosensory receptors/chemoreception in moth ovipositors.

*Spodoptera frugiperda* (J. E. Smith) (Lepidoptera: Noctuidae), also known as the fall armyworm, is an important agricultural pest that is native to the Americas [42,43]. In 2016, *S*. *frugiperda* invaded Nigeria and then over 40 other African countries within two years [44,45,46]. It invaded Yunnan province in 2019 and has spread rapidly in many provinces across China [47,48,49]. *S*. *frugiperda* is a highly phytophagous pest causing severe damage to a great number of cultivated plant species [50]. Wind tunnel and field trapping studies demonstrated that *S*. *frugiperda* uses *Z*9-14: Ac and *Z*7-12: Ac as two principal sex pheromone components at a ratio of around 100:3.9 [51,52]. Electrophysiological assays combined with oviposition choice tests on gravid *S*. *frugiperda* moths revealed that the maize volatiles methyl salicylate and (*E*)-alpha-bergamotene are oviposition attractants, while (*E*)-4, 8-dimethyl-1, 3, 7-nonatriene is an oviposition deterrent, and geranyl acetate can act as an oviposition repellent or attractant depending on the host volatile context [53]. Studies of chemosensory receptors in *S*. *frugiperda* are currently confined to antennal transcriptome analysis and functional investigation of PRs [54,55,56]. The identity of the chemosensory genes in the *S*. *frugiperda* ovipositor and their functions in the ovipostion remain to be determined.

In most female moths, the ovipositor (OV) is anatomically closely connected to the sex pheromone gland (PG), which is the site of sex pheromone biosynthesis and emission. Together, the sex pheromone gland and ovipositor (the PG-OV) are important for the reproductive behavior of moths. To reveal potential chemosensory systems of PG-OV in *S*. *frugiperda*, we used Illumina sequencing in order to conduct a systematic analysis of the moth’s major chemosensory receptor genes. Phylogenetic trees showing the relationships between the candidate genes and homologs from other insect species were then constructed to gain insight into the possible functions of the candidate genes. In addition, we conducted real-time quantitative-PCR (RT-qPCR) to compare the expression profiles of these genes in male and female antennae, proboscises, and tarsi, and female PG-OVs. These findings provide a basis for further functional investigation of chemoreceptors in *S*. *frugiperda* ovipositors and in the ovipositors of other moth species.

## 2. Materials and Methods

### 2.1. Insect Rearing

Larvae of *S. frugiperda* were originally collected from a maize field in Shidian county, Baoshan, Yunnan Province, China. The collected larvae were maintained as a colony in a laboratory at Henan University of Science and Technology, Luoyang, China. The colony was reared for several generations with an artificial diet that mainly contained wheat germ, yeast, and corn leaf powder. The rearing conditions were 27 ± 1 °C, 70% relative humidity, and a 16 h: 8 h light/dark photoperiod. Pupae were sexed, and males and females were placed in separate cages (25 cm in diameter, 40 cm in length) for eclosion. Adults were provided with a 10% (*v/v*) honey solution that was renewed daily.

### 2.2. Tissue Collection

Because the mating activity was highest for two- to three-day-old moths, the PG-OVs of three-day-old virginal female moths were collected for transcriptome sequencing. Three replicates of PG-OV samples were collected, with each replicated sample collected from 80 individuals. For RT-qPCR measurements, tissues including male antennae, female antennae, male proboscises, female proboscises, male tarsi, female tarsi, and female PG-OVs were collected from three-day-old virginal moths; three replicates of each tissue sample were collected. All samples were collected during 2 to 3 h of the dark period and were stored at –80 °C until total RNA was extracted.

### 2.3. RNA Extraction, cDNA Library Construction, and Illumina Sequencing

Total RNA was extracted from the PG-OVs of *S*. *frugiperda* using Trizol reagent (Invitrogen, Carlsbad, CA, USA) according to the manufacturer’s protocol. The quantity of RNA was determined with an ND-2000 spectrophotometer (Nanodrop, Wilmington, DE, USA) and by 1.5% agarose gel electrophoresis. cDNA libraries were constructed at Sangon Biotech (Shanghai, China). The total RNA was treated with DNase I (RQ1, Promega, Madison, WI, USA). mRNA was then isolated from 10 µg of total RNA using a Dynabeads mRNA Purification Kit (Invitrogen, MA, USA). Paired-end RNA-seq libraries were then prepared by following Illumina’s library construction protocol. The libraries were then sequenced on an Illumina HiSeq2000 platform (Illumina, CA, USA) at Sangon Biotech (Shanghai, China).

### 2.4. De Novo Assembly of Short Reads

De novo assembly and annotation of unigenes were performed as previously described [22]. The raw reads were initially processed to remove the adapter sequences and low-quality bases using the Trimmomatic package [57]. The Q30 and GC-content package was used to verify the sequencing quality. The clean reads were then assembled to produce contigs using the Trinity RNA-Seq de novo transcriptome assembly program (https://github.com/trinityrnaseq/trinityrnaseq/, accessed on 1 September 2020).

### 2.5. Gene Annotation and Identification of Chemosensory Receptors

Unigenes were annotated as previously described [58]. Unigenes larger than 150 bp were first aligned with BLASTx to protein databases, including the Nr database in the NCBI, Swiss-Prot, KEGG (Kyoto encyclopedia of genes and genomes), and COG (Clusters of Orthologous Groups of proteins); an e-value cut-off of 1e-5 was used to retrieve proteins with the highest sequence similarity with the given proteins along with their functional annotations. The Blast2GO program (https://www.blast2go.com/, accessed on 1 November 2020) was then used to obtain GO annotation of the unigenes [59], and GO functions were categorized by using WEGO 2.0 [60].

Transcripts encoding putative ORs, GRs, and IRs were then manually aligned and compared using the NCBI BLASTX. The open reading frames (ORFs) of these genes were predicted with ORFfinder (https://www.ncbi.nlm.nih.gov/orffinder/, accessed on 1 January 2021). TPM (transcripts per kilobase of exon per million mapped) values were calculated by using the RSEM package to indicate the abundance of different candidate genes in the *S. frugiperda* PG-OV.

### 2.6. Expression Profiling by RT-qPCR

The expression profiling of candidate chemosensory receptor genes was carried out using RT-qPCR. Trizol reagent (Invitrogen, Carlsbad, CA, USA) was used to isolate the total RNA from the antennae of 40 male moths, the antennae of 40 female moths, the proboscises of 30 male moths, the proboscises of 30 female moths, the tarsi of 50 male moths, the tarsi of 50 female moths, and the PG-OVs of 50 female moths. The extracted RNA of each sample was first treated with DNase I (RQ1, Promega) and was then subjected to reverse transcription for first-strand cDNA synthesis by M-MLV Reverse Transcriptase (Promega, Madison, WI, USA) according to the manufacturer’s manual. RT-qPCR was then performed with a Roche LightCycler 480 (F. Hoffmann-La Roche Ltd., Basel, Switzerland). Operations were carried out following the manufacturer’s instructions for SYBR Premix ExTaq II (Tli RNaseH Plus, Takara, Dalian, China): a 10-µL volume of SYBR Premix ExTaq II, 0.4 mM of each primer, and 2.5 ng of sample cDNA were mixed before sterilized deionized H_2_O was added to make a final volume of 20 µL. The reaction programs were 95 °C for 30 s; 40 cycles of 95 °C for 5 s and 60 °C for 30 s; followed by 95 °C for 1 min and 55 °C for 1 min. Fluorescence values were measured over a 55 to 95 °C melting curve in order to check the absence of primer dimer peaks. Non-template reactions (replacing cDNA with H_2_O) were conducted as negative controls. Expression levels of all detected genes were calculated using the 2^−ΔCt^ method [61], with the *β*-*actin* gene as an internal control for sample normalization. Amplification curves (S-shaped) and CT values (ranging from 16.4–18.2) for the reactions of the *β*-*actin* gene were carefully checked to make sure it is consistent across different tissues. Three biological replicates were performed for the evaluation of gene expression levels. In each biological replicate, samples of different tissues were collected separately, and three technical replicates were performed for each collected sample. The results are reported as means ± standard error (SE). One-way analysis of variance (ANOVA) with Tukey LSD tests was used to compare the RT-qPCR data (*p* < 0.05). Figures were made using GraphPad Prism 6 (GraphPad Software Inc., San Diego, CA, USA). Primers (Supplementary Table S1) were designed using Primer Premier 6.0 (PREMIER Biosoft International, CA, USA). The relative copy numbers of the chemoreceptors and *β*-*actin* genes were calculated using the relative standard curve method to avoid the unequal efficiencies of the primers.

### 2.7. Phylogenetic Analyses

A neighbor-joining tree was constructed for phylogenetic analyses of candidate chemosensory receptor genes in *S*. *frugiperda* and their homologs from other insect species. The OR data sets contained sequences from *S*. *frugiperda*, *Bombyx mori*, and *H. armigera*. The GR data sets contained sequences from *S*. *frugiperda*, *B. mori*, *H. armigera*, and *Danaus plexippus*. The IR data sets contained sequences from *S*. *frugiperda*, *H. armigera*, *Dendrolimus punctatus,* and *D*. *melanogaster*. Amino acid sequences were first aligned with ClustalX [62]. The phylogenetic trees of the ORs, GRs, and IRs were then constructed using MEGA 7.0 [63]. The evolutionary distances were computed using the JTT matrix-based method [64]. All ambiguous positions were removed for each sequence pair. Node support was assessed using a bootstrap procedure based on 1000 replicates. Phylogenetic trees were finally colored and arranged with Figtree v1.4.2 (http://tree.bio.ed.ac.uk/software/figtree, accessed on 1 September 2021). The amino acid sequences of the genes used for phylogenetic tree building are listed in Supplementary Table S2.

## 3. Results

### 3.1. Transcriptome Sequencing and Sequence Assembly

The RNA extracted from the PG-OV of *S*. *frugiperda* was reverse transcribed and then sequenced using the Illumina HiSeq 2000 platform. An average of 40.73 million clean reads were produced, and the average percentage of Q30 bases was ≥89.67% (Supplementary Table S3). An assembly of 119,928 unigenes were finally generated, with a mean length of 569 bp and an N50 length of 785 bp. More than 12.13% of the unigenes have a length longer than 1000 bp (Supplementary Table S4).

### 3.2. GO Annotation and Classification

Of the 119,928 unigenes, 27,815 (23.19%) had hits in the NR database with an E-value cut-off of 1 × 10^−5^. Among the annotated unigenes, 23,547 (84.65%) had best matches to lepidopteran sequences. The highest percentage of matched sequences was to *Amyelois transitella* (23.30%), followed by *Bombyx mori* (18.17%), *Papilio xuthus* (8.86%), *Operophtera brumata* (7.36%), *P*. *machaon* (7.25%), *Plutella xylostella* (5.39%), *P*. *polytes* (4.76%), *Spodoptera litura* (1.69%), and *H*. *armigera* (1.63%). The remaining 15.34% sequences were matched to other insects (Supplementary Figure S1).

Gene Ontology (GO) annotation was performed to classify the 119,928 unigenes into functional groups using BLAST2GO. Based on the sequence homology, 38,496 unigenes (32.10%) were annotated, and each identified sequence was allocated to at least one GO term of the three biological processes. A total of 20,531 (17.12%) were assigned to a cellular component, 18,336 (15.29%) to a molecular function, and 38,544 to a biological process (32.14%). The most abundant and enriched GO term in the molecular function category was “binding” and “catalytic activity”. Among the biological process terms, “cellular process” and “metabolic process” were the most represented. Among the cellular component terms, “cell” and “cell part” were the most abundant (Supplementary Figure S2).

### 3.3. Analysis of Odorant Receptors

A total of 12 ORs were identified in the *S*. *frugiperda* PG-OV (Table 1). Transcripts for all ORs have complete ORFs based on the presence of predicted start codons, stop codons, and blast-based alignment to other homologous sequences (Supplementary Table S5). For uniformity, the identified chemoreceptors here were named following the reported sequences of *S*. *frugiperda* (whenever possible) [54,65] or the best matched sequences in *H*. *armigera*. All putative ORs identified here displayed 99–100% amino acid sequence identities to the reported SfruORs sequences in a genomic analysis [65].

**Figure 1:**
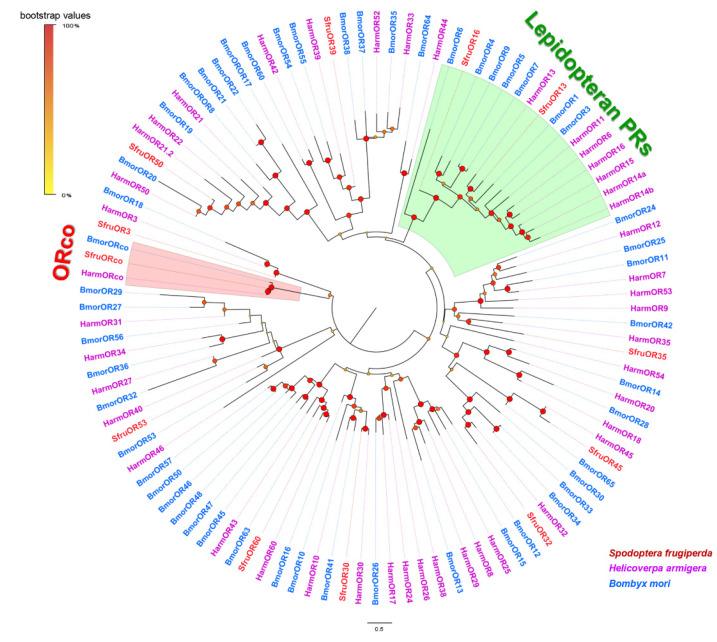
Phylogenetic relationships of ORs from *S. frugiperda* and other lepidopteran species. The neighbor-joining tree was constructed using MEGA7 and was based on candidate ORs from *S. frugiperda* (Sfru), *B. mori* (Bmor), and *H. armigera* (Harm). Branches of the ORco clade are highlighted in pink; branches containing the lepidopteran pheromone receptors (PRs) are highlighted in green.

A phylogenetic tree indicating evolutionary relationships of ORs between *S*. *frugiperda* and the selected Lepidopteran species *B*. *mori* and *H*. *armigera* was constructed. The results showed that ORco genes from the three species were highly conserved and clustered in one branch. Two ORs in *S*. *frugiperda*, including SfruOR13 and SfruOR16, clustered in the lepidopteran PR clade [55,56] (Figure 1).

**Figure 2:**
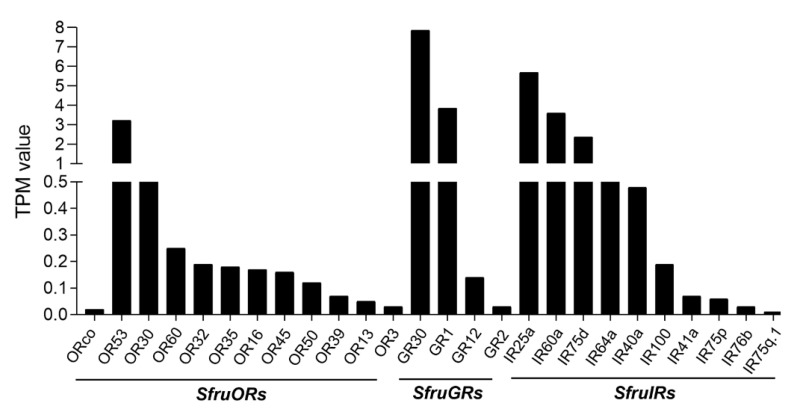
TPM values of candidate ORs, GRs, and IRs in the pheromone gland-ovipositor of *S. frugiperda*.

The expression levels of the 26 candidate chemosensory receptor genes were normalized across sequencing libraries using the TPM scaling factor. TPM values of the *SfruORs* indicated that *SfruOR53* was the most abundantly expressed OR (3.23 TPM) in the PG-OV of *S*. *frugiperda*. Two candidate PRs, *SfruOR13* and *SfruOR16*, showed relatively low expression levels, with TPM values of 0.05 and 0.17, respectively. Most importantly, *SfruORco* was also detected, but its TPM value was very low (0.02) (Table 1, Figure 2).

**Figure 3:**
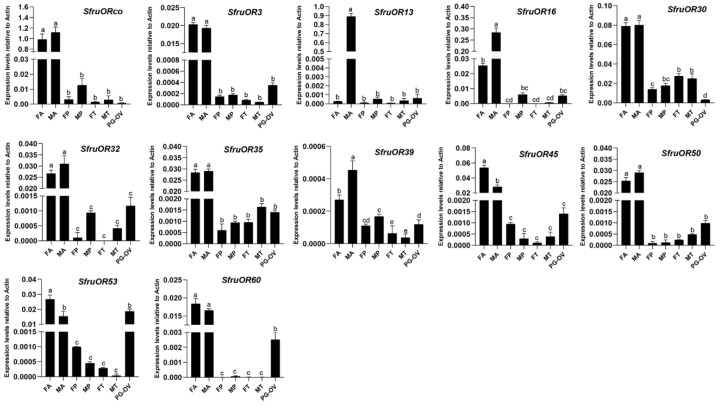
Expression patterns of candidate *ORs* in different tissues of *S. frugiperda*. RT-qPCR analysis of candidate *OR* genes was carried out in female antennae (FA), male antennae (MA), female proboscises (FP), male proboscises (MP), female tarsi (FT), male tarsi (MT), and the female pheromone gland-ovipositor (PG-OV). Values are means + SE; in each panel, means with different letters are significantly different according to a one-way ANOVA followed by Tukey’s multiple comparison test (*p* < 0.05, *n* = 3).

RT-qPCR was conducted to further investigate the expression pattern of all candidate chemosensory receptor genes encoding candidate SfruORs in various tissues, including antennae, proboscises, and tarsi of both sexes as well as the female ovipositor. Although expression levels of the candidate OR genes differed among the tissues, all of the OR genes were mainly expressed in antennae, and expression levels of *SfruOR13*, *SfruOR16*, and *SfruOR39* were significantly higher in males than in females (*p* < 0.05); *SfruOR13,* in particular, was almost exclusively expressed in male antennae. Expression levels of *SfruOR45* and *SfruOR53* were higher in female antennae than in male antennae. The RT-qPCR results also indicated that *SfruOR53* and *SfruOR60* were highly expressed in the PG-OV, and that the expression level of *SfruOR*53 in the PG-OV was similar to that in the antennae. Although transcripts of *SfruORco* were also detected in PG-OV, its expression level was much lower than that in the antennae of both sexes (Figure 3).

### 3.4. Analysis of Gustatory Receptors

A total of four putative GRs were identified based on the analysis of the transcriptome of the *S*. *frugiperda* PG-OV (Table 1). Complete ORFs were identified for all of the annotated GR genes in our study (Supplementary Table S5), and their amino acid identities with the consensus sequences derived from the genome of *S*. *frugiperda* were 100% [65].

**Figure 4:**
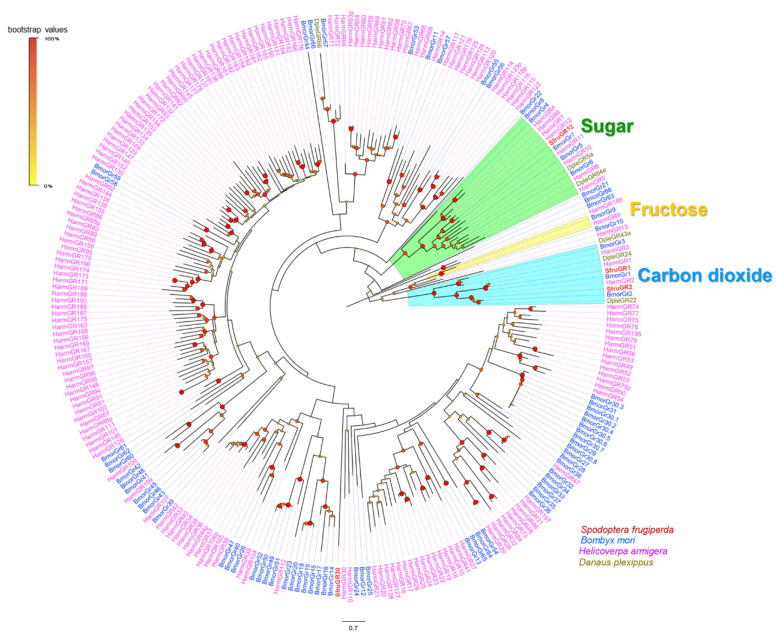
Phylogenetic relationships of GRs from *S. frugiperda* and other lepidopteran species. The neighbor-joining tree was constructed using MEGA7 based on candidate GRs from *S. frugiperda* (Sfru), *H. armigera* (Harm), *B. mori* (Bmor), and *Danaus plexippus* (Dple). Branches of the putative carbon dioxide receptors are highlighted in blue; branches of putative fructose receptors are highlighted in yellow; branches containing “sugar-taste receptors” are highlighted in green; and branches containing “bitted-taste receptors” are not highlighted.

A phylogenetic tree constructed with GR sequences from *S*. *frugiperda*, *H*. *armigera*, and *B*. *mori* was used to infer the functions of the candidate genes. SfruGR1 and SfruGR2, which grouped with HarmGR1/2/3 and BmorGR1/2/3, were putative candidate CO_2_ receptors [66,67]. SfruGR12 clustered with the BmorGR4/5/6/7/8 lineage, which detects sugar in *B*. *mori* [68]. One GR, SfruGR30, clustered in the clades containing putative bitter-taste receptors (Figure 4).

As indicated by TPM values, the abundance of GR transcript levels in the *S*. *frugiperda* PG-OV was highest for SfruGR30 (7.85 TPM), i.e., SfruGR30 transcript levels were the highest among all of the annotated GRs (Table 1, Figure 2).

**Figure 5:**
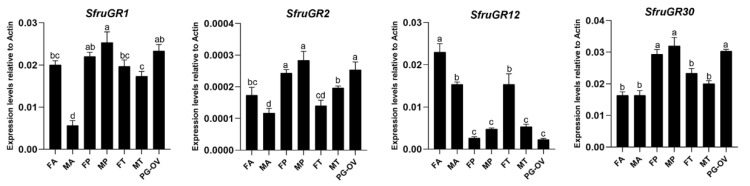
Expression patterns of candidate *GRs* in different tissues of *S. frugiperda*. RT-qPCR analysis of candidate *GR* genes was carried out in female antennae (FA), male antennae (MA), female proboscises (FP), male proboscises (MP), female tarsi (FT), male tarsi (MT), and the female pheromone gland-ovipositor (PG-OV). Values are means + SE; in each panel, means with different letters are significantly different according to a one-way ANOVA followed by a Tukey’s multiple comparison test (*p* < 0.05, *n* = 3).

According to the RT-qPCR results, two GR genes, *SfruGR2* and *SfruGR30*, were mainly expressed in the female PG-OV and in the proboscis of both sexes, and their expression was significantly higher in the female PG-OV and in the proboscises of both sexes than in the antennae and tarsi (*p* < 0.05). Although *SfruGR1* was enriched in proboscis and the female PG-OV, it was also highly expressed in the female antennae. In contrast, the expression of *SfruGR12* was significantly higher in female antennae than in other tissues (Figure 5).

### 3.5. Analysis of Ionotropic Receptors

A total of 10 predicted SfruIRs were annotated in the transcriptome of the *S*. *frugiperda* PG-OV (Supplementary Table S5). Among these candidate genes, full-length ORFs were identified for eight SfruIRs, but only partial sequences were identified for the other two IRs (SfruIR41a/75d) (Table 1).

**Figure 6:**
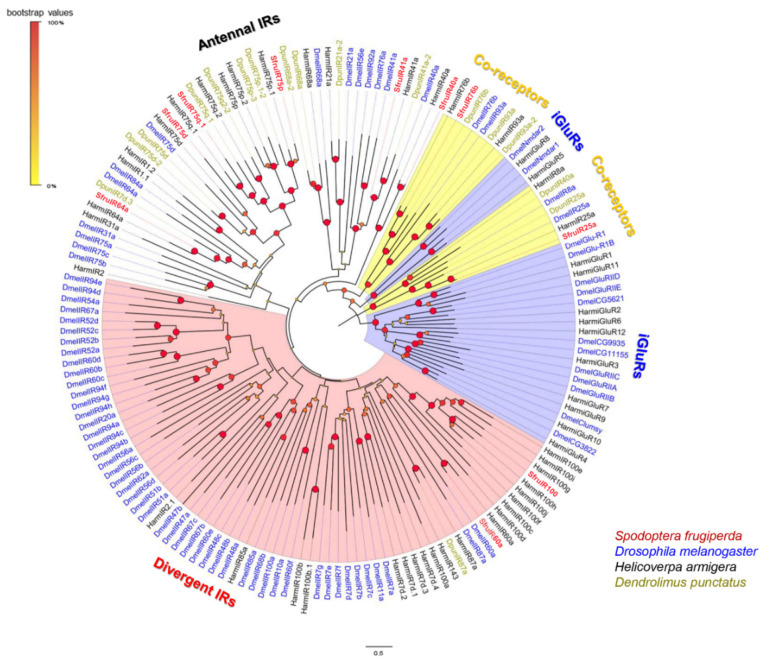
Neighbor-joining tree of candidate IRs from *S. frugiperda* (Sfru), *H*. *armigera* (Harm), *D. punctatus* (Dpun), and *D. melanogaster* (Dmel). Branches of IR co-receptors are highlighted in yellow; branches of the putative ionotropic glutamate receptors (iGluRs) are highlighted in blue; branches of the putative “divergent IRs” are highlighted in pink; branches of the putative “antennal IRs” are not highlighted.

A phylogenetic tree was constructed to indicate evolutionary relationships between *S*. *frugiperda* IRs and a selection of those from *D*. *melanogaster*, *H*. *armigera*, and *D*. *punctatus* (Figure 6). The putative IR co-receptors of *S*. *frugiperda*, SfruIR25a and SfruIR76b, clustered with the highly conserved co-receptor lineages of DmelIR25a and Dmel76b, respectively. Other than SfruIR60a and SfruIR100, which were in the “divergent IRs” clade, the other SfruIRs (SfruIR40a/41a/64a/75d/75p/75q.1) were in the putative “antennal IR” clade (Figure 6).

According to TPM values, SfruIR25a, which is an ortholog of the co-receptor DmelIR25a, was the most abundant of the *SfruIRs* in the PG-OV of *S*. *frugiperda* (5.69 TPM). The putative “divergent IR”, SfruIR60a, was also abundantly expressed in the PG-OV (3.69 TPM). Another co-receptor SfruIR76b, which is an ortholog of the co-receptor DmelIR76b, had quite low TPM values (0.03) (Figure 2).

**Figure 7:**
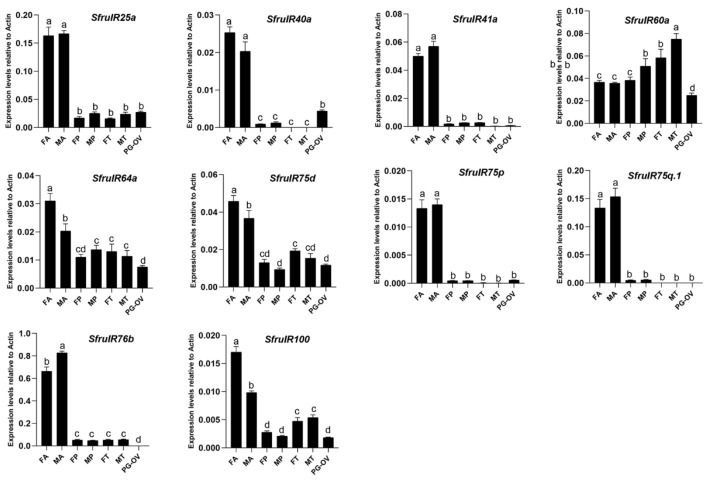
Expression patterns of candidate *IRs* in different tissues of *S. frugiperda*. RT-qPCR analysis of candidate *IRs* genes was carried out in female antennae (FA), male antennae (MA), female proboscises (FP), male proboscises (MP), female tarsi (FT), male tarsi (MT), and the female pheromone gland-ovipositor (PG-OV). Values are means + SE; in each panel, means with different letters are significantly different according to a one-way ANOVA followed by a Tukey’s multiple comparison test (*p* < 0.05, *n* = 3).

Although the expression levels of SfruIRs differed among different chemosensory tissues, the expression of most IR genes (except SfruIR60a) was highest in antennae; the expression of three IR genes (SfruIR64a/75d/100) was highest in female antennae; the expression of SfruIR76b was highest in male antennae. The “divergent IR”, SfruIR60a, was highly expressed in all of the tested tissues, but its expression was significantly higher in male tarsi than in the other tissues. Most of the IRs were expressed in the PG-OV at a significantly lower level than in other tissues, except for the SfruIR25a and SfruIR40a; although the expression levels of SfruIR25a and SfruIR40a were significantly lower in the PG-OV than in the antennae, their expression levels were similar to or even higher in the PG-OV than in other tissues (Figure 7) (*p* < 0.05).

## 4. Discussion

It has long been reported that some olfactory and taste sensilla are distributed on the ovipositors of moths [40,69,70] and that the ovipositor may therefore function in moth olfaction and gustation.

In the current study, we attempted to increase the understanding of the chemosensory roles of the moth ovipositor. To accomplish this, we analyzed the transcriptomic data of chemoreception genes of the *S*. *frugiperda* PG-OV, and also analyzed the expression profiles of these genes by RT-qPCR in different chemosensory organs. Our results provide direct molecular evidence of the chemosensory roles of the *S*. *frugiperda* ovipositor, and also provide a foundation for future research concerning the molecular mechanisms of chemoreception by the PG-OV of *S*. *frugiperda* and other moths.

Odorant receptors (ORs), which are located on the dendritic membrane of olfactory sensory neurons (OSNs), selectively detect volatile ligands in the environment and are the primary determinants of OSN sensitivity and specificity [71]. In our research, a total of 12 SfruORs were annotated. This is fewer than the number reported in the PG-OV of *H*. *assulta* (22 ORs) [41] but comparable with that in the PG-OV of *H*. *armigera* (10 ORs) [41] and *S. nonagrioides* (11 ORs) [72], and more than that in the PG-OV of *M*. *sexta* (3 ORs) [40] and *C*. *suppressalis* (2 ORs) [73]. Most importantly, ORco was detected in the PG-OV of *S*. *frugiperda*. The expression of ORco in PG-OV may reflect the olfaction roles of the *S*. *frugiperda* ovipositor. Two SfruORs (SfruOR13 and SfruOR16) were clustered in the lepidopteran PR subfamily [23,24]. In addition, SfruOR13 and SfruOR16 were more abundantly expressed in male antennae than in female antennae, suggesting that these ORs are putative PRs that specifically function in sexual communication. Guo JM (2020) and Guo H (2022) had cloned the PR genes of *S. frugiperda* for functional studies [55,56]. Functional analyses by the *Xenopus* oocyte or *Drosophila* OR67d neuron recording system demonstrated that SfruOR13 robustly responds to the major sex pheromone component *Z*9-14: Ac [55,56]. SfruOR16 expressed in *Drosophila* OR67d neurons was strongly activated by *Z*9-14: OH, which was demonstrated to be the antagonists of *S*. *frugiperda* sex pheromones [56]. The behavioral responses of *H*. *virescens* to the major sex pheromone component *Z*11-16: Ald was found to be mediated by the pheromone receptor HR13, and PCR revealed that transcripts of HR13 were present in the ovipositor of *H*. *virescens* [39]. We thus speculate that SfruPRs expressed in the PG-OV of *S*. *frugiperda* may be involved in the detection of sex pheromones during mating or the feedback regulation of sex pheromone release.

Although the majority of SfruORs annotated in our study are extensively distributed in the antennae, *SfruOR53* and *SfruOR60* also had high expression levels in the PG-OV. We suggest that these two *Sfru*ORs likely function in the detection of oviposition-related plant odors. *HarmOR60* (76.7% amino acid identity with *SfruOR60*), which is mainly expressed in adult antennae and larval maxillae of *H*. *armigera*, was activated by multiple plant odorants but especially by the larval attractant *cis*-3-hexen-ol-1 in the *Xenopus* oocyte expression system [74]. Whether SfruOR60 can also sense *cis*-3-hexen-ol-1 remains to be determined. Common plant volatiles (such as linalool and *cis*-3-hexenol) and floral scent components (such as phenylacetaldehyde and 2-phenylethonal) had been suggested to be the chemical cues used by moths in their seeking of food sources [75,76,77]. Those *Sfru*ORs that are predominantly expressed in antennae but barely detected in the PG-OV may function in the perception of these odors.

An interesting phenomenon in this study relates to the expression profile of SfruORco. According to the TPM values and the RT-qPCR results in the current study, some *SfruORs* (i.e., *SfruOR35/53/60/*) were strongly expressed in the *S*. *frugiperda* PG-OV, but expression of SfruORco in the PG-OV was extremely weak. HassOR31, which is highly expressed in the PG-OV of *H*. *assulta* (21.25 TPM), showed strong responses to the egg-laying attractant *Z*-3-hexenyl butyrate when co-injected with HassORco in a *Xenopus* oocyte system; surprisingly, the expression level of HassORco in the PG-OV of *H*. *assulta* was extremely low (0.87 TPM) [41]. Researchers have long inferred that ORs cannot function in the absence of ORco [16,18,78]. For example, single-sensillum recordings and the *Xenopus* oocyte model system both demonstrated that HassOR31 cannot function without HassORco [41]. A similar situation was reported in several other studies [79,80]. Research on *Anopheles gambiae* has revealed that some AgORs are abundantly expressed in testes, but that AgORco transcripts are present at a very low level in testes [80]; the same study found that AgORs and AgORco are localized on the flagella of spermatozoa where ORco-specific antagonists, agonists, and other odorants activate flagella in an ORco-dependent manner. We, therefore, speculate that in addition to cooperating with ORco and functioning in olfactory perception, ORs might be involved in other non-chemosensory processes, e.g., mediating cell responses to endogenous signaling molecules.

Gustatory receptors, which are mainly located in taste organs, mediate the perception of CO_2_ and other contact chemical cues [81,82,83]. Seada et al. (2016) reported that four gustatory neuron types in the ovipositor sensilla of *S. littoralis* detect salt, caffeine, sugar, and water [84]. In the current study, we identified four GRs in the *S*. *frugiperda* PG-OV transcriptome. This number is comparable to that reported for the PG-OV transcriptomes of *H*. *armigera* (3 GRs) [41], *H*. *assulta* (6 GRs) [41], and *M*. *sexta* (2 GRs) [40]. One *S*. *frugiperda* GR (SfruGR12) belonged to the clade of putative sugar receptors, suggesting a sugar-tasting function of the *S*. *frugiperda* ovipositor. Similarly, Li et al. had reported the repertoire (HassGR9) of the sugar receptor subfamily in the PG-OV transcriptome of *H*. *assulta* [41]. Sugar-taste sensilla have also been found on the ovipositor of *S. littoralis* [84].

CO_2_ is of great importance for phytophagous insects in their foraging and oviposition behaviors. For moths, CO_2_ gradients may indicate the quality of flowers. Fresh flowers, which may provide more nectar than older flowers, produce more CO_2_ than older flowers [85]. Specialized receptor neurons that detect CO_2_ are located in the labial palps in Lepidopteran adults, and three GRs, GR1, GR2, and GR3, are responsible for CO_2_ sensing in Lepidoptera [67,86]. In the current study, the expression of two CO_2_-sensitive GRs (SfruGR1 and SfruGR2) were annotated in the PG-OV of *S*. *frugiperda*. They were clustered in the lepidopteran GR1 and GR2 lineages. The absence of SfruGR3 may be due to its expression level being too low to be detected. To date, moth sensory neurons specific for CO_2_ have been described on labial palps and antennae; the annotation of two candidate CO_2_ receptors being expressed in the *S*. *frugiperda* ovipositor supports our hypothesis that moths may also detect CO_2_ via their ovipositor.

The sub-family of “bitter receptors” mainly participates in the perception of the large variety of toxic substances that evoke aversive behaviors in caterpillars and moths [31]. In our study, one putative bitter-taste GR (SfruGR30) was identified in the PG-OV of *S*. *frugiperda*. Given that it is highly expressed in the PG-OV, we suggest the SfruGR30 may help regulate the sensing of bitter substances during *S*. *frugiperda* oviposition.

IRs are involved in olfaction and in the sensing of humidity, temperature, taste, and even sound [32,87]. In this study, we identified 10 IRs in the PG-OV of *S*. *frugiperda*. This is comparable to the number of IRs in the PG-OV of *S. nonagrioides* (9 IRs) [72], *M*. *sexta* (9 IRs) [40], and *H*. *assulta* (13 IRs) [41]. Two putative co-receptors of IR families were detected, indicating that IRs may also be involved in the chemosensory process in the ovipositor of *S*. *frugiperda*. Although they displayed high expression levels in the PG-OV, the two putative IR co-receptors had the highest expression in both male and female antennae, which is similar to the findings in other studies [22,88]. Six SfruIRs (SfruIR40a/41a/64a/75d/75p/75q.1) were located in the “antennal IRs” subgroup. Consistent with findings reported for other species [89,90], all “antennal IRs” annotated here were mainly expressed in the antennae. However, one of these IRs, SfruIR40a, was also highly expressed in the PG-OV. We speculate that this receptor may be involved in the PG-OV perception of odorants. Although “divergent IRs” were reported to be the largest sub-group in *D*. *melanogaster* [91], we found only two such IRs (SfruIR60a/100) in the *S*. *frugiperda* PG-OV. According to the RT-qPCR results, both genes were abundantly expressed in the PG-OV, and SfruIR60a was also highly expressed in other chemosensory organs. The functional importance of this class of IRs in PG-OV remains to be investigated.

## 5. Conclusions

By analyzing the transcriptome of the female pheromone gland-ovipositor (PG-OV) of *S*. *frugiperda*, we annotated 26 putative chemoreceptor genes. We then used RT-qPCR to compare the expression of these genes in different chemosensory organs. The high expression of several of these genes in the PG-OV suggests that the *S*. *frugiperda* PG-OV may function in chemoreception. The results should facilitate the study of the molecular mechanisms of chemosensation in the PG-OV of *S*. *frugiperda* and of other moth species.

## Figures and Tables

**Table 1:** Unigenes of candidate chemosensory receptors in pheromone gland-ovipositor of *S. frugiperda*.

Name	ID	ORF (aa)	TPM	BLASTx Best Hit (GenBank Accession/Name/Species)	Full Length	Identity (%)	E-Value
ORs							
SfruORco	DN43518_c2_g2	473	0.01	AAW52583.1|putative chemosensory receptor 2 [*Spodoptera exigua*]	Yes	99	2.4 × 10^−258^
SfruOR53	DN34590_c0_g2	404	3.23	ALM26238.1|odorant receptor 53 [*Athetis dissimilis*]	Yes	78	1.0 × 10^−187^
SfruOR30	DN44157_c8_g3	387	0.55	ALM26205.1|odorant receptor 16 [*Athetis dissimilis*]	Yes	80	1.0 × 10^−173^
SfruOR60	DN36386_c0_g1	392	0.25	ABQ82137.1|chemosensory receptor 2 [*Spodoptera littoralis*]	Yes	98	0.0
SfruOR32	DN41457_c1_g1	397	0.19	QEY02574.1|odorant receptor 5 [*Spodoptera littoralis*]	Yes	72	0.0
SfruOR35	DN38262_c0_g2	453	0.18	XP_022831643.1|odorant receptor 85c-like [*Spodoptera litura*]	Yes	95	0.0
SfruOR16	DN42787_c4_g1	432	0.17	ACL81182.1|putative olfactory receptor 16 [*Spodoptera littoralis*]	Yes	93	0.0
SfruOR45	DN39388_c1_g1	429	0.16	XP_022825109.1|odorant receptor 13a-like isoform X1 [*Spodoptera litura*]	Yes	94	0.0
SfruOR50	DN34812_c1_g1	404	0.12	QNS36220.1|olfactory receptor 23 [*Mythimna separata*]	Yes	72	0.0
SfruOR39	DN33131_c1_g3	385	0.07	QNS36227.1|olfactory receptor 36 [*Mythimna separata*]	Yes	82	0.0
SfruOR13	DN37955_c1_g4	435	0.05	AGI96750.1|olfactory receptor 13 [*Spodoptera litura*]	Yes	90	0.0
SfruOR3	DN36417_c0_g1	379	0.03	XP_022827581.1|odorant receptor 30a-like [*Spodoptera litura*]	Yes	89	0.0
GRs							
SfruGR30	DN41240_c0_g1	357	7.85	QHB15310.1|gustatory receptor 10 [*Peridroma saucia*]	Yes	92	0.0
SfruGR1	DN33391_c0_g2	464	3.85	XP_022828173.1|gustatory and odorant receptor 22 [*Spodoptera litura*]	Yes	99	0.0
SfruGR12	DN30079_c1_g1	396	0.14	XP_022826955.1|gustatory receptor for sugar taste 64f-like [*Spodoptera litura*]	Yes	93	0.0
SfruGR2	DN28721_c0_g1	413	0.03	XP_022814066.1|gustatory and odorant receptor 22-like [*Spodoptera litura*]	Yes	98	0.0
IRs							
SfruIR25a	DN42711_c2_g3	918	5.69	XP_022828195.1|ionotropic receptor 25a [*Spodoptera litura*]	Yes	99	0.0
SfruIR60a	DN43581_c1_g1	585	3.59	QHB15321.1|ionotropic receptor 60a [*Peridroma saucia*]	Yes	80	0.0
SfruIR75d	DN36075_c1_g1	314	2.37	ALM24944.1|ionotropic receptor 75d [*Athetis dissimilis*]	No	73	1.0 × 10^−29^
SfruIR64a	DN38775_c1_g1	603	0.94	ARB05666.1|ionization receptor 64a [*Mythimna separata*]	Yes	80	0.0
SfruIR40a	DN39774_c1_g2	712	0.48	XP_022834254.1|ionotropic receptor 40a [*Spodoptera litura*]	Yes	97	0.0
SfruIR100	DN42291_c0_g1	586	0.19	QHB15322.1|ionotropic receptor 60a1b [*Peridroma saucia*]	Yes	60	0.0
SfruIR41a	DN41275_c0_g1	537	0.07	ADR64681.1|putative chemosensory ionotropic receptor IR41a [*Spodoptera littoralis*]	No	88	0.0
SfruIR75p	DN38967_c0_g1	764	0.06	XP_022816386.1|glutamate receptor 1-like [*Spodoptera litura*]	Yes	90	0.0
SfruIR76b	DN42735_c2_g1	542	0.03	ADR64687.1|putative chemosensory ionotropic receptor IR76b [*Spodoptera littoralis*]	Yes	95	0.0
SfruIR75q.1	DN44064_c7_g1	662	0.01	ADR64686.1|putative chemosensory ionotropic receptor IR75q.1[*Spodoptera littoralis*]	Yes	78	0.0

## Data Availability

The authors confirm that the data supporting the findings of this study are available within the article and its Supplementary Materials.

## Supplementary Materials

The following supporting information can be downloaded at: https://www.mdpi.com/article/10.3390/insects13050481/s1. Table S1. Primers for real-time quantitative-PCR of candidate ORs, GRs, and IRs in *S*. *frugiperda*. Table S2. The amino acid sequences of ORs, GRs, and IRs/iGluRs used in phylogenetic analyses. Table S3. Evaluation of sequencing data of *S*. *frugiperda* samples. Table S4. Assembly summary of pheromone gland-ovipositor (PG-OV) transcriptome in *S*. *frugiperda*. Table S5. Sequences of candidate ORs, GRs, and IRs in the pheromone gland-ovipositor of *S*. *frugiperda*. Figure S1. Distribution of *S*. *frugiperda* unigenes that are homologous with genes of other insects in the non-redundancy protein database. Figure S2. Functional annotation of *S*. *frugiperda* pheromone gland-ovipositor transcripts based on the gene ontology (GO) categorization.
