# Supplementary material for: Identification and Characterization of Chemosensory Receptors in the Pheromone Gland-Ovipositor of Spodoptera frugiperda (J. E. Smith)
Source: Insects. 2022 May 21;13(5):481. doi: 10.3390/insects13050481 (PMC9146910; doi:10.3390/insects13050481)
Supplement: Supplementary file 1 [file insects-13-00481-s001.zip › insects-1715090-supplementary-Table S1.pdf]

Table S1. Primers for real-time quantitative-PCR of candidate *OR*s, *GRs*, and *IRs* in *S. frugiperda.*

ID	Forward primer (5'to 3')	Reverse primer (5' to 3')
ORs		
SfruORco	ATGTTGTCAGATTGGTAGCA	TCTTCAATGAGCCTGTTCC
SfruOR53	CGGTAAGAATGCGACGATA	CAGAGCGAATGGAGGAAG
SfruOR30	CCTGAACTTGGTGTCCTG	TTATTGGCAGCGTTGGAA
SfruOR60	CCACGCATAACCTCAGAC	CCTCATCGCAGCATTCTT
SfruOR32	TCAGCATCACACTCCAGAA	CGCTCATAGAAGATAGGAAGAA
SfruOR35	TGAGAAGCAGAGTGAAGTAG	AGCCAGCGATTATCAGATG
SfruOR16	TTCCTATTGTTCGCATCCAT	CGGCTGTCTACTGTTACTG
SfruOR45	TGCTGATCTTGAAGAATCTG	ATGGCGATGGAACTGAAT
SfruOR50	GCCATCTGCTTCATAATCTG	CCTCATACTGTTCTTCCTTCT
SfruOR39	GCACTTGTCTACGCTCTT	TTGTTCTTCGTGTTACCTCT
SfruOR13	ATTCCAGAAGCAAGAGACAT	CGGCAGATGATGGTAGTG
SfruOR3	TCTAGCAGCCTACGAGTC	TTGAGAACAGCGAAGATGG
GRs		
SfruGR30	CTTCTTGACGGTGCTGTT	CCATCTTCCTGTGCTTCC
SfruGR1	GGTAATCTTCTTCACGACTAC	TCCGAGGTTCACATTCAG
SfruGR12	CGACGGACCTCATTATCAT	AACCAACCAAGCACTATCA
SfruGR2	GTATCATTATCCGCTCATATCC	ATCCACGATCAGTTCTACAG
IRs		
SfruIR25a	CGGCGTTCCAAGAGATAA	GGTCAGTCAAGGTTAGGTT
SfruIR60a	GTTATCGCCGTACCTCAA	TCCATTACACAGCCATTCA
SfruIR75d	GGACACCACCTACAAGAC	TGATTATGTCGTAGCCAGAG
SfruIR64a	GAATGGCGTAAGCAATATGT	GAGGAAGGAATAGCGTTAGT
SfruIR40a	CATTCGTAAGGCTCACAGA	GCATCACTATTGTCAGAGGA
SfruIR100	GAGCGGATAGCAAGGTTC	ACGACCACAACATCATTCA
SfruIR41a	ACCGTATGTGCTGTTAGAC	TCTTCTACAACGCTTCCAAT
SfruIR75p	TCATCATAGGAGCAGTTCTG	AGTGGTGAGTTGAGGAGAT
SfruIR76b	ATTGGCGGAGAAGGACAT	AGTTCTTCGTAGGTAGTATCAC
SfruIR75q.1	ACATAACCAGACACCAGTAG	TGGATCTTGGAATGGAATGA
SfruActin	TACTCCTAAGCCTGTTGATG	TTATGTCATGGTGCCGAAT
