# Supplementary material for: Identification and Characterization of Chemosensory Receptors in the Pheromone Gland-Ovipositor of Spodoptera frugiperda (J. E. Smith)
Source: Insects. 2022 May 21;13(5):481. doi: 10.3390/insects13050481 (PMC9146910; doi:10.3390/insects13050481)
Supplement: Supplementary file 1 [file insects-13-00481-s001.zip › insects-1715090-supplementary-Table S2.pdf]

Table S2. The amino acid sequences of ORs, GRs, and IRs/iGluRs used in phylogenetic analyses.

ORs:

>BmorORco
MMTKVKTQGLVTDLMPCIRLLQAAGHFLFNYHADTSGMNMLLRKIYSSAHAVLIVVHYICMGINMAQYKDEVNELT
ANTITVLFFAHSIIKLAFFAFNSKSFYRTLAVWNQSNSHPLFTESDARYHQISLSKMRRLLYFICGMTVFSVISWV
TLTFFGESVRMIASKETNETLTEPAPRLPLKAWYPFKTMSGGGYVFAFIYQIYFLLFSMALANLLDVIFCSWLIFA
CEQLQHLKAIMKPLMELSAALDTYRPNTAELFRVSSTDKTEKVPDAVDMDIRGIYSTQQDFGMTLRGAGGKLQNFN
AENNPNGLTAKQEMLARSAIKYWVERHKHVVRLVASIGDTYGTALLFHMLVSTITLTLLAYQATKINGINVYAFST
IGYLVYTLGQVFHFCIFGNRLIEESSSVMEAAYSCQWYDGSEEAKTFVQIVCQQCQKAMTISGAKFFNVSLDLFAS
VLGAVVTYFMVLIQLK
>BmorOR1
MLLSFKDDSRSPDIQKPQNFQYMKILRFNLKIICAWPEKQLNEIRSLGHSIHRVILPIQSVVCLACGILYIHFHFN
EIPFFILASTFITVMMNLATCSRTALVMLFERYLVLTGRFITVMHLFNFQKNSDYAYKLCTFVNRMSHFYTLYVLF
SMFMGLGLFNLLPLYNNYVSGAFSDPYGPNVTFFHSVYFAFPFDYSHNFRGYIIMALFNSYVSVTCSIGLVMFDLL
MCLMVMHVWGHLKILSHNLINFPRPKASHVITTPNGPTNVETYTEEESKEVFARLRECIKHYGTVDDFANDMSETF
GVILLVYYGFHQVSLCMLLLECSDLSTKAMLRYGPLTLIMIQQLIQISIIFELLGSVADRIPDAVYQLPWECMDVK
NRRVVYGFLRRTQNPVRFKAMGMLDVGVQTMASILKTSIS
YFVMLRTVAT
>BmorOR3
MIFVDDAVIGIKDPREYRHLRVLRTSLRLLGAWPGHYLGEETGSKYECAPMFLLMFIKIACLYLTIVYLRNNADVL
GFFELGHVYLTIFMTFVTLSRGFSLTWNPNYHKVVKKFITEMHLLYFKDNSEYAMKTHRRVHKISHFYTVFLKVQM
IAGLTLFNVIPMYNNYRQGNYASDRPANITYDLSIYYETFDILNTPNGYIFICVFNWFASYICCSFFCSFDLILSL
MISTVSGHFRILIHNLLTFPLPEAITASKKFVDKHRCNGNRSEFVLEEAKLYSPAEMWQVTDRLRQCIDYHRKLVE
FTGDISEAFGPMLFVVYYLFHQVSGCLLLLECSQLNTAALVRYGVLTVVLYQQLIQLSVIVESVGTVTGRLKDAVYE
VPWEYMDTSNRKTVAIFLMNVQEPLHVNALGLAKVGVQSMAAILKTSFSYFTFLRTVSE
>BmorOR4
MFKIIKNIIVENDALKQVEKPQEFQYMKWVQYHLKYIDGWPNMDMNKKNVSKIRFHKRHLLVVEQTITFLSQMFYI
VKNYGKLSFFEIGHSYITALMTIVIFSRSVVTALGRYRKIARYFVSSLHLYHYKDISEYALQTHLLVHRLSHYYTV
YLISLVVTGMLLFNITPLYNNISSGVFNSPRPENMTFQHAVYLGLPFDYTTDIKGYFVVFILNWHLSHIAASYFCT
FDLFLSLLILHLWGHLRIILNNLKTFPKPYTNNSMYTEEENQVVLLKLQECIRYHNFIISFTVMMSNVYDVVIIVY
YLFHQVTGCLLLLQCSTLDWESLSRYGPLTLIIFQQLIQVSMIFEILGFLSDKLPNAVYSIPWEAMNVTNRKLVQV
LLQKSQKPIQFKAMNMMSVGVQTMASIIKTSISYFIMLRTIARD
>BmorOR5
MLLYYPNTQVKEKVNNVEEFTYIKFLKSFCKIMDFWPEREEKNSKTRIFRLRYILVLQFCFTLVAGVLYLTNSVGK
QTFYDLGHTIITVLMNVVSLSRLILRCFKKYDVVGQQFINKIHLYHYRNDSEYAMKIHTVVHKISHNMTYIFSFCI
IFGTVTFNLTPIFNNIGSDAYKNPRPDNVTLQQCVYYALPFDYTGNFKWYLLVAIFNVQKTFFCTSLFILFELSLS
LMIICLWGHLRIFIHNLNHIPAPRNSFEYTKEERQEVDDTLKKCIQHHTLIIGFVRIMSETYGLAVLIYYAFQQVV
GCLLLLQCSQMELKTVTRFGFLTLVLNQQLIQISVIFELLGYMSDKLQDAVYCVPWEYMDTSHRKMVYMMFRQSQI
PLQLKAMNMLSIGVKTMVSILKTSVTYYLILKTVTTD
>BmorOR6
MKEEYYLQHPRTQLFYKVLAHVSTIESTIDLTWWGYTFPKYVGWFYHLQCNVVRLFGKCVVVSQILFIILNYQTID
KSVFIIAITITPLGALVGIKAESAKAECYVNLMKNFMDKVHIHSIYRKNENNEFVKKKVIQIERVSRFTAYFLVIL
IAINCLSWMLKPTLHNIKHFEEIMNKSMEFQYYIYFWTPLDYKYNLRDYIIIHTLCIYLGATAVTVIVTFDIFNFI
AVFHVVAHIQILKNNVKSNWSDDFNESEKKGYLVSILEYHAYIIRIFGEVQSAFGLNVASNYLQNLIEDGLFLYQI
MNGEKENVLMYGLMIILYLGGLIFLSIVLEEIRRQNYDLCEYVYALPWEGMSLENQKIFVVFLQRTQPDLEFETVC
GMKAGVKPAFSIVKSMFSYYVMINSRF
>BmorOR7
MLLYHPNTQVEEKVNNVEEFTYMKFLKSFCKIMDFWPEREEKNSKTRIFRLRYILVLQFCFTLVAGVLYLKNNFGK
KTFYDLGHTIITVVMNVVSVSRLILRCFKKYDVVGQQFINKIHLYHFRNDSEYSMKTYKAVHKISNNMTYIFSFSI
FVCVVTFNLNPVFNNIGSGAYKNPRPDNVTLQQCVYYALPFDYTGDFKWYMLVAIFNVQKTFFCTSLFILFDLLLS
MMIIHLWGHIRIFIHNLNHIPAPRNSLEYTREERQEVDNTLKKCIQHHTLIIGFVRIMSETYGLAVLIYYAFQQVV
GCLLLLQCSRLDLKTITRFGFLTTMVNQQLIQISVIFELLGYMNDKLQEAVYCVPWEYMDTSHRKMVYMMFRQSQI
PLQLKAMNMLSIGVKTMASILKTSVTYYLMLKTITANEA
>BmorOROR8
MSLSTRCLLKDFCKYVYYAGAGNFWYEDIYKETVPYKMYVVISFFTYTVMIFLENLAALFGKLPEVEKNSAVMFAA
IHNIVLTKMFLLLYHKRSISKLNCEMAAVGENLEEASIMRRQFRKMRLGTALYFISVYLSLVAYGVESARRTIVEG
APFYTVVTYLPDYDNTTVLASFLRIFFYITWLYMMLPMMSADCMPIAHLITMTYKFVTLCRHFDQIREKFQINVKI
MAKTEATEILKLGFIEGIKMHQKLMYLADEIHRVFGIIMALQVCESSAVAVLLLLRLALSPHLDLTNAFMTYTFVC
SLFLLLALNLWNAGELTYQASLLSNAMFYSGWYFCDFEKDWCRDIRRLVLIGCAQAQKPLILKAFGVLDLSYETFV
SVARMTYSVFAVFYKRGD
>BmorOR9
NVDNVEDFKYVKWLRNHLKTVDAWPVHSKSKRKIQKRYVLPIFSAACFISQTVYLKNGIGTLSFVVLVHSYICFLI
NGSCLCRGILIATERYKRLATCYLKTVHLFHHKNRSEHAMKIHVIVHRLSHYYTIYLISLVFVGMVLFNFMPIYNN
INSGAFKSPRPENVTFQHAMYLALPFDYTTNIKGYFVVFILNWYISLVTTSHFCTFDLFISLMIIHLWGHIKILMC
SLEDIEGFVPGSSFKFTIEQNRKIYLILQECIRHHQFTIDFTNEMSSTFGLVILFYYFFYQVSGCLLFACMLTNES

LSRFGPMTFILFQQLIQLSIVFELISSLSENLPNAVYNVPWEFMDKNNRKMIQVLLLQSQKLIQFKATSMMNVGVQ
AMATILKTSVSYFIMLRTMYQEH
>BmorOR10
MRTNAKSFLFVPSKVLTLCGVWPVEKTSIFSLIYRSIMLSSQFCFLVFNGIYIGLMWGDLKAVSDALYMFFTQTTC
CSKAIGFYFNFMKIKRIVASMDDVLFTAMSIEDQATIFSHSRTVNKLYKGVLGFTGFTLVQWTVLSLIGSGRTLPF
NEMWVPTDISKSPNYEITFVVELWMMVISAALFMSVDTITVATMMFSCAQLDIIMKKTQQIQEIPLSPDLSSRNRS
ELHEKNNGILIDCIKQHQAIVRFSELCEGTFQVHSFFHLGGIVFMICVIGFRMAGESPVSAQFWAALSYLVIILGQ
LYLYCWCANELTTKSEQLRDKLYLTPWYDQDVKFKRNLCIAMECMAKALTFRAGSYIPLSRAMFVSILRSSYSYFA
FLNQANEQ
>BmorOR11
MDEHSHFETSLNKIKVLFKYSGMNLENTVTNTYEFLNHRWVYILNHAWTLAAVTFICIGISNGQNFIEMTCIAPCV
AMTVLAVSKSFFHYINENAVKSLLENLIELERTDFERTKSVQRTEIVATEKQLLNMVINVLYVLNCSMILVFDMTP
LIIIAIKYWTTNKFVRLLPYLDIFVFVPYKFEYWVMAYILQIWAECIVLLFIGAADCLFFTCCTYIRIHFRLLQYD
FERLTSSRRESDGLRDDEDFRETYTNLVKRHQGLIESSSILEMIYSKSTLSNFVLSSLVICLSAFNVTVIVVNDVT
IVMTYLIFLAMSLMQVYFLCFFDMLMSASEEVGNAVYNCSWYTEKASTGKDLLFTITRS
>BmorOR12
MTRITDVFSLNFIFWKFLGLWGKSAPSKYNMAYTVFYLFASLFVYDIFLTLNLIHTPRKLETLVRETMFYFNHLVA
VTKILMMFIMRKKILVIFDLLDCEEFKPNDENSQEIMKRKTDFYYIYWRIVAVTSNLSCFMLVIGPLIKMLIWKIE
LGLPVCKFYFMSDELRNKYFVIWYIYQSFGIYNQMVNNLNLDTFNCGMLWMAVGQLQILKTKFVNLKLNDFENGLD
LKSRDDMQIERLRKYLTHYEIILKYCATVQDILNITIFVQLGMSSIVICVGLCGFVAMPSNTETAIFMSSYLITMT
MQIFVPSWMGTQISFECGELMSAAYCCEWIPRSKLFKRSLILFVERAKTPVRITGLKIFTLSLDTFTSIMKTTYSF
FTLIRQLQVDEVN
>BmorOR13
TSRPYKFFNILRLCTMAPKQIDCFEINWKFWKFLGIWSENKPHRYYKYYSKIFITFFVILYDVLYTINFYFVPRQL
DLIIGEMLFYLTELSVLSKVFTFIIMRHKLKIIFEILESDAFQTDTEEELKILHRAKVFIKRYWKIVALVSITANL
THISSPLLKNLIFKVELVLPVCSYSFLSESFLKTFEYPLYFYQIVGIHFHMLYNLNIDTYFLGLMILIIAQLDILN
VKFRNLKSGKDHTQLNESIMGLNKNLDHYNEIERCVLKHDYYYNPIPFYFNKQQTSRICLICILQSVPVEYYIFLA
TYMFIMIIQIMVPCCFGSRIMDKSILLSSAIYNCDWTSNSKDFKINMRLFVERANKPLSITGGKMFSLSLATFTS
>BmorOR14
MSNYIFKPFHETYRIITFTMIAAMIYPNPATEKRRLIYIGLMLLSVIPLAFMIVTEMYEFFMASDLNNTIRHSTVI
GPFIGGFVKSPFFEIMFVYTFFSSFIYIINYVGYDGFFGLCINHACLKMKLYCRALEDAMRSDSRRHEKIVAVIEE
QRRTYEYIALIQDTFNIWLGLIYVATMIQMCTCMYHIVQSFNIDVRYIIFVISIIHIYLPCRYAANLKCMAAETPT
LIYCCGWESVSDLRIKRMMPFMVARSQVIVEITAFNMFAFDMELFVWIMKTSYSMFTLMRS
>BmorOR15
MMTLVYQTDIFKPNVFFWKMFGIWADRKSSKTYKYYSFVFLFITLIMYNSLLAINLLYTPLKIELLIREVIFCFTE
ITVTTKVLMILFKRNKILDAFDLLNKNEFRGNSEESSAIIQKNNSAYKTYWKLYAILSNFAYSSQVLGPLIVKLIW
KTKLELPICNYYFLNEELRHDFFSGWYIYQSFGMYGHMMYNVNIDTFISGLLMMAVTQLKIIQTKLLSLKLNPRER
KMDRGLMNITEVLKLNEILKHYELVLKYCSTVQSILDVAMFVQFGVASAIICVAMCGLIMVRSSTETLLFMVTYLF
AMTLQIFVPAWMGTQLHFQSQELVFAAYNSEWIPRCQSFKRSIIIFVERAKIPITITGLKMFPLSLATFTSIMKTA
YSFFTLIRNMQTLQEE
>BmorOR16
MPVSPERSPHYHLGYSFQLVTICMSAYMYFGVDSVAFSSVIFGCAQIGVIKDKIMSIKPLGIYRNHKTYTNISRYN
RKTLIECVKHHQAVISFTELVEDTYNSYLLFQLVGSVGIICGLAQCPITIPIAILCYLSVMISQLFVCCWCGHELS
ATSEELHTILYNCAWYDQDVKFKRDLNFMMARARRPILLRAGYYISLSRQSFVSILRMSYSYFAVLNQTNK
>BmorOROR17
MREDKMEINNSQKFYTKMIFRYLYSVGLGDWWYQHEDRSDSHRKLYCLWAVISNAYIFLNICNELLANFRKDLTDV
EKNDAIQFSFAHPLIFAKIASFFFNRKKIREVFGRLLEENRSVYSCGELEKESMKQIKRYSLAFIGVSYMTLVMST
IDGLRAHFKEGIPIRTEVTYYPSPSNSGVIVNILRFLVEFHWWYIVSVMVAIDSLAVASFVFVTFKFKLLQRYFKD
MGLTVRRDQSNMTDEALADKFRRDFIVGVKLHENALWCAENVQKAFGWVYSVQVFETVALLVMCLVKLVTTNHNMI
FLLANFAFMLCVIILNGSYMMPAGDVTYEASEVPTSIFLCGWELVRQTDLRFLVVVAIQRSQVPVIMKAFGIMTLS
YSNFIAVSLFKFYVQFQINLF
>BmorOR18
HFFDFNIKYLFYVGLWPSNEAKRIEKIAYKIYEYQLHVLSLIFLVTTGIGTYKNHKDIIALLTNLDKTLVAYNFVF
KVIVFVYKREELRKLIEQIVQSGDQITEDRKALMAKLVIVLTGISTVIITAFSCLALFEGEMTIDAWMPFDPMKSK
MNLFAASQILAATFVVPCGYRAFAMLGIVCSLILYLRDQLVDLQNKIRDLRFATGNVEKLRDDFKLIVKKHVRL
>BmorOR19
MHEFVINVQNETTKLYDQLNIILYILGLQGIWVDEIKLSRRFHVFFKVVTFILHIMCGMFAGLQFFAIFTQNSLNS
QQKSDVIVIGISNPMAYIFCINFIRNRNEIKDLFYHLAVVLKIYYNDVEIEKSMVNKIKSYLSTYVFASITILVSN
GIIAFFQTINSDEPFLGIITAWPDKTDTSKTASYARIGFYLFWCIHFFRISTVFAVIVCILISIKYQYKFLCSYFE
SLNKIFDDETSSHEVKEAEFENAFCNGIKIHTQIIWCVRRCQIMCRTVFSANIMLDTFVLVILMLAMVNSENDFYG
LCSQMSSVLVTVVLMAFFMWTAGDINVQASQLPDAIYGSGWYNCRGKSSARIRSLVTISMNKAQQPILMWALGFVE
LSHKNFVAIIKSAYSVFSVFY
>BmorOR20
MIQASKYPNSKTKELFRKIAHIAYICGLPNFWIEELNLPKSFIRVYDKIVRIFNVATYFFLGIEIAAHFTQHHLTN
KQKFDLLLYSISHPILNGYGVIVSRQVGNVKKVLLDLIVNLKVKYNDPVIEEAMIKISMTYSVSFITNCVLSMLTY
TFDALLMVYKKGVTFNVIITAWPDVEDTTTEASIGRIGFHIFWWLFVTRPFAVYVLVINLTTCLSHQYMNLQSYFF
HLEDIFKENLSQNEKEAKYEAEYKIGVMLHANTLRCTRRCHMVWNGVMSGQIIFNISLIVIIMAQMMPFLSAPVSS
CGMLVTSQFRSVHRFYASRLATAMYCSGWQNCRGKSSVSIRNMVMNTIAVAQ

>BmorOR21
MNKNMNKNHYILKTYCDKIFLVGSGNFWYQKTESRNDKTLLYKIYSCVLFFTYGFMTVLEIMAAMMGDFPEDEKRD
SVTFATSHTVVMIKFISIIKNKELLKTLNRKMMMICEAHEEQTLMDEMYRTVKINVVAYCVAVYGSATFYVFEGLR
KFYNGSHFVTIVTYYPSNDDDTLAATIVRIATTLVLLMMLLTMIISVDTYTMAYLIMYKYKFITLRHYFKRLRENV
DELVAAGKARLAAEKLAQGLVEGIKMHNELLSLSKDIDKAFGTVMALQLCQSSGSAVSLLLQIAVTMYLLLALFLC
NAGEITYQASLLSDEIFYCGWHKCNSPVLSTQRNIRDIVLIAILRAQSPLVMKAFKMVVRSTYSVFALFYAQNK
>BmorOR22
MNKNMNKNHYILKTYCDKIFLVGSGNFWYQKTESRNDKTLLYKIYSCVLFFTYGFMTVLEIMAATMGDFPDDEKRD
SVTFASSHTLIMIKFISIIKNKELLKTLNRKMMMICEAHEEQTLMDEMYRIVKINVVAYCVAVYGSVTFFVFEGLR
KFYDGSHFVTIVTYYPSKDDDTMLASIFRIATTLVLLVMMLSMIISVDTYTMAYLIMYKYKFITLRHYFKRLRENV
DELVAAGKARLAAEKLAQGLVEGIKMHNELLSLSKDIHKAFGTVMALQLCQSSGSAVSLLLQIAVTMYLLLALFLC
NAGEITYQ
>BmorOR24
MPEELFLDRSIKKIESYFRWMGINIRSGDNNNKKDVFKIRCIYFINFVLLNTDVLGAIFWFRSGLEQGKTFTEVTY
NAPCLTFSFLANFKMLSLIFYEKTVHELIAALQKLEIKHFLRQNCAEELKMLKDEKNFLHAVFKGSKIVNYASILT
FGCSPLVLIASNYYKTGRMDYLLPLIVLYPFDVDNITVWPIIYVRQIWSVITAVIGVCATDYLFYTFCVYISTQFR
LLGHSIERVVPNNGLSVRTRLNGNLRMKFVENLKWHQELIRAASLLEQIYTKSTLYNFVTSSVIICLTGFNVAVVE
DFAVILSFLFFLFMSLLQIILLCFFGDKLMKSSTNISDAVYNSKWYLTEKNVGKVLLMVQIRSQRACRLTAYGFAE
VNLRAFMKILSTAWSYFALLQSLYSSHE
>BmorOR25
MFEKALRSANFYMRVIGIPTDIRDGNRTLMERLRNRWFYCINFLWLNTDVAGEITWFVKGLLNGSSTLIENTYLIP
CLTLCILGNVKTFFTIKYANHIIDLVAILKDLEIKNNAARKNETEIVKERLKFLTTSNKFLLFVIGTGIIAFGIGP
LMLTASIYFSSGDMKLKLPFLIWYPFDSSDIRYWPFVYVHQVWSACIACCAVYGPDCFYFTSCTFIHIHFIHLQND
ITNVIVESSRARKNGLYRGCHQAFLELTNRHKDLIRCVNLLEIIYSKSTLVNVVSSSLLICVTGFNVMVTFCWFAA
PFASFLALGLVQTYLLCYYGDTIMCSSTEVSDAVYNSTWYGTNISQMRDYLFVMKRAQKPCKLTAYGFSDVNLRTF
SRILSTAWSYFALLITIYRGNGQQ
>BmorOR26
MSTGSAAGDSVAPHLRRLRQVGFCQLDPTSQSRRPILALMHRVYHRLVLAATVLYIFEQLTYAYQARNDMERLSRV
LFLMLCHLTCIAKQFVFHSDADKINQLVVGLDDALCNQPVETHRLLLLETSRRAARLLMLYSGCAVSTCILWAVFP
LLDQLRGRTVEFAFWIPIDYRHNAFQFAVVLAYAFYSTSLVAVANTTMDAFIATVLYQCTTQLRILRMNFESLPER
AYALSRKTRQDYHTVTHELLVDCLLHYKKITETCNLLEQIFGKAILVQFGVGGWILCMAAYQIVDLSVLSIEFASM
ILFISCILTELFLYCYYGNEVSTESERLVTSIYSMEWVGARLGFQRGLLVLLERARRPVRPAAGLVIPLSLQTFLK
IIKSSYTFYAVLRQTK
>BmorOR27
MPSSFFLPNLENPDYPSLGPTLKGLKYWGMWQSGGIKRILYNSIHAFATFFVITQYVELWIIRNNVELALRNLSVT
MLSTVCVVKAGTFVCWQKYWSGIIGFVSNLEKEQLSKNDAATQAAIVKYIKYSRRVTYFYWSLVTATVFTVILAPL
VGFLSSPERELIANGTLPYPEIMSSWVPFDRSRGFGYWVTALVHTLICFYGGGVVANYDSNAVVLMSFFAGQMKLL
SINCSRLFDDGNEVISNNEAMKRIKECHYHHVYLVRL
>BmorOR28
MHTLALVFALLYPSNCNIIKRAIGITLIIALSGGQLFWCMTYTFKCVYELSIFNFARNNSSNNLFFFCSSVIFKTM
KYCVILVKISRHFLKGNNDLGEDYKIVYKEYNKTHDFKRLMVHEMKLKYFEDIQSVGPPFHCMFAYNFLQVCVLIL
NYSGFDGSFCIASIRLCMKLKLVVYKVQKAFAESKSVSELKHQLNDAIKDNLDALKFHEQIQNVFFIALSFDVKFS
SLCVFGTLLVFLPCHYASNLTK
>BmorOR29
MFDFLQNLEDSERPLLGPNFWLINKTGLLLPKTNFGKLAYILVHEIVTFFVVTQYVELYVIRSDLDLVLTNLKISM
LSIVCIVKVNTFVFWQTSWREVLEYVNEADKFERNQTDETRGKIIETYTKYCRRLTYFYWSLVFTTFLTTTNTPLM
RYWSSPIFREHLRNGTEDFPHIFSSWMPFDKNHSPGSYCTIVWHVLLCAYGAAIMAAYDTCIVVIMVFFGEKLNLL
RERCKKMLANDLYNHAFVIGQLHDIHVQLI
>BmorOR30
MSVSNLKFEVLFKPTTMSLHMNRSHPSIKRNKIWLLQFISLMTLTVFCATGLITSLLFHDLKFGKYMEASKNGTIA
MLSFTTTFKYSLLLYLQKSLNRLIAKIDMDYEIAKGLTPQEKAIVLNYAKKGVIVSKFWLFTAFAITFCFPLKAFI
IMGYRFFIKNEFRLEPMFDMTYPEPIESYKTSFPVYFILFVVFFLFGCYASSLFVAFDPLVPIFVLHACGQLDLLS
LRITKLFSDTKNPRIIAKELKVIISKLQELYGFVNFIKVNFSILYEYNMKITTISMPLSAFQVVESLRRGEFNIEF
TYFFFGCILHFFMPCYYSNLLMERSENFRFAIYSCGWENHNDKNIRQMLLFMLTRATEPLGIATVFTNISLDTFAE
MCRQSYTIFNLMNAAWA
>BmorOR32
FRKSLRGSQEEPRANGKSENVRFLINSHILHCGLRFNETNCHTHYIAKVAIFCFIVTYMLQVMELYWSKGDQEKLF
ECFSILSFCGMGVMKLVILRVYHQRWRFLLNQVSILENRHLDPGPLSYDSDNDNDDNEIVTFITKYTDKFKRTSSI
LIKMYASTLVIYVLSPFVEYIFRQFRGDLNIAYPHILPAWTPLDEFSVTGYLIMVSFETVACIYCVFVHVAFDLTC
VGLMIFACGQFYLLRYRSERIGGKGRICRLLKSTEVRAHYRIVFCHGIHVLLV
>BmorOR33
MIYYRKCKMELNFDKIFKIAIISQKFSGTYPYTKRDKKWATHFILMHGELTIICMLFIYNIIEFDLKAADYSQMCR
NMCLSFVYMVITLLYINMLYYQSKLKMLIETMKAEYELAKTMSEEEQNVILEYAKKGRWLCRAWAILTTCGMAQFF
LKSIVCTIYSAIQGNFRIVQYYEVICPEVIERHRNNPVIFITLYFCTFFYSLYTSALYTSVLPLGPIFLLHGCAKL
EIVRLNIKNLFDNDDYVVQERLKKTVLQMQDIYCYSHEINECFQILYEFLLKATSLVLPITIFAVIQALGRGQFIP
EFFAFIFGAFMVGTTPCYYSNMLMEKSEDVRMTLYSCGWETRFDLNTRKCIILMLCRALRPVSIRTIFRSVSLTTL
TDVFQQAYALFNLLNAVWN
>BmorOR34

MIYYRKSKMELNFDKIFRIAIISQKFSGTYPYTKRDKKWATHFILMHGELTIICMLFIYNIIEFDLKAADYSQMCR
NMCLSFVYLVITLLYINMLYYQSKLKMLIETMKAEYEIAKTMSEEEQNVILEYAKKGRWLCRAWAILTTCGMAQFF
LKSIIVCTIYSAIQGNFRIVQYYEVIYPEVIERHRNNPVIFITMYFCTFFYSLYTSALYTSVLPLGPIFLLHGCAK
LEIVRLNIKNLFDNDDYVVQERLKKTVLQMQEIYCYSNEINECFQVIYEFLLKSSSLVLPITIFAVIQALGRGQFI
PEFFAFIFGAFVVGTTPCYYSNMLMEKSEDVCMTLYSCGWETRFDLNTRKCIILMLCRALRPVSIRTIFRSVSLTT
LTGVFQQAYALFNLLNAVWN
>BmorOR35
MKLWQSIREFGLEYCDLPTTLQNVASLLRAITLNIDSRHTARIPFICYVMTVVITLSYFYVFLVSMAWFVFVRSAE
TRDYLAAMVVLSLGISSEIGTLKFFYTFIYIKKVQRIVREYLECDHMVVPESRFADNVLKTMRNVKKRAILYWVVV
IGNGVVYVTKPLFMSGRHHMEDRYIVYGLEPMFESPNYEVAYFLMMFGLCFICYPPANVTVFLIVVVGYTEAQMIA
LGEEMLRIWEDAVAHYNNKYHTVGALTNSSEKNKIINQYVKFRLTEIIKMHTTNIQLLRQVEFVFRSAIAMGYVFL
VLGLIAELLGGLENTYLQIPFALIQVLVDCYTGQKVMDASSLFEQAVYDCKWENFDKSNMKTVLLILQNSQKSMRL
SVGGITVLGFSCMMSVMKSIYSAYATLRTTMS
>BmorOR36
MVFNSKKNIISLFSLLEDSRHPSVGPHLRLLSLTGIWYPNSKTNITLLKRACFYVIVLFFVSQYLKCIIKFKIDSL
QLILEYAPFHMGIVKTCFFQKDYNVWQDLVSFISKTERDQIAKKDPKSIKTIQSYISRNRKITYSFWALAFIANIG
VFSKPYQNNQSDVNGTVTYNHLFDGYTPFSEEPPGYYFSMGIETILGHVVSFYVLGWDTLVVSIMIFFAGQMQMSR
LQCSRMINGSPERTHKNIIKCHKFHTDLIKYQKQFNSLISPVMFVYLFVSSINLSVCIVQIAEIEDDFATVLSSFI
FLLACLIQLLLFYWHSNEVTVQSELVSYSTFESNWTSTQNKLQKEVALLGLTTSKTLVFTAGSFNHMTLATFISVS
LTFELFELID
>BmorOR37
MELGCSRHLKLPCSLHPIGISKHGNTLSELLIYFPAIPKITYAILAVLLTVYYYIYLCSITWFVFVRCPQTGDLAA
ASIVFSLGVSSEIGAIKLFIIAKLRDITGEYLQCEADMAPGRLRARVGRSLRTVRRRAFVYWLVLVVNAFAYDLMP
AFLPGRHLSEDVFVIYGFEPMFESPNFEIASTLMGVSVVFICYTAGSISAFLIVIVGYSEATMLALSDEISCVWDD
ACASECQQPNDFIRARLGKIVAIHTKQIRLIREVEVVFRGALAGGFACVAFGLIAALLGGLENTFLQLPFCVIQIS
VDCFVGQRLRDANVAFETAVYNCKWEYFDKSNMKTVLLILQNSQKTMGLTAGGVAALDFTSLMTIFKSVYSGVHHS
QTDD
>BmorOR38
MNLSQSVNEQANEYVKMRLERISKIHSPMLPFEDIQDFRELCCIPLAVYAVTGSITASYVYAFLISLLWFLFARCT
DPEDFQVAMVVFSLGISSEIGSTKFFNSIIYIKELRKLFKDYLLYDATCPAQGRLRLHLLTTLRYVKRRAIIYWLV
IIGNGFIFAIKPLLVEGRHLAQDDLVLIGLEPMRQSPNYEIAYAIMTMGVCFICYPPAHVTMFLIIIVGYTEAQML
ALSEELKHLWNDAIEHYEKHSRTEREADAAMKSKILNSFVNFRLVQIIKSHSTNVNLIGRVENVFRGSLAVGYVFL
IVGLIAELLGGLENTYLQVPFALIQVAIDCFIGQRVNDANIDFEKAVYDCKWENFDKRNMKIVLLLLQNAQKTVSL
SAGGIAKLNFSCFMSVIKSIYSAYTTLRTTMK
>BmorOR41
MMGNSTDLFLDRTKSILNFFAMWRSFEKPIPLKVYMAFIMTTQYLFLIFEIIYIVNVWGDMAEVSEASILLFTQAS
VCYKITSFISKTNNFVILLGLIESEIFSAQTELHEKILILKARKIKRLCMFFLVNAVTTCSLWAVIPLLDISSKML
PFKIWMPASTGESPHYELGYLYQMITIYISAFLFIGVDSVPLSMIMFGCAQLEIIMDKIGKVKSRPLDQQPMQRQA
VLNSNYELLVECVRRYQSVVRFIELTEKTYHANIFFQLSGSVLIICNIGFRIAIVDSNSLQFYSMLTYLVTMLSQL
FQYCWCGHELTIRGEELRETLYQSPWHEQDIRFRKVLIITMERMKRPIIFKAGHYIPLSRPTFVAILRCSYSYFAV
LNRVRNE
>BmorOR42
MDIPKFEELLKQIQMNFWLMGIPFDNPKIQIRYYVLLLTLSLMLIDEIAFFGSRMSSENFLELTQLAPCICIGVLS
VLKILALTAKRQKIYELTQNLECLHKIILNDTRKTELVRKNLVLIKFITKYFFVLNAVLIFVYNFSSPVIIAYNYI
VSNEVQFVLPYAVLLPFKTDSWIPWLIVYVYSIFCGFTCVLYYATVDVLYCVMTSLVCNNFSLISFKLQKVNRNTA
HLLKEVVKEQQYVLKLAEDLENIFTAPNLFNVLIGSVEICALGFNLMIGDLTQIPGCILFLSSVLLQILIMSVFGE
NLISESSRIAEAAFLCKWYEMDQKSKKTILTIMIRSHKPKKLTAYKFSIISYGSFSKIISTSWSYFTILRTMYTPP
GTKFQDDL
>BmorOR45
MKVLDNVNHAVKVTMNCCRLYGLFVSDDLTKRQLIIMRAFSLMLYLFFVGFFITTQSALIITMWGDLNLMTNVGLV
LGTHLTLSAKVFTLHYKEKEITNVIYKNEVRLRAETREQGKYIISEMNRETTLFMRLFIPFGMGTVTAWLLCTPKG
ELYTPAWYPCNTTKSPAHEIILAHQGIAVILTATLEIAIVLLMTSIVAVCRCRLKLVGLSFETICDDLPSNIMNKL
TADEQVIVAKRVRENVIEHQAVLECINDIQDCFSSAMLVHIAISTMIICATAYQLAVEKSLDLTQRMTMASFLGGM
STEIFLFCYQGGHLSIDSMEVATAVYSCPWYTFPTSLKRSLLVIMIRAQQPALLTAGGFAPLLLDTFVSIMKASYS
FFTVLQNASE
>BmorOR46
AATYVQIADLIDIWGDLDLMAETSLLLFMELAVISKILTLIFKYDKIMEIINGTEDILCSENRLEGQKIIASIDKE
TTRFFQYYTSSVIFTTFFWFLGEHSSTFFIRAKYPFNELKSPGYEFALIHQCMMMVFTGYFEFNINIFFASVVAGC
RCRLKLVALSLRNICINIPVNKKNLITPEEEKLITERLHCAISQHKYALDAAEDVKHCLSKVLLVQLTVSIVIICT
TAYQMAVVRILYWWQEKINYYASLTMAGYLFGTSLEVFLFCYQGEFLRESSEEIADAAYECPWYTLTRPLKKTLLI
IMTRAQRPATLTAGGFVTLDITEYMAIMKASYSFFTVLQQVSE
>BmorOR47
MKLVFDNFIFALKVTLNWCRYFGIFIPDELTGRRQKLLVQAYSVFMFMLFIGFFIITQIILFILVWGDLSLMTDVG
LVLGTNLALSAKIAVFFFKREELASILKKNDDTLRFETREEGKKIISEIDRETNAFMKVFFCFGVGTVIAWFLSTP
KGELHIATWYPCDTKRSPAYEIIMIHQLAITADLLMLSMIAVCRCRVKLVGLYLQTICDDLPCNVKNKLTSDEEVI
VAKRIREYVIEHQAILDCISELQNHFSPALLVQLLTSVVIICVTAYQLAVEKSSDLLRKFTMASFLFAMSTEMFTF
GYQGGHLSHDSMEVATAAYSCPWYTFPTSLKRSLLVIMIRAQQPALLTAGGFTTLSLETFVTIMKASYSFFTVLQE
ATD

>BmorOR48
AAACVQIADIIDIWGDINLMAETALLLFMEFAVISKILTLLLRYDRIMEIINGTEEILYFENGLEGQRIIASVDKE
TTRFLQFNSAFVVLSTTFWFTGEHSSTFFIRAKYPFNELKSPGYEFALIHQCMMMVFTGYTVFNINIFFASVVAGC
RCRLKLVALSIRNICINIPVNKKNLITPEEEKIVKERLHCAISQHKCALNAAKDIKNCISEFLLVQFTVSIIIICT
TAYQLAVVCLFQNKAIGNIQKTSMFGYILGASLEVFLFCFQGEFLRNAVRDCEEIADAAYECPWYTLTQPLKRTLL
IIMMRAQSPVILTAGGFIDLSIREFMGILKASYSFFTVLQQVSE
>BmorOR50
MPSLLKTESLALTLTLNTLSWAGLILRDDYTKTQRIIMKVYGGLVFLYLFVFTAYVQIADLVVIWGNIDFMTETSL
ILFMQLAVSAKVLTLMLKSKKIMEVTNEADAILNSEKKVEGQRIIASIDKNTTLFLKYYGFFVAFTIICWFMGENT
STFFIRSKYPFNELKSPGREFAFVHQCIVVIFTGSFDFNVDIIIISLVAVCRCRLKLVALSLRNLCLDIPMNKRNL
ITSDEEKVITERLRNIISQHKRALDAAEAIKHYLSGALLVQLMVSIVVICTTAYQLAVKKSTTMQSLTMAGYLFGT
SLEVFLFCYQGEFLRESSEEIADAAYECPWYTLTRPLKKTLLIIMTRAQRPATLTAGGFVTLDITEYMAIMKASYS
FFTVLQQVSE
>BmorOR53
MALKKMLALTKGLEDPTHPLLGPTLKALSVFGLWQTGSQKSTVIYNTFHFLTFLFVITEYIDLYTVRKELSKMLNN
LSVTVLSTICMIKTLSYVCRQSHLKVLVREISELELELMKTTDKNIVKRLRQYTVYTRAVTYVYWFLVVGINVVLL
TSPLLKYASSEIYRSEIKNGTEPPPLILCSWFPFDSARMPGYFWATMVHIIMSIQGCGVVATYDMNAVAVMSYLKG
QTSILKDKCKAIFDETASSRDVLNRIRDCHRHHNILLRHYYMFNSLLSPIMFVYMLICSFTICCSIIQLDSSETTI
SQRIWIIQYSIGQISQLFLYCWHSNEFAAKVKKKHFPLFPINLF
>BmorOR54
MGLNTIKEFFVNVKRRFQDVSIDSLLWIVNIVPSLAGFSIRSDRVSAPFWIVHWSLLVYVYAVGNAVYQWKFANEA
IDYITSFINVSLLILIGNNSWWFLANRRLLKSVLHKIEVNDELSRRSEQSRLKHKKLLKIIKRIVLVFYMSNYVNA
SFIYLPNRVDVLNNYAMTPCVGMEPLTVSPNRELCLTILCMQEFSIMTVVLNFQALLLCFIAHTAVMFQILADEIM
ALNNYENLEEHQAYVKEMLPIFVKRHSLTLSAVDNYKSLYSVPLGVNFGSNALTILLILYLPVLEWFKFIPIFVFC
FMLFFLYCFLCQKLVNASEAFETAIYCCGWENFALREMKMIYVMLHQAQKPVELLAADIVPVNMNTFATTLQAMYK
FVTVVKF
>BmorOR55
MCFLKIKQQIIDIQKHFKDYSLNGSLWIVNLLPRLMGFNLRADKVGVFFWTIYILLLVYVFGIGIFVYLWKHVDTM
SGLMKSYLNLSLILVIVNNSCWFLSKRSLLNKVLKKIHLIEDLSCESEHALAKYRRVFKIVTHLLLASYVLFYFTE
IYFMFLFRNYDLLEDYSLAPCVGLEPLSSSPNSEICLIIVLIHEFISTTVMMSFAALFLVLIAHTAVMFLVLAEDM
TKLTDLINLADHRKMIRESLRSLIHRHSLLLQIVYELRLLYSVPLGINFISNAMSILVLLCLPIHEWPSFLHIIGY
CFFAFFLYCFLGQNVINASEKFIDAIYCCGWEHFGVAEKKLVHVMLRQAQKPVEIIALGMISVNMNTYVEALQLIY
KFVTVLKI
>BmorOR56
MKLLEKLEDPDRPLLGPNVKALKFWGLLLPESRSKKYFYLFMHFAVTVFTATEYIDVWFVKSDLALLLNNLKITML
ATVSVLKVTTFLLWQNAWRDLIGYVSRADLEQRATSDSRKLALINGFTGYCRKITYYYWFLMYTTVAIVTVQPIFK
FFSSAAYRLDVQSGNGTYLQVVSSWIPWDKNTLPGYLLASIYQTYAAIYGGGWITSFDTNAIVIMVFFRAELELLR
IDCAALFDDEKSFGDMAFMRRLKECHRRHTELVKHSRLFDSCLSPIMLLYMFVCSVMLCVTAYQITIETNPMERFL
MTEYLVFGVAQLFMYCWHSNDVLYASQDLSRGPYESAWWSRDVKYRKNLYILVAQFNKVIVFSAGPFTKLTVATFI
RILKGAYSYYTLLSQSQMNKT
>BmorOR57
MPSLIKNRIFGLTLTLNTLSWAGLILRDDYTKTQRIIMKVYGGLVFLYLFVFTAYVQIADLVVIWGNIDFMTETSL
ILFMQLAVSAKVLTLMLKSKKIMEVTNEADAILISEKKVEGQRIIASIDKNTTLFLKYYGFFVAFTIICWFMGENT
STFFIRSKYPFNELKSPGREFAFVHQCIVVIFTGSFDFNVDIIIISLVAVCRCRLKLVALSLRNLCLDIPMNKRNL
ITSDEEKVITERLRNIISQHKRALDAAEAIKHYLSGALLVQLMVSIVVICTTAYQLAVKKSTTMQSLTMAGYLFGT
SLEVFLFCYQGEFLRESSEEIADAAYECPWYTLTRPLKKTLLIIMTRAQRPATLTAGGFVTLDITEYMAVSLISNT
>BmorOR60
MVRPCRYFAIHFILLRFLGLGWWHHPHENETRNYPGLYLYYSILTQLVWVVGLVGLETIDPFVGEKDMDRFMFSLS
FVITHDLTLIKLYIFYFRNVEIQDIVRTIEIDLYRYYQNDDKIRATIRISRIFTAAFLFFGWVTIGNANIYGIVQD
LRWKDIVKNLNETTSKPLRTLPQPIFIPWPYQEDKHYILTFILETMGLLWTGHIVMTIDTFIASVILHMSTQFAIL
REAIVTAYDRTMIALSEGALQSGVLCENSNGNEENNQIFLESFYSKEHIESVLESTLLSCIRQHQLLIGCVEKFSK
TYSYGFMTQLLSSMAGICVVMVQVSQGASSFKSVRLVTSLAFFFAMVIQLAIQCFTGNELTIQAERIADAVMESKW
EKMPVRLRRLLLVTMMRAQRPLHLTAAGFAYIDNTCFLSILKAAYSYYAVLSQKQG
>BmorOR63
MKLWIRNANFTISLSLTLLRCLGFWSPDGLAGNKRLLYNCYSFVFFMFLLGIYILIQVVDMIKIWGDLPLMTGTAF
LLFTNFAHATKVINIVIRKNRIQRVIQQANAVLMGVQSEEARRIVKSCDFETSIQLCLYFLLTFVTTVGWATSAEK
HQLPLRAWYPYDTSKSPAYELTYIHQVAALLIAAYINVAKDSLVSSLIAQCRCRLRLVGLALASLGQDLKIDYQSQ
LSPAQENILNLRLKTCVLEHQTVLAAVTELQACFSKPTFAQFTVSLIIICVTAFQLVSQTGNLVRLLSMGTYLMNM
IFQVFIYCYQGNKLSVESSEIAGSVYFSPWYLGSVKLRRALLIVMVRSRRVAKLTAGGFTTLSLASFMAIIKASYS
LFTLLQQVKQKK
>BmorOR64
MGVSNGRGTVKPFLYPLVDELDYNLIVGVHLPFEYKTPSRYPLAYITVVIAFIYVSYFVMVTDLIMQAHLLHLLCQ
FNVLADCFENMLNDCVKGFEGPLVSLHEYIHPLIDEFEYNLMVGLRLPFSFDTPLRYLFTYVIVLIAFNYTAHYVM
VTDLIMQSYLIPLICQYAVLADCFENILIDCSNDYGDHARRNDIVYSRSMELRAILSRPMLGQLASSGLLICFVGY
QATTSISVNIVKCLMSLFYLGYNMFTLFVVCRWCEEITNKSLNIGNAVYCSGWESGMTVVPTVRSTILLVILRANK
PIVFTAGGMYNLSLTSYTSLVKGSYSALTFLLRIQHE
>BmorOR65
MRLGFEVSISEYLYRNIFYIYTLFHILLHFYYILHMIKLDLEAIFDDIDESVALLPHRDTRRIEVQKILNGRMKRV

VTWHISVFKAVEAVSSIYGPPLAYQVMFTSIAICLIAIQITQKLENGILDIRFTMLGVAACLQMWIPCYLGTLLRN
KAFGVGEACWNSGWHQTPLGRMIRQDIIIVLLRAQQPVTIKFPGLQSIQLETFSSVIFNLYGYYYFLLLRWVDELT
AHLVLSGYWSP
>HarmOR3
MTLSVLDRFYLIDDGFFSFNLKYLFFVGLWPEKTLTRNQKILYKMYEHFISFLTTTFIVLAGIGTYQHKDDLVVVF
CNIDRCLVVYNFFFKTIIFFIKRNQLRDLIDEIEMSGDEVTEERKKLMANYVMFITGVTAAVIGAFSLLALFEGTM
SIEAWLPSDPMESLMNQILSLEILAFCVFPGLCRAFAMQGLVCSMIMYLCDQLIHLQKELRDLTYVKETEMVMRTK
FKNAIRKHIRLMGYSGRMENIFKEYFLVQNLAVTVELCLNAVMMTVVGVQQITLLITFLAYLMLALVNAYIYCYLG
NELIIQSQGIALAAYESTWTSWPVDLQKDLLIVILAAQRPLKLSAGGMALLCIQTFSQALYNGYSIFAVLNDAVN
>HarmOR6
MSFRKFLFENEAVDGIKSPSDYLYIKILRFTLDVIRSWPRKELGEPESASFTVFMKYFYLVLTIATVVGSILYVVV
HVSELSFLEAGLMYLIILMSFLDALTVMSLTFSAKYRVLAKDFLTKIHLFYYKDRSKAAMEIHKKVHLISHLFSLW
LLFQMLSGLSLFNLTPMYSNLAAGKYRRGGLGNTTFEHSLYYLYPFNTSTDVFGYIVACILHWIISYLCSTWFCMF
NLFISLMVFNLWGHFKILIITLEEFPRPKSIGTSESAYKYSQEELVEVAERLKDCINYHREIKNFTNRMSDVFGPM
LFVYYSFHQASGCLLLLECSQMTAQALMRYLPLTIILTQQLIQLSVVFELVGSESEKLKDAVYSVPWECMDTKNRK
MVRFFLMNVQEPIHVKAMGLANVGVTTMAAILKTSMSYFTFLRSM
>HarmOR7
MKIKMSKPLIFDQSIEKLGVLFRFSGMNIKNKIVTPLDTIKYRWLYTLNFLVVFSAIIGSVYYVILGIKQGKNFIE
VTSVAPCLTFSILSMIKSLYHLMYEEHIQELIDLLTEHEIRENNREKCIEKEEIIANETGFLNKVINVLYVLNCSM
IVVFDMTPIVMIAVKYYKTNEFEMLLPYLDVFSFIPYELKYWPFAYIHQIWSECVVLLDMAAADYLFFTCCTYIRV
QFKLLQYDFERMIPDRSISKGLFFEENELRNKFTELLKWHQDIIYSSTILEIIYSKSTLFNFLSSSLVICLTGFNV
TIVDDIVIIITFLTFLSMALMQVFFLCFFADLMMTASLEITNSVYNCKWYSANIKVGKQILFVQTRAQEPCKLTAA
GFADVNLNAFMRVLSSAWSYFALLRTVYGAK
>HarmOR8
MILFAMNFSFLPKQLDIFVEDLLFYFTDCAAISGILTIVFMREKVCELLEMLESDIFQPDDVEGLAIVEGAGKFIK
LYWNIFASVSFTSSAVHLSPIIVHFVIGTELKLPVCSYRFLSENFGQMFVVPLYLYQGSGNMFHMMYNVSIDTFFA
GLMVLTIAQLDVLDKKLRRVTDKDEHEDADGETFRQRHDKHREAVRKINQCIIHYEEINKFRRLVQDVFSISLFVQ
FGMGSCIICICLMRFTMPAPLSYFFFLATYMFLMVIQIMVPCWFGQRIIDKSNFLAFSAYNCEWTSETRQFKSNMR
FFVERANKPLSITGGKMFRLSLVTFTSIMNSAYSFFTLLQNVKSRK
>HarmOR9
MLDQFDRCLKSVNLYLKFLGLYLESKDTDKTFVERTRSHRLYFAHLFSLNLEVVAQVLWVLEAVITGKSFVEITRL
IPCLILCLISNFKTLSLLYYGRHNNEFIVTMRSLLLNQMQVEEKEHRFRKNLIDKHVLILTSISKKISYVIVLDLL
MFALAHAFIIIPHYFKTDEVKLEMPFIAYYPFNEFDLRVYPWVYFHQVYSAVIAMIMVYGPDCFFFTCCTFIHIQF
SLLNNDMERIVTEETPRYDKTKFKKLAVRHIELMRCVNLLEKIFSKSILFNALTSSVIICVTGFNVLVVDNIVMMA
SFTAFLIFGLMQIFLYCYYGDTIMRSSMEVSTSIYNSLWYNIPAADRKGFLIVIIRAQKPCALTADGFFKMNLSAF
ASILSKSWSYFALLKTMYHPE
>HarmOR10
MAVKNTSLFLGRPKKILSAHGVWPHPNNFVILRKLYMLFVMWTQYSFLLFEIIYIADVWGDIDAVSEASYLLFTQA
SLCYKSTAFMVNKQSLLELLEIMDCEIFEPKSAEHEKILAAQARKIKRLCLFFLTSATTTCTLWAMIPLFDAASKR
SFPFRIWMPVTPLKSPDYELGYLYQMVSIYISAFLFISVDSVAVSMIMFGCAQLEIIMDKIQKIKYVFESADSEEG
RRNIIKTNNEFLVECVKQHQTVERFIQLCEDTYHANIFFQLTGTVAIICNIGLRISIVEPNSVQFFSMLNYMVTML
SQLFLYCWCGHELTIRSENLREWLYQCPWYEQDTEFKRALFIAMERMKKPIIFKAGHYISLSRPTFVAILRCSYSY
FAVLNRVNTE
>HarmOR11
MHLAGNAVTGITGPMDYKYMKVLRFVLRIISGWPGKALGEKTLRIEGMGHAYYNTILSLVYLALGIAYLKKNFHRF
DFLELGQLYIVLLMNMLSTSRAFTLCLSQKYREVAKIFIQKIHLFYFKEKSDFAMKIHITVHKISFISAVYLSVLL
FIAACMFNLIPMYNNYSAGRFASFDNLENTTYEQAISCLYPWNFETNFNGYLAATLSGWYGTILCGSSVSMFDLFL
CLMIFNLWGHFKILIYNLEHFPRPASEVVDAEGEERSGRTVGSEMYSQSELEEVAVLLRDCIQYHMLIYNFTNNMS
DAFGMALFIYYSFHQITGCLLLLECSQMTAAALTRYLPLTIIMFGELVLLSIIFETIGTMSEKLKDAVYKVPWEYM
DTKNRRTVLIFLIKVQEPIHVKAGGLVDVGVTTMASILKTSFSYFAFLRTF
>HarmOR12
MEDEPLLIDKTVKNIEFLFRCTGINIKSGTKTRKDMIKSRTVYIINFLWLNIDLAGAVMWFFTGIANSKSFTELTY
VAPCITLSFLGNLKSLFLILREKHVDKLIQVLRDLEINEKARPKSEETDAIIKYEHNFVTTVISVLNVLYFVLLVA
FALSPVSLVALKYFTTNELELLLPFLIVYPFDPYDIRYWPWVYLRQIWSEVVVIIDICTADYLFYTFCTYIRMQFR
LLKHYIERVIPEDDGGGRLTNIEQVRAEFVLLIKWHQDLISSANMLETVYTRSTLFNFVSSSVLICLTGFNVMAIS
DVAFVATFLSFLFMSLLQIFFLCFFGDLLMTSSTEISEAVYNCRWYLADTSLGKDLLLVQTRAQTPCKLTASDFSE
VNLKAFMKILSTAWSYFALLQTLYGAPT
>HarmOR13
MKILSDGSDLEGVEKVEDIFYINLARKSMWILDSWPRTPNESVTYRYFVLALNVATLVGGAVYLRNNTGVLSSFEL
GHTYITVFMNCITCSRCIMILSREYNEVMLSFVNKIHLFHHRHKSEYAYKTHIFIHKISHFYTVYLLGLALNGLLL
FNMIPFYNCYSRGMFRDVIPANATYDHSVFYSVPFDYTTKFKGYIAMTSFNCFISYTCTSYFCVVDLTVSLVIFHL
WGHMRPLTYHLANFKKPASVLESNENTDAIKDHSYTQEELKEVFGKLREYIRHHNLILKFSSEMSNAFGPALLAYM
VFHQVSGCILLLECSQLDMKTLVRYGPLTVVILQQLIQISVIFELLGSSNDKLIDAVYLVPWEYMDTKNRKLVFVM
LRQSQRSIDLKMMSMLTVGVQTMTAILKTSFSYFVMLKTVAEEE
>HarmOR14a
MGGIRDFIFNLEAKEGITKPTDYPYMILCRHLLTVITCWPKEPKEGLDTRAKLKARIWVTFQKIFHLNGCFITTIG
MAMYIALHKNSMSFFELGHLYISLLMTVVIFSRVTTLCWNPEYQAVATDFLTKIHLFYYKDDSDFSMQTHKQVHKI
SHLFTLLLTGQMVAGMSLFNLTPMHNNFSTGKYKKGGLKNSTFEHSLYFSYPFNASSDVRGYILSNIFHWIISYLC

STWFCTLDLFLSIVVFHVWGHFKILIHDLNHFPRSLNTISFRLDQSNITLTTEMYSSRELVQVSERLNKSVEYHRR
IVSFTDKMSEVFGPMLFVYYGFHQTSGCLLLLECSQMTVEALVRYLPLTIILFQQLIQLSIIFELVGSVSDKLKDA
VYGLPWEDMDTKNRKTVAFFLMNVQEPVHVKALGLADVGVTSMTAILKTSMSYFTFLRSM
>HarmOR14b
MAGLRDFFFNYEANEAITTPKNYPYLIIMRISLSLIKCWPKKTTENLAAGAKMKAKVWGMVQNVLHLAFCVLTIVG
TATYVMIHKKNMTFFELGHLYITLMLSCVVFSRLATLTFNEEYQVVANEFLNKIHLFYYKDNSEFSMQTHKQIHRV
SHLFTLYVTGQMLGGLSLFNLTPMYNNYSAGKYSKGGLKNSTFDHSLYYSYPFDVSTGVRGYIFSNILHWFFSYIV
STWICTLDLFLSVIVFHIWGHFKILLHDIDNFPKPSKMVSFKLKNTNVTISNENYSTEELEQLADKLKKCIDYHRE
IISFTNKISEVFGPMLLAYYGFHQASGCLLLLECSQMTPEALARYLPLTLILFQQLIQLSIVFELVGTVSSKLNDA
VYGLPWEDMDVKNRKTVAFFLLNVQEPVHVKALGPADVGVTSMTKILKTSMSFFTFLRSM
>HarmOR15
MTFYELGHLYISLLMIVCTFSRITTLCLNDEYRVIAKDFVTKIHLFFYKDRSDYSMETHKKVHMISHIFTLYLSGQ
MMLGLFLFNVTPIYNNYSAGKYTSGGLKNSTYEHALYFSWPFNASTDFRGYVVSNILHWLLSFSCSSWFCVVDFFL
SLMVFHVWGHFKILLHDLDHFPRPANKISFILEDSYVTITDEIYSRNELNQVFDRLNKCIDYHRDIVSFTDKMSEV
FGPMLLAYYGFHQASGCLLLLECSQMTVAALVRYLPLTIILFQQLIQLSIIFELVGSVSDKLKDAVYGLPWEAMDT
KNRRIVAFFLMNVQEPVHVKALGLADVGVTSMTAILKTSMSYFAFLRSK
>HarmOR16
MGLRQFLFENEAVEGINSASDYLYIKVLRFMLLIVNSWPRKEIGEPESPKLSAFVKYFYLVVTVLASAGFILYLVK
HNSELTFLETGHMYIVLLMSFNDVSRVATLTMSTTYREVARDFLTKIHLFYYKDRSKQAMETHRAVHKIAHLFTLW
LVSQMLSGLSLFNLIPMYSNYAAGRFSGEVSKNSTFEHSMYYPYPFDTSTDIRGYSIACITHWIISYLCATWFCMF
DLFLSLMVFHLWGHFKILNYTLNDFPRPSSKVEAAKYSDEELVEVAARLKDCILYHREIILFTDRMSNVFGPMLFL
YYMFHQASGCLLLLECSQMTAQALIRYGPLTIILTQQLIQLSVIFELVGSESDKLKHAVYGVPWECMDVKNRRSVV
IFLANTQEPVHVKAMGVANVGVTSMAAILKTSMSYFTFLRSL
>HarmOR17
MFLRSECARSVAPHVRVLRCVGFLRGAALSSRGRAERLALRSYHALALAATSLYVLQQAVYAYQERGDMDKPSQVM
FLMLCHVTCVVKQIAFHVDADRIDRLIASLDEPLLNQCAGERGALLRGTARGAARLLRTYAGCAVATCVLWIVFPV
INRIQGISFEFPFWTGFSYDHNAVFTLVLLQSFYCTNLVAIGNTSMDAFMATILDQCKTQLRILRINFESLPERAR
ALHVESGENYDTILDKLFVDCLVHYNKITEMCTELHDVFAVPLLVQFGVGGWIPCMAAYKIVSLDVLSIEFASITL
FITCILIELFIFCYYGNEVTVESERVSQSLYSMEWRRARLTFRRSLVLVMERAKRPLRPAAGRVIPLSLDTFVKIL
KSSYSFYAVLRQTK
>HarmOR18
MEMKVDVLPEKKYKGFNETFKLCAFSLAFAFLYPNRTTALRRCITITLIVTFCGGQLFWFITYTFKCLYTLDIYNF
ARNMTLAVVLVLFFIKTYYVIYATSKFAPLLDKISDDLLEANNLEEEFQVLYDDHIKIAKVGEISWLLIPTIMSAL
FPIYAGALMTIESIQTDDYERRMVHDMELLFVEDIQSETPFFQCMFAYNCVQCVVLVPNYCGFDGSFCIATTHLRL
KLKLMTLKVNKAFKYSKSRQELRMRLYDSIKDHQDALDFYVQLQNVYGPWLFAVFLLTSFMISFNLYQIYLLQRID
PKYTSFGVVGVLHIYLPCRYASDLTRVSEEIPDDLYLAQWEAWADPSITKLLMFMITRAQKEMIVTGMGLVVFNME
MFKSILQTSYSFFTLITA
>HarmOR20
MDEELEFKPFHETYRLITFSLCIAMIYPNPRTEKWRLFSIPILIATVAPVAIMIFLDMYKCWKNGDIVNIIRHSTV
VGPFLGGFFKMILMYHKRVQAKQILDEFDRDHLMFNTVAETYKDIARASIRNCQIYSERLWACLVTTCVMTFPVMA
IVLNIYNFMFKSEPTKYMIHDLEKPFSKEPEERFESPYFELLFVYMFYAAILYVVNFTGYDGFFGLCVNHARLKME
LYCKALEEAMMADREEVYGRVIAVIREQCRMFRYVDLIQDTFNIWLGIIFIATMIQICTCLYHITEGYGFDIRYMI
FVYGAVVHIYLPCRYAAKLKAMSMETSNRFYCSGWERVDDERVRKMIVFMIARAQVPNEITAFNMMAFDMELFLSI
LQTSYSMFTLLRS
>HarmOR21
MIKRSKFFSWALILNCVLSLLMYTVEAVMRVIRAGATYTTVITAYPDVEDRSALSHVVRVIAYIIWCIYLTRIFAV
YSLVISLTIAMSYQFKNLTSYFCNLSKIFEDERMTQTEKEQEYERAFRVGIKIHSETLKCTEDIQAICRDVFSGQI
IFNILMLIVLMHQMVNSARNLTNAVTLVMAALTILLSTGFFMWNAGDITVEAQLLPTAMFSSGWENCGRDSSVRVR
KLIVIAMMQAQEPVVLTGLGIIALSYQSYVSVSTFTK
>HarmOR21.2
MTKFLDELNTIFFLVGLTDLWISEVKFSKRFIQIYKKINYVMDFLCLFFVVFLLGSYFTQKDLTEKLANDRLMFSI
ILPGNLVFYYISVYYKEEIRNLLYHHRVLKEQHNDTRLEREMIRNIRVFSITLNSIAFVVDTSYGFGALYEVVTKG
ENFNTIVPVWPDVHDNSSLAGAMRVFFYFCWLNPIATRVLTTFSLLLTEMVAVCYQFRNLQSYFYSLDDIFSDDTL
SQKEKEIKYEEGFKIGIRMHIMTLWCKKLHQHVNKEILAIEMVLFFAMLMSELTTLLGGERNASQLCMMFLISVST
CISLGFFMWNGGDITIEASKISEAMYSSGWQHCRGHSSVRMRKLVTFAIKQAQDPVVYKTLGVVDLSHTSYVTLVK
MPYSAVSVLY
>HarmOR22
MMAIHVIMVIFCFSCTLSLWTQKNLSESQQSDRLAYGASAPIITIFYHFVILCYKDDVRKVLYKLVVVLKVDHNDK
QAEREMMEQSRLHNGLFFSSCVCNMVFVGLYNCYLAVTTDATFITCISAWPDIEERSLPAGLTRVVVYFVWFAHVV
RNMGVFLIIHTVLLCLTQQYKNLQSYFEDLNKIFDETKLSQEEKELKYEIKFKRGIEQHALTLWCVDETQRVFKIT
FSSHVLLWCGLLISILPDVMNNDDHTLKMLVSNAPRVAAALVGLGYFMWPAGDMSVEASNLPQAMYGSGWQCCYHR
SSRVRKLVVLAMMQAQRQIEMKAFGHLTFSYE
>HarmOR24
MDKLARVMFLLLCHITSIAKQLVFHLKAERIDEMLAGLEDPLYNQPEEAHRRLLGATAASASRFVRAYSGCAVVTC
TLWITFPVMYRLQGLPVEFPFWITVDYNRPTMFILVLAYSYYVTTLVGIANTTMDAFMATVLNQCKTQLRLLRMNF
ECLPERAAALSRQLGGSYDAALFALFRECLVHYEKITETAKMLQNIFGTAILIQFGIGGWILCMAAYKIVSLNMLS
VEFASMALFISCILTELFLYCYYGNEVTDESERVSQSLYSMEWRRARLTFRRSLVLVMERAKRPLRPAAGRVIPLS
LDTFVKIIKSSYTFYAVLRQTK

>HarmOR25
MPSDQSRMFDPPLTVLKIFGVWEGRTPSKYYKTFSFLFLFVSWFFYNFLLTLSLVYTPRSVELFLRELMFYFTEIS
ITSKFLTVLLLRNKILEVFSVIDSDEFVGDYENKDGILYRTNKGYSLCWKVYNVLANIDYTCVIIMPVVIDLIQGT
KSVLPICNYYFLSEDFRDSHFVILYLYQSIGMYGHMMYNLNMDSLAWGLLAVGIAQIKVLNKNFTDLKLSAEESKL
PLEIQDNIQKTRLFKLLRHYEAILNYCDAIQNLLSVTFFFQFSFGALTTCVIMCSLLMPGTMVYRIFLVIYLFAMA
GQIAVPGFFGTLLTHESQELVTAAYNCEWIERSQSFKRTLILFRERAGTPIIISGMKMFPLSLVTFVAIMKTTYSF
FTLIRNA
>HarmOR26
MDCTIVAFYAQAKIQIQMLRHNLEQLVEFDDSAKINTQFNKTGLYSTSYKDEQQERVAIQERLKKCVQHYYQILRF
AKEVESIFGEAMVVQFFVMAWVICMTMYKIVGLSIYSAEFVSMAVYLGCMLAQLFIYCYFGTQLKVESELVNQSIY
CCDWMKLSPRFRRQLLVMMQCCGRPIAPRTAYVIPMSLDTYIAVLRSSYTLFTFLNR
>HarmOR27
MGKDDPDRLFECFSVLSFCAMGMLKLLSLRKNHRKWRKLLTQITILENTQLSNRSISCVEYQSDSEDSDNFSEHIS
IYTKKFRGTSIVLTRIYSFTAFLFILSPFAERIICEIRGVECVGYPHVFPGWTPLDDFSIFGYLVTVLCEVFSAVY
CVCVHMAFDLTVIGIMIFVCGQFSLLRDYSSRIGGKGRQCNLSMRRDERARFRIIRCHDINLLLVNSITELDMLLK
NIIGVYFFVATLTLCSVAVRLKSEDMGVMQLVSLIQYMCGTLTQLFLFCRYGDAVLHESTMGMGEGPFAAASWCLS
PRVRRDLSMLSAGMMSQRHLRAGPFSFIDLPSFIQVVRAAYSYYAVLGKKE
>HarmOR29
MGYQQIDCFDIHLKILRILGVWPHDNPSIYYIYFSRIFVFTFTVLYVVIYTMNFYFLPQQLEVFADELIFYFTNVG
ALSKALAFIFLRDKVKKMLFMLESEIFQSDDPEEIKLIKEGKEKSNFYWKITAGLSVSANTVNVCLPLLVHIIFSV
ELEFPVCRYSFIPEKYEAMFAYPAYFYQSIGITTHMLYNVNIDTFLLGVMFLAMTQLDILDRKLRKVTDVCINPDA
ARGSVDKFIDDQNAVLEIIKCIKHYDAICEYCKLIQDAFSEILFVLFSSGSCKICMCLFRFTMPATTGYFVFLYTY
VTVMTLQVMVPCWFGSRLMDKSSQITIAAYDCDWTPRCRRFKSNLRLLVERANRPITIIGGKMFLLSLGTFTAIMN
SSYSFFTLMRHMQSR
>HarmOR30
MVSSQITCGLWAMKPLFDDADRKFPFDMWMPVSPEKAVQYYIGYAFQLGTICISAYMYFGVDSVVFSSVIFGCAQI
DIIKEKLMSITTVDRKQGTKEALAQNYNKLVDCIKHHQAIVTFTELVENAYHPYLLFQLVGSVGIICMSALRILVV
DWRSMQFFSILTYVSVMISQLFVCCWCGHELTATSEDLHTVLSNASGTSRT
>HarmOR31
MNSILQNLEDPNRPFLGPNYWIIKNMGLLLPKNFLAKILYIILHEIVAFFVITQYMELYVIRTDLDLVLTNMKISM
LSVVCIVKVHSFIFWQKHWHDVLDYVTAADKFERQSDDPIKSRIVETYTRYCRRLTYFYWVLVFTTFLTTTGTPLM
RYLSSSTFRQNMRNGTEPFPHIFSSWMPIDKYHSPGCWITVLWHTLLCAYGAAIMAAYDTCIVVIMVFFGGKLDLL
RERCKQMFGPSTISDRQCEEVVRQLHGIHVMMLKYSRLFNSLLSPVMFFYMVMCSLMLCASAYQLTSAQNAAQKLL
MAEYLIFGIAQLFVFCWHSNDVLIKNENMTSGPFESNWFLANYRQRKDVLTLSGQLCIKNIFTAGPFANLTLPTFI
NILKGAYSYYTLLRK
>HarmOR32
MNKDFQTSKDLPPKERDSIKKYINQGLWVCKQWLFLTISGCAIFLFKNLGLMLYYYCMNEFRLVPFYEVVLYPPIM
EENRDNIFVYLLMYAIMLLFSAYSALMYAAFVPLGPIFILHACGQLVLVKLRIDDLFVECDDEVIRKKLKGIILHL
QYVHSFVDRIQQVFKIGYELTLKFTALILPITIYAVLEGFYRGEVNVEFVTFIVGGVMISGSPCYYSDLLMEKGED
VRMSLYTCGWEQHYDRRTRTTLQLMLQNALKPIAIQTVFTVMCLDALTDLFQQSYAIFNLMNCMWN
>HarmOR33
MWRYIRKFGLEYCDLPTMLWNVSVLLKVLTVNIYGKNRKAIPLIFYIIVTVGLLTYFYVYVVSMIWFVFSRCPVTG
DVLAALIVFSLGVASEISTVKFLYMRIHIKDVRKMVADCLDSYSKVVPGTRFSNNLLRTLREAKRRAMLFWMVIIG
NGLMYVVKPLLLPGRHFMDDVVLLYGLEPMFETPNYQISFVLMGSSCVLICYLCANISAFLIIITGYVQAQMLALS
EELTHLWEDAEENYRGNELEDITDDGDQNDRNKDAILNDYVTVHLKDIAKSHAENINLLGQIEGTFRGAIAIEFCL
LVVALIAELLGGLQNTYMEVPFALMQVGMDCLIGQRVMDAGAVFEDAVYDCKWERFNKKNMKTAMVLLLNAQRPMT
ISA
>HarmOR34
MSAKFSIMQIFKFLEDPAYPSVGPHLKLLGFTGLWHPNRHTLVGRFKHILFYITISFFFSQYIKCFINFNASSLKL
ILQYAPFHMGIVKSCFFQKDYKTWQQVIDYMSSVELAQLSKSNKEQYKIIYDYIKRNRKVSYFFWALAFFSNFSIF
TEPYQKNQINVNGTSIYLNIFDGYTPFQKEPPGYYISMLIQTVLGHIVSAYVVGWDTLVVSIMIFFAGQLKITCLR
CKMMIDVTNPMKSHLKIAECHRFHTTLVEYTRIFNALISP
>HarmOR35
MCLYLPFDLVIVIMTSNVSALLRLLQVDLKNAIQLRDEQHKTKSHLNVSDTQSYEELKRIVDIHQRLLRIADQLSS
IFGLVIFIHVACAALEICFFGFLTMVYGGLAETIANMLTVLNAVFTIFLLSLSGQFLCDTSSEVADAAYESYWYES
DHKVKKLTLSIIIRAQRPSYLSALGFSQLTLKSFSKIMSSAWTYFSLLIQMYEET
>HarmOR38
MIILSENIKQKLAFLSPYLPYGVIESWEDLNPRLYHAVHIYWLKFYGMWFNNYSPNNIKFWLHMVYTLTVLWLACF
FPGIGEVVYLLKQRENIGDIADGLYLFLSEMYTYVKIAVFWMNRDKVISLLEYLHCKEFKPKEPEHRDIITKSIKS
ARFVMTYYSTMCVGAVSVGIIMPLTENFDILPTNVEYPFFNVYRSPAYEAVYIHHIYYKPATCIIDGVMDTILAAF
VASAIGQIEILAFNLRNFNLVAERQRRRDLAQNKYIEEYPAQHYVRSVLKECIRHHNCIIRYVSMIESAFSLASAL
QFMLSVMVLCLIGIQFLSIENPSAHPMQIAWMGIYLTCMLIEVFILCWFGDELIWKSMDLAKAAFEGPWMNSDRQT
NMFIIILLERCKRPLRLSAGKIFTLSLDTYTVLINWSYKA
FAVMRNMKK
>HarmOR39
MHRKRKEFNDLLEVAQKNDDLIIETGRFLHVHEKMLRSIKIIILFCYVFHFINEVVVYIPFRILRMEDFSIASCVG
FGPLNVSPNREVCMGLMTAHILISIMVICCYDISLLFLFSHTTAVFQILFEEMMSINDITQTCQNSDEDYAVIVAR
LKNVIVRHVLALQTVGKVEDIFSVSIGICFGLDAISLCLILCVATRSLYAFCSDFTIAFSIFFLYCCQGQRLTTAS

EKFEMAVYCCGWENLRVKERKQVLLMLKRAQKPVIVYAAKVIPIRIYTFASTMQAIYKFVTIFKV
>HarmOR40
MAMRNLSLTMLSTVCVFKACNLMLWQNSWKELIDYVSELERSQLSKNDPVVNKIISDYVKYARRVTYLYWALVTAT
VVTVILAPLFIYLSSPNYQESIKNGSAPYPEIMSSWTPFDRSRGLGFCGATLYQMLACFYGGTVVANFDSTAVVIM
TFFTGQLKVLSVNCERLFGDGNELVDYDEAVKRITECHLHHYYMVKFSSVLNSLLSPVLFLYVIICSLMICASAVQ
LTTEGTGNMQRIWIAEYL
>HarmOR42
MTNSRPRHYFGFHYRILRFLGLGWWHHPEEGKTTNFPGWYLYYSIVTQVVWVAGFVGLETIDPFVGEKEMDRFMFS
LSFVITHNLTLIKLYIFFFKNVDIQEIVRTLEIELYDYYQNIEKNRKTVKISKIITGSFIFFGWLTIGNGNVYGTI
QDLHWKSLVATLNDTSQIPVRTLPQPIYIPWNYQKDKSYIPTFVLETVGLLWTGHIVMTIDTFIASVILHMGAQFE
ILNEAITTAYDRTMTSLREGIRPEDSGHQGQQSSILSVEDSNERIVHAFIPKEEIDAALQTTFRNCFRQHQVLINC
VEKFSRTYSYGFMTQLLSSMAAICVVMVQVSQDASSFKSVRLITSVAFFFAMITQLGMQCFTGNELTLQAERISDA
VMQCKWERIPTRQRRLLLMMMMRAQRPLRLTAAGFTNMDNACFLAIMKAAYSYYAVLSQRQE
>HarmOR43
MVKNENRSLQYCLTVLKVAGFLHPLGDGRIPRLTRRLYCFGVFMFLVGCIIMAQTGAMFEIWGDLALMTSASFLLF
TNLAFATKIINVVVRCREIQEIIDEGDADLLAEDRYLGIEVIKSCNVETSLSMGLYTLLSGVTVFGWAASAEKNQL
PLRAWYPYDTSKSPAYELTYIDQSSAVTLAALVNVCLDTLVTSLIAVCRCRLRLVALSLRTLCDGIPLPDKQLISP
TEERIVLTRLSQCIIKHEAALKAARQIQRCFSLPILAQFAVSVVIICVTAYQLAMELNNRNWFRCIPMVAYLLCMA
LEVFLYCYQGNELLEESSEIAGAAYECPWYHCSVRMRRTLLIVMVRTRRALRLTAGGITTLSLACFTSIIKGSYTF
FTVLQQAEDRNPK
>HarmOR44
MGYMIYSSSARMLSALVVICEIWHALGNNMSLDELISSVNVIFIHLITLWKLMIMVSNKKVFKKLARALESPSFDI
STENRQAIVNHWVLTHKKYLKVLLCLAYLTLAVWVLHPLVDDMDFNLMVDVKLPFAYDSPLRYVISYLFVGTMFSY
ASSMVIMSEVIMQAHLIPLVCQFNVLANCFENVFEECASEFPDINKHELVKHNMFVEKYRKRLGDLVKQHREILDQ
TTDLKTILSAPMLGQLACSGLLICFVGYQATATIAENLGKFVMSLFYLGYNMFTLYIICRWCEEITIQSQRIGQSA
YFSGWESGVSHVPGARATIILVIARSNKPLVFLAGGMYTLSLTSYTSLVKASYSALNILLTTKHE
>HarmOR45
MRLQILEDFLLKKTFDFDRPDINLYNFHPQLRILLAVKGVFFTNRRSLLRFIWPCICIQLSIVAMTLEEIFIWRGV
TVKDYSFATECFCYWVILGCIPMVYVSIVVNTNKIYDIVVTMNEDFIYVCSLGDRYRKPFLEGQLLIWQLCYAWFI
FVCFVGGLYVIIPLVGLLYQSLFATIDENTVRPLQFPMWLPNDDPYRTPNYEIFLVIESTLIFCFVQTFCVYIYTL
LHILLHYYTIMNMIIIDFSVIFEGLDESVALLPRHDPRRRETQLILNARIAKIVRWHLSVFKAVSTVSSVYGPPLV
YQVSFSSLAICLIAYQIAEKLDNGKVDILFCLLGIAACLQLWIPCHLGTMIRNKAFEVGDAGWTCGWHETPLGLMI
RNDILIIIILRAQKPVTIKFTGLPSVQLETFSSTMSSAYSYFNMLRQYSK
>HarmOR46
MSSLGTIADSLPLLVSLIIVAYYAMYRQDLYDLMEYMERNFKYHSAGGLTNMTMEESCKTAQRFARIYTACTMFSV
TMYATMPVIIHLWTKEPIQSWMYMDITRSPFYEFVFLVSCLAQMFVGLAMGQFGVFFASNSILICGQLDLLCCSLR
NARYTALLQHGVKHAALRVSHATIQDDEKHNYIYNVSEMKESVYHYDKKVSNLYAEAKTQFDIYSSEFDDATVNAL
RDCASLCQVINRYKEMFENFVSPLLALRVVQVTLYLCTLLYAATLKFDMITVE
>HarmOR50
METTTYTRSKTTEFFYKMNFAIYIFGLPNFWIEDLKLSKRFVKIYDKISLFNDLLVYLLLVMEFGAFFTQHNLTDK
QKFNLMVFAISHPLLCSFCVMVSKLKKKVRLVMYSQAVALKRDYNDPEVEKQMIARSLTYVLAFMSSCTITMIMFA
IEAIWDVIRHGATFTTLITAYPDVQDRSILADVVRVLAFVTWWIFLTKMVAVYMLVIPLTISLRYQFKNLQSYFLS
LAELFERSDLSQKEKEEKYEAGLKLGIKLHSETLSCAEDTQDVCRGVFSGQIIFNILLLIVLMAQMVTSERTFVNM
FGTVATSCTVITSTGFFMWNAGDVTVEASYLPTAIYFSGWQHCQRDSSMRVRRLVVTCMSHAQQPVIFKGLGYIEL
SYQSFITIVKSSYSVFSVLY
>HarmOR52
MRTLREIGQEIRKFGLEYCDLPTMFENVAILLRLLTLNIDIKYKGGITFYSYIIITIVSGACYYYVFFFSMTWYVFW
RSRELGEDIGAMIILSLGITSEIGPLKLFYMSYNKDKTQKIANDFLECDANTIKSTRFYANLLKHCRTVKKRAMLY
WIVVAGNGVIYLLKPITMKGRNLPENYFLIFGLEPIFETPNYQIAYCMMVSALFFVCYVPACVTAFLIVVTGYAES
QMLALSEEMIQLWPDAIKRAEERTQLDPSKVLDVYNLEVKTIMNQFVEKRLKEIIKRHALVINLLNQVEIVFRQAI
AMGFVLLIVGLLAELLGKLENTFLQMPFAFMQVSIDCFAGQRVMDASLVFEKAVYDCRWENFDKANMKLVLVMLQS
SQKTLALSAGGISTLSFTALMSIYRGLYSSYTALRSTVK
>HarmOR53
MSEKEFDKTLKLTNYALIMSGIKTSENDMNKALEYFINHYLFYCNAIALHTVIFGEVYWIVDGIRTNHPFVELSLV
SPCATISILSTIKCGFIFSNKGILMRVVHKLKEIHTSFDDNELSKESSARTKIVTDSLKLLQFVQISFATIYIFVF
FSFCFIPVILAEYNYYRTGEFVVTYPFFVKYPFDFDVHHCPVWQLIYFHQVWATAIVIMSMFGCDSLFYGLCVYIK
THFQLLGLRFENIVGATKSETQRNLAKAVVRHQELIDLVNQMEMLYSKSSLVNIITSSILICLSAFNITVVDKLNV
ILAFVTFLVMSLSQISLVCYFADLLMAASMEISGSVYRSPWYEADNHSKKILLLVIMRSQKACKLTAWKFADLNLG
AFTTILSRSWSYFALLKTVYK
>HarmOR54
MLTASSRLKTVFEMSHHGLSLIPEGTASKKIMLATLQQARYFSWLVVANLAVTHVTYLLMPFLFTVLGNDRYLPTT
PGETYGLSPKYETPFFEITFVLTSVATAFSAINQTGYIVLFVTLICHELGHFYAITEALHEIHTILTKEERSRNNS
EQNEQKSVDKLLIFCVKHHQFLMNFHGKIRDMYKVIFGAHFLSMTVVLVTTLQTMNVWDYRNTILTGMSGIMPLFL
YCFGGEKLISAGLQMSSAAYSCGWEMMEAKQAKVVLLMLCLVQRPLYLTAADIFIMNRETFGDVAQVVYKIYAVFN
>HarmOR60
MGVLVRNATMSVSISLTALQFVGFWAPEYLGKTQKQLYTCLSVFSFMFLLGTYLIIQVVDLFLIWGDIAMMTSTAF
LLFTNMAQAAKIVNIVYRKERIQRIVNDCDAVLSRAQSLEEKEIVKSCNREMIVLQILYFSLTLITSLGWATSAEP
HQLPLRAWYPYDTTKSPAYELTYVHQVGALLIAAYLNVAKDTLVAALIAQCRCRLRLLGYALRTLDKGMGNEAYTF

TSEQEKTLNLRLGSCVMQHQKALDVGKELQECFSEPTFAQLTVSLIIICATAFQLSMGHSDNMVRLLSMGTYLLNM
TFQVFIYCYQGNQLSEESSEIAGAAYECPWYKCSVRVRRGLLIVMVRTRRALRLTAGGFTTLSLACFTSIIKGSYT
FFTVLQQAEDRNPK
>HarmORco
MMTKVKAQGLVSDLMPNIKLMQMAGHFLFNYHSENAGMSNLLRKIYASTHAILIFIHYACMGINMAKYSDEVNELT
ANTITVLFFAHTIIKLAFFALNSKSFYRTLAVWNQSNSHPLFTESDARYHQIALTKMRRLLYFICGMTVLSVISWV
TLTFFGESVRMVTNKETNETLTEVVPRLPLKAWYPFNAMSGTMYIVAFAFQVYWLLFSMAIANLMDVMFCSWLIFA
CEQLQHLKAIMKPLMELSASLDTYRPNTAELFRASSTEKSEKIPDTVDMDIRGIYSTQQDFGMTLRGAGGRLQNFG
QQNPNPNGLTPKQEMLARSAIKYWVERHKHVVRLVASIGDTYGTALLFHMLVSTITLTLLAYQATKINGINVYAFS
TIGYLSYTLGQVFHFCIFGNRLIEESSSVMEAAYSCQWYDGSEEAKTFVQIVCQQCQKAMSISGAKFFTVSLDLFA
SVLGAVVTYFMVLIQLK
>SfruORco
MMTKVKAQGLVSDLMPNIKLMQAAGHFLFNYHSENGGMTGLLRKIYASTHAILITIHFACMGINMAQYSDEVNELT
ANTITVLFFTHTIIKLGFFALNSKSFYRTLAVWNQSNSHPLFTESDARYHQIALTKMRRLLYFICGMTVLSVVSWV
TLTFFGESVRLITSKETNETLTEIAPRLPLKAWYPFNAMSGTMYIIAFAFQVYWLLFSMAIANLMDVMFCSWLIFA
CEQLQHLKAIMKPLMELSASLDTYRPNTAELFRASSTEKSEKIPDTVDMDIRGIYSTQQDFGMTLRGAGGRLQTFG
QQNNNPNGLTPKQEMLARSAIKYWVERHKHVVRLVASIGDTYGTALLFHMLVSTITLTLLAYQATKINGINVYAFS
TIGYLSYTLGQVFHFCIFGNRLIEESSSVMEAAYSCQWYDGSEEAKTFVQIVCQQCQKAMSISGAKFFTVSLDLFA
SVLGAVVTYFMVLVQLK
>SfruOR53
MFLKKVIKYTKRLEDPKNPLLGPTLKGLYLFGLWQTGGKFRTVVYNLIHLTTFFFVTSQFVDLYFVRHDINKVLNN
MSLTVLSVICLAKCFSYVFWQSEWRKLAQSISEEELKEIKNGDPIILKHMEGYTKYTRIITYMFWTMVLITNFLLI
LTPLLKYVSSHSYREEIRMGTEPLPQILCSWFPFDNERMPGYLISVIVHIIMGSQGSGVLAVYDMNAVAIMSYLKG
QMIILREKCNSLFDDVTSTQDVLDRIKECHRHHNVLLKHSSVFNSLLSPTMFVYVLMCSITICGSVVQFSSKEATA
SQKLWVFQYTSGLISQLFLYCWHSNEVTLHSKLVDRGIYSSDWWKSNVRVRKQLLLLAGKLNHPLILDAGPYTTLS
IPTFIEIMKGSYSFFTLFSQMQEN
>SfruOR30
MVSSEDLFLNRAKFVMKYLGVWVLPENASCFLKAYRGFMMTLQYLFLIFQMIYIVQVWGDLDAVSQASYLLFTQAC
LCLKVTVFQINMPILKELLRLMDAEVFKPENDVHEKLLELQAARIKRLLLAFMVSSQITCGMWALKPLFDDADRKF
PFDMWMPVSPEDAMQYYIGYAFQLGTICISAYMYFGVDSVTFSSVIFGCAQIDIIKEKLMSITSIKERRGTKEVDE
ALADNYNKLVDCIKHHQAIVTFTELVENAYHSYLLFQLIGSVGIICMSALRILVVDWRSMQFFSILTYLSVMISQL
FVCCWCGHELTIRSENLREWLYQCPWYEQDTKFRRSLFIAMERMKKPIIFKAGHYISLSRPTFIAILRCSYSYFAV
LNRVNTE
>SfruOR60
MGIFVKNATLSVSISLTALQTVGFWAPEGLTETQRCLYRWFGVFSFMFMLGSYIFIQVVDLFMIWGDLPLMTGAAF
LLFTNLAQATKIVNIMVRKQRIQNIVNDSDKVLTEVQTFEEKEIVKSCNREMIVLQVVYFSLTLITGLGWATSAEK
HQLPLRAWYPYDTSKSPAYELTYVHQIGALLIAAFLNVGKDTLVAALTAQCRCRLRLLGLSLRNLDKGLDTEDKFM
LTPDQEKTVSSRLRLCVVQHQETLQAAKELQECFSEPTFAQLTVSLIIICVTAFQLSMTQPDNMVRLLSMGTYLLN
MTFQVFIYCYQGNQLTEESFEIAGAAYECLWYKFSLRLRRALLIVMVRTRRALRLTAGGFTTLSLTSFMAIIKASY
SLFTLLQQVNEE
>SfruOR32
MTNHKELNFESTFKITTMALHISGAHPGVPKDLKWVLKFIILHGIFTFTFSIVIYSIINHDLKEKNFIQICKNGVM
FVVFCVISFQYFVLVIHQKNLVELIKNVNADYEELQNLSEKEKRLMYKYVDQGIKVCRQWFILTFAGCMIFIVKSI
GLMLYYHLINDFQYVPLYDIKYPALIEDRKNDNLFVFLGTYLLLLSFACYSSLNYTSYVPLGPIFMLHASGQLELV
RNRIEDLFLECDAEAIRVKLKGIIMKLQYIYSAVDDMKKVFKFGYEITLKGTAVILPITFYAVLETAKNGEISLEF
ISFIVGGIMISGAPCYYSDLLMEKGEALRMSLYTCGWEQHYDRRTRTTLQLMLQHALRPIAIQTIFRTLCLDALTD
LYQQSYAIFNLMNATWN
>SfruOR35
MTSLYRRYFFKKKSKQRSNERIYDKSDYDTSYAPTKKVLGWVAIRMTHNISEKTTMLWDMFYWFEMANLFLVGPSE
LVSMLTTAYEAKTFRDSIKVFRTMPCFGCVVLSMFKSIKMVVHRPVYENLANELREMWPEGEVSEEEHHIISSALK
QLNFIVKGYYWCNNALLISFLSPPYFITIARYFGYDSPMGLHFLYWLPFDPYQPVYYEITLVLQTWHALVVIWFNV
AWDMLFCLFLCHITTQFDLLARRVRRLFYVQVDKQLVSSYPMASVSKEFLETEGDRVNSYGAQYWEARYQKEITEI
VLRHHSLIRLTNDVENMFSLALLINFMNSSIIICFCGFCCVLIEKWNEVAYKSFLVTALSQTWLLCWYGQKLIDSS
QRLSDALYGCGWYNSSKRARSAVLIMLHRAQKGIYVTTHGFSVISLASYSTIIKTAWSYFTLLLNFFKEKSVN
>SfruOR16
MDLKKFLFENESVVGINTPSQYIYIKIVRFMLVIVGSWPRREMGEPEPRHQTIMINSFFFCVVNAALYGSISYVCM
HTSELSFLEVGHMYIVILMSAIDMSRVFTLTYSQRYRDLAKEFLTKIHLFFFKDRSPYAMLTHKKVHLICHLFSLC
LLSQMLTGLSLFNLIPMYTNYASGRYSTGGTQNSTFEHSLYFPYPFNTSSDMNGYVVACILHWLLSYFCSTWFCMF
DLFLSIMVFHLWGHFKILINSLNNFPKPSAETRCELEKGFIINADKYSKEELVEVSQKLKECIDYHREIVQFTNAM
SDVFGPMLFCYYIFHQTSGCLLLLECSQMTAQALIRYVPLTIILTQQLIQLSVIFELVGSESEKLKDAVYGVPWEC
MDANNRKVVAFFLMNVQEPVHIKALGVANVGVTSMAAILKTSMSYFTFLRSM
>SfruOR45
MRLQIIKDFLLKQYFDFDRPDINLYNFHPQLRIFLAVKGVFFTNRGSRLRLIWPSICIQLSIIAMTFEEMFIYRGV
TIKDYSFATECFCYWVLLGCIPVVYVSILVHTNKIYDIVVKMNEEFIYVCSLGPVYRKPFLEGQLLIWQLCYAWFG
FVSFVGGLYVVFPLAGLIYQSLFATLDENTTRPLQFPMWLPHDDPYRTPNYELFLLIQSTLCFCFVQTFCVYVYTL
FHILLHYYIIMDMIIIDFSVIFADLEESVALLPRYDTRRMETQRILNARIEKIVKWHLSVFRAVKTVSSIYGPPLV
YQVSFSSIAICLIAYQIAEKLDQGSLDILFSLLSICACLQLWIPCHLGTMIRNKAFEVGDAGWKCGWHETPLGLMI
RTDIIIIILRAQQPVTIKYTGLPEIQLETFSSCMSSSYSYFNMLRQYSK

>SfruOR50
METTTTSYTHSKTTNFFYKINFIVYIFGLPNFWIEDLKLSKRFVKFYDKFSMFNNTLIFLLIIFELCSYFTQSDDQ
LTEQQQSNRLIYAISHPMLFMFRVMMTSIKERVRLVMYSLNVGLKRVHNDLEVEKQMIACTFMYLSALLLSCLMSM
LMYAAQGFGEVFRRGKTFTTIITAYPSVEDDSDMANVVRAICFIIWWIFLTRIYAVYMLVISLTTCLSYQYKNLQS
YFVSLNDIFERSDLSQTEKEEQYEAGFIVGIKLHADTLRCTQLTQSVCRGVFSGQIIFNILLLVVLMAQMANSERT
LVNLCSAGFTACAVLISTGFYMWNAGDVTVEASHLGTAVYFSGWYHCQGPSSVRIRKLVVFTMSQAQRPVVLKGLG
YIDLSYQSYIRIVKSSYSVFSVLF
>SfruOR39
MANFRDRFHNLRDRLKQNSCVNLVWLINFLPKLAGFPLLSQKFKFLFWIVHISFLFYVYLLGTAVYQLYFAEDFVD
SINSFFNISVFILIGNDSWWLISKRNDLNDLLKMVRKNDDLIIESGRFLDVHQKLLRSIKIIVIMCYVFHFVNDVM
IFIPSRTIGMDDFSTVSCVGMEPLSSSPNRQACMAVLALQELTAIVAVCSYDVALLFLFSHTTAVFRILYQDMTDF
ANISKSHETYHVIEDRLKNIVFRHVLALHTVRKLEDIYSVAIGIGFGLDAISMCLFFVLPLDVCLNFAPLIYHSLF
IFFLYCFQGQRLTTASEKFEMAVYCCGWENLRVKERRQVLLMLKQAQKPVLVYAARVIPIRIHTFASTMQSIYKFV
TIFKV
>SfruOR13
MDDIKLSTVKIFSDGSDLEGIEKVENILYLRILKKIMWVIDGWPKEPNKRQIFRYYICILDMLSLVPGSLYLVIYT
GKIPSVELGHSYITVFMNAIAALRTVLVLTKEYNAIVLYFLKEVHLFNFRRKSDYAYETHILVHKISHFFTMYVFM
LMCCGILLFNLTPIYNSYAAGMFRDERPANASFDYAVFFALPFDTATNFKGYVVVSLYNWYISITCSTYFCIIDLT
IFIMVFHLWGHMRVLSYNLENFPKPASVLAAADDGSAYTLCENKYNEEEQVEVFIRLRDCIQIHSLVINFSSMMAD
SFGWTLLVYLFFHQVSGCLLLLECSQLDTAALMRYGPLTIIIFQQLIQLSIIFELLGSSNDRLVDSVYSVPWEYMN
TANRKNVFVMLRQTHRSMNLKACSMVTVGVQTMITILKTSFSYFVMLRTVADEEE
>SfruOR3
MALTVLERFYLIDDGFFSFNIKYLFFVGLWPEKTLTRNQKILYKIYEIFIHTLTIIFLIMAGIGTYQHNDDLLIVL
SNMDKSLVVYNFFLKTIIFLIKRDQLKDLIDEIEASGDEVTKERKKLMANYVMVITGITAAVVSAFSILALLEGAM
SIEAWMPFDPMESSMNLVLSLQIIAVCVFPALCRAFAMQGLVCSMIRYLCDQLIHLQKELRSLDYVKDTEMITRMK
FKMIIRKHIRLMSYSMKMESIFKEYFLVQNLAVTVELCLNAVMMTTVGVQQITLLFSFLVYLVLALINAYIYCYLG
NELIIQSEGIALAAYESTWTSWPVDLQKDLLIVINASQRPLKLSAGGMALLSIQTFSQALYNGYSIFAVLNDAVN

GRs:
>DpleGR64e
MDYATTYNRPVKKIIKSGVSPLVFYSSTTMITILFLHIAMKWPNLCRYISCTEAIDPLNDYALVRKCNVACLLVLS
FAFVEHGLLELSGFVFATDCSPPGKVYETIIVNSFPWFNNIVNYNLNLGIITQLLNIQCTFNWNFSNLFVINISLY
LTSRLEQINNRISAIKGKAMPSTFWRNVREDYNRITSLVRKVDKVIGGVIFISFANNLFFICFQLLHTLAFTYLSG
RCAYTLLTIVLQTLLLNILFLLKYFKDTNTSIVGNTPLVFYSSTTMITILFLHIAMKWPNLCRYISCTEAIDPLND
YALVRKCNVACLLVLSFAFVEHGLLELSGFVFATDCSPPGKVYETIIVNSFPWFNNIVNYNLNLGIITQLLNIQCT
FNWNFSNLFVINISLYLTSRLEQINNRISAIKGKAMPSTFWRNVREDYNRITSLVRKVDKVIGGVIFISFANNLFF
ICFQLLHTLAGEVKATPSCHTPDQRIFRGYEQATYFLYSLIFLIVRSLALSLIAARVHTVSRQPVYTLYELPSADY
GIEVQRFIDQIHGDTVALTGLQFFKVTRGIALAIAGTIVTYELVLMQFTGVNPTVDIYTDDSTKV
>DpleGR43a
MNDDRLASTYVAVIAGFAIDLQTDPSRSLRTSTPTSCLVWVSNFTIVIMVASAGVYQGSETFATNIKTLKQMIEIK
TFIGLKAETDSDRNNRFTVAFISSGLCFLLISDLFSWVKEAIYSDKDVLTLIMYSFLYYEYILIELIQLQFVLLAW
ELRFILKNINEDLKKKTSNALPTKGKSFTRYLHRSNPIGITALNNYVTALNRPKCQSLLLKPESKPSYGASFTDES
RDDELEVISGGGIRDSSVAYGLACDVLRQINDNYGIVLLFIVLSFFLQLIITPYYMLTTLYTSVDWRLNLFFQCCW
CIFHICNMILVVEPCHRTQQEMDQTQILVCLILRSETINKFMLAELDLFYKQLTLNRPSYTPMGMCTMSRPLMVTI
IGAVTTYLVIVFQFRTFELHLKH
>DpleGR24
MSVYKNNLYPQIKNIPNGFAKQISDRPNNKIVFLDVPSPRTEQLNHITTNSIVPLRDNLISPQINSDIIYENIKPV
FTLLRIMGVLPLTRTSPGHNQFKMLSPSMVYSIFCFLGLVSYVMYLSLHKVQILRTTEGKFEEAVIEYLFTVYLFP
MMVVPIIWYETRKIANILNGWVDFEVCYKKLSSRVLPIKMYKKALTMAVVIPILSTSSVIITHVTMVDFKVMQILP
YVFLEILTYLLGGYWYLLCETLSFCAGILAEDFHQALRHVGPAGKVAEYRALWLRLSKLAQDTGLANCYTFTFVNL
YLFLIITLSIYGLLSQISEGFGIKDIGLFVTACCSIFLLFFICDEAHYASLNVRTNFQKKLLMVELSWMNTDAQTE
VNMFLRATEMNPSQISLGGFFDVNRNLFKSLLATMVTYLVVLLQFQISIPDNTQDTYEDEKLITNDTTATDATKIT
TTLATTILTTLAKKKKKN
>DpleGR64a
MSISSGQGRGPFTCTSLSISLRRARNHRIKQMTKCTFETQIEVFEGSMRYFLLVGKCLGLFPLIDITSYGRNDIST
RHGVPLCPNNNATGPYYKYEQMVTNAYFIYSSLFVVVRTFFVSIIAAEVHSVSLEVAPVLYNVPSPTYCLEVHRFV
EQIHGNKVALTGLKFFYVTKELVLSMVGTIVTYELVLLQFT
>DpleGR5a
MTGQILMTIMCIYNTVYSKASLNIETPVIFYGMTCITMAMFLRVATLWPKLVRHITAVEESDPNYDTSLNRKCNVT
CVIILLLSSFEYVLSLLFAFVKASELKNKDSWCESFAEYLYPWVFNYLPFSPLLGVMVQLIHFQATFIVIFSDLFI
ICMSYYLTSRLRHINTKVLVARQKHLPEIFWGRTREDYTRAVKLVRKVDDVISGIIFISFAHNLFFVCVQLFHTLE
RSSALGGYKTETYFVFSLIYLIIRSVAVSLITSQINTASMSPALVLYDVPTSLYCIEVQRFLDQVTGDKIALTGLQ
FFRVTRKLLLNIAGTIVTYELVMFQFDTTTSNELGTPFPSTNLMNNRSFQRSNLTG
>DpleGR22
MIPDHLFDEGINNSLLRNDMKHVHLNRIVYNKTQKDYERDQRNLLSSQDGDTCEIHDQFYRDHKLLLVLFRALAVM
PITRSRPGTITFSWKSRATTYAIFFYIVTTIIVLVVGYERLMILRSIKKFDDYIYSVLFVAFLVPHFWIPFVGWGV
AHQVAIYKTNWGKFQVRYYRVTGENLKFPNLKTSIVIISVGCLLLAVCFLLSLCALLDGFLLRHTTAYYHIITMIN
MNCALWYINCKGIKIASQSLSNCFSRDVSIECTASLISSYRFLWLNLSELLQSLGNAYARTYSTYCLFMFFNITIA

VYGALSEIVDHGFRFSFKEMGLIVDAAYCSTLLFIFADCSHKSTLKVAAGVQDCLLSIDVLSVDRPTQKEVAAGVQ
DCLLSIDVLSVDRPTQKEIDHFIQAIEMNPAVVSLKGYAHVNRELLTSVCINS
>HarmGR1
MNKEEHGFRVYNTNTVHKNETRKREMFQRIDEKDGIKEYDAKDLYGPEITDKDGALLDAHDSFYITTKSLLVLFQI
MGVMPIMRVPKNAQTTKRTTFNWISKATLWAYLVWSLECIIVVKVGRERLANFQSSANKRFDEVIYNIIFLSILIP
HFLLPIASWRHGPQVAIFKNMWTHYQLKYLKITGTPIVFPNLYSLTWGLCVFSWGLSFAVILSQHYLQDDFELWHS
FAYYHIIAMLDGFCSLWYINCNAFGTASRGLAMNLHKALEAEHPALKVAQYRHLWVDLSHMMQQLGRAYSNMYGIY
CMVIFFTTTISLYGALSEILEHGLSYKEMGLFVIVGYCMTLLFIICNEAYHASRKVGLEFQVRLLNVNLGAVDRST
QREVEMFLVAISKNPPIMNLDGFTNINRELFTANVSFMSTYLIVLMQFKLTLLRQSARKTLKTIVRAVFNTTTTIL
DDDFTDDVDEE
>HarmGR2
MTIPDHLFDEGINNTLLQHDMRHVQQNRIVYEKTQREYEQEQRDMLSSQDGDTCEIHDQFYRDHKLLLVLFRALAV
MPITRSRPGTITFSWRSTATMYAVCFYIAATAVVMIVGYERIMILRSIRRFDEYIYAILFVIFLVPHFWIPFVGWG
VAHQVAIYKTNWGKFQVRYYRVTGENLKFPNLKTTIVMISVGCLLLAVCFLLSLCILMDGFLLRHTTAYYHIITMI
NMNCALWYINCKGIKIASQSLSECFRRDVEAECSAKLISRYRYLWLNLSELLQSLGNAYARTYSTYCLFMFANITI
AVYGALSEIVDHGFGFSFKEMGLFVDAAYCSTLLFIFVDCSHNSTLTVAAGVQETLLSIDVLSVDRPTQKEIDHFI
QAIEMNPAVVSLKGYAHVNRELLTSAISMIAIYLIVLLQFKISLPRDPQIVAT
>HarmGR3
MTVPIPNGFPVQINSKPKNKIIFLDVTPVSTPIKPHSPNVVAPMRNNLVAPHISNDIIYENIKPVFTLLRIMGVLP
ITRPSACVNQFQIASSSMLYAILVFLSLVSYVLYLSLHKVQILRTAEGKFEEAVIEYLFTVYLFPMIAVPLLWYET
RKIANVLNGWVDFEMVYKQLSGRTLPVKLYKKALAMAVIIPILSTTTVIVTHVTMVHFKPMQLVPYVFLEILTYML
GGYWYLLCETLSICANILAEDFQNALRHIGPAGKVAEYRALWLRLSKLSRDTGIANCYTFTFVNLYLFLIITLSIY
GLLSQISDGFGIKDIGLALTAFCSISLLFFICDEAHYASHNVRTNFQKKLLMVELSWMNTDAQTEVNMFLRATEMN
PSQISLGGFFNVNRTLFKSLLATMVTYLVVLLQFQISIPDESQNRDEEEEVPYNITSATTEAMTTSTTTIMTTVLT
TLAKKKKKN
>HarmGR4
MEIKLCKLFVVLTEGIEYMKGNSVKNLNEKKRDDFLPTLNNVFLKARFFGISGYGLTISFFWSLILFSMLVVMESV
AIWRVVTLLGEWLVSASNNGLIGRLSGAIFYGNALISLFLSSKFVHSWRSLSNYWLRMETSTALDFPADVRIRKRT
IYITAFVVSVAVVEHILSMISATGVGFPPEEFLYRYVTLSHGFILKAQDYTIWKAIPIFVLSKLATALWNFQDLII
ILISMGLSSRYNRLNLYVRHVVSVEKQFESKQRFGTELYLQIQVWRRLREAYVRQSTLVRMVDRNLGSLVLLSNIN
NLYFICLQIYLGIHKSSGSTISRCYFLFSLGWLIFRACSVVLAASDVHLHSQRALKSLHACPSAAYNVEIKRLQYQ
LAHDFVALTGMGFFSLRRELLLEVAAAILKYELVLIQYDK
>HarmGR5
MQNGWNNVISNISVGSVNTVNYLFRTWERLAPNRNMDLYSLEKFKKYKNDWNYPVHVRYQDQVMAEKEKPRMTFQT
AMKVTLTIGQCFGLNPVQGIREKDASKLRFKLLSGRCLFTFFSLIGQFIMAFVLFLSLFKETSSTVDTASNFGFTT
TILFFRIATNWPKLCMHIAKVESVDPNTDNKLGKKFNIACISILFLALMEHLFSELHGISIALDCFPDTPVYESFM
KLSFQWLFGFIPYSDFAGGMAHFSNLQCTFNWNFADVFVICMSMYLTARLEQVNQRIIAAKDKNSPSSFWRTMRED
YNRSVHLVRQVDKIIGGVVFMSFASNLFFVCSQLLHTLAGGIKASQRCKPEIGADRRFFYGYEHSIYFVFSFSFLV
IRSLAVSLTASKVHAASLEPAYSLYDVSSANYCVEVERFLDQIHGDTVALSGLQFFHVKRGLVLTIAGTIVTYELV
LMQFTGITPTTSPESVSGVIK
>HarmGR6
MGQTSFRRNMSFWIPVKKNKVDVAKPKVKNITSFQDALRVTVIIGQVFSLLPFVGVFTNVASNVKFVKTSWKCVYS
LLSLVGQMFMAVLCINKLAKTTVSLNGTSPVIFYVTTCVTMMLFFQVARRWPALVQHISKAEDMDPNFDCSLTRKC
NITCAVVLILALCEHILSLLSAFAGASACYSGMDTYEGFVTHFYPWVFSYLPYSIVLGVITQFLHFQSTFIWNFSD
LFVICMSYYLTSRLEQVNRKLLAAQGKYLPEIFWRATREDYCRATQIVRKVDEVISGVVFISFANNLFFICLQLFN
TLEDGLKGTGECTPKLKKIVVSKSGPLGGHEAAAYFLFSLVYLLSRSVAVSLIASQVNSASSVPAPVLYDVPSPVY
CVEVQRFLDQVNGDKVALSGLQFFSVTRGLLLTVAGTIVTYELVMFQFNSSTPTLNITSPTVVTHTITTLAT
>HarmGR7
MSSRGFGQFLRDGNLILPEQPNHDDFLTVMEKVFKWSCLIGVLGSKRHINYAWSGFILLVLLFMESQAIWKVIKAL
AGWAIDTAGQRSVTARLAGTIFYTIAILSLVLSSRLYRSWGQLSALWARVERIMAVKAPPDKTLKRRMYFFLGFMT
VCSLLEHIMSVVSAIGLDCPPALIIKRYVLISHGFMILRHEYSDWYALPLIFMSTLASLLWNFQDVLIVLISMGLT
SRYSRLNQCLAKICALERKQMDSDKKNEATKVYAWRKLREAYVKQAMLVRKVDDAIGSIIILSCFCNFYFICLQLF
LGITQSKASEPIKTAYYFLSLGWICFRVLCVVLAASDINVHSRLGLKYIYTHDSHSYNIEMGRLQDQLSKDYVALS
GKGFFYLSKSILLQMAGAIITYELMLIQFDDQGTDDVQLNLTKNAIGV
>HarmGR8
MSSKEFKQFLRQNKLLLPQQPFHDDFLDVIEKVFHWSCFYGVFGSKRSISLIWSTLILGSLVIIEVLAIWKVIRAL
AGVARDMSGHRSVTARLAGTIFYSISILSLVLISKLYYNWRTNIAGLWGKVERSVGVKIPVDKTLKCRMSFVAGLM
TFCSFFEHALSILASVGFDCPPSLILKRYVLVSHGFIFMGQDYSEWFAMPLVIISTIATLLWNFQDQLIVLISMGL
TSRYRRLNECLAKFCELEKQHMDSDKKVEAVKVYTWRKIREAYVKQAMLVRKIDVALGGIIILSCSCNFYFICLQM
FLGITQGMSTDFLTGVYYMVSLAWLCIRVLSVVLAASGVNTHSKLALNHLYTYETHCYNVEVERLQDQLTKDYIAL
SGMGFFYLNKTILLQMAGAIITYELVLIQFDDQGNDGIALNATNI
>HarmGR9
MGVESAKVEEVTAAPVPSESGARPSRPTHCVVGGAHAFILRISSFFGLAPLRFESRSNGFTVSISGAMCVYSYILV
TVLVICTIFGLVAEINVGVELSVRMSSRMSQVVSTCDVLVVVATAGAGVYGAPRRMRNMLKFMENIASVDTSIGGQ
YSLVTERKLCGIILAILIFFSILIADDFTFYALQAKKLDREWDVVTNYLGFYLLWFVVLILELQFAFTALSVRARF
SAVNDALALTARQVSIPVEKPKSSSPLNIYAIRVAPVDSQRSANVSLLVDTMTGREHVVIIKRTASGEPRLVVSPC
DAVRRLAALHGTLCDVVNSIDDSYGLPLVVILISTLLHLIVTPYFLIMEIIVSTNRIHFLVLQFLWCVTHMLRMIV
VVEPGHYTIAEGKRTEGLVCRLMTSAPSTGVLPSRLEIFSRQLMLQSVSYAPMGMCTLHRPLIASVIGAVTTYLVI

```
LIQFQRYDN
>HarmGR10
MEYGLDAKISKELESINFRVLQETQLDDEAKKRSTKPWIEEDNKLVGKRRIDVKDQFTAFQKAMKVLLVWGQTIGL
NPVTGILQKDPSKMRFTVYTWKFLFSLTVAVAQTIGTTLCIYKLFREPTSISALGFVTFFTSTCFTTFLFILIASK
WPTLMQDIVRSKLDEYVDKKIITKCRITCCIFIGMALMEHFLSILSRVARVIECSQNETDHGEVFVKVTSPWLYDL
NVPYVVAVAVMVQYVNLITTANWNYSYIFIVCVSMYLSSILNQINKRIALEAQKTHVPAKIWINLREDYTRATHLV
KRFDDVISGIVLVTYANDLFFICLQLYNVLSNMSKAAQLVNKLCPDQDGTFRAYSYPAYLIYSVLYLLVRFLTVSI
VASGVNTASLLPAPILYGIPTTAYTKEVERFQNQVNGDVVALSGLHFFYITRDLVLTLQN
>HarmGR11
MPSKLFLKTFNMATRHRLDKREICGLHSTVRGTLFCSRVMGLLPVSGLTCPTSRRLRFTFRSPYTVLYVASLFGQL
LMFVMTLCWLMMNGISLANITNAVFYTSSLISSLILLHIGRCWPALVGSVETLERELPPFHRNVASISNVTTIFIL
TAAIVEHLLSVFYGLKVACACDSNNVAENYFRFNMPWIFDYTPFTIWKGALSELFNIQSTFVWSLNDLLIMVISIY
LTEHLLIHNELLKKAAEQEHFSCLEFRTQYLKIVRLVKLINGQFGIYILTSFGSNLYWICTQLFYSLSRTQTGHFI
TCTFKDSPTKVPPANEYENPLCPWMLGEKGALNGVEHSIYFTYSFSFLLLRTLLVLLLAARIHSNSVAPLYVLYGI
PSSRFHIEVERFIAQINNLKVAMSGLDFFYVTRTMILTLLGTIVTYELVLLQFNR
>HarmGR12
MKVHQLTMRRSNKRCGLHVCLRHAMRLARWTGFFPLQGLGQAYADGTRYKILSLYFIYNFTTLLGQLVMSCFAILL
FFQTEVTLNSISNVIFYVTSLISAVLFLKLAKQWPRLMARATETEQGLTELKLPNKVIVKCCVIAYVAMALALVEH
ILCTSFNLTFVMHCLKEAGITTNVMENYVVHRMPYVFNYVPYSLFWAFLFEYLCLQSTFVWSFNDVLITCFSIYIT
AYFRSLNQVVTANSKKDKDNMIPWSTLRVHYSKLVRLVKEIDNHISSFILLAFFTDLFYICLQLFNSLHRNYASFK
FCNELQTKQALTSPSYLLYYLYSFIFLVLRATMLSLFASNVHCAALEPVHAVYDVPSTLYDNEVRRFQLQLHHTKV
GLTGKFFYVTRNMVLKVIGTIITYEIVLLQYTITPNPYYNGTKVILNISSHSYS
>HarmGR13
MSAFRDTECVVTGTLSMMLRMSQIAGVAPLSFRRTHGGWYIRTSRAANCYGKALSFCLWFLSSFTIAIDILIQPER
SFRTRTNSTRIVWLADVATVAIVVCAAAFTGISRMRCLTVYALKLEEINLRLSLFHEEPSNEANRRLIAVSSMIFV
VSTILVDYSIFIYQVITEHGKIVTSCMYIFYNISTIVQQVILVTFSETVTSVLTSLQMLNNCLKNLLQEILDSSEL
TNCALNYDSYINMNPNRSAIPNKSINSVVDTMAVYKGYRKNSIKAVPSTIRRLALLYCSICDVIRLVNDSHGLILV
ALMLCLLLHLVITPYHVITNIFNKERSDRSSPLLQLNWAVLHFVNLLLIVEPCHRTHEEMEQTRHLISQMIRYTPS
EHGVLLTELQMFYQHLILNEVSYAPLKMFSLNRSLIVTVI
>HarmGR14
MRKLNLTPLKYLLIIENLTCVFRNYLCLRKCTRCLVTIWVVFETGHMFFNVYYNIVHGQNAELKAQRIYFFSSTAF
SIYVMTSSLYFSKRFYKLLSNFDEFYNIFEDDVYNKKMKRAQKVMIWMVVCFIGIKFIVFYIMQVKTEDLENGLVS
NTVSEYSVTVNDFRYIFQYFILDCILLIVAEQLRAISRSIDSELSAMMENQRNVGHVEGLPRVVLNYEKINKWAQA
YESINESTHLCNSMFSVQLTIMLLIVTAYYIILLYSIAIITVEGVHTVKTLVMNLFSMSVFLLALLVISRAGQKIQ
NSSQQLRQRLCELCVHTLGNEEYYKLAKDLLRCVRTRPVRIHVFGTLDVNMSMLPSIVVLFTSYTVIALQFNNVL
>HarmGR15
MELVKLEMFIKAIMIFRIVCGLYCKITSNKIIAAIIKIYCALVIVIVFVISLSYLRALKNSASSAFAFGMPASKYF
VNSVVHFCFDGDNFYEFFHSLKNLQITQDSELKYDIPITIFLFISITGARLKNYSRYYWLGMSDGHFRFDRFMSFC
FGVLISYIGSITCVMRFMMFELLWRRMAMLRKRLEQDLLNARRFENEEGVLRKQLRACLSIYKGILDSTRKNDAPM
KLLVTHLYIIFLNNVFQY
>HarmGR16
MAFVKSETFIKVILIYRILSGYYWKISSNKLVSVLLRIYCLFIATAFLCSMYQFYLFVSIPGKIHMCCICYLFSIN
VATNLIFNGDNFVYFLQEIRNVNIPRHIGCGDKIPITTVLIILTIFLRIGSRIQFDDHTHIDIMQGVFSIQNFSLT
FDFLYYSSYLTRIMMFELLWQRITMLRKCLEQDLSIARRFEDGEQLLRNNLRACLKIFRSLVNTTRVCDAPMKLLV
RDSVLY
>HarmGR17
MALVKPVALIKLVMFIRLISGFYCKISSNSKINFLVKTYCILIATLIIFLTITVAFVRVNIESKCHIGFMSTLYII
SVVTSICLNGDNFEDFLTKIRGITTLPNVESADKITFSIFLFFLSLCSRIVVNVKFTIDNVQSISDPLFYVSLVSL
TLATLQYSASFTRIMMFELLCRRMVILRKRVESDLSIPMTYQAKEMVREKIRKCLHTYKSLLDTIRGTDMPMKFLV
SFLLKNLIEINRMQRVYKPGVHAPQIPSV
>HarmGR18
MFIKAVMIIQIFCGLYNKISSNRIICAITKIYCALVIAAIVIIAVIHIFFFNIFIVAKSSVVISVTTYVLYAMVHF
YFNGDHFNEFFYSLKHLQTTHGSESKYDISITIFLLISISSARLYGYLKFTFMHISILPIDEFTGLFLDLLVCNIS
HTTCVIRYLMFELLWRRMAILRKRLEQDLLNARRFENEEGVLRRQLRSCLSIYNEILDTTRKNGGPMKLLVIHLYI
IFLKIVPLKLTNITVVLLVPQTLNHMFGFLLKNAIGSELTVVDMYILFFETLSPALLAEMVQSEIECMKLSIVKQL
LVCKDERTLNAIQDATTYLEQNPFKYTIWRVFAVDMSLILNMIALLTTYTVAMVQFAHFYD
>HarmGR19
MASVKLETFIKSVIFLRLFCGLYYKLSSNKITVALTKIYCTFVAKIIITIFVLFIKNLKVPFQIYLSFGLMCSGYL
TNVVFSAYFDGDNFMKYFSALKEIRNPQDFPSFSDIKISIILLFFFASSRIFNYAAYSVSAMFTFQNPFPYNIYTT
TAFIGSIAVLHSLVNSTRMMTFELLWRRMAILRKRLEQDLLKARRFENEGDILKNNLKQFLNTYKSILDTIRISEK
PMKLGVTVVTNNKSSYLNTNHIAVNILR
>HarmGR20
MVFLKQEILIQILLVYRLICGFYFRISANKIVAPLFKVYCIFLASIVIAKTVWLIVNNKLDTLGTFHMCSACSLYV
SHVAINLFFNGDHFIDFLEKIKNINEEDTCAPRDKILISAFILFITTLVRFYSYVQLEYDFFKRFFGSLDSVFTFS
LLVDVFQYSSTITRTMMFELLWRRMGNLRKRLEQDLSIARRFDEGDEVMKEKLRLSLHAYKNLMKITFETCTPMKL
EVIYMRFVIKNLKLSFILKRLYRRF
>HarmGR21
MEATSVFGFHRSVLLSANSITMAFVKPETLFQVIMVFRAILGIYHKISPNKLINAFLRVYCVIVAIVVHLGTIYIM
```

QFFNLTEVHLIILNLFYSVDAVMGLCLDIDYLKGFLQKVRNMDTEHFEMKLKAPVTVFFIFYILIARFYCHTKFRY
DNGESFFSPLFSEASLGLIFDLVRYSSIWIRILMFELLWHQMAMLRKHFKTELSVARRFEEGDHMREKLRSCMTSY
KNLLNASHKFDPVVKTQVRV
>HarmGR22
MADIKPNQMIKAVMILKIICGFFCKISSNKIVITLARVYCLFFGLVCMSMLIILANHAKSFSALLQVFFGVYVYLI
YFLLNFYFEGENFMGFLHKIRKVVTSQHMKPCEGVQISIRLFTISLAANILLRLKITLDQSHYTFSSTYSFLAFVS
TINLLEFSPILTRLMTFELLWRRMAMVRRNLERELSTHQGYENSECILKEKLRRCLNVYKCLLEVTRVNETPMKIA
VYNQKLKYARRSQRLSLIS
>HarmGR23
MVARLMVGFYWKTSSNKLVNALVKTYCVTIATVFNIMSITYITNEDEHIPTANTIYMAFVICLYDIIVVVNLFYTG
EYLEDFFDKMENPAIAQDMEAGDKLIVTTAVIIFSLTTKFITGSIYIYEKFTLTNTSKNFILLIMQIINPISNFTR
IMVFELLWRRMAMLRKSLQQDLTSARMFEGEEIFKTKLRTSLIKYQLILDTLKKIKHPIQFLVIVSFMNYVKSSYH
MPDKKKLFSVVILFSDFVKYSSLYSTCTASYVWFYCIWRS
>HarmGR24
MVARLMVGFYWKTSSNKLVNASVKTYCVIIAIVLNILSINFITSEDEHIPTINLINMAFVICLYDIIVIVNLCYAG
EYLEDFFDKLENPAVPQDMEAGDKLIVTTAVIIFCLTTRFISCSKYIYETYIALNNTAIDIHCLLLIMQIINPISN
FTRIMVFELLWRRMAMLRKSLQQDLTTARIFREEEILKMKLRTFLMKYQLILDTVNKIKHPIQFLVIVSLMNYVKF
SIHMRDKKKLLSVVYLIVFRFFQVQ
>HarmGR25
MVARLMAGFYWKTSSNKLVTALVKTYCVTIATAFNIMSITFITSEDEHIPLLNQFYLAFVLCLYDIIVVVNLRYAG
EYIEDFLDKMENPAVPQDTEAGDKFVVSIAVIMFCLTTRFMFYSKLLYKSLIDPSDLSISRNFILLIMQMIHPVSN
FTQIMMFELIFRRMAMLRQSLQQDVTNTRIFGGEEILKMKLRTYLMKYQLILDTINKTKQPVKYLVIV
>HarmGR26
MDVQATPASIKTIMFIRLLCGFYCDISANKIVTVLVRVYCVAIITLVMAFGIYLWNGIIGISSKIHFLFITTPYIT
SMVTNICFHGEYFSEFLNKMENFNLTHGFLSSIKIPISSLFFVFVFLQRFLFQMKFTFDAIGLPFRGVLTHASFIL
ILCLMNYTAEFSIHIMFELLWHRMGMLRKRLEQDISTARILRDGEESIRENIRTCMRRYQHLLETARVTDGPVKFL
VDTYIFITAR
>HarmGR27
MYSVNAIIIYRFIMGFYTEFSSNKIINFISKIVCVLLNIFITTKLFSLIDYNIVSSFIAFAGYSIIYLTNALASLF
NSKWIQTYVNDLKSIGNAMGTGADIDIPIIRIILLCLHGLSSTTQIVTCEHEFCSGFYQKINGFVYSLATAMATIL
HMVVFELLFHKVRANRKYLENQLTIFKRHQNIMDLKNNLRLCMSNHVKLIDSLDKTDKSLKTTFFVADLAVIPIIM
SRVFEIVKSPDRSALVNILLIGKGVVLRSMPGILADLTSREIDKMKHIIGQNLLVYEDDDVKYLLKDTHLFFEHQP
FQYTVWRLFSINTAMNVVAFKMVVSYTLAMIQFAHFFG
>HarmGR28
MPSFRESGLESFFTLKGILIMRSIFGYYYKFSNSVPISVLLKLYCIACCIIIWCHIFVWSDVNSRAAFYDVTLLIE
ITINMLISLFVEDEFSTLNDLIASLPTKQNLRLTYAVIICTIIEQVTAFTYGRSLFTAEDAALYMFQYISCTYGRL
SLIYQAHNNEKAIKTLCDSLKDKIEDMNMDAAEKRGHVEKFIDTFKQIINFSFDAKCQVMKFKVAVLGYVCDFFRV
QFMVYYIYEFHLKIQVTLMLPWYCGVAYSIFIICLPSMLGELANYHLDEIKVVLVDEVIRNEGTYYFVIILGLRFD
>HarmGR29
MPSFRESRLESFFTLKGILIMRSIFGYYYKFSNSVPISLLLKLYCIACCIIIWCHIFVWSEVNSRAVFYDVTLLIE
ITINMLISLFVEDEFSTLNDLIASLPTKQNLRLTYAVIICTIIEQVTAFTYGRSLFTAEDAALYMFQYISCTYSRL
SLIYQAHNNEKAIKTLCDSLKDKIEDMNMDAAEKRGHVEKFIDTFKQIINFSFDAKCQVMKFKVIQFISTSHIFVK
FKIMQ
>HarmGR30
MAYLQKNLHIVKFLEPLMSVKTIMLIRISGGFYHKISSNKLISCITALYCLAITAFLSVNLLTLNYPSYFILVKNY
VGFLIYYLLVGICLLTDSGYFQNFLNEIKKIDHMLGASSNIKVPISSFLLLGIITGVTGMDVLYVIYEVENNRQEK
GFFITLFCMSLLVQTCAIANYTNVMAFELLWYRMRIFKQFLENTLKREFRSQDEEIKINAVRNCMVLYQRILDTVN
KNNLPMKLLTFVLVTKFIPKTVATLYDIVTNAIPEVSYMLIHEFFVDFMVLCAPAIFADLICGEISSIKSMFKKQL
LTCQGTFTNCYLVLAKKRSQ
>HarmGR31
MDKSLYKLLLFRFFFGHYFKLSSSKRICFIAKIFCLFTVIYISVIFVKFFLLFGSVISSAINLWILLIEAILSIFL
SLHTEEAYVLKFSAKIKNYVSSSSTCRVTNVLAFLIIPINLFYLLAAYQYEFSITNNLLYNITFTVCYCSYLTSLY
ITEMFASAINNLTSDTVIKLKDVDITDEEKRFCIENFLDNYLKLMNIYNVTATVSRIKVSV
>HarmGR32
MPWLKGTEIESFFTVKGILLFRLIFGFYFKLSNSIIIDLLIKSYCFLCMILMWLHIIFVPNPENPAARVYDTLLAI
EITLHIFLSLSKNEFGLDLIHSFDSAILQTKPRCLISYSMIFPVVFLEIYVAAFIINWNETKFINITMFYYQFVAC
YASRLAAIFQSENYAHATKTLYVQMKENFESNMNSAGKCEHVQQFNDKITITVDFFDKHRNVTRFKVKHLCISIFF
FYIKK
>HarmGR33
MTDDTLVCDIKNVLFIRFALGFLQNFNGTSKTRWLSYLYTICFLLLFAVLSLFPNEVIYVSYRILALIEYFFLFMI
SFLSKEEYIYESYKLIYGLDTIPGAKLIFQNLEYCLKVYFFVSVFTGSFFTGISICFRIQEACSITNSFAVILTIL
DRTARDIGAYSLIMFIGLLYSRVKLLRNYLDTKSANTAWDRYSVKQYINMYESLTNTIDDSAVPVKVTVCFSCTLL
L
>HarmGR34
MTDDTLVCDIKNVLFIRFAFGFRQNFNGSSKIKRLSYLYTIFFSLLFTALTLFSNDLSYHSLSYLILALTEYFVLF
TVSFLTKDEYIQRNFKLIYGLDTLPGAKKIFQNLEYFLKVSFVLGLANILFFATMICFRISGLCSIANLLSFFYIL
LHRLACDLGDYVLIMFIGLLYSRVKLLRNYLVTKSANTAWDRYSVKQFINMYESLANTIHDSAAPVKVTVCFSMYS
SSLELSIN

>HarmGR35
MMIDSLVSSLQYIMFLRFIFGFRLDCNSSPQMRLFTKLYPVLFFIVLNFAVWSSENVLNPTLRYSTVIEYCAQFFV
SFLAKQEFVNKNYQFIYGIDSLPGAHKNFKRLRVFIKFFCLYYILLRLLAILQLVVYVGPLLKNVTADIIYFHVCD
IGRLNIFLVFCILFCRVKTFEMNFQTITDVVVLDKYSVKKYINMYQTLIDYVESMDMLFKLMVIFFIRYNDRFR
>HarmGR36
MIGDSLVCNIKYVIWLRFAFGYLPNFHGSPKMRAFSYFYTIFLFISFTTIVIAPFYKFPWFFRVLALLEYTTHFLL
AFVTKDDYLYQSFRFIYGIDTNANVRKLYRNLEVFFKFIILYFLANKILVVMMLCYRLPSICLFSNTLDFSVNIII
RLACDMGRFTVILSIGLLYVRSKILKMNFLTQSPNTICGRHSVRNFINMYESLINTFDKIKTPTNITVCFVITY
>HarmGR37
MIKNNLVVNIKPIMIFRSLFGYRQKFNSPLKSNFFDVFSLISFFMWSLSFFFKTYYFPLVYVMSDNIEYCICFFVA
FFTKEEYIYFFYKRQSSIDNLPGAEKLFSRLNLILRAFVVYCFLTKIVVIIILRVFAPEIFQLGLYLDIVMGLSQS
MSCDMGRFTIILMVGLMYCRMKIIKDNMDMIGSDLRNRFVARNFIQMYQSLVCSLQRIDVPLKVSVSPLFSINQK
>HarmGR38
MTDDTLVCDIKIVLFIRFAFGFLQNFNGSSKIRRLSYIYSIFFLLLLTALLLAHNELVALSYRIMALIEYLILFMI
SFLTKEEYIHQYYKLIHGLDTYPGAKKIFQNLENFLKVSFVLGLTNNLLCASFICFRYPKTCSIATPFFFVPIILH
RLACDVGGYTLIMFISLLYSRVKLLRTYFDTKPANTAWDRYSVKQYINMYESLTNTIDISAVPVKVTVCFSMCSPS
LVLSIN
>HarmGR39
MNSNKKVCNLHHILYFRLIFGYYFKQPTLKLRILTKLYIALFISGLTWLFITKVNGMLYYLQYCDILEYITFIIYS
LITEDSSLLRSYEEKIKIDSLPVAKQYFRQLEKFLYLLLFLICGLRLFASSLFCIYAFEICKMAPFGVSVTNTFLT
AMDWRHLNTVIWFSLLQTRVKILKNTLEIQGFDRGPMQRFSPRMYIKMYEELVGFSEFNGYAMKSIVRITSH
>HarmGR40
MTDDALVCDIKNVLFIRFAFGFLQNFNGSSKTRRLSYLYTICFLVFFAVLSLFPNEVIYLSYRVLALIEYFILFMI
SFLTKEEYIHQSYKLIYGLDTIPGAKKIFQNLEYFLKVYFFVSVLTGSFVIGIVIYFRIQDTSSITNSLIITILDR
MTRDIGAYSLIMFIGLLYSRVKLLRTYLDTKSTNTAWDRYSIKQYINMYERLSNTIDSAVPVKVTVCFLMHYPSLE
LSIN
>HarmGR41
MILKYLYKINSLLKIIYSVNGIIITRFTLGFYTEFCSNKKIILLSKIYVVLTILNVSVNHIYTKEYQNISATLSLS
FFAIIYFMNSTASLIIDSKWIQKYVNDLKSIRQSMVADKCFDIPISRIIMIIYQAFTLMMILVYCHYDICRHHIYL
RVIEGYYAVTSVTTIVPTLVVLELMFYAIRENRKCLEKNLSKFNTSGSVDDLKKNFRNCMSNYTKLRDCLKSTDAP
VKTMVSMNEV
>HarmGR42
MTDDTLVCDIKNVLFIRFAFGFLQNFNGSKIKRLSYLYTICFFILFAVFSLFPNELIFLSFRILALIEYFILFMIS
FVTKEEYIRQSYKVIYGLDTIPGAKKIFQNLEHFLKVSFVLSWTNNLCFSSLICFKIPEACSISYLYSVSPLLLHR
LTCDAGRYTLIMFLGLLHSRAKLLRNYLDTKSANTAWDRYSVKQYINMYESFTNTIDISAVPVKLTVSYSMFSPSL
ALSITLSR
>HarmGR43
MCILYGFSYIESEDPLYTIIFTFVDYMSCDLGRFSLFIIFGLFYCRTKGFRLNMEGEGTDIPWDSYTVKKYMNSYQ
ALYNSLFIAYKIIITVVRFKAYLFVARLFLQVG
>HarmGR44p
MIGGNLVCNIRSIMLFRFAFGYRQKFNGSFKLRVFSYIYPITFLIICNLDIFIISTLSLYXIANILEYSIYFFVAF
LTKDEYVFKCYEFINGIDNLPEIQKYLNYLNKIIKLGPWLFVLKNSIMCIIYAFYYSESEDNPLYIIFFTFVNDMS
RDLGRFSLYLIFGLFYCRTKGLRLNMEGEGTDIPWDSYTVKKYMNSYQALHNNLFIAYKIFIKVVSYKVYLFVFFV
LQVGHRKIFIA
>HarmGR45p
MKSMIFFYCINKALLMYSLCILFPNICHGPLKLVFIADHIYGMGADLGRFGFLLVLGILICRLKTVSMSFKNIATE
VSRDKYAVKKYMNMYETLINYVEEIDTPIKFTV
>HarmGR46
MVKQFINKLKTQFQYYFGLNAVLFFKLIFGSYYDFSSSVILRRFAKAYCIFVIIAYWFVIYFIWTVNSKISLAFFL
IVMTVDAAANILFSFITEEKYVIEFSSMVLFESGLNDRNIYFYLQIFHLVIVISSYLKRYYSIYAYAVAFVNISAY
NNRIISFYVMNTFKDAVRSLRLSLSKHFKNKNLTSDQKLLQINKFLNAYMKLLRIIDKVFKIIRFKVSFCTICDII
FLPASNFLKCFVCPVLDCLCIDSRLLQNIRYIIPWISICISGKCYFALFIYLYAYR
>HarmGR47
MFQQIMIKIKNKIRSFYSLNSILFVRLLFGLYYKLSESIIIRLFAKIYCILYLILYLIFVKIYNTTDTDNFTFEFY
GSVILIETVTNILFSLFSGELYVMKFLSSLPESDLYSFGVPLNLIICHSIKVILEYLFVPIPVFIGWVYLSVHMTI
YNSRMTTVCVMDMLRQAYKSVSGTLIRNIMRRNVTDEEKITEIKIFVKGVMKLGHNMSIDINITRFKVRF
>HarmGR48
MLQQLLINLKSQFRSYPGLNAILIYRFIFGLYYDLSSSAILCLFARLYCILILIATVYTINFVKAISPLMSVTFYA
MTKIINAIVNMLLSLITAEKHEIEFSSKVLSETGLRHRNFYRNLIFCYLAVNVSVCIEHSEAPTTYPISFLNITAL
NSRATSGYILQIFKDTVRSYRLSLINNFKNKQLTSQEKKYELNKFLNSYMKLIINLERVLKIARFKVRQLNGNFFI
TGLGK
>HarmGR49
MIAKIKYILKQRSPWILKLVTFLCLFYGQYLKISESKIICGFLRVYCIFISSVIIWGFYISYTKNVIYYGFAIIEY
VFTIVIYFILYNDSIFKFYNNLEIYDRIMGFKEIPHFACYMIMFLIVNAIFRPITGFFRSTYIFSTWIQRISVGGA
LCIIEMNMFGIAHFFSLFHQRMQLMRKFLESNSVPVNITGMDEVAVSVRNVKKSLYYYDKLLDCLQSLDIQVQIMV
IVFIIFVF
>HarmGR50
MEMLSKLKLLLSSCVDHFLPILRLGFRCYSRISDSKHVCYIAKLYTVIIFIFSTSGFLYWHRSTVISILLATQYCV

HAIYSFVTSDKQAFRFHRSVKTSDAIMGFKNLPYISKPVLVPIYITVLLWMFYVYYFWIDDLHLSTYIMTICSDLN
TVTVGILLIGVFSRMPIMKIALENNFVPVNIVGKDQLQKNVKIVRKCVGYYSNLLDSFDVIHTQFQFTVSDFMKCA
SCHYIVQ
>HarmGR51
MQEVKKLTKTQISAMWLVIFFTSARAFLGCYKKISESKLICFLFRIYCIFISFTLVAYYFRDNIDTGSSSHMLVII
EYIIIVIFHMFTGDTYMRTFVNAIKMNDRIMGFKGIPTFTKYVYLLMFLGTVSKTVAGSFRYMYSLATLLRCLTIG
AILVSVDFNQLTIIISFSMLHYRMKVLRRFIDSNSVPVNITGRDKVAISIRNTKMSLFYYNNLLDSLQLINKELQF
LVKNKFCY
>HarmGR52
MITKIKYILKQRSTSKWIIKLATYLCLLFGLYSEISESKIICGFLRAYCIFISSLTVWAFCVTNTTNFISYVFVIV
KYVSAVVVFIILHNDSFIKFNNNLEINDRIMGFKEMPYFSIHGFMFFMCNSIIRTATGFSRNTYIFSSWFQKLSIG
GALFIIEINMFGFYYFFSIFYERIKLLRKFLGSNVVSVNITGRDEVAVSIRNVKKGLYYYDKLLDGLQCINMQIQM
MVNAFNIFVFDCTLCYYLYYTIAIIAT
>HarmGR53
MISAIKKYVKNDKLFMWLTKSYIFMRLLLGVYVKISESKLICVFIRFYCICIFFVFASIFTYNPENYSVLFEAAEY
VFLVIYHFICENNSFVFIDVIKINDRVMGFKETPLVNKYLSFYMVLNSVLRYTTGQLRYTYHFVSWNEHISLGIAL
ISVDISMWGSIAILTMIHDRIKILRKFIQSNKVSVNITGRDEVFISLRNIKKSLHYYDKLLDSLDLVNNQLQFQVN
VLFRIVHIKYIC
>HarmGR54
MPQYSPTAILITIQYSVTVIYGYITSEKHAIRFFTHVITSDAIMGFKNLPFVRNAVLVPLYIFYLIWGFALYYRWV
DLIRLPTIIMLLATDFNALCTGILFFGLYERMQLIQRTLENNFVPVNIVGQDQLQKNVKIVRKCVGYYSNLLDSFD
AVETQLQVMVSGLMKVIEQIRCSSLHKIATYEGVVLFLKIYSVIFLGSICVALPRAVLVAGI
>HarmGR55
MDASKKLKFILEVSIDIFLPLTRNLLGYYTKLVDSKYVCYFCKFYCVFVNFYLVTQTYYLHGFFSVRDLIVFQYSV
GAIISFLKADVCGVVFFKHLKTSDAIMGFENVSFGKRSVIAPLYFLYIARQIMVYIIWFDAMSFPVYFMCAASDLN
SLLISIYLISIFGRMKLLKKTLENNLVPVNIVGKEQLDKNVNIVRKCVGYYTNMIDAWNIIDNDMQIMLAVALLTN
TPIWIMNFYAVVMLFLQPTGQQEAVKLVQGNILSLLMATSPTIVSELISNEIDAIKATLVTQLVRCSDPSLSSELE
VALHYIHIPPFKFVICRAVPVDINMPITIIGFCITYVIVFMQFLHFSNL
>HarmGR56
MIVKRNNFIQNRQPSLWLYKFITILRCLLGNYREVSKWKPICYLFKLYCIFICSVIVVSFYLSHYSSNFTNYIVIL
EYVAIVFFHFVCGDNYIMSFFSDVKINDRIMGFEEIRLPNYVCYVGFAGLLLRSTVSIIRIPIVGNSLRYYSITSA
ILSTDINQLTVIVIFSIIQDRIKTVSTFIASNSVPVNITGRDEVAISMRNVKKSMLYYNNLLDSLQHINKQLQFLV
TTFLTILKIFGNLVGFIPCN
>HarmGR58
MKVNPATVKKQESKPNMEQLEESEYNDKIAKVMRSMKTITILEYSYGLFKFQFTNGQLQPINILIKMTAFLSIAAY
IFMLYINVCLTNGTFFLGSYTVIQFVPSMVVLLQYIISTFKNFSISESLLNIRIFTTIAKLDSLLRVEVLNDFYEI
LRSKTNLIMLIFVILHAANFVLEFLTSNKTLWLIFISYHLHMTQEIELLAFFNCVSMITYRIQLINKFLTFFNNGQ
EQRDLTVFIVREKNNEAQEKLNFVGRVSETNVKLRDLATMYDIIGKICLMINKAYNLKLFMILVTAFTFILVTIWQ
ALSFYQSPQYCTQDFVKLSLWCFSTVCNLSALAFVCERLLRARNKTRILVNKIIMNYDLPKTMRVQAKAFMELVEA
WPLRIYIYDMFSLDITLLLKFISVATTYLIVIIQIFHFI
>HarmGR59
MEAEYDNKNKYFTRMTLTKSHEKQIIETLNIFIIFEYFFGIFRFERVNEELREPNWKKKVLSFCITSTCAISFVAY
SVITLNIVMRATMREAIYINLTCITTMLLQFCSSALSSTFFFNFTNIRIITTLANLDVMLQIEDYKNFYKKCLFTT
YKYVTVVFITQVTLSIIDGFTMYLGWAVPAAFLDFSQRLTILTFCKYVDLLRRRLKIINNYLKAFTDECEKETITV
FTLRSRTNKTTQAINFIGHASDNNTKIRDLSRMYGMIGQACSMVNKIFNFLILTILFNSFIFIITIMWISLVMYRN
SSDNLGLYINVVLSFFCWISYLLFITITCQRLVSLRNKTKILLNKIVMNYDLPTTMRDQAKAFMQLVEAFPLRIHV
YDMFSIDISLMLKFISVATTYLIVVIQIFNFF
>HarmGR60
MKEKIKGKLNSSPEKFNIRSVKRIVDTINVIIKAEYFLGIFRFTIVHGSLREPNWKLKILSALIVLICSTLFLLLS
ESYYGLSSHLYRNFTDSTVINLSYFVMMAMQYSIHAITITFCFNASNICIINMLANMDTMLKAKILNNFYKKSLFQ
SYIYVLLVVSTQFSITTLACCTLNTSWSLIAGILDIVQRLEIVIFCRYIDLLRQRLGVINNYIKKFVSEQEKPSAL
VFTIRNRTIETTETINFIGEASESNDKIRDLAKIYGTIGHTSSMVNKMFNFQILTVIMSTFIFIIAIMWTCLKFYR
NNYSNTGYLLNLILSTMFWISYIALMSITCERLILLRKETKSLVNKIVMNYDLPTTMRDQAKAFMQLVEAWPLRIH
IYDMFSVDITLILKFISVATTYLIVVIQIIKFF
>HarmGR61
MQRFVMKDNNEVKAKEPSESNMEKQEENKYNDKLAKIMYTKKPVTILEYCFGVIKFYFRNGQMHAPNKIIKGTYVF
FVVLYTFVLFEYFYEPIQTNDLNSKIVLAALPGIIVFIQYIASSIKNSSNSYPKLNIEIITTFAKLDTLLQVEDIN
NYYNISRSKVNLMVFLFIVFHSVNLLLEIMTYTYQIWPPIIWFHLFVTQKMEIVNFLHIVYMSTYRLDVINKMLRT
FILENRQKDVTVFIIRKKIKNTQTKNFIGSASEDNEKIRDLSAIYNIIGNNCSLTNKVYNFNLLMSLVTAFVFILV
AIWQTLSLYQSSSQYDMKDIMKVTLWCCNTVFNLAALAVVCEKLLRTRNKTRILVNKIIMNYDMPTTMRDQAKAFM
ELVEAWPLRFFVYDMFSIDITLILNFISVATTYLIVIIQISHFI
>HarmGR62
MENNMTEEKNQENKQNMEYSEENECKIKIQKVMYTVKPVTVLEYCFAMFKYNFCDGQLQPTKTGMKIYSSLCIIVY
ALVFFRFFFMPAVGYPYITLVPPICAFIHYVISSFIAFFLSGSKAYICIFTTIAKLDQLLQVDIVKDFYKKSRSRT
NILVFIAVSIHALNCFLEIIGDLKEYVMTLTSFHLFFTQRIELASFFNCVAMVTDRLNVVNKFLDTFVTEQDKKDI
TVFIVKERKKESKETLNFIGRASENNVKIRDLAAIYDILGRTCMMINKAYNFSLLMILTNSLAFILITFWQALSFY
QSQEMDSSDLIYMAVWCMSTISNLIALAFGCERLLRARNKTRILVNKIVMDYDLPRSMRVQAKAFMELVEVWPLHI
YIYDMFSVDITLILKFISVATTYLIVIIQISHLI

>HarmGR63p
MEFQLEEKIPSYRNTNKRRGEEQIFNTLKIIIKIEYFVGIFRFTLLNEKLSRPNWRMKSISIFIITISVVWFFSFA
AYNLELPSVDDVTSYKFMNLICIIFMFLQFFASASTTFTFNTSNICIISMLAKVDTMLKVEILSNFYKKCSLNTYI
YLTFVIATQILISIIDILTVRISWAITAGILDFVQRLEIAAFCSYVDLLKCRLNIINRLLKTFVDDQEKKATAALT
IEFRSGIIENFSFIGQFRENNTKIRDLAKIYVMIGQICSKVNEIFNMQILTILIPLYL
>HarmGR64
MEFQLEEKIPSYRNRNKRRGEEQIFNTLKIIIKIEYFVGIFRFALLNEKLGRPNWRMKSISIFIITINVVWFFSFA
AYDLELPSVDNVTSYKFMNLICIIFMFLQFFVSASTTFTFNTSNICIISMLAKVDTMLKVENLSNFYKKSSLDTNI
YLALVITTQILVNITDFLTVHISWAITAGFLDFVQRLEIAAFCIYVGLLKGRLNMVNHYLKTFVDDQGKKAATALT
IEFRRGIIENFTFIGQVRENNTKIRDLAKIYVMIGQICSKVNEIFNMQILTILMNTFITMIDMMWTCLVMYRAPTS
PKLGVLINVTLSCCTWISFIAMMSIRCERFLYVRNETKILVNKIIMNYDLPTTMRDQAKAFMQLVEAWPLSIHIYD
MFSVDISLTLSFISVATTYLIVLIQVIKFF
>HarmGR65
MEFGKDQDNESNIDQDYSNQRKKIVNIYNNIKRELIFEYFTGIYRFQLIDGELRTPNWKLKALGIVIVSIYTAAFI
WFIIPDPSDCMTGLHVFLNNIDDFPCIVVLIQYIASMITCNFLMNSKNIRSITLLGEVDTILQVEKIEDFYKKIES
KLNKYLILLILTHFIHGVLDVFSSDDIVWEMTILPLYLNQKIVVLIFCSYVIMLNSRLRLINSYLREFIQEQDKRS
VPVFTVRGTKTKNEKTLNYIGRPSIRNTKIRDLATTYDIMGEICFMVNEIFNFQIFISLVTTFTFIVITIWTSLNV
YRKPDYQSSQLINVLIWCMNMICNVAAMSFACERLLVLRNETRILVNKIIMNYNLPKTVRVQAKAFMELVEAWPLR
IYVYDMFSIDITLMLKFISVATTYLIVLIQISHFI
>HarmGR66
MLDALLLGMVWAFVAGIITFVQRLEIITFCKYIDLLRRRLQIINQYLKTFANEQDSKYVTVFTMESETIKTKETVN
FIGNASESNTKIRDLAQMYGMIGHTCSMVGKIFSLQILTILMSVFILLITIMYSKQSHRLPNFNGCLWSNGKCSSF
IFLLDFLLGFHVIQM
>HarmGR67
MANVKKVEPNPKVENKKCDQKRTMLAIINTIKPMLFVEYLYGIHRFYFIKGQLRPPNWIMRAYAVIHTGSFLVLFF
AFLNFPAVFSGSMKIVEIMDEFPPIVVLIEIMSSTIIATFVVNTVNISIFIKLAAIDAKLQAESLSDFYKKSRFET
YVLLLALSVSHFINSVIDFVTADDITIKGMIVLPLYFVQKLETFTFCKYMYMVRRRLTIVNDYLRAFVEEQEKNSA
NIFTVTNNKVEKKREVNFIGRASDTNTKIRDLATMYDIIGKICHMINKVFNFQIFMTLVSTFTYVVITIWTSLYYY
RKPGSNLGELINTAVWCCSAIYTVGIMSVSCERLLLVRNETRVLVNKVIMNYDLPKTMRVQAKAFMELVEAWPLRI
YIYDMFSVDITLMLKFISVATTYLIVIIQISHFV
>HarmGR68
MDDDKEQDNESNTVKDDVKTDENPKKNMVEIINGMKTELIFEYCCGIYRYQLVDGELRRANWKRKALGTLIILIYT
IVFFWFLFMDPDDDDLHFFMSTIEELPSVVVLFQYVSSVIANNFIFNSKSIRVITLLAEVDTMLQVEKFADFYKKI
SFRLNKVVIFLIISHIVNTAMNFCSVQDVAWGITVLPLYFIQRVEIFIFCGAVYMLKCRVKIINNYLKEFIQKQDK
KSVTVFTVGKAKPKPDTTLNYIGRPSIRNAKIRDLATAYDNIGEICSMITDVYNFQVFLTLVSTFSYIVITIWTSL
NFYRKRDYRIRQLINVMIWCFNMIFNVAAMSFTCERLLVARTETRTLVNKVIMNYDLPKTMRVQAKAFMELVEAWP
LRIYVYDMFSIDITLMLKFISVATTYLIVIIQISHFL
>HarmGR69
MEDTTNEDTNYAKEKEKLISESKKQILDTMHLINVIECINGIFRFSFVNNELLPPNRIMKMLTVFCILIYVIIFVF
CFISLSPLSDDEFDVVALVIQFSITLDFLQYAACTVTATFLVNSNYIRIIDSLACLDTELEIGKLSNFYKLSRFET
YKYIFLVVFTQGLRAIADWFSDASTILYTLNFLLSFIQNVEVMTFCKYNDMLRRRLKVINQYVQVFADEQEQDTAT
VFIVKPKNEQEKEELHFIGRPSDDNTKIRDLAKMYNAIGQICTMVNEVFNFLILAFLATTFTYIILNMWTCLHYYR
IGLNDLGMLINITSSFFFWILYVVVISITCERLLLVRNDTKIQVNKIVMNYDLPKTMREQAKTFMELIEAWPLRIH
VFDMFSVDISLMLKFISVATTYLIVVIQMFNLM
>HarmGR70
MEPIDKTEIRNRAAKILHTLKPITIFENVYGIFKFNLVNGDLRPPNWKLKTVAVIYMSIFCYLYVVKHYSDQFVES
TTKWSREYIMKQVPAWFVLIQYVVSCLKASFPDKSKSDIHIIKTFAELDCLLHFETLNRFYAKSRSRTNILVLILL
IYHLLNFVLDIFSDYDYPRVFLEAHIYLVQKLKIVGFFRLMYMVTHRLDIINGCLNKFILEQERANTTVFSITKRQ
KRMKDMFNFIGHPSESNVQIRNLALMYNIIGKQCCMINELHNFKFFMILLAAFGYVVVTIWTALSYYQTQQFNATG
TVIIAIWCLSTICNLTAMAWACEALRRERRKTKISVNMIVMDYSLPKTMRVQAKAFMELIEAWPLRICIYDIFSID
ITLLLKFISVSTTYLIVLIQIYHLI
>HarmGR71
MFVTIKKKPFNILGNNHNIIEALSRTNFFRRFSGISVFTLKLSADNRVIRGFSKFGFTCFLTWLSLYIYCTYRAHA
EDQTVLRVLFSTKVQRYGDDYERISSTIYVIFAFWKIPFRLNINNGFMGMVVKVDKALEELGATVNYNFDALLALS
MSISQTFLCVTRLLSVWLTLRHLGVPVPSEKMFQVILSDSLALIATAHFSFFLTVLRCRFRHMNKILQDIKNHKSW
EHKLFIRGSMFSNPQKAVSLQDKFICEKIKACANIHAMLYQMTDLTNKVFGSILMVTILIYQTYTIWFMFSFMEAT
AAGLFHDVERYVVFCINVFWEIGYATFITFMVIYVSERAVYEVSVTHRRA
>HarmGR72
MSATMKVPVKNKYFSYPFKLDGCKNIVEALSRSNFFRRLGGISVFVLKVGSDNRVTREISTYGLLFYIVWYAIYVY
CTYKAHYEDQTILRIIYSTKLQRYGDDYERISSTAFIIFAYYKIPFGVNINKVFISMTVDLDKALENLGENVDYRL
DALLALVISISQIALSTTRFFSIWLTIYQLDVPIPLERMHQVICSDTLALIATAHYCFYVKVIKCRFQHMNKILED
IKNHKSWEYKLFTRGSMVANVQKAKGLEDKYICEKIKACGKIHSMLYKMIEANNKMFGSMIMLTVLVYISYITLYM
FYFMEATAAGLFHHPDKYAVLFVYVLWEIGYASVTIYVIVYISEYAAYEVSRK
>HarmGR73
MKFDHNVSLKQLMWLKIIFFQFCNWSSSVYVSVFAKLYCISNYLFIFIYGSIKMSHFNQYSASIRLNYITSIVELN
VDTLFSLIHGEEYVRRFVKMLEKDFPGLQKFYKYYVTIYLSLIGLVGFLIFFTMDIRGGDNILKPCLFFNRSVACY
FCRISVIYVIEGYRNTVSILRKQLNTQLNMNNITEAQKTVFIKEFTHSFIKLTEHFDSAMTIIRPLTIFRFVIDFC
KILNIIYYISFFDISSFVISWTFEVIIEMLSLCCPMLILESAADDLDEIRKIIAKELLTYEDYYLRSVIYESMEFV

DGYTTGLNVWNQYPMNKDMVLAFIGLITSYVIALLQFSY
>HarmGR74
MNITESKTICYFMKLYCICFSAVVIANFYFIEVHEKNIYSLIFVSIVEYALISSLSLIYGFNLFLNFFTAIKTNDR
IIGFERMSLITRYSYLIIFVNSVMRYVITVSFGSTYIHSLLEHFFVFLAAIALDLYYVTIIILFALIQNRMKCLRL
FLASNSVPICITARNNVANSIRNVRKSLVYYNNLLDYLEGLGQQLQYMVHFLFIYFFVLLELVKDTISYLSLFFLL
QIFVNWVCYTSRVIILLYSITVFYSTGVS
>HarmGR75
MITFQSFLSKDLTLRMLQIIAFLRLFYGNYVTISESRAICYLVKLYCIGVCSFIALFGFYAITQLYSFTIISFTLI
DYFMTALIFVVFSDQPILHFFSGVQTQDRIIGFKKMSYINKYMYFILFCTSFMRLAFFIHRIITSYRSNLEFILVV
YILTATDLNYLLLIIIFSVLHNRMNRLRLFLESNSIPINITGQNRIAISIQNVKKGLIYYDRLLDSLQSLDKILQC
QLMVYSITQFLRISMMSYRLIQQFMVEYILLRSTINVLELIPTALFVFAPLIFVEATTYEVERIKTILTAQILRSS
DECLRFELQTALQYVRLRPFRYTLCRAVPLDINLIFTTASLCITYVIVACQLVYFSK
>HarmGR76
MKPIQNFLFRDNATLSELLLRTIAYLRLFLGNYVHVTKSKGIRYFLKLYCICFFMSVMSIYVYTSVPFQFTVFSFT
VFEYILTAIVFVVSDDKHIHDYFINIITNDRIMGFKKMPYMTTYVYLILFSSTITRFCLCIIRIHTNIHTVITFCA
ILISITAMDLHYLLVIFLFSILHTRMNMLSLYIESNSVSVNIIAHNEIAKSIRAVRKSLSYYDKLLDSFENLAKIL
QYELIIYWVTEFLRASIITYNGLQGLISSKGEHLVLQHSNSVMTLNLMEVIISVALISFPAFIVEATVYEVNRIKK
TLTAQILKCSDECLRFELQTALQYVRLRPFRYTLCRAVPLDINLLFTTAALCITYVIVALQLTHFAA
>HarmGR77
MIPERQLPNQQIVSEWVLKIIAACRLLLGSYMNITESKPICYFMKLYCICFSAVVISNYFTEVYENHTFSVFYVFI
VEYICMSFLSLIYDYKLFLDFFTTIKTNDRIIGFERMSLITHYTYMIIFVNSVMRFGITVAHGSTTTKSDLGHLCI
LLTFTALDLQYLTNIIIFALLQNRMKCLRLFLASNSVPICITARNNVANSIRNVRKSLVYYNNLLDYLEGLGQQIQ
SMHYTDLFLGTLTIGVVHSKVMIYSPALIVEVTIREVEKIKAILTTQILRTSDECLRFELQTALQYVRLRPFRYTL
CRAVPLDINLLFTATAFCITYVIVALQLNHFAE
>HarmGR78p
MAIQYSVHAIYSFVTSEKQVIRLYTSVKTSDAIMGFKNLPYVSKAVLVPIHITLVLWMLFIYLFWIDSLYLPRYII
AIASDVNLLTVSLLLFGLYSRMRVIQNALENNFVPVNIVGKDQLQKNSNPMHGFTLAIINMASIIVSTSPAIISEL
ISNEIETIKATLVTQLVRCSDPSLSSELEVALHYIHIRPFKFVICRAVPVDINMPNTIISFCVTYVIVIMQFIHFS
SVY
>HarmGR79
MNPKLLFQFKSPTLCLIQIIALLRLLLGNYINLGHSKLTRFLTKLYCVCVCSSIVSMYILSLSERALFPVYSFIII
DYTCAVILSIIFGYDRYPAFLAANMTNDRIIGFKINLSLTNYAILSIYGSAFLRICLIIAHFLSHPSSILAFCSIT
CAIAATDVNYIAVIIIFTMLHRRMKNLRTFLETNSIPINITGQDEIAISITNVRKSLLYYNNLLDNFQSLDELLQY
AVSKYTIWRMFQITINWITNFSRTCVVVYFFVQHIIAQLYPSLVLISSPAIIVETIMYEVDRIKETLTTQILRCSD
PKCYTSIGISLSSVCHQTSVSGSSCRRRCSTCACVRSDTRSAARCRSTSTCCSPPPHFALLMSL
>HarmGR80
MDRASTFRSKLNVKRGTKLNPIFTKNLHSEVLLDNFIEKDLQSLLKPLNIMYSVFICAKYSIRDNFITCNNFLYNF
IGVFTTGLFLCISVYRICSTLSSKRIQYVIFEWNTIFNFMSYSLGLIMNYIINIRFSDKNIQLVVKIQSVLRVLKM
NRKDLTCLLVYNWSGVLAINALIVGSIAYMMYIFFPHVDILDVIYFYSTIVFDVNIMYSLFVANFSRKALCSWIND
VLQSGDDSDFYWNRMFNVYLNMLDIYTTLEVVFQYSVRFHYLEIFLNY
>HarmGR81
MHNLQPKKMLKMKKIIQKREKSFDQPNRIGFDYQLDKELHQIVNSFNFALNLCFSSKYYVQSNHIEARGMKYRLLT
SCYTIIMGLLCIYRIVTADIRDALMSYSENCFLRFLSGLYYTDYLLGFILCYILDIVHSHNHIILVAIFKVIHRSI
DCSKIVSRFIIWNWITLCTTICIDLFIYVMYYGFFARFSLIENISDCLCDVMFITFYINYIIAIRVIILLKVYLDE
WILNIRNLSNGLDRDEVCLKLIRVYNDIMKAYDLYKSIYQLLVSSLMNHLFQKFSVAFLRECFIFADSNSRTGHIF
AKFNVLLSDHHKPQEECTGRR
>HarmGR82
MSSITRYDVGTGNSCHSVNSRCPVNKVDKDLQSIFLPLNLLQILVLNPKFYIRKNLVKPNDCLQKLILMCGLVIFL
SGYVVYRVTEIILDDNLKRYGSINFLYYASYFDFVFYSSGFIMNVIIHCKQSSKMVTFVLIFQKIHVFLNTGSIKRS
VIRNWTNVTVIFVFYILVMLFSSLSIYNQASWNFALNLLYLASLDSNIIYAISLMRLLVDKLELWSVRVLMTSTDG
NDVTYRYQMFEAYKQILKCYDLCKDVFQQQVSQVVNLNAYSETYIDSVES
>HarmGR83
MYVRSVEDIPTLPINRLHINNMVDNDLQALLRPLNLVPRLLFCASYRIRHNFICPNSFLYNVLVVFYFVSFRCLAL
YTVIYLCIYIVDFHGGSKIFFTLFDCLDFTIFSIGFLINTYVCFKESDNNILLILKIQYVLRNLNVSRLCLKSLIA
SSWWSVILIHAFFIVSGTYYCYYFSELRITDVLTQYPTILFDVNVIYASLLIKLLEKTLRVWIEAVQKQNIDNSER
ERHLEMLFDVYFNIQGAYKIIDKTFQIQVNIYFHVLLIIKNVMTMLLIKNYESIKNIKYFVLVFISQTWLLKSIVV
FIYLSVECERFYAAMRDVHDTSIMLMNSEQFAELDMRVYKNIHRARRSLFSKLDGCRLFQVDAELPLQLSRLISSY
IIVCLQFAFL
>HarmGR84
MKFDHNVSLKQFMWLKIICFQFCNWSSSVYVSVFAKLYCISNYLFILIYGSMLMVYLGQYSASVYINYVTANTELN
VDTLFSLIHGEEYVRRFVKMLEKDFPGLQKSYKHYVTIYLTLIMFIEISIFFIMDIGERQFLQSAMFFNKHVACYF
CRISVIYIMEGYCNTVSILKKQLTTQLDTKNVTEAQKTVFIKEFTRSFMKLTEHLDSAMKIIRPLTIFRFIIDFCK
ILNIIYHVCFFDFSRFILSWCFEVIIEMFALCCPMLILEWAANDLDEIKRIIAKELLTYEDYNLRSAIYESMEFVD
GYTMGLNVWNQYPMNKDMVLAFIGLITSYVIALLQFSY
>HarmGR85p
MFSLLNFKRKQQSDPVVLTKAPNIPYSEGLLDNRVEKDLQSVLRPLNLTLGIFICSKYSIRYNFITPNGIYYNICG
FVCVVIYCSYSIYTTVSSSIELVQLKNNFNPRLHWSSIFEVFFNAFGLMLHYINNVKHRCNNTAIVLKIQFAIKVL
EFNSNELKSLKFFNWLSIIILNGICLVSIAFFSYFFFENLRFFGILSFYCSVIFDVSLLYAACVIKLLRKLLAAWN

DHVVRSKNILDSQRESYWNTMYDVYISILEAYMIVEKSFGALFTFLAIIAKAWLCKSVVLGIILSLECEKFYSAMK
KVQITVTPLIQSTRYLESQLPFCKNIHRVQLADYEKMSACGLFTVDAMMSLRLFDFITTYTIVIM
>HarmGR86
MPFYTIFNSRIAARRRSLTTANQRHEIETNEGLGRENVVEKYLQRIFQPLDIMQAIFLSTKYKIRDNIITPIGRIY
SFISISGEFGLMIVYYFLYIHTDIWTDNINHFIFGVVDFILYFCGVLLNPLVNVIQKLNNVLLVLKLQNIHRVLNM
NENYFKHIIISNWIFSFTVSNFQLLWLCGYYYAFYNIGVDNILTVYICIRFDMNVVYATRTIKLLCKSLEKWTEDL
WRSGYFEDSDDQYWDRMFEAFLDTIKAFHIIETIYQQTVGFLNSNHSDLKYGFLTFLSLSWLMKNLTLQTLLSVET
ERLYAAMREVQSSCILIPTLKQPSVYQRRFYKNIQRVQEISFKKMSMCRLVTVDAELPLRVLHTITTFTIVILQFE
FL
>HarmGR87
MIFTNNKISAKTREPEVLLDNRLGKDIQRMLYPVNLILSLFLSSKYTIKDDYITPKGKKFYIATFFFILLLYGLGI
NRVFFEDIEDTMGTDNRDIVTIIFSFAFVFYSIGFTLIFVLNIIHSDCSISLILTLQKVFNSLDFSDKIVAITRWN
WFAICIAFGTNVFLYMLYYVTYHDFNPVDLVMDIMFITFDINLVYGILVITWLRKILEKWIEDVLAFEDGDEEFYS
EYFQVYRNILNAYNCYKTLFQLLVRIFSIFCFSKNLMYYFFLPVVYFLQVLFHTADTFFRCLCYFAIILQILQMPD
STVYEQMVQYTVVKVVAAVWQIKDVLLVVMQCLECEKFYMAVEDVETTCIQRLKKKHHLAAEERLCSSVLQANRTS
YCKMSACGLFDIDATLPLDLIGLLTNYIVIMLQSFFL
>HarmGR88p
MSEKRKIVPYPSPMLPNTEIDKEMQGIVKSFNFVLHLFFSSKYCLRRNNVYPRGTKYRFMTVLHTSFLNGLNVFRA
YRDSSIGVTVNDFMRIMNCCYDFVQMYAFILLFILDFVHKQNHVLLILAIQTINRSFDLSKSIRSFIIWNWIVLFI
TLCLESYMHIVYYAIYAHSRFSDVIPDCICDFMFISFNVNYIFATRIIILLKIYLDEWVKFILILNEREENNEYCL
KLLEIYENIMQAYNLAKTVFKNLISVSHTLDLFFWVTKTMLLSLVHCAYCERFYISVEEAECACIQLIKNINCPKS
HKYLCKAVIRINRSFSKMTACGLFYIDASLAICFFGAVTNYAIVMLQFTF
>HarmGR89
MLAYFKKKPKSKVNDVIIKTLPTVQYKEVLLNNVIEKDLQSVLKPLDLMQRLFICAKYCIQDNFITSNSRSYNILG
ITLAITLRSLLFYNLLRIVSSEDQNNYVLAFSNIFDDVFFSIGFILNYYSNIIQCNNHVLLVLKIQEVHRILRVNG
KQLKCLIIINWVYVIVLNVVYILGALFYCLIVSFARILDPVSNYCSIAFDINIVYTAFVMSLLRKTLSIWIEDIRT
SKHVAIESYWTVMFDVYSNILEAFKIFEK
>HarmGR90
MTESGIPLTNKLSKEVQQIFQPLYLMQMLVLNPRYQLSNNFIHPNMWLNKFILLTSLGLYVSFSLYRIFDIYLYFD
LTMFLLGNYLQFASYFNLVFYCVGFIINFTNLFIHSKRYVAFVLIFQRIHTCIGNKASFKSAVNISRIYVSIFFGA
YFLMSCFVITIFKQTSWVIVFNTAIMLTIDANMIYAITLIHLLTNEVKLWNSHISMFSKKGSDEDQHKIMFETYLH
IAKSHRICRTVFQYMVRYFIILSRKCSNGIGLNSSVFLHVPRSYRIHLHFHGDLCLDSKSRGHTNSAGPQFRSLLP
SYRRCTRFLRSHHDMVKQSYARTYCVSTVPVSAG
>HarmGR91
MITKITQMVKQPITEPDSSFRVLKDDRLDKELQHIVRPINFALHLFFSSKFDVRYNHIYPIGTKYRLLTFCYTMIM
IIFCIYEIFTFDNLVEAAFDYQKILVKFLHIVYFTLYVVQFLTWFVLDNVQTQNIVSFILMIQVIHRGIGCSTEFR
SFVIWNWISLFSVLCVNLIIHIIYYIFLDDLSNMTTFIAFLLDILYISLDVNYAVAVSFIRLIEKYLEIWTKEVLK
MNAEKENGEQRRKLLKIYHNIMDVYDLYKTIFQYLVCLK
>HarmGR92
MLHIVRKLQTAIKKVKCASSYMNTDNLAVNVIEKYLQATLLPLNIMQELFFCAKYQIQNDFIYTNGLKYDIVSAIG
TILYVSLSFYLISSSFNKIHNLLLDLVVFAIGSILNYFVNIMQKNNNVLLICSIQNAHRMLDINGMVFKRSLIFNW
IYVIALNSFHIFWLFYYCYTFDDISIRDIFTSYLHICFDVNVVYAAKLLEINRKTAQIWIERIQQSVGNIVHCDSN
NLFKAYLEILKSYQLIEKTFQYLVSLFVACN
>HarmGR93
MTVESCVKKNSITRPDQPVKALPGDFIDNDVKRMLRPLRLIHFFSFCPYYRLKGNLILPNSLCSKLLSFCVTMFFM
FLFAYRCYDHRYIRQQRRNVAFHTINSYVLVLISCFGVLSNFISALQSKLNVKFVLKIQDVHAFLNDSKVFKRFIY
RNWIFVISIICYEVFGWISVNTLLKLSYMDVLCGAASMSFNVNIVNATRLIVLLQDKLNLWNDRVLQLEGMESNSD
TEDYCQKLYQKYIDIMECYDIHKLSFQMKVS
>HarmGR94
MLLENRLDKEVERIAFSFNLPLNLLLYSKYRLKYNRIYPNGIKYDIYALFCTLFLGVLCFYRIFTLDMTNASMSYM
ERAILTVVPILFFTIYFIGFVIVFVSDIVYKDNNVLLILTIQTIHRSISFSKTNHSFNMWTHISFATVILVNLITR
GTFYLTCRYSHVSEEISDIVRDFSFVTTDVNMVIATRIIILLKQYIDLWIKAILTTNVAQATDLYCQKLFDVYMNI
LKAYKIYRKVFQALVSFDFYIFHLFI
>HarmGR95
MLSHLNKFWKPNKSATTLEHGTRESTENEPNSFVIEDDLQSILKPLNLMLGYFFCSKYSIRDKLITYNSYIYEYIR
VLVVIIIYSWNFYNTILLNKKMLDQHWHGLICLGLGSMSGFFLSLIGDTIITWSNIIQKQCNILLVIKIQQILRVL
QIYGNELRNVINWSWICVIVLNIFSMSYFICYCVTIEEINVIGIIISFASVTYDINVVFAFLLLKLCEKILRVWLE
KIKLLKDNGDEASDEFWNRMLKVYLSLLDIYLMIESTFKHMASIS
>HarmGR96
MLTTKTMRQTQNNAEAVSCQILPKNRLEKEIQKIVCSFNFALRLFLVSNYCIKNNHINPNGKIYHTFAFFWMLFMS
VLCVYRMFTLEEAVEHLEAAILVNLLFLYYAVYWLGFTVIFIQNLVNRNRIVSLILKIQTIFRSLGFLKDIQSYII
WNYVSLASIVCANIFVHVTFYISWSHIKFIDQIIDNVTNALFVSIHIYTIIAIRVIILLRKFLEEWMSVVNTMNVE
HDNNELCLKLFESYKNILEAFNLFKEVFNLLVSNRNVYCL
>HarmGR97
MSQTQINVATVSFKILKNNRLDNEIQKIAYSFDFAVQLFLFSLYNIKNNHIDPNGKIYHTSGIIFMFLLNSLCFYR
MHSIAGLRGKIEQFDLIIFLIFFYFGWYCVVFTMMYIQNILHKNYIVELILKIQAIHRNIGYKQSLHSFVIINWIS
IATIIISNIMCLITFCASGNYNDVIELVSDNICHFTYVAHHINVIVATRIII
>HarmGR98

MYQPNSRSDISHCKNLYNRLDDEILKIVYPFRCVLYLFFTIKYRIRNNRIYPSGKLYRIFAFCWMLFLNSLCILRI
SNVEVRNNGKTKQLEYSILLVLCTGFFVTYFIEFTLMFIFDMINEENNILLILRIQNIHRSIGSRKTIQRYITWNW
ISVAIIIFSDFAIRVLYYISSYYPHFMHSVYDAIIDAMFIALDVNIVITMRILVLLRVYLNEWINNIKTMKADDEE
YRKMFQDYKNILLAYDLFKTVYQAFVGSVSTYLFNCCEY
>HarmGR99
MLKSQLVNSLNNFIDKDLQSMLLPLSLMQNFTFCPKFRIKNNRITPISYFHKFVAFVGTVMFIYFYVLRVYIQSFE
KIFKNDFFELYTCCYCSFGIAINFIDSIIQTKLYVNFVLLIQKVHRLLNDEHHFRFYVISNWLRIIAVYGFFIIIL
IEVSVWIQMPYYYIASCYPMVAFDLNLVYAISLITLLKDKIILWDIHVSNLQAMQEENEPKKMYQTYVNILKCYEI
YATCFERNVSGFFLVISLISLFLVLLPKLF
>HarmGR100
MLSSIKKTNLNKIALMVVKRPTVSSERRNKIEEDLESILKPLNIMQSLFICAKYSIRNRIITHNSKLYNALRVFCT
CIYSCIYLYRMISQDHQTINKSWRKFWFGMAWIAGFILYMVGDLINTISNIKHSHKNILLVLKIQHVLSILRISGT
DLRIFVCYSWASVIILNIFSISYVICYCFTTNIIIFEIISAYTSIAHDINIAYAIILMKLNEKMVRVFMEELESSK
CDDSKIEEYWNRMLNMYLNILEIYNIIWKTFQQMLVLLPLMAQIWNAKNMVVLMCLSTECERFYTAMKDVETACTK
IFKSRNCSVHLTRVCKNIQRGQNTYFKKMNACGFFYIDVHLPVMLSSFITSYTIVLLQFVYLD
>HarmGR101
MSPARFQKISNNRLERDMYRTAYSFNLPFIMLLASKYQFKYDRIYPNGKKYLLFNFVYMLFMNGLCIYQCYSVEMN
SDRINFLGKIAIEISINFYIVTYFIGFTMMFITDFVFKNKYVLFILKIQSIYVEIGSRSIITSFITWNWIYLLLTI
AINILLHGLFYSNYNERAFDVSIYIIRDFHLIVLDINVVLAIRIIVLLRKFIESWIQYISVKNDEADNALYCRELF
GIYVKILNAYSLYEKLFQLMKPIIAILKVLLLVKDLGLIIVHSEQCEKFYMAVTKSESVCIQLIKNGHFTKYQKRL
YKNVIRRNRVFSKMSACGLFDIDATLPIRFTEALTHYVIVLLQFNYL
>HarmGR102
MTETNDDSQTNNQLNMRPKYPKSKPCQVLPNNRLDKEVQMIANSYNIALFVFVSSKYYVKDNHIYSRERTFLLFKI
FHILFTNTLCIYRMFTININTLGSMGHYEDECMKILNVVFYVTYFICYTFIFINDVVQQNNYVVLILKIQEIHGYI
DCSEKIRSYVMFTYFGVIFTICIDSLIIVAFYMFFDTLYWFDIITTCYCDIMILSFNINYVISMRILDLLKLYLEE
WTIEVLRNVGKNDDEQCIKLLKMYQTIIEAYNLHATIFEKLVG
>HarmGR103p
MSIIKTDIFTKMIIGLQLICGFYCKISTNKVVNALVRAYCVSIAATNIVLILCELFITIYRSGIGIVVFVATLYFL
HLIIDLCSNCENFLKFVNNIRQPISQDLQSDVSTPITAIVL
>HarmGR104
MIGYHQFYRLRFFIYFRIFFGLGNFMFSPFQNFVLKLYSIVVAIIITAAGAIFIQYYGTDTFHRVVVLVEYFAYSL
ISFVAKGEDVRSFFQYLPSLDSFPGANREYRKMMNSVIFVLSFSIAWRITVTVIVLYLYSASLNTLVGMEIFFFIV
ITVAIDLGRASTFIYFSILYFRLKIFKTMLRSTDFNSTRNISVVYKFIQIYELLADKFRKIQKVLKLQVSIARQIV
>HarmGR105
MFEPQTVCNIEPLMYYRFILGFCQNFQHSRLTRILSKIYPIVLVVALIVKTFIFNDTATLYNKYFIGLEYSFNIVV
SLVTTDKYINMYFKYYHTIDSISGAKKIYKNLEKLAIISVFYASSFRSWAFIVISRYNEAFFKGFDRSNIIELAVM
YYVNDLSKITTLLCFTLLYFRTKVMKMALEATEFNDALRDRFAVNRLIQMYETIIDTFEIVAQPLKFTVRSIIMNR
YYLGVQGRFRPRWLFHRKRSASWAGHIIVHEHVRRHKCTPYIPSLS
>HarmGR106p
MRKMELFARLCVIYSVFFRLYTFHVFCQNAPPVSANSFETFDIAVLVFLNIANDMRRIMTLFCFVLLYCRTRVMKI
ALDAIDFNDSIRDRFAVNRLIQMYEALVDTLKYIGYPFKVAVCIYPESVNRQKKTVILEIFLFSGYILFDLFVIKN
NLRIDCKIITY
>HarmGR107
MNADLLKCFAPIHNVLLFLGSSRLKIKNNMIAPSTRYQKMYALCCIFIVTLSFSYIQLYYYLTYYHEDTTIYVCYA
IGIMVQNVSYLSHTIFARFLDVESSVKLCQNLQKVDNILRLKQFKRYNEQQYYWNVVVLVFIITSFECGFLVHIWY
TVEYPILAFFAGIGLLNVYMELVLAASLIVYLAIRLKFLNKIAHHNFKIKGYYNNTRAACAVDEHLLINSDVKDAN
IDLGNFLICMKEILKMYQHITQVFSFPVSRCTTRAKIIKVRASWRALSSRLLSF
>HarmGR108
MPRNIEELSEDLFTDDFIRIFQPILFVLRALGLARVSIKYRYPTGTSKWYLLYSNVFWLLNALSAVYFFFHCGESF
DSKYADSTLKFGVLNSGINGILVVFRNNLERNNKFGAMYVKLQKIERHLNMEDTKSINKQLRSQSTIVMVVAFFVT
IFWIVLFKYLFMKSMCIPLIVNVVTSIGLQTEMAQIYFIIKFIITRVNYINDMLRQVSLLSIEPLTKPMDDGILFV
VSNTLKHQDGEVPGELVSGMHCIFETLSDFTGLFQFSLFYFICQILAWNLVTIHYLVTSMKEQGAADTDLLLCVMP
VLVALQFIILTLCLKAQDLSTKLEEARKLCIDIISSPLINGKSREHAKQLTLLVEGRRSVSIYNICTFGTRLPLHL
LAITASYTVVLLQLALL
>HarmGR109
MKNLKENIKIEYLSKDILDEEFMKSFSLLYYTQRLIGSTRVQIKHRFVTTPSFLQKFHTLISVVLLLGLDYLVIQK
YDKILFDRETIYYLSICVTGLQTITFLCNIINVRFINGDANVELLVNLQQIDRRMNINRNKSITTLLVKTNVISLV
VVLIMFITLLGVASAKGTAAFWPYTGIAYSQFSFVIELISCSNMFMYFYVRARFINSIIKNYIDQKGTQEILYSKE
RFLSSYFASKVFMRRLAAGSHNFVSSDTDVYLKQLLEGFFKFQDIYKFQVFMFCCKLVASALLTFEFLLYAVQNDT
VGLWDSLTPSFFTVIDLVMAILLGVRCEVFIREVKETKRLVITVMSRHYDGRLREKSKRMLKLVEETPPHFSVYDM
WQLDANVLLQMFMLVTGLIVTQMQFAFL
>HarmGR110
MISLKSVITIKTLMAIRLGISGLYFPITSNKIISLLLKIYCAIFTITVMYYILTCTYGLPFRYNLTSYSILTMYFA
NVIHTVFHNGDGEYLKNFFVAINKIDLAIGERPDDEIKISRLIFIVVFLVMRTTGMVIYCQSEYKKYCSLLRSLLL
AGLFVSIAGQWCHTSYIMMFESVYHRMRLLRKRFENRLSASRQFEADEKVMENQLRQCLDIYKNLLGVTGLYGARI
KIMVITQLFIYVHIFLAKMYILKKNLLIDLKIELIT
>HarmGR111
MSPARFQKISNNRLERDMYRTAYSFNLPFIMLLASKYQFKYDRIYPNGKKYLLFNFVYMLFMNGLCIYQCYSVEMN

SDRINFLGKIAIEISINFYIVTYFIGFTMMFITDFVFKNKYVLFILKIQSIYVEIGSRSIITSFITWNWIYLLLTI
AINILLHGLFYSNYNERAFDVSIYIIRDFHLIVLDINVVLAIRIIVLLRKFIESWIQYISVKNDEADNALYCRELF
GIYVKILNAYSLYEKLFQLMKPIIAILKVLLLVKDLGLIIVHSEQCEKFYMAVTKSESVCIQLIKNGHFTKYQKRL
YKNVIRRNRVFSKMSACGLFDIDATLPIRFTEALTHYVIVLLQFNYL
>HarmGR112
MLFNGLPNTNVSVNRSSKVVPITVKPTQRNNYVPSKTVSAIQTIKYQVCTALVFGINRLYLFKPNTFVLLLSYVYT
IFLPILVWDIMFNADDLSATYFVFKYTCCIEYVLLISISVFTSRSKLVNLLRDLDKFDSLLNIRKDLKVIDSGYIS
VFWFCGCFIYSLCEYICCYFYLTVFIDRSVYCLYVMMLAHDCEQILFFVLLRTIYTRLRVIKAHVLKVFSAENRTN
NYRRKLDKVEALSNNAQLDISSLHRVYDLLHKCAEQLNSIMSLSVSFEELFAN
>HarmGR113
MNLFYVLIFCENFMCVYRNYFDINKYQRIVAITRIIVELTLSVVITAHNVLLTKAVNYESTEKDVLLTMLFQILTL
FKSIVIIIGGIMNSESFKQFYENLRKLYHCFENDVDYKMFEKKLRIKSLVGFSIFTFMSLVQMFGKIFQYYFLGTY
QLTEIIVLVLYELWVDMRYSLEHVVVFCAISCISDFLKCLNISVNKVLMRFSEQPSLEPNSNEENVNLPEKVNNWT
EKYQKIMACCKNISLCYQELVSNHHLPSLFMTRLGSASSLTACS
>HarmGR114
MSEIKLLTYLLHIENLLGIYRITNHHKNVKKYLITIQIFLISLLYTSVVVVEIDLNIRRVDGDDHEIDDIYLIFSS
SWYINFLTSLASSVYYRSAFESYYESINRVYDWFRREKSNVTSMTKFQWCTLMFSSFTMFFNLFQPVEALFKYDFT
YPLFVAYILLTVSFLKITLLFEHFVLFSIIILIVRVLKCLNHLVNAAEKRLRSQISDSECEIATKQIQEWASLYTD
LANC
>HarmGR115
MTPIKLLNYLLFVENILGVHRKYNIQNKIKKYLIIFQIVIQTTFHAITLISEIYLLFKEEKWKNYIDINVIKSCYA
VTAHINAISAVLTGICYSQSFLSYLDSITRVSDSFQDDRKLAKSLKKMYYLSISLMFLSISFVMYRVKEYLKRFSY
MHPLIVVPVVVSQFFIRSTLIVQPLILFTIIMIVAHLFRSFNYFISVVNKRARSVDSLSEENDITREEIQNWVELY
RDLENSCENVAIFFGHQYFFTLTMSISNSIMMVYHVGCSVLLKAITPDLRRMVIGLLNYVAWSMLPVISGQVVRNQ
AVKCHREFARLYNTVVIDPSEEKGKLIKDFIRVIKKKPLDIKLLSKLPIGMYMLPAMLTMGVNYAIMVLQINHII
>HarmGR116
MSSAKLFKYLLVFENILGIYRNYGNKNRKVKCLIIFQILVQTIYHIINVSGELYYLLQKRLSTQTFIELGFVLSAN
INASVTLVSGFLYSREFQKFHRTISLISERFKHEKSLKRSLKTLFYVTGIITGFLITSVILRAREVYVRHYSFSDA
LLVNFMFLPQLFTRLTLTYQLIITYVYVMVVVNLVKCFNSLISDGQRKVSRNTSVLVNCGCDVTKEQIQDWVELYQ
EFSNCCEDVTICHGWQASLFCICY
>HarmGR117
MADHLINLLTKTETIIGIYRNYDSLSKKQKILCKLRIFFEILFVFSIATYNVLVLENFLSTRIFHYMMMYHISNFL
GGPIVAISGILCSNTYKNFIDNFMTMDMHYQKKSAYVKCLKKMKILFVVTCIISCLSIVFFLITKITARFFIHHHY
VNIGFVLMLVVAVFVQQRFFLEHTLMYIFIRMIQNVLRCLNDCMLDAQVGYNDMTRSGQSDSREWRPLLTAEQVQL
WAEHYKCLLICSKNLSICFRSQVI
>HarmGR118p
MTKIQWCTLMFSLFTMFFNVFKPVEAIFKYGYGFARPLFMAYVLFSVSFLKITMLFEHFVLFSIIILIVRVVKCLN
QLVNAAEESLRYQTFDSESEITTKQIQEWASLYTDLTNCCKEVTQCFGGLVIFFQLLYSSVGANSHNVDSLTSLVL
APFWQA
>HarmGR119
MIEDKVIKTFVISENIMGVCRNFAALTTSQKIFSIIRIVVEISAYIIIYSLFFLDKCNHVLIEGGHFLSLMMIYHP
VNFVCGSMILLCPVYNPCGNKLFIKEFTMVQCEFRHTPFYAQSMKRVKSYIITCITFFSIIVAIVLYTKVLLVFEW
STFGPQSLYIGLITIEIIFEIRQTVESVTIFSYITLLQYHLKTINSCIASVVAQYDPLEARSDSQTNNDHLTVDRV
QYWADTYEKISNCSKLLSQCFSTQVNFGLNSILLFIY
>HarmGR120
MSLLNNLIKSENYMCIYRNFMLMKNYQKIIVLIRIFLELCATIIIIITHKIFFRDLGNVVSSLLFVPYCYQALVLLK
NLVIVVGSVLNSKSFLIVNEKTRVLHNRFRNEPSYSKSVKILNLKCSVISIAFLILVFIVIIIRIYIISFNKDRFN
KGRVVIIILFEAWVDVRFMLEHLVIYTVITLIYDFLKCLNNYVYEDLKKYNIDTKDEEVHLDEQFNETADKLTVWT
EVYQDILSCTKNTSICFNELVIKYEFI
>HarmGR121
MLTKIETILGVNRNYPSTSKTRKILFEIRIVLEVLYIIGFIYTYRTKIKFKDGQFYLMEIFHLANYLSGPIIMING
ILTSQQYKRYLENFIPVHAYYIKESKYAEKMKKIKTIFIIVTSISFAISCAGFLVKYYNRYVQNQEITFLVTIFLL
SALLVHYRFMLENGVMFTHMAMLRNLLKCLNDCILDAQVGYSYFVQSGRSDRNEWRPLLSEEKVQLWASQYMSLLN
CSKNLSVCFRAQVREPQIQ
>HarmGR122
MSSVKLIKNLLFGENMLAIYRTYDCQNRLMKCFIIIHILILILLNTFIVSLELYYLFDLGEPDQMSVVFCGFSIAS
YVNTLSSIMSGIYFSSGFLSFMKSITFISESFKNDTIVIKSEKRLRWFSLLILTFPFGLFLIRLQEVLKKFKDLNL
MILIPIVVSQTFTRMTLLYQPIVFFIVISTVVIYFKCLNRLISIATDSLKICRLRSGLHGECDLGRDQIEGWVELY
RDLANCCEKVSICFGRQFSFSLVLTMSNYILLLYQICYMNTYHIPNDFEFKKIILVIISYIIMTMLPIFAGQLICN
QELKCHRVLSRLYNTMLIYSNEAEVKLVKDFIRLMKKYPLDIKLMNKLPAGMYMVPAILSFAVNYTIVMLQFHHVI
>HarmGR123p
MTKQQWRTLMFLIFTMFFNVLKPVEAMFRYEFTHPLFVGYILLSVSFLKITMLFEHFVLFSIIILIVRVVKCLNHM
VNAAEESLRNQIFDNECEIVTKQIQEWASLYTDLTNCCKHVTQSFNCLLSFSIMLSISHFVLLTYEIIYTIYRKGI
IITDDLLQISFVILADTAVMTMPMIAGQLLLNQGVKLHRLLARLYNVLIIRPEDSEAKLIKDFLRLLKKKPLEVRF
GSNYQAGMSLLPVMLMMSVNYLVILLQFNHVV
>HarmGR124
MIKTFLLAENIMCVYRNYVSFSGYKKVLILIRITLEIIWMLITILIFVWQVSEQFSTRNHFLYLMGVYHLTNALCG
IALVICGVQYSESYKLFLNNFEVIRHEFQNSPAYTKNIKKLKRNLTILIVFFAFVSIIDFFFKLRANMITWRQNHI

KITFLTLLFTLVYIEIRKILVNSIHYANITILRSTLESLTDRVSEIREKLSKPGMSDRSYKLTVAIVDEWAVNYQK
VLISSKLLSECAGCQVICTFFLICVLFYLYLCCLKSICFYFGYCLLLFFYPVHNVV
>HarmGR125
MVLYHSSNLLCGSMIILCAICCAGSHKLFINNLKIVQREFWYTPTYIKGMKKLKIYIAVTTTFFSLAVASVLYSKC
RNATIWMTPDIILCYIALLVIEIYVEIRQILESIIIFSYISMLQYSLKCLNVDVLGTRKQYNRLGIFLYVELKSSN
FLNVQVEEWAAKYQNILVCSKLLSDCFSNQVSWYNSIKITLQTKQLADW
>HarmGR126
MLFTSKFSIAYNCIRPHRFFYYVFSFIGVLTFILYHLQRLFNGNFATYNYLSLAATLNVFFVVIPFPIFYTLNVLQ
REDNVEIILKIQNALKIINYKRYMIRTYWNWFYIIRHLIAYLIIVCITRNSQIAIYYYTLNYVDVNVIYGVIMIEL
IRDGVIIWVSEVEHYSKLCLDLNEETYNKTMKKLFHAYISLMEAFDIFKGLFQYSVSFYVISWQICFTRDHLAPVG
LHSLSEG
>HarmGR127
MSGAESSVVIRIIIAVRLLCGFYTKVSSNKIVDALVRAYCVIFSMTVFSILCTVFSRVPLIKVTFMITFSLIYACN
VILDHCFYGDKFFEFVRKMRHISISQDSRLRHVKLPITMFLTILLFCSRLFSHLKYMLENKYVIHMISMQSLGLAY
DLIQLVSVLPRILMFELIWRQMIILRKQVQQDLSSIRRFEAGEELLKNKLKVFSNAYKNLLNSTREIDSASKLLVR
NAILIEIAIKFVVIISPALLAEMVNSEIDKMKLCIVKQLLVCHGDSTRDAIEDVMLYLKQNPFKYTIWRLFTLDMT
MLYLKQNPFKYTIWRLFTLDMTLILSFIGVFTTYTVALVQFTHFFV
>HarmGR128
MSITKTDIFTKIIIAIRLICGFYYKISTNKVVNALVRAYCVSIATTVIVIIIYEWFISLDRSAVKILVFSSTLYSL
NIFVDFCSNRENFMLFVKYIRQPISQDMQLVVNTPITAILLISTLCLRVFSHIKYQYDNGIWFAFISEETLMLVLD
ILQYVSISQKMMMFELLWRKMAALRNHLERDLSSARRYETREDLLKNKLKACVDIYNNILNSTREIDAQTKFLVIN
>HarmGR129
MIQKNYVDKDLQSMLLPLNLMQNIMFYPKYSIFNNCIVPNSVLSKVVALCSTMAFVLIHLYRSYNLYYNQMIREFV
NILYITSYFDIILSCIGFAINFIVSIYQTENNVLFILKFQKVHRFLNDKNLFNRFVFMNWLILVLLFSFVIFIMSS
FFLYMEVPLPDFFCGLAAFCFDINIIYALRLIKLLEDKVKLWNFEAQHLLQIYHSNIESHCQRMFEAYFNILECYN
LYKYSFQLMVCMYGPESLFLRYFLFHHLRC
>HarmGR130
MLPSLENSLESPENVHTIESISPEASNEETVNNKIDKDLQSVLRPLNLMQAIVICSKYRISDNKIKPHSRLYNWLG
FTLVIAFRILSIHKLLTSNYPSNVSRLVAFLYMINIFDFVFNAIGFFLNSYVNIIHRYNNVWLVLKLQHIHRILNI
NSKNLQMLIIYNWISAISIYIVFIMYMCYISLFFPVFSVFGTIVTFSTISFDINVIHALLLIKLLRQTLRMWILKL
LDLKNTDTVSNDESLWTQMFDAYKNILDVYKMIEKLFKLMVSIFL
>HarmGR131
MKFYRKTETKTNVHLVFNNLLDKDVQSIFFPLNLMHYIVFCPKYTIKNNFIIPTSFIVKLISILGTLVFISVTLYR
NYYLFFYQESVTISPFMYYSSYYDALFYSFGFSMNCLFGIFKSELIIRSIMTFQNIHRYLNNESNTRRNIILNWTY
VIVTFVGYFSIYTYFYSQLSNSYNLTNAFFLVSFDINAVLAIRSLNLLEDKISLWNVSICKNQELENVNDRNYAKK
MYQAYVNVLECYETLKTLSRSFVST
>HarmGR132
MRSHTRQTFRQHGIFFPFESILNNQLDKDVQSILFPLNLLQFIVLNPKCHIKNSFINPNNSFNKVILFFGMIIYVS
AYIYRVLEITLDVNLRAYGTLSFLYIASYFDFAFYSTGFILNSIINFCKTKDMVNLILMYQDVNRFLKDKSNFRWS
VIRSWIYVALIIGFYVFTMLFMSVAPFHIVFNLIILISLDSNIIYTIILLKLLTEKVVVWNSAILKVHKNGCSTSY
CTKMFDVYVDIFSCYNLIKDMFQQPVSLLKFFSTTFLIYYYEASKIFRLCYFVFRFYTKL
>HarmGR133
MLLPLNLMQTIALYPKYSISNNVITPNSAISNVLSLCATMAATVTHFYEGFKLCYDADVVFKYIASNIQYFASFFD
IFLTCIGFIFYFFICIFHSKNNVLFVLTFQKIHRFLNDEKISKRFICWNWITMAVVFIFDIVVLTYFNLRLHLPLY
TFVCCLFAITFDVSIYYALRLMKLLSDTIKVWNMEAQNLVRLRHSNINQPNCQKMFKAYSQILECYNLFERSFQQI
VCILMFYFFVLEPSLL
>HarmGR134
MAEKHYVDKDVQSMLLPLNLMQTIALYPKYSIWNNVITPNSAISNVLSLCATMAATIVHIYEVFELCYDADVVFKY
IVSSIQYFASFFDIFLTCIGFNFYYFICIFHSKNNVSFVLKFQKVHRFLTDSKRKRFIFWNWITMASLIIFDVAVL
IYIHVKLHFPLYNLFCCLMSISFDVSMNYALRLMKLLSDKIEVWNMEAQNLRLLHHSNSDMHCQNMFKAYVQILEC
YNLFKCSFQQIVCITDVFFCIF
>HarmGR135
MESARQKTTNKTKSCSVSIATIKTIDKDMQRMLLPLNLFHNILLCPKYRIKNNFINPNSFLSIILGLFGLILSIFS
FCYRVYKYYRINPKKYVMNVMYVTSYIDFVIFSIGTIINFKINVLETVRNVSLVLKIQDIHRFLPGQNYFKCFTIW
NWISIFSGVGFYTYMLIFTVLTFEMHIDGMLFGFTLLFIDVNIIYCIRLIKLINNKVDIWNRRALKMHQMDPIDNE
DYCEKMFEAYMNILKCYDFYKDSFQLMVRNNLFHSSARYF
>HarmGR136
MCGKRYEMRNTRKRIICRKIRVHNYKNNQIDQDIKWMLFPLNLMQMITFFPKYSIRSNIIKPNSLILKFVSLTATI
LFISAFIHRHFTLLSRSNIHSSSLIYTYAATVAFCLGCIINFIYSVIKTRDQIIFVLTIQRIHRFLNNRNVYKHFV
IWNWIYVIGLFIFYISAVTYFTIMLNLPIYSTYSSVILICHDVNLICAIRLMRLLQDKAVLWNDKIWFQENENGHH
NNVCRRTFKVYVDILKSFNVYRTVFQIPVSKFMLNS
>HarmGR137
MEPKGRIKRIFRRYIDKDIQKMILPLQMMQTICLNPKFSLKNNFIKPNNIANNLLAVVGVIFFVSLLIYRICDMML
DENLRRYQTVNFLYFATCVDSFFYGCGFIMNFILHFVHTMNNVNIILIFQEIHRHINDKASSNMAVFRNYVIVSMV
FAFQTAASIYVYIVYMHPPWYVVCYVLVLISLDSNIAYSVCFMKLIADKLVLWNAKLLWSLQHGSHVMRCKKMARA
YVQILNCFDVYKNIFELPVSIR
>HarmGR138
MAINIEDISTQNNVVEKEVLSLFKPFRMMHALFVTAKYKIKDDVISANTLLYTCMSGFTSIVILVFYFFSIFQTAF

VFKWEGLNLAKQVCNIFIYAIYLMGSMMNFCSDIINKDFNVLLVYKIQSICETLKIKGKSLKNFIMINWIYVITLN
VYHMWWIVFFSYAFSSSYLYYEVVTNYFYIIFDMNVLYGMRVMKIIRQPLQIWLEEVRNLNSVIDEDYEYFWNKMF
KIYEETLETYQIFAKIFRSVVSKVNWRLLIILAYEICLKLILRNYLSCSLIRTSTIYFRCSL
>HarmGR139
MVTVEVIPVNKNKQIIEKDVQSLFKPFNIMFALFFCSKYRIRNDVIHTNSLFYKVVSGICCLAIFIGYCISVFIKI
FTIHLEGINYSKFCYNITVCALFFSGYTLIYYTHVIESNRNVLLMYKIQNIYKIVKTRGVFVKNFIKYNWIGVAVV
ALYQLLWILFFTIAFASNYEYYEVIANYVYVIFDLTGLYCVRIMRIIREPLRLWLDDVKNVQHVDHEGKASFWNKM
LRIYLETLDAYQVAARTIQPGVSLIFNY
>HarmGR140
MKLKVVAMEASIVAKNNIIDEDLQSVLKPLNFMQAVFFLSKYSIRNNLIKPNSLIYDLISVTCLLMFRIVSIYRII
IFSFASKWTPLLQFLYVSQILDSIFYSVGFLLNNYINIVYSKINIGLVLKLQFVHKVLNINRHKLRPLIIYNWIFA
ISVYSYFIIFNLYMWLKFPNPSYYALILVFSALAFDINIVYALLLIKLLTQMLRIWLVEIQELTNVGMSGSDESYW
NKMFDVYKNILEAYKTVEELFKLLVRFYFCE
>HarmGR141
MRSTSRKIWNRIRIILSSEEFSNNKLDKDVQSIFFPLNLMQLVVLGQKCRIKNNRINPNHCFNKVILFCGMVTYLM
TYMNRFLEIMLDENFRTYVKNPFLFLSTYFDHFFYMSGFILNFIISVTKTKDVVNLILTYQKVNRILKDESSFKGT
VTRSWIYVGAIFAFYLYTLFFSLLASFNVLFNTVVVITLDANLIYTMVVIKLLTEKVKLWNARIITDSENDCNNKM
FDVYVDLLKCYDLLKNVFQQSVSQATPF
>HarmGR142
MKIIIINNYKKINFVDEDIRSMLLPLNLAMLCPKYSIKGNLIAPNTFRNNCVSIVITLVLISAMCYRTYGLSFYQD
GFSNIVYYYSYYDVCYYSFGYIMNYIISVYQTEQNISLVLTLQKLHRLFNDAAAFKRFIIFNWIFVITALVTHLLL
VTSACLDMLYHSKVNLIGYLLVLFDIYIIYCFRLMKFMEDKVHLWKSKLESSEEFDVCKFCEIMFESYVDILKCYD
MIKDCFQRFVSMILFFNVSNIENSCWSA
>HarmGR143
MAVTIEEIQNIIDKDLQSLLRPLNLMYILFGCAKYKIHDNKISPNSVIYNTISSITAIFIFCISFYFMIGTFSLNF
NGYIYINHLGKIYTYILLIVGCLSDLYTNIFQKSNYISFVMNIQNIYRSLNISGIFRSYIFPNWVSVIALNCFHFT
WMFYTFYAFQSLDHSFVFASYYCIVFDMNIVYAIRIMRLINKSLKYWLEDVEMSGRFVTESYWNKMFETYIEILKT
YQIIESTFQRTVCLSV
>HarmGR144
MVKIDSKPSFEKSELSSNNVIEKDVQALLKPFNVIFALFISSKYRIQNDVIYQNTLLYKILSGISYTFLIAGYFYS
IFRTAFIYKWEGINFTKQWCNVFIYAIYFLCCVINYHTNIVCSKINVVLVFKIQNISEILKVKGISLNDFIKFNWV
YFTILNVYHVFWIVFFTIAFSDTYEYYEFLTNYVYILFDMSVLHGTRFLKVLRQPLKLWIREMRNSDSVLDEDNEY
FWNNMFRIYEEILDTYQILTKTIQPVVSY
>HarmGR145
MVLSRRNSMSLIETNQGDVEILSNNFIDKKLERLFFPLNLMQNLVLNPKYIIKQNRIKPNDVFNKFKIFLSMVIFL
AVFAYRLCEVIFDENLRRYGSVKFLYFEIYSECFVYCTRSVVNCIVNLVQSKNFVAFVLTYQEIHRILTYEHMIKF
YIIRNWVYFSIVFGYYIIVLVLIPLIFERWAFHFDINVFTYIILDANLIYTIALLKHLNDKVKQWNIEVVRSPHRI
CSERMFQVYVQIFECYEIYKNVVQENVS
>HarmGR146
MELITNNYLDRGFQNMLLPFRCLNHLVFISRFSIEYNCIRPHSRSYYIISFMGVLCYIIFHSLKFFDANLTAIPNQ
FIQFFLKVNIIMLLIPYAGFFILNVLHRNKHVQILLKMQKAFRIINYKRYKLAILWNWFGVFRHIGGFIITTAYIR
LLSVAEYFYTLIFFDVHITYAISLITLIRDGVITWIAELERHSQNLEVDKDKHDERMKKLFQAYINLMEAYEIFKK
LFQVAVRILSF
>HarmGR147
MFPCFKHEGQSDPSSEDVVHEDLQSVLKPLNLMQAPFFLSKYTIRNNCIKPNSIIYNLMAVISMLIFRIVNVYKIV
VFPFVTKVNSSVTLFLYVSQILDTVFYTVGFVLNNYLNIVYSRINIGLVLKLQFVHRVLNINRRNLKSLIIYNWIF
VVSLYSYFIFIGIFSWITYPFITLYSYILVVSALSFDMNIVYALRLIKLLTQLLRFWLMEIQELRNLGVCRSDESY
WMKMFDVFKNIVEAYKTIQDLFSLTV
>HarmGR148
MPKKNRIVEVIVRKDGIKNPLIKDIKRIFLPFNLALNLFLNSKYEIRNNNIYPNGPKYNIFASFFLILMNALCVYR
MFTFDVADNSSIEEDLTKAILGFLGTSFYFVTLIGFTITFISNTLHRENVVLLILMIQTIYRSIDISKSINSYIIR
NWICLVIVIISDFTERLMYHVTCHYHVLFEQAFDIITDIMPLVLDINIMLFNRILVLLRIYLEEWIKIVETTNDDD
EEQWVRFYKIYTNILNAFNLNAKVFEWLVRLIPYLMQNSIIFLSEYLYMS
>HarmGR149
MIDNTNSNYIDKNLQTMLFPLNLMQNMMFFPKYSIYNNNIRSNSLLSNFVSLCTTIAVISLHLYRSYKLYSDNIIR
EFINILYITSYFDIILTCIGFTINFIVSVYQSKNNVLFILKLQKVHTFLNGEQQFKRFMYRNWMFLVFEFLYFTFG
LCFFCVKLNLPKYDYFCSLTALCFDVNLVYAIRMIKLLSDKVELWNIEAQKLLQLNYVDIESHCQKMFDAYVHILE
CYDLFQCSYQQLVRIFFSLSYLFNKVFFVLKIFTIFYRSCSTAFNSFFTSSFISKLRLTF
>HarmGR150
MNTVDKDVQSMLLPLNLMQYLTFCPKYRIKDNFIIPNSRVSYFISAIASLIFMFILETFYYQILKSPDFDEEPAYL
IACTTYDTLFYGFGYTLNIMDSVIRSKKNIQFILTFQRVHRLVNIEKCFKNFVVWNWIIITLFLTLQTLLITVFCL
LSDFFDATVGYFYVLAIFDLSIVYAMRVLKLLENTTVLWIQVLNSHQFGNLYDCKKLFQAYVDILQCYDMFKSCFQ
HFVSFYQAKCFFHVMF
>HarmGR151p
MVFRLLCGFYFKISSNKIITVLVRAYCLFIGATIVLGTIVYISQLEAILISILNVTNIINVVTDCFFYGENFTAFL
QKITKLDISGDLEGQIDTPVTMFILLFTLFVRLCNHIRYLIIIRVYFSVEFCFGIGSTALCFYHTKNNDVRATVAP
DEKTP
>HarmGR152

MVLTFKTVCDKRNKKIKVKPRINMPTEAKLNNEIDKDLQSVLKPLNLIQGLFIMAKYKISDNRIQKDTLLYNLLSI
ICLIIYRLVNFYKITISSLNRDWEGTRFFIYMSNINDTIFYTFGFVLNNCINIFYSDSNILLVLKLQQVHRILKIN
SKHLNDLISFYWRFIISFFISHLLFEMYFIFQFPVYTMYGVLLSFAILTTDINIIYAYFLMKLLNKTLRVWIEEIQ
KLRNFVTFSINDSYWIEMFNAFEYILKVYNFIRKVFKLMVSYCFIIIMTETFIIIVLYSFFTFTIVEIMSFPCHF
>HarmGR153
MTPDTSVSLRNDKINHSFAIHNFVDKDVQTMLWPLNLIENILLCPKYCIKNNIIKFNSLTCILVSVIGFIICESLR
LYRIYNLHFDYFTRNFNNIKYIMAYVDFVLFSFGFFIVYFVNIFHMKYNVLFVLKFQNIHRFLNEKKYFMRYIIFY
RISVVIIPIFFTGVILYSFLRHSVTVMDCICAISLICFDTNYVYAIRMMKLLKSKVDLWNIQIGQLQKLDQDEKVI
CCNKMLEAYKNILDCLDLYKTVFQPLVSLNFFCILYHY
>HarmGR154
MKMKIICNTIKKYFSKPVMKKLIDKDLQSMLLPINLMQNILLSPQYRIKDNLIKTNTLTAILVSFCGVMISIFAFL
LRICLTSEAIKQYYSSLYIVSKIELVLYSTGFIINYISVLRSNKNVLFILKVQDIHRFVNDGIYLKRLIVCNWISV
ILIFSFDFTIIIYAWVKLELRFYNLICGVSVICFDINFIYAIIFLKLLRNQAELWNIRLKNFSGQSNGESVCRSMF
EAYDNILKCYEMYKDYFQQNVCNFNHQRVIFVY
>HarmGR155
MSVVEDGISNSNDRNVIGKDAQAWLKPWNLMDALFICSKFKIKDNVISSNSLFYNIMSITSCLVLVLIYFYCIFKD
CFHIAWEGLLLAKYVHYCFEYLVYVIGVIIAYFFNIKNRHSNVVFALKIQRICEIFKIHGKSLKSLIVLNWVLVIV
LNSYQVFWVFFFYYAFSRYGFPIEELVPNYFNIQFDVNAVHTSRIMKLMWQTLRTWLEGLQNVYIVEDEDVEHYWR
KIIAVYKEIIEAYDIFRKSFQVLVRE
>HarmGR156
MSNTLRSKVFQNHLHKDVQKMFYPFYYFFLLLLSPKYCIKDNYITPNSLKRNLVSFLGAFYVFVTSIVYACEEGYK
DYYNESNVHSLAMMSFIYSLDISSFALGIMLVFVQNIIYSRKNILIIILMFERIRQSIDISKSIRSMVIWNWVFGTL
YFSIHAIVFIELHVLSQVNFLMQIGGFICNYMYAIFDVNSVYGLRIMKILTTYLNRWTEMVLKLNEGEENLISCVK
LFDIYTNILKAFELFKDVFQVLVRSVDLIGSNYSACLFELERIIYFTGIIYNNQRVYS
>HarmGR157
MLKPCIFLRKTRSYPVEVLLNNRLDKEVESIFYPFHVILTLLCASKYCIRDHFITPNEYKFYTVNFISLSYVVASF
AYQMYNNQLVHIHRSDNNIVVSFLSVFLPISRCICHILYFVLNIMHCQNNVFIIVLINIIYKSLNSFQKVRCHIIN
SWILLALILLIHAWLTITYIVIYEIYFDVIHHVSEVLLYIFDIDFVYNVRVLLMLTNYLNSWIENIKLFDDGQEYD
KIHYIKMFQTYLNILRAYDVYKTVSQVLVRWQKSFTQIKCMSNLCFILGLPPSLTKCS
>HarmGR159
MCSVLNMYKYIRNKLRCAREAVLIDGVSPAAYKEVLFDNIIEEDLQSLLKPFNLMTALWIGKKYTIRDNFITFNSN
VYNYIGITLCVAFLIARVSYRLIVEGIDLDSFAFSLLRTNAMLDLIIFLFGILLTYSTNIIHSYHNILLVLNVQNA
LRILKINRKDLRRLTVYNWTTIACIWAFFFVPHVFYSMGTKRFDIFENLGILFKISFDVNMMYIIFLVNLLEKILH
EWIRNFEKSMFKTSDELYWKKMLYLYLNVQESYQIVETVFRTHVSLQYHYSKFLHTESV
>HarmGR160
MTPYSREVLLNNRLDKDVQRILFPFNFFLTMFLSSKYCIRDNYITPSKRKYYVFGLFGICIITAANVHQMYGQIAN
MDLNKRGLLILIFLHVTQIFNFALSIVLNIIDCHKNVLLIVIIQAIHRSFDFSKSIRNLVFYSWMILLIGLCINVY
TIAYGYAILQSWHILSFIHDVLMVVLDIDLIYKIRLLLILLTTYLNEWIKNICLKKDDWQQDQANCVNLFATYQNIL
KAYDVSNELSEIIVSYEVYL
>HarmGR161
MRSFKNIVNIYPKIINNKVDNDVQEMLYPLDFMQCLIFISKYHIRNNLIAPIGVITTFISMIATMAFVVVHIYQTL
FATSETNSIASITTENITRYFSCVFYCSVFTINFIMCVIQTNKSIKFVLTYQKVHRFLKNSGSYSYNNNYIIWNWV
FVITAVIWHTSTIVYCVVSIGYNFFVATCYMYPIMLFDINMVYAMRIIKLLENEINIWNDVIKSRLHTQDENYCRD
LLNIYVEILECYEIFKDCFQQSVSISTNRLD
>HarmGR162
MKLKICRYCSVSTKKTSLTMNSVLDKDVQSMLLPLNLMQYLTFCPKYRTKDNFIISTSRVSNVISVIGTLVLILSL
ELYYYKIVFIDNISEKQYYRFMYSLIFDSIYLSFGFILNFMDGILGSKNNINFVLRLQGVHRFLTKGNNDKKRFKY
FIICSWIIVISFVSVCFFSVPLTFIHTEFYIHIIYYLIILIFDINLIYATRIIKLLEHQVATWIQVFSCNELDYLC
EETLLKKLFRAYVDILQCYDIYKLCFQHFVSIGRKSDYIF
>HarmGR163
MFQSLNNVKHKQKIPRNILNFPPEVCLNNYLEKEIQNIFRPFNYAFILLLSSKYTMQDNYITPNGKLRTFLSCMSA
SYVSGVGFYYMISNKYLEYNSSVYSITVVTIVVQLIIIGYCFGVILIMVNNFVFSQKNILLIVTIQTIGKNINLSK
VVKNFIVGNWIAILIPSAIYAGIQGSFYIFYYIIDYTLVLATLCTIAFLAFELELVYALRVIVLLRKYLQGWVQMV
SKLNYDQDDGYDCVKLFKIYQNILQAFELYKAVSQFLVIGLHLLSSQKSFLKRTLIL
>HarmGR164
MHILFKNILDKDVQCMLWPLNLMQYMMLCPKYQIKNNLITPNSLISNIISIIATVGFISSSFYRTYEIIYYSVLKS
SFFMSFVLYYDCIYYVVGFIMNCAMGILQTKNMVKFVLIFQKIHRFLNDGSLFTRYVIMNWIYFIAALGFFFIILM
LFVMLFENWIFIIYGYELIFFDLNVVYLIRIIKLLEDKVLLYNKYLLNCQNLTHDESYRQKMVQVYVDLLECYSIL
KKSFQQFVSNAFIFCFSD
>HarmGR165
MNTVNKNVQSMLLPLNLMQYLKFCPKYCIKNNFITPNSNVSKLISAIATLALILFLELCYYKLVFHDHYDKEKHYY
LLASYTFDSIYFSFGLITNCLDGIMCSKNNIQFVLIVQRIHTFLNIKRNFNHFTAYNWLTVISYISLYFTLITIYC
IHLNLASSTIFYYMFIIFYFNLIYAIRIIKLLENQVVLWIQGLNCTQLENTYDKNCYKNLFQAYVDILQCYDIFKS
CFQHFVSILICKFSTDFNICIIIYFFGLQYLLYVTFQILYYISEVFIYSLINAEEAIILLKIGWVYLHQVRSFLES
DMKVFIYNCICRNVKSLSYFCYRPKHGSYHYS
>HarmGR166
MHNIQILKAVKNNNNIVDKDIQSMLLPLNLMHYIMCCPRYHIKNNLIIPNGLISHCVSIIGTIVFIALLCYRTYVL
SSEYTAMFDVLVYYYSYYDIVYYTFGLTMGCTLSIIQTKKNVEFVLIFQKVHRFLNDETSFKNLIVFNWIFFVAAI

VCHFFIVSGFFSLLTYYSKFVWTAYLLVFLDFYIINVIRAIKLIEDKARLWSLNLLNKNIENMDVQNYCKRMFESY
FNILKCYDIIKVCFQQFVSMIITWLYDNLIVAEL
>HarmGR167
MFNSHVAVTSCYPVEVLLNNHLDKEVQAIFYPFNFLLTISMSSKYCIRDNYITPTQRKFHILRFVCTVALLVIYDS
VTFKVFAGENIIFYIARFLSVIRNLTFLQNVAVSILCNQDNVLLVVLIQMIHRSIDLSNKIRSFIAWNWIIIATIS
AFNIMVATAFSSGKDGFNFIGCFTDVIYTSFDVDFVYSIRVLNLLNKYLYEWIKSVRIMNEGKENDKMNCTKLLKT
YENILRAYDVYKKVTQYLVSILRKGYIILCFSITKQKTM
>HarmGR168
MHNIFKIFQKKRNQTIILNPVHHDTRLDNEVLRIIYPFNFAFFLLLSSKYSIQDDRIMPIGMMRKCLSLFNVFYAG
ALSLFFIYFYIVTNDFSKSSIIMSIIHVSGTVTFSMGLTLIIVVNIVFENYNIQLIKMIQFINRGIDFSRSVKSFI
IYNWVFVIFVFSIDLFTYIFFMVTYYMDVLGIVTLWARLMFISYDINRVYAIRIITLLRKYLDEWNKNVSQVNNED
GTRFMKLLEVYENILESFKLYKTIFQELVSIFNELNVYLL
>HarmGR169
MRSNCPPAVLLNNHLDEEVQRILQPFHTLLTVFLSSKYRIRDNHINPNGFIFEFSGFSGLCFAFGATVYRLLKNEN
SGFDSNVMTNIIHYFLPAMRLLGFVTNFVLTIIHRYNNVILVLHIQRIYRSIDFSKSVDSYVLGNRITVAIILVTN
GVIFTVFITMYNGFDVLNVLFDIYFVTLDVDFIYAIRILILLVRFLEEWIKSNKLIEEQAIDGEYSSKLRESHRYI
LLAYEMYKTTTQLMVSQEGPQKVDR
>HarmGR170
MCSYFKEYFESCQELIIIKDISPEADFGNNIVDEDLQSILRPLNLMQSLFLSAKYCIRDKNITSTTRFYTFLRIIF
VLVHRCFQAYQIIVWNTNMFNENSTSPNYYSMLYLCIASTVGFFIYFIGDVISIISNIVFRRSNILLVLKIQHVFS
FLRLNRNEIRGFIVSSWVLVIVSNVMSLSFVVYYVFTFAEVHFITMFTAYASISFEINVLYAFILLKLTENMLREW
IKKFEATRNIDDSEKLFQVFGIYWDTLEIYMIIEKTFQHMVWFDSTC
>HarmGR171
MKHNIIRYTLCITDMYPSLTMYLKQFRNFAKVENISAVTSEDRFQNNKVEEDLQSIFKPFNFMMNLFLCAKYSVRG
KCFTPNTRFYNWFRLICVIVNRCFNLRQFIIWHYTIKKTHFVFSYYYGHLCINIGSAIIYILYLTGDLIISVSTIT
QSDYNIFLVIKMQEVLKSLKINGSEIKGFLSFSWWSVIISNILSIGYIILYCFTLADMVSIVDVISAYASISYEIH
VLYALLLLKLTNKMLTVWIKEFRNSRNLGDSTNEEYINRMFFIYWDVQDIYMTIEKTFHHTVRNSIYCFIWIVAVV
QYIYIFQILFYIIFTISISLWEIFSSLVFKTAGHQSVSIKVTWLILSGIL
>HarmGR172
MTRRTRIQQNNNNIFQVEMLLNNYVDEDIQSMLLPLNMLHFIRLCPKFIIRDNFITPNSSKFNSVFFIATIVWLFA
LFYDIHTEFWEELVNFNITDSVSFSVFFSSGLIVNLSLSVFRTRDYVSFVLKFQNVHRFLNNESDFKVFTFINWFI
VILFVIIYGGFVIILILITDTLTIQLFCAFVLLSFDADIIYIIRFMKLLKDKFNLWNEQALQVRNMRDGNKEEYCQ
KLYQAYIDIMECYRLIKTFSRLVVSKKQASLPVSLESFLVNYFIAKTSCYCSISSTSRNYYPQ
>HarmGR173
MWSCFKQYWLPHHRSVIIENTSSFSPDDIIEYNRIEEDLQAILRPLNFMENIFLCAKYSIRRQYITSNSRIYNFFR
VFCIILNRCFHTNQIIDWNITVWNERNTTLHFYSTLCVSISGFIGYMLYLTGDSVSTVSNIVLSRYNILLALKIQC
ALSSLRINRSQVTGIIICSWCCVIISNIYSISWVIFYCCYYGNINIVTIVTAYASISYEISVAYAFILIKLTDKLL
QQWINECRALSLEDSENVENVDKLFIVYCDIQEIYMIIEKTFQHLVSFEPS
>HarmGR174
MFSYLKEYFKLYKMSTVENISRVPSDNICENNRIEEDLQLILRPLNFMQGLFFCAKYSIRGKSITCTTRGYHLLRV
ICVIVAHGYNAYLFIVNSIAFWNNPIASSFFYSSLCLWVSSFIAYILYMIGDSLNSIVNISMSHLNIVLVLKIQHV
LSFLRLRRSDVKGSIICSWACVIIANILSFGWIAYFCATAPEINYIPIITSYASITYEINVIYAFTLLNLTTKMLN
VCINEFRTSSCLKASENVKYLHELFHTYCNILEIYMIIEKTFQHMVSI
>HarmGR175
MWDSVRNAQIFIPSSQPVALSKNRLNKEVYRIIYPFHLIFLVLCSSKYTVKYNFILKDGVLRKIVSFLSVCFVVIV
SSCYMFLEKYTAYINNVQSIKIIFFIFHGVLILYCIANIMLFSMNIALSKQNIELILKIQLVHTNIDFSKSSKNFI
IWNWIYLIIFLVIDLAISSSYYVTYYEQDVVDALGYLCNYLFAVFDMNVIYACRLITLLRKYLNKWSKVILKLSDG
VNNRNCGQLFEIYDNIIRAFQLYKTVFQVVVSSLLTIAVAIDSFVHTFKQISCWCHVPLQILSTTVNIFSRNLGFI
ESRLQIITDTAESVNEVIFVLLSLKFLLSLFYL
>HarmGR176
MTTVRKIVSIKPIYNFTIDKDIQSMLLPLNLTQYMMFCHKYRIKNNLITPNGLRTKCITIIGTIIFIFSIAYRTFS
LSFNQNSAAFSPLIYYYSYYDTIYYGFGLILSCVLSIRNTKKHVRFILIFQKVHRFLNDKTVFKQSVVFNWLFVIT
CLVIHFTTVISVALMLIYYIKYVWNGFVLVVFDLNVVHTVRFIKLLEDKVEVWRTRLLNSPDLEITDLPSYSKGMF
QAFFFFF
>HarmGR177
METLPLSNAPKSSFSSNVIDKDVQAIFYPLNVMSVLLLHPKYVIKNNKITPLSNVIKIFSACVTTLYLCQHAHKFF
SVVLDDNIRRIQPVSYLYYATGSDLLFLTWGFIMNCAGNIIYTKKYVAFILKFQESHRFLGSACFKRFIIVNWLSI
ISMFGYFISSCIYTYFTFFHPPWGTIFHMMVLANLDADAVYACRLILLISEQFIQWNERALLLKENGEDKDYCRKM
FETYGQILKCYKIYRNTMQFMVSRILGFLMIISCL
>HarmGR178
MSIKMKPTAHPKEFTCTYIDRDVQKMYQPLNLMQQLTLNPKYQIKAGFIKPNNVKSILLSFCGLISYVGVSIYRVW
ELATDENMQRYTPIKFLNFATLIDASFNSTGYIMNFILPIIEVKQNITFILTFQEIHRFIHKTDNCKIIVNNWISV
ITYFSFYIFACIYIYVYYMPNWYVMYYVFILCTRDTNPIYAIGVIKLLTEKVFLWNSELLMSSKAGHREMRCDRMF
RAYGHILSCYDVYKNIFQIPVSIIFGKLRNYNCVYIKSSFLFLN
>HarmGR180
MQVSDIIKPGDFNNKSYNSMIPIIRILKVFAINGNVAPNKTSMLIKSICACSIFGCLSSYCLYYKTKYVYNRLDIS
IRVTDMTQMICDFFQYTVDLFFVYKFGRSLYIEYFRQFEIIDVCLETSCYAEMKRRLLKTMTFFLVIWFISSFTDL
GAWVITYGWMIPVVHSLSYLYLLIKILATLDLIANIIQVEVRLRIINNFIKNCYNCASACPVGILADCIRNKNWLH

GEDGSPDQSLKARSIDSHEIKRLSKCYLMLTEQVMFINKMYGFRILLNTTSLLFDMVKILNLAIRIIVGSQRTLYN
SAGYNFLPGVSGFVRFLTCAAILITLVNRCEQAYRQRERILNVIDHLLINKNPDLTLRSAIQDLQSLLQDRPICFN
MAGFFTLNFSLLVSIASVVVTYTIILLQSVN
>HarmGR181
MAQTVMNFVVGVSQTHKSIRFVLTFQKVHRFLKNDPHFDNLIIWNWIVVIIAATFYNSAFVYFTNYLGLPMYFIYV
SVILSAFDFNIVYAYRLMTCLTHKLEVWNIKVLSSGETNCDIFSKNMFQAYLDILECYDLVEACFQHYFFFVFQIL
FYIGEVFIHYLDLMSIAGMAIFSTVIWLMKNLAWQIMLSQQCEKFYSTVQSAQDNCMFVLKSNCTESVQRLCKNVR
RLHRSRFSKLRVCALFRADAALQLSLMALLTDYIVVVLQFAFL
>HarmGR182
MSLMCTVATALRSSLNTKKALDIGRLQNINVMPFPSNVVDKDVQSILLPLNLLQLLTFCPKYRIKNNVVYPNSLIA
ISIIVIATSIFVLSFVYRSYYLLSANVLPWFSDFSVYFDVIFYSIGFIMNCFFIIVQSKQNVQFVLIFQSLHSFLK
ETNLKNLVIWNWVFVILVLLVFHIIFIYLLVILKLPFHFLYYSILLNSLDFHIVYASRLMKLLEHKLILWNIQVLS
CQGNIDESYGKRLFQAYIDMLQCYELVKVFFQQFVKVLVHSLFYIKASLDLFSMTVKLGQMHRIAMAALSISSIVI
WLVKNLLWQIQLTVQCERFYSVILHSQDTCAIVLNSNGSEAEKRLCKNVRRVTRARFSKLRVCALFYVDASLQLSL
MALLTDYIVVLLQFAFLDP
>HarmGR183
MSINLVDKDVQSMLLPLNLMQYIAFCPKYCIKNNFISINSLFSNFISFCGLLIFLSSFLYRNTLIAQSLGHSFTVF
MYITAYFDFVYYCCGYVMNFIVGIMQTKNSVNFVLTFQNIHRFLNNETYSRNFKVLNWIIVILTVVGQTTIFAYFN
ITVGLSHYFIYISFIITVFDFNIIYATRLLGILENKLLLWSNNVLDLREIGEIYDENYCRNMYLAYVDILKCYELH
KVCFQEYICFYITETFLHSLICIQVSIEMCKMAASRGNISTIGTAILSTMSVLLWILKDLFWQLLFCRQCEKFYSS
MENVPDYCTLILKTSCSESVRRLCKNVRRVHRARFSKLRVCSLFDAGAALQLSLMVLLADYTIVLLQFAFL
>HarmGR184
MEIFVNSRQINSNNIVDSDVQSMLLPLNLLQNVFFCPKYRIKNNYITPTNLMSNLISSIATLVFIIMYAYRNYLIG
LFKTSQFSTAWKYSSYFNGFCYSLGFIMNLVIGIIQSQNSVQFVLTFQNVHRFLKNENGFRSFIIWNWVAVCLTLV
YYVFFFIYQYTRGTIGKIHACVGFLLSSFDFNVVYATRLLRLLEYQLVLWNNRFFKLRETSDIRDKDIIQKLFNAY
ANILECYDIIKISFQHYVSFTLQLIIDLLKYAANNDCQKTVIIAIRVSIAVLLWLIKNLMWQMTFSHQCERLYLAN
ERTLDHCAFILTSYCSGMEKRLCKNVLRMSRVRFSKLRVCGLFYAGAALQLSLIALLADYTIVLLQLAFL
>HarmGR186
MWKKSIHAFKILPNNRLEKEVQRIVTPFNVILTAVCSPKFRIRNGYITPSSKKIHILLFFGITACNVWSCYGITKS
QNEYTTTSIVSYFFSLTIFFYYIDFILFTSCNVLHSRRQVSLILKIQELYRNIDITKKVKNYIIWTWIWFLATFSV
FLVNFLSFLMNLDRIFFIHLSTYFANLQFDLNFIYSVRIMTLLVIYLKEWTKSVVDLNEEEQNKEYFVKKFKTYQN
ILRAFKIFTLCFKDIVSTSSYI
>HarmGR187
MADSSRRLFELVFRNKLDEDTLMIIKPFNIFLRIFFSSKFKIRNGYITPRDKTYYILPFIFVSLFKVWTVYYVYIY
NSSILNNTFRHIYFWHIFISYCIYYSLLVYCNIVNSQNNVVLILRIQEIFRSIHLKNGIRSYVIWNWITFVVLASL
ECFCTTIYARTMNLLSSLNSFDILLSICYDFNVACSIRLIKSLTLNLVEWSNTDK
>HarmGR188
MWYSHRPSQLLLFTNKLDKDVQRILKPFNIILTIFFSSKFKIRNGYITPCDKKLHIILFICVIFLNAWSVYEMRVY
ISGKASIIINSQIIFSFLLIICFFTYFILIFSNIAYCQSNILLIYTIQDIHRAMRNSSSFKNYITWNWITILICIC
FDILIMSSYCMLLSKIHIFSVSTLYLNMASEINVFYCIRVLAFLIMSLEEWIENVLVVKSDDYCEEYCGKLFKVYQ
DIIKAHKLLQNCFRLLVRSLIFILIKIF
>HarmGR189
MFQSKRSVILLTTNKLDEDILRIVRPFNVVLTAIASSKFKIKNRHITPCVKTFHLLICFSIIALKLWSSFMFVVGR
GDFKHKKIVDNFFCLTVFFYCVNYTLLTYNNIMHSCHNISLFMKIQDINRNINISVQSYVVWTWISFLITLILFIS
DISWFLLKSGIVGTVHVSENILVFQFDINFVYGIRLVTLLVMYLKEWARSIIIMTEERPNEYFVRKLETYVNILEA
FKLFTICFKAMVSWFYKLDEMLS
>HarmGR190
MKVTVVHPFVLSPSNKLDKDILRIVKPFNIILTAVCSSKYKIRHGYITPCGLNYYILTYAGIGVFLVWSTYNMISI
NIDELNQRADLAAYLYCTMFLFYISYGQFIIYNIIHRQDHISLILKIQEIYRSVDISKCVQNVVLWNWLSFFLMFC
CMIPNISLLYLSQNFVSYTHITNHISNIMFDFNFIYGIRIIALLVVFLKKWSESILDKEFNLEKKLKAYQNILEAF
QLFAICFRAIVSSS
>HarmGR191
MEGKTREKKFSCNYVDKDVRTIFRPLKIMQTCSFNPKYQFKNNFIYPANTISDVIAYFGVITFLTIMIWRITDILF
DENLRRYQTLNFLYFASYADSLFYSFGYIMNLILHFLHSKDSVKMILIFQEIHRFIRKSASLKNIVCRNWVALALM
GGFQSIVVIYLYIVYMHPPWYIIFYVFYLLSPDYNTVYAICFMKLLLDKAVLWNVNLLLSLQGQRKVLCRQMTRTY
GQILDCFDVYKNVFELPVSTYKIFIC
>HarmGR192
MNMNQEFAPVFTVDKDVQTMLMPLNLMQNVFFNKKYRIKNNTILPNNLTSNILSFVASLTCTLIFLYRVYLMSALE
DSRFTSILYYSSFFDCLYYCVGYVINFVSGVIFTKKSIEFVFIFQKIHRFISNETDFRLFVIWNWVSVSVAASGYV
LICIFFVTNADLSVFNGFPCLFLCIFDFNIVYAMRLIQLLRSKLVLWNLSLETTSI
>HarmGR194p
MSIIKTDIFTKMIIGLQLICGFYCKISTNKVVNALVRAYCVSIAATNIVLILCELFITIYRSGIGIVVFVATLYFL
HLIIDLCSNCENFLKFVNNIRQPISQDLQSDVSTPITAIVL
>HarmGR195
MEIKSSSFFKSFQKCMGPLYFYKVLILLQVLLGRYFSLSKSKLTRFFTKLYCVFMYIHMIYKWNDVVLVSHKFVLP
PFIMSEYTGYFVISIILSEDYFFNFCDNLLTNDRVMGFKNIPHVPPNVIGFMLITVISRVAFVLTRHFTVSLPSVH
LIYVTVLLISLDLSHIYTCVIFCMIQLRMKVLRCFLENIHIPINIVSGNEVEMSIKNVRKSLYYYNNLLDSMAAID
KHTQCMVSKLYLHQ

>HarmGR196
MFSYLKEYFKLYKMSTVENISRVPSDNICENNRIEEDLQLILRPLNFMQGLFFCAKYSIRGKSITCTTRGYHLLRV
ICVIVAHGYNAYLFIVNSIAFWNNPIASSFFYSSLCLWVSSFIAYILYMIGDSLNSIVNISMSHLNIVLVLKIQHV
LSFLRLRRSDVKGSIICSWACVIIANILSFGWIAYFCATAPEINYIPIITSYASITYEINVIYAFTLLNLTTKMLN
VCINEFRTSSCLKASENVKYLHELFHTYCNILEIYMIIEKTFQHMVSI
>HarmGR197
MDNSVYKLLSLRLLFGHYFKLSSSKWICYIAKIFCFSMLIVNFAINCILLLDFTSFDISQISLWMLLWIMLVESSS
SILISLYTDETYLLKFSAKIKNYVSSPTPCRATYVMASFIIPLNFSSVLIAYLYEFGVAVNIFYNISYTTCYCSYL
TSLYITEMYAKAINNLTSAIVNRLKDINISDEEKRVCIENFLDNYLKLLKIYNATMTVSRINVSVCKSFE
>BmorGR1
MNRHDHRFSIYNPKRNEAMWKRELFVNNEGKDIKDFQIKDIYGPEITDKDGALLDKHDSFYLNTKSLLVLFQIMGV
MPIMRVPKSAQTTRRTTYNWISKATLWAYLVWGLECIIVVKVGQERLANFQIGSNKRFDEVIYNIIFLSILIPHFL
LPIASWRHGPQVAIFKNMWTHYQLKYLKITGKPIVFPNLYILTWGLCIFSWVLSFAVVLSQHYLQDDFELWHSFAY
YHIIAMLDGFCSLWYINCNAFGTASRGLAINLHKALEAEHPALKLAQYRHLWVDLSHMMQQLGRAYSNMYGIYCMV
IFFTTTISLYGALSEILEHGLSYKEMGLFVIVAYCMTLLFIICNEAYHASRKVGHEFQDRLLNVNLGAIDRSTQRE
VEMFLVAIAKNPPIMNLDGFTNINRELFTANISFMSTYLIVLMQFKLTLLRQGARKTVTAIVRAIFNTTITDNG
AGGSDEDQE
>BmorGR2
MIPDHLFEEGINNTFLDYDMRHVQRNRNIQEKTQKDYEQEQRDLLSSQDGDTCEIHDQFYRDHKLLLVLFRALAVM
PITRSRPGTITFSWKSTATIYAVCFYIAATAVVLIVGYERIQILQSIKRFDDYIYAILFIVFLVPHFWIPFVGWGV
AHQVAIYKTNWGKFQVRYYRVTGENLKFPNLKTLIVIISVGCLLLAVCFLLSLCALLDGFLLKHTSAYYHIITMIN
MNCALWYINCKAIKIASQSLSECFQRVAAGVQDTLLSIDVLAVDRPTQKEIDHFIQAIEMNPAFVSLKGYAHVNRE
LLTSVRFTTIIEADLLMIY
>BmorGR3
MSFEIKNNFFRTSVPIPNGFPVQTEAKSKNKPIFLDVSPAPTPKVNSPNAIIPMKNNLIDPFINKDIIYENIKPVF
MVLRIMGVLPLTRTTSGVTYKQLSNRILPVKLYKKSLLIAIIIPILSTTSVIVTHVTMVHFKTSQIIPYVFLEILT
YMLGGYWYLLCEILSLCANVLADDFQQALRHVGPAGKVAKYRALWLRLSKLARNTGVANCYTFTFVNLYLFLIITL
SIYGLLSKISEGFGTKDIGLALTALCSVFLLFFICDEAHYASHNVRTNFQKKLLMVELSWMNTDAQTEVNMFLRAT
EMNPSQISLGGFFDVNRTLFKSLLATMVTYLVVLLQFQISIPDATQPEIPTNIDDHVQNITDTTTEASSPISTLMS
AFAKRKND
>BmorGR4
MSRIFSMTRYFGVSTCKPSIAFGWTVILLLMLLAIEVGAIWKIVRLLGGWAVHSTDSRGFTARLSGCIFYGNALLS
LILSIKFVSSWEQLSERWSRTETDPGLRLPSDSRIKRRTVLVSAFVMTCACVEHMLSMMSATGFDCPPEEYTERYI
LSSHGFLVQNDEYNLWLAIPIFIMSKLATALWNFQDLIIILISMGFTSRYNRLNTYVHRVVMLERNLKEGAQVSSE
NYMRFQIWRRIRQAYVRQAALVRLVDDQLGALVLLSNVNNLYFICLQLFLGINSKDRGSFINRLYYFISLGWLMFR
ACGVVLAAADVYIHSKKALISLYLCPELAYNLEIKRLKYQLKNDEVALTGMGLFSLNRELLLEVAGTIVTYELVLL
QFSNED
>BmorGR5
MYACYKIIVALSLNRQYNHTVTRVGNRKHIKSTTRGNFRERILRKVQNRISPEPIQEDSKIPLTCQFQLFQTAMKH
LLISGQFMGLNPVSRISDHSPTKIRFTVLSWKFVYGVTIGIAQACATVLCFCKLLKDSVNIVALDFVKIVSAYFAF
YLSTGCNTFIFLRVASKWPTLIKHVYETQLDSYIDVKVKNKCFAAYIIFFSMSMTEHMLSLLSKFVITMDCLPKGS
DLFESYIIRNFPWLFEFDVPYYLPIGVILQFLTLVSTINWSYSDLFIVCMSIYLTSILKQINKKIEMAGNSNHLPI
PFWRTLREDYTRATRLVRSFDDTISSVIFLSFASNLFFICLQLYNILSNGVTSKYNLLKEMCPNYPSGPLGGYEQI
MYLLFSLSFLLGRSLVVSLVAAKVHSASMVPASALYNIPRNMYCSEIQRFLDQVHGDKVALSGLRFFYVTRSLVLS
VAGTIVTYELVLLQFSNED
>BmorGR6
MLLRNYKQNLSFWTSAKKSKIHKIQSQETVTFQGSLKLVLFIGQLFSLFPVCGLLSNDANKVKFVPISWKCGYSML
SMIGQLFIIVMCILYVAHFETTLNGTTPIIFYGVTFISMIAFIRASRRWPELIQHISKSEELDPSFDFRLKKKCNI
TLLLVLVLAILEHIFSIRSAYSASQICYPHTGFYEGFVRYLYPWVFDFLPYSAALGMVTQFLNIQSHFIWNFTDLF
VICMSYYLTSRLDLVNKKLLPAQGKYLPEIFWRTTRETYCRATKLVRKVDEIINGILFISFANNLFFVCVQLFNTF
DDSVDMVGLCYNYSERRTKPVGREPVIYLLFSLGFLISRSITVSLIASQVNLASTVPAPILYDVPSAVYCVEVQRF
LEQVNGDNVALTGLQFFSVTRGLLLSVAGTIVTYELVMVQFNQAPASDSFTEKLVENNISTIETFYNYS
>BmorGR7
MVLEAHTQIQYCTAKANYCEFHAGLRHLMRLARWAGFFPVQGLSQTNPDDVRFEFRSLYALYHAITVIGQTVMTFL
AFYSFVDSNVSLSVVSNFLFYFTNYVTLVLLWRLSKNWSALISKTLEFEQSVTEIRTTRNLVSRTNTLTYVVLIFA
MIEHALSKVFNIRSVMCCLGETSLNHTVINNYFKFKWKFVFDYFSTSTTYSYFVGFIAEFLCMQATFLWSFTDVLI
MCFSIYLSSFFEDFNSTVSSFMKKASKTVPWSTLRVQYSQIVLIVKQMDEQLDYFVLISYFTNLFFICFQLYNSLN
RIYDANDVCNENMDIIATASVTYLTYYVFSFLFLVTRALLLSIMAANVHSCAQVPQLALYEVPTADYSLDVQRFQL
QLRYTTVGLSGVCFNVTRGMILRVIGTIVTYELVLIQLTKKNLDNDTSIRDYYLPKHLI
>BmorGR8
MAPRSVRSMVGTSKKDMLKGGFYETVRIPLYIYRLIGILPISGLWHRSSKYNRFSLKSFYTIIYAPTIVMQTFLLL
VHIYDLFAFFFGHQRLGRLIYHMNFYTITILIFMGSRKWKNVIKEIETIELTLPRLRNSKKALALTKSFVFAFFVF
SLAEVVLILQFTLRLTKQRHVLPGDSGLYLRSYFVYIFPYLYDHFPFSYVMGFIVQIIKVQGIITLNMVNCSVVIL
SIYLTNRLKHYNRIVFAKGSKTNNTRLKWVELNLLYTRISNLVKIIDKNLNPFVFISFTANLSYICAQLFYILNKL
TSSRTVKITSFLEDKRSDWETVLYISISFALVVLKVLLVSITAAEVHTTSREPLRLLYTLPTAEYTIETQRLMTQV
YYSNLSLSGLNFFHITRGMLLGMVATLLTYEIVLLQI
>BmorGR9
MPPSPDLRADEPKTPCLVGGAHAFILKISSFCGLAPLRFEPRSQEYAVTISKGKCFYSYILVTFLVICTIYGLVAE

IGVGVEKSVRMSSRMSQVVSACDILVVAVTAGVGVYGAPARMRTMLSYMENIVAVDRELGRHHSAATERKLCALLL
LILLSFTILLVDDFCFYAMQAGKTGRQWEIVTNYAGFYFLWYIVMVLELQFAFTALSLRARLKLFNEALNVTASQV
CKPVKKPKNSQLSVYATSVRPVSCKRENVIVETIRVRDKDDAFVMMKTADGVPCLQVPPCEAVGRLSRMRCTLCEV
TRHIADGYGLPLVIILMSTLLHLIVTPYFLIMEIIVSTHRLHFLVLQFLWCTTHLIRMLVVVEPCHYTIREGKRTE
DILCRLMTLAPHGGVLSSRLEVLSRLLMLQNISYSPLGMCTLDRPLMVTVLGAVTTYLVILIQFQRYDS
>BmorGR10
MTMSIKPRLQCMVPPSLALALRVSRLAGIAPLKFVAKQSNIMIRLSTSLCVYSYLLVTALNVCTLIAVMIDFSVPV
KLSIRMQTETKRFVWIADVVIMGILSGVGVYTAPIQMRRLIAYLHRIHKINSDLGTYSSSLTDKMLHRLTIGMLLI
TSVIIVTDFTFVMYLADLNHRQLLIAIMYWCYYCSYFIAHLLEMQFVLIAALALSSLKLVNNGLRTLLHQSGIESL
TEIPNSNEQHTANAILPQPPKKSVNNSIDTLAFVVTKRSVRFPTAGWMDQRTIRRLALSYGSICEVVRQIDNNNGI
IVLLLLASFLLHLVVTPYYLIISFVTESPHTGFEKVLNPILQTVWCLYHTFGLVMIIEPCHRTHEEMETTRELVSR
VMCSADPRDPISIELEMFFRQLVLNKASYAPLKVCTLTRSLVATILGSITTYLIVIVQLEIKNMQ
>BmorGR11
MKPFRFFLFVENVICVYRNYSFHKRYARAIILSRVMFEVSLIILTLHSCRNFGAVKYKTEIIFTYLATASSTILIL
LALYKTNRFTELFLNFKAFYRNRNLDVDHLEKWNRKQKMATVIIVLFCVIKFSTLIYTDLIGEYSTPCRGYFTEYL
FYTNLFMCNARYLFEFSTACVVLHLVSEQLDYIAISMDCTMFLYIDISKKNIMSSAKKRKLKYFDIFKQFEKWTDA
YMNVKRSANLCDTVFRAQLAIMITTITLYYIILLYGITSFNIERGKFSVVKSLSYLISLFGFLIALLLLSKAGQRI
QKSAENLRRKLSKFLLHSLEDPEFHRAATNLLRLVCTHHIKMRCFGFIDIDMTLLPSCLMFVTSYT
VIALQFNNVV
>BmorGR12
MKNLKLCRTTFYFKIIMCSRFISGLYFKATSKKWISYLYKVICVLYIICITRLFYAKEDTFKPLVFXXQFIGNSIE
SLRTGEGHVLKCYSTIFSLKLIRNYLPDSNNHIPISSITNFLVIIWKVFDQVYIVLMHYFYTTDIIIHLRILSILT
TIGVNLSLMPIIVIFELMWRAVKALRKSLGEHLKGPVLIEGRERLKAQQILRCLNVYKDLNATLKFNSTPMKTMIL
ISTLATFIRLTLFLYQAILGHNEGLHLPRKILAIIYYALPVCLLGVLMELVARECDKLKTLMTKELLVCKDDSYCT
VIVDAVSYIELNPLKFSILRAFNVNSTLILGLTNLCTTYLIAVIQFTYSCEDINGLSHSHSH
>BmorGR13
MEDSFNRLLSIRNMIIFQNVCGFYHMCTEKLYISRIIKMYCVALAIVLSVFCFQNPDITYLSWDVVWVTFGYTLNV
IICLRYNGNYFFQYWNGLHEIDIKMNLTSIDKEKVPISRAVFTVFLILRSTAFAMTIFVFGYLETGILSNTIISIY
SINLTEFYRNMSNIPMILMFETFYVRIKILKEQLCSELSTVLGCNNDARQLKLILKYLRNYRSLVRHLMDTTLPFK
ILILVILVGSFLRSLLIGYAFVYNSDQIILLSLPVMFSTKILSEVVEIKLICTKELLKNKNEGLVLLDLDSKKPTF
LTSKACGEQLQDALSFLNNRSYSYTLLQVIEFDCSLAFVFTSFCITHLIVVVQFTHVLD
>BmorGR14
MNLHKNIIPIRNNLFANKVTAIALPKTLSVLFKLIHIFFLLDLGVYEYKTFKIKCIVKFLTISGSLTISVVCFSFM
VSNLSEHTFVGWYGFFISTYIFVVLFFNLSNRMTFVEFYKTLLRFDANYGIDSNEYKFNFKIIFVNILFIANRMVL
SFVYCSYYPQNCIRPRYAQILFMLPWLTLDVLLTTNMFLFYATYCRIAKFPMLIKNSMNIVALRNSYKLIVDSLEK
TQTSFDIVFIIALVFSVPEIMMSIYSTLLEVISKHFLEVASILSLNYVAIAQSLLLTLAPSLCAGVLPWKTNNIKI
ILHEKLFTEKDKASAREIELFIKYIESRPLKLRACNLVPLDFSLTIIVLNICVTYLIVIIQFTHLY
>BmorGR15
MISSSDINHKRNKVFAYNVPGIALSKTLTVLFKLLHYVLLLDVGIYEYKTFKNKCIVKFLTIATGVSVSIVYFCLI
ATVLRKNAFFYWFYVLFISQYMIIVFIFTLSNGMSFTDYYKMLLRFDAKYQINSNNYYFNIKIILVIIISILNRIG
MAIIYCSYYTKNCYEMSFSQIIFVLPWLTRDVILIMNVFLFYVTYCRITKFPALLENTKNVGSLRNSYKLIVDSLE
KTQKPFDFVFTISLVFNIPEIMLSIYFTLLQVIHSHFLEVAPTLSISYFSITHSVVLILAPSLCAGVLPWKTNTIK
IVLHDKLFLEKDKNSARNIKLFIKYIEARPLKLRACNLVPLDFSLPVIVLNLCVTYLIVIVQFSHLS
>BmorGR16
MIMNLTTDRISKRNKVFAYNVPEVTLPTTLKVLFKLIQFTLSLDFGVYKYKTFKMKCVAKVLTLAGCLAASAACVS
LIISNIFENQLFFGWYTLFVCQYTIVIFMFTFSNGMTFIDYKMMLLRFDAKYQIDSNVYHFNIKIVLVVVISVTSR
LFLCAVYCIYSTENCIKPWYNQLLFFPWLSLDIVLIMNMFLFYATYCRLAKFPSLFENPKNVVPLRNSYKLIVDSL
EKTKKSFDAVLIAALIFNIPEIMMSIYYTLFQVMNKHFQEVAPVLSLSYFTIILSVLLILAPSLCAGVLPWKTRHM
RLILLEKLFAEKDKNSAREIELFIKYIEARPLQLRACNLVPLDFNLPVIVLNLCITYLIVIIQFT
HLF
>BmorGR17
MGFSLGTTALSMFFFEKPVVFTIIQITMIIVKPAKYKLSDPFRPKDTSKLSESIIMYFKLFHIFLGIDLGGFRYQN
RQVKYAVRLISLIQPLAIYGLCIYALLKIIANTEFLWYTISFTEYVAMSVAITLFSNEMTYCNFMINLKFIDTKLK
IGDESFRIGVKLISSTILIGVTRCFTTTTYCLLGFCAKPTAAQILFQIPWLTIDLMLLQYMFIFYACYCRLVKILR
ILKKRNTDIEEMRRIYKTLVDVLDRARAPFDLAYLLGLLFSIPDVLYSIYESIIKVGEINTAKALSMSIIYITNIQ
SLALMFAPALTAGFLPSLTMKMRIILHDKLLEEQDKKTYRHIVLFIKYIETCPLKLKACQIIPLDFSFPIIILNIV
VTYLIVAIQLTHFL
>BmorGR18
MRRSTKVISMVNQSDKGEIKTCSRFMKIYFFVIYILTGFNFGFYTGRGLNFLRVIQASVLLLRFIIASNCIYIAFH
FRLLEAIWYSLTFSESLAIVVCFMLSRSALSCKNLFEYLYSVDQELKKSVGPSIEVKLALYTVVVSVLRLTVYVFC
AIAYYETLHEGFCVELVYNTPCYCSDLYLVIHFTIFHSVYCRLKALRISMNEKFDVYKGTLIYKSLIDNLEEIKKS
LDVPFFVILLNAVAIAMINILVTLEISYGQTMKFIRTAPRYLETVLLFSSAFAPVLAADMMASEAQKIKVTLNNIL
QRDDSLLEDDRRKVKQFAGYVSARPFRLRACRVLSLDCTLPVTVLSICVTYLIVVVQFTHLY
>BmorGR19
MRRSTKVISLVNQSDKGEIKTCSRFMKIYFFVIYILTGFNFGFYTGCGLNFLRVIQASVLLLRLSVASYSMYIARY
SPLLEVIWCCLTASENLAVVVCFMLSRSALSCKNLFEYLYSVDQELKKSVGPSIEVKLALYTVVVSVLRLIIYVFC
ATAYYRKLFDGLRLELLYHTPCYSLDLYLVVHFTIFHSVYCRLKALRISLNEKFDVYKGTLIYKSLIDNLEEIKKS
LDVPLFVILLNAVAIAMINILVTLHISYGKTGCRIDYKSVAEMACSQLVIRTSRSETANGSSEAKRKKLVVGPLMK

LITAAPRYLETVLLFSAAFAPVLAADMMASEAQKIKVTLNNILQRDDSLLEDDRRKVKQFAGYVSARPFRLRACRV
LSLDCTLPVTVLSICVTYLIVVVQFMHLY
>BmorGR20
MRRSTKVISLVKQSDKGEIKTCSRFMKIYFFVIYILTGFNFGFYTGCGLNFLRVIQASVLLLRSIIASYSIYVAIH
FRVLEAIWYCLTFSESLMVVVCFMLSRSALSCKSLFEYLYSVDQELKKSVGPSIEVKLVLYTVVVSVLRLTVYVFC
AIAYYESLHEGFSVELIYNTPCYCSDLYLVVHFTIFHSVYCRLKALRISMNEKFDVYKGTLIYKSLIDNLEEIKKS
LDVPMKFIRTASRYLETVLLFSSAFAPVLAADMMASEAQKIKVTLNNILQTDDSLRKSYGATVFSFALLVTMIVEA
TMVGVEDNNIIGPYIDVEREARLTGLYVMAIILTLMFLAKFIFDLVFVYGVVMERAGIVKAYFIMWAVFFFLSVSV
FFLNCLDFNTSTIVLEVFYIGLNIYAILLSHSFYKQLNTREDV
>BmorGR21
MAQRTNSINLFRSRPPDIRAGVGEPRIFSKFICGTMFTQKSLVNFDLGKTPRGGDQEHSKFFKIYFLAVHSVTVLD
FGFDRNAKKLTKILISMFSISVRMGLAAVSFMSLWGRPNALALGWAPGTLLCENILVAVTYSASRSTFKCGDLFAD
LSTIDELFGSACDYRIESKMLLFTATMTVLRVVIYSTSRLVRADGFDFVDVLEVLNNLETMCMYLFLTVYFFVLFS
IYCRFKKLRELMKNDFEIRRANLIYIALKDCTDKIKQSLDVPFLVVLVFTVLVVMVDVFITLEMIISNKYNMAVYV
VRYLEITLDFLMLFAPVLLADMMAVQVDGLKITLHDRLCLNNGVGHDDLSDAMWMSFVDMKRYSSLAEFIGYVEAR
GCRLRACRVVPLDLTLPVTVFNVCVTYLIVMIQFADLY
>BmorGR22
MDFGFSLGVYKRMKVLENISLVLRVMVAIMCAAMVMKQDILDSAWADITLTESLLVIVSFKLSKPKLSYRELLENL
SIVDETQGAPPAGYKVERKLITYIAGVTALRLTVLCLYCVAHTEQYSIDNFIEFLYNVPCYCLDLYLIVHFIIFHS
IYCRLRTLRKALSNNFDVYRAHLIYKTLIDCTEEIKKCLDIPVSRSDRHRHSLHKPMGTFLKSYRTPRVMFQLVVI
LIATILVVMVNVLVTLRMLFKGEVTQRGSVHVQVVLPENLPLSYFNIVHCISLQSIISAFLLRYIEVILSLALLKL
LDELKPDAFKSHWKKIWRVLIVGNDGQFARNQTSTDNIVQAANQWNMTRWIRADRTSDIREVAGSQDHLTETDLEE
MLTTRSCVTAYWIFDAGSEHVWYVAAAEERREVRQFAHYVGTRPFRLRACHVLALDSSLPITVVSVCVTYLIVIVQ
FTHLY
>BmorGR23
MAQFQIPSMAGSGLNVAPFSRGRRHEGPKHSNFTKKYFLLVHLVTCLDFGFHRDNDTKTYKWFHAANIGVRLVLSA
YVCSVSLSQDLSFASAAWTILNNSKHLLVVAIFTIFKPKSSCAEILKDLLMIDEALKIHRGCDVKGQITVCIVLVT
AARLLIAAASSLSLHEAFSASVGAAEVLYSFQSYCLDFYILANFFIFYSVYCRLKNLRRVLQNNFNIYRGNMIYKV
LVEHMDDIKKFLDIPFVTSLLVTVIMAMINVLKTLQLIHDGENDVLTIVLRYLEMFLSFSLIFAPVILSDLMSIEA
DNINVVLHNYIYETDAADIAVAVHPSARPSPVSATEERTAAAAAASVSLAIFSSRLTSFNLCWRARGAGAETFVAV
CVVRLGAAVPTPRVSGALAGLHAPRDRAQHLRHLPHRGRAVHASLLISLSLL
>BmorGR24
MCINKKIQSIIKSLVSIRTIMLVQSILGFYHKMSNNFFVSFLFLTYTTILISVLSFYSVNDVMAHKFAYTLSMILE
YDINTILSLITAGRQYFNFFEEMKKIDFSIGFGELNIEDLPLSRTLFVTIFVTNILLSIMTAALILFFSTPFLIIS
SGSTYAMAIVFFGLSLNVLPRIIIFELIYKRIKYINLSLKRK
LKALALECDHTIARFEIINENLIIYNKLLQSLGNVNVSLKSSILLTTFTCFFRCSLICYYVITMNEDKVYIMQIIE
LTKQTLFLGVLIILAEYIKNEIENLKMTVSLQLFTCTDTQLYHQVSDXFEIHRITPIQLCGFKNMSVDTNLFLGLI
NVCSTYLIIITQFLNAYVN
>BmorGR25
MFVKCLKYVKKFKPMFSVMFIMNFRLICGLYYRIHSDAFVCFVFKVYCILCSMFLFFTSSDLAAPFSRSIPILATL
FEYVANVLDCILTGQSYFFHLRMELMRIDPRLRGLDRPPASSIVFTAILSYKIFILAVYIHGKARTTYLQYEWFSS
IGIHLLVLFSNLVHMNRMLIFEMVTFSLEAQKKTLGELLKSSLRRERVERKCEILNRFLKTYKRIIELFNNTMAAT
KLMTLISVVSCFIRILTYLYQVLTTQLSSSSAGSIFTTRHLTFIVTFVREHVLSVYFLKKVGICQRLQINFKIKCN
VKTETLELISQDDEYTEKLEDALDFINSCSSKITILRAMTVDATLPLTFISLCTTYIIVVIQFSHIYD
>BmorGR26
MNKTKIYRKKLDKNERLVCSVQPAMFARLIVGLYYDIKVSNRVKWMIKSYCISLSSFICYLIIFRDDNFSLHPKLT
SVMEYITYVTFSFLTCDKYLFRYLRFNPRTDGYPIFLYLCKKFEKFFKIIICLFVSFKILGVVLMMQSWPILSTPK
YIWGTLALHFLWLASHMGRLVFILVYGILFCRMRTIRIIFENRGFQNTPQNRLTPKRYILMYEAVLNSIESVDFPV
KFLIFTFICCFAPKLVVSLFEIMEEMKKGELSLTTFIWFLVELSPSYLFLLLSAIALDLVSEDVQELLSITIDRRL
NCKNEKERSEIQEFFQYLRNNPFNYTLWQVVSLNLRTLLVATSFSIANVIAIMQIKNSKI
>BmorGR27
MVFKYKIMTKAPKSLPVLKILMLFRLVFGNYFRLSSNRYINFLVKSYCSTFTILLSVMCGKRLKNDSPYMLSLTEY
ILNKILNYATSEGYIFKYCNSIKTCDKIMGFKKLPIITIDVFIAIIITVITRTAITIYFGFLFPFDKYQVVLYVGC
IVFSNDLNSLTIMNVFGLLNNRMNLLRKSLEAMTVPINIIGKNEVAPKVRLVRNAFRYYSNLLDNLDSVNHCVQYS
LSVTLLLKFPKAVLLCYDSIKTYFVKIDNNFAMDIVDPTEIILSIVVMSFPAMLCEMITNEVEKIKAILTKHLIQC
SDNSLRFELNITLLYICHRPFKYILWRAIPLDTSVPIGIVSLIITYVIVLIQLLHFST
>BmorGR28
MAHKIATVGPTNATATVKNNKRKLKISKRVTIFKVVRCLRFILGHYTELTSSKLKAFLIKCCSLLLAVIIIYAPLN
YIKMAYVMGLIEYLLFVLLSLFTGDEYFYKFHNSIKSIDVLMGYKRGKIIDSNAIIFLLSVITIMRIVIIYCRSTV
LAFRFTIIGVYLAIFSLRISYMLITVIFFAMYHRMKFLRKKFEIITIPVTIIGKQKVASKIRLIRKYLINYHHLLD
CLRDINGGLQYFLAIMIACNLPKYIFFAYSAIKIQVLEHITIHSAVQNVEMFEGFLFVVVPAIFAELTTAEVERII
DVINRQLLRCTDEHMELELKVALEFIRRRPFDYVIWRTVPLNASLPIAIISLCITYVVIVIQLTQFHDNF
>BmorGR29
MYLRSKKSRFKLFSFERMIKILLMICGHYVQTDSSNVVSSIHRLFSIAITICLCPNFEFNPFYFHVIESVLYSILS
QFTQYGFFFRFCSTIKTFDLLSGFKQIPLYTKRVCFFLLITLFMRLFTVLIHFLAYQSKFVTFCAFIIMLSANTGH
ILMTIMFSTLHTRMKSIQKLFANNPIPVNIVGKNENASHIKRVRKGLICYNNLLDTLKDAEKEIQFTLTVTCLCHV
PKIICYVYFVITVIYKSKFSGYNLVPLFDMILACMAVTAPAVFAELTKNTVDKIKKILGSQLLRCSDESLRYELEI
TLEYVIQRPFSFSIWRAVSLDASLPVAMTSLCITYVIVILQLTQLRP

>BmorGR30.1
MYLRSKKSRFKLFSFERMIKILLMICGHYVQTDSSNVVSSIHRIFSIVITICLCPYFQFNPFFFHVIESVLYSILS
QFTQYGFFFRYCSTIKTFDLLSGFKQIPLYTKRVCFFLLITLLVRLIIVLIHFSAHQTKLKTFCAFLIILSANTGH
ILMTIMFSILNTRMTLIQKLFANNPIPVNIVGKNQNASHI
KRVRKGLICYNNLLDTLKVAEKEIQFTLTVTYLCHVPKIICYVYFVITVIYKSKFSGYNLVPLFDMILACMAVTAP
AVFAELTKNTVDKIKKILGSQLLRCSDESLRYELEITLEYVIQRPFSFSIWRAVSLDASLPVAMTSLCITYVIVIL
QLTQLRP
>BmorGR30.2
MYLRSKKSRFKLFSFERMIKILLMICGHYVQTDSSNVVSSIHRIFSIVITICLCPYFQFNPFFFHVIESVLYSILS
QFTQYGFFFRYCSTIKTFDLLSGFKQIPLYTKRVCFFLLITLLVRLIIVLIHFSAHQTKLKTFCAFLIILSANTGH
ILMTIMFSILNTRMTLIQKLFANNPIPVNIVGKNQNASHIKRVRKGLICYNNLLDTLKVAEKEIQFTLTVTYLCHV
PKIICYVYFVITVIYKSKFSGYNLVPLFDMILACMAVTAPAVFAELTKNTVDKIKKILGSQLLRCSDESLRYELEI
TLEYVIQRPFSFSIWRAVSLDASLPVAMTSLCITYVIVILQLTQLRP
>BmorGR30.3
MYLRSKKSRFKLFSFERMIKILLMICGHYVQTDSSNVVSSIHRIFSIVITICLCPYFQFNPFFFHVIESVLYSILS
QFTQYGFFFRYCSTIKTFDLLSGFKQIPLYTKRVCFFLLITLLVRLIIVLIHFSAHQTKLKTFCAFLIILSANTGH
ILMTIMFSILNTRMTLIQKLFANNPIPVNIVGKNQNASHIKRVRKGLICYNNLLDTLKVAEKEIQFTLTVTYLCHV
PKIICYVYFVITVIYKSKFSGYNLVPLFDMILACMAVTAPAVFAELTKNTVDKIKKILGSQLLRCSDYS
>BmorGR30.4
MYLRSKKSRFKLFSFERMIKILLMICGHYVQTDSSNVVSSIHRIFSIVITICLCPYFQFNPFFFHVIESVLYSILS
QFTQYGFFFRYCSTIKTFDLLSGFKQIPLYTKRVCFFLLITLLVRLIIVLIHFSAHQTKLKTFCAFLIILSANTGH
ILMTIMFSILNTRMTLIQKLFANNPIPVNIVGKNQNASHIKRVRKGLICYNNLLDTLKVAEKEIQFTLTVTYLCHV
PKIICYVYFVITVIYKSKFSGYNLVPLFDMILACMAVTAPAVFAELTKNTVDKIKKILGSQLLRCSDESLRYELEI
TLEYVIQRPFSFSIWRAVSLDASLPVAMTSLCITYVIVILQLTQLRP
>BmorGR30.5
MYLRSKKSRFKLFSFERMIKILLMICGHYVQTDSSNVVSSIHRIFSIVITICLCPYFQFNPFFFHVIESVLYSILS
QFTQYGFFFRYCSTIKTFDLLSGFKQIPLYTKRVCFFLLITLLVRLIIVLIHFSAHQTKLKTFCAFLIILSANTGH
ILMTIMFSILNTRMTLIQKLFANNPIPVNIVGKNQNASHIKRVRKGLICYNNLLDTLKVAEKEIQFTLTVTYLCHV
PKIICYVYFVITVIYKSKFSGYNLVPLFDMILACMAVTAPAVFAELTKNTVDKIKKILGSQLLRCSDESLRYELEI
TLEYVIQRPFSFSIWRAVSLDASLPVAMTSLCITYVIVILQLTQLRP
>BmorGR30.6
MYLRSKKSRFKLFSFERMIKILLMICGHYVQTDSSNVVSSIHRIFSIVITICLCPYFQFNPFFFHVIESVLYSILS
QFTQYGFFFRYCSTIKTFDLLSGFKQIPLYTKRVCFFLLITLLVRLIIVLIHFSAHQTKLKTFCAFLIILSANTGH
ILMTIMFSILNTRMTLIQKLFANNPIPVNIVGKNQNASHIKRVRKGLICYNNLLDTLKVAEKEIQFTLTVTYLCHV
PKIICYVYFVITVIYKSKFSGYNLVPLFDMILACMAVTAPAVFAELTKNTVDKIKKILGSQLLRCSDESLRYELEI
TLEYVIQRPFSFSIWRAVSLDASLPVAMTSLCITYVIVILQLTQLRP
>BmorGR30.7
MYLRSKKSRFKLFSFERMIKILLMICGHYVQTDSSNVVSSIHRIFSIVITICLCPYFQFNPFFFHVIESVLYSILS
QFTQYGFFFRYCSTIKTFDLLSGFKQIPLYTKRVCFFLLITLLVRLIIVLIHFSAHQTKLKTFCAFLIILSANTGH
ILMTIMFSILNTRMTLIQKLFANNPIPVNIVGKNQNASHIKRVRKGLICYNNLLDTLKVAEKEIQFTLTVTYLCHV
PKIICYVYFVITVIYKSKFSGYNLVPLFDMILACMAVTAPAVFAELTKNTVDKIKKILGSQLLRCSDESLRYELEI
TLEYVIQRPFSFSIWRAVSLDASLPVAMTSLCITYVIVILQLTQLRP
>BmorGR30.8
MYLRSKKSRFKLFSFERMIKILLMICGHYVQTDSSNVVSSIHRIFSIVITICLCPYFQFNPFFFHVIESVLYSILS
QFTQYGFFFRYCSTIKTFDLLSGFKQIPLYTKRVCFFLLITLLVRLIIVLIHFSAHQTKLKTFCAFLIILSANTGH
ILMTIMFSILNTRMTLIQKLFANNPIPVNIVGKNQNASHIKRVRKGLICYNNLLDTLKVAEKEIQFTLTVTYLCHV
PKIICYVYFVITVIYKSKFSGYNLVPLFDMILACMAVTAPAVFAELTKNTVDKIKKILGSQLLRCSDESLRYELEI
TLEYVIQRPFSFSIWRAVSLDASLPVAMTSLCITYVIVILQLTQLRP
>BmorGR31
MYLRSKKSRFKLFSFERMIKILLMICGHYVQTDSSNVVSSIHRIFSIVITICLCPYFQFNPFFFHVIESVLYSILS
QFTQYGFFFRYCSTIKTFDLLSGFKQIPLYTKRVCFFLLITLLVRLIIVLIHFSAHQTKLKTFCAFLIILSANTGH
ILMTIMFSILNTRMTLIQKLFANNPIPVNIVGKNQNASHIKRVRKGLICYNNLLDTLKVAEKEIQFTLTVTYLCHV
PKIICYVYFVITVIYKSKFSGYNLIPMLDMILACMAVTAPALFAELTKNTVDKIKKILGSQLLRCSDESLRYELEI
TLEYVIQRPFSFSIWRAVSLDASLPVAMTSLCITYVIVILQLTQLRP
>BmorGR32
MCYTNFVSRQVSKCINFFSTIRYVIYLRMFCGLYYNCSSSFKIRCIARLYCFIIYCLNLHYNLYIFTSSVSLTNFL
HTFITLAEVSIHILFSLYTGESNFMSFCIEMNKLTSGPIDFVATKCVATHFIAFFVIGLHILSSTLICGAEVSCFT
FSVVLASMTFLTTLLSRFTTIIMFDLVWIRMRSLRKILVNALESDLSEDEKVKSIESFLKAYKQIIASIRITKLAT
RNLVTFNFVSLFGKIMTLIYFCINCPGYLNTYLISSWIFGILLAGFVTCAPPVLVEMNVNELDEIKYALADQLVDY
TDDKYRTAIYNALDYVEVHSIRYTLWKNFPMDLTMFFGFAGFCATYIIGLLQFTY
>BmorGR33
MCYTNFVSRQVSKCIHFFSTIRYIIYLRMFCGLYYNCSSSFKIRCIARLYCFIIYCLNLHYNSYIFTTNVSLTNFF
HTFIILAEVSVHILFSLYTGESNFISFCIEMNKLTSDPNEFIATKCVTTHFIAYLVIVSHILSSTLICGARASCFT
FSVILTSMTFLTTLLSRFTTIIMFDVVWIRMRSLRKILVNALESDLAENEKAKSIESFLNAYKQIIASTRITKLAT
RNLVIFNFVSMFGRIMTLIYFCINNPGYLDTYHMSLWIFGILLAGFVTCAPPVLVEMNVNELDEIKYALADQLVDY
TDDNYRTAIYNALDYVEVHSIRYTLWKNFPMDLTMFFGFAGFCATYIIGLLQFTY
>BmorGR34
MCYTNFVSRQVSKCIHFFSTIRYVIYLRMFCGLYYDCSSSFKIRCIARLYCFIIYCLNLHYNLYIFTSGVSLTNFF

YSFITFAEVSIHILLPLYTGESSFMSFCIEMNKLTSGPNEFIATKCVATHFIALLVIVSHILSSTLMCGARASCFT
FSVILASMMFLTTLLSRFTTIIMFDVVWIRMRSLRKILVNALESDLAEDEKAKSIENFLNAYKKVIASIRITKLAT
RNLVTFNFVFMFGKIMTLIYFCINNPGYLNTYLISSWIFGILLAGFVTCAPPVLVEMNVNELDEIKYALADQLVDY
TDDNYRTAIYNALDYVEVHSIRYTLWKNFPMDLTMFFGFAGFCATYIIGLLQFTY
>BmorGR35
MYSSLKLKDYIVNLESVMCSDQSITISFKNVFTVFFDYISSLDFMMVCRLCFGYYYEFNCSNLCKIMFKCFSISVC
IFCVSMHLVQLISPPYLNHCVIMLESTVSIITSLVTEDKYFFEFCLDMKDINSMMNQPRNIKSFKIIYVIISGAIC
HVIRHISICREKALSFCFSTEYLTASFTIISGYWNYLNITMMFDLLYQRLVAVKQMLTNGLNICDTDEYKIKSVQK
FIDVYKALTVSLSKTSNLIKHTVSLGIFCALWRIIFFVYYCISMDFQVESAQFITWVSSMCLSVFLVYIPALIVEL
CSNEVDAIKWILASELLEYRDKRLRTSLRDALDYIDVCPIDFEIWHCFPMNLSLCLGFIDISSSYIISIL
QFKY
>BmorGR36
MTVPYDKIKSALSKFVQLLFSINVVLFVRFLFGFYMKIGSRKYFHIATKIWIVTLTIFRVYFQCRNFMNYPSYALI
LHDFTCTVELVILCIISSLGGEQHFYTYCSEMAELIDNRKNKRASYFTTSTLLIGFIILIVPTSISCKKVSNALSM
LFLNIFNYVACFMHHLTIIYVFELLWREIRKCRISLERLEIMSVDDKIMKIENFLDSYKRSLDSLNKANGVMIPTM
ALAYFAIIAKIVFFTYNILSLRGFNILVHGDSWLLSTSIAIIFICAPALLVELAANEVNKIQNLLAVELLKMKDDK
F
>BmorGR37
MSAKINYQDKLCSIKSIMYLGLFCGLYFRSSTSRMMLLMTKVYCIVLLFLGISFHLNMLSSDLPTETTLHSSIIML
EFFIHIITSVSTGQAKFLHFCTEMMKINGHNSKGSLDFQLVITNIALIIIIITQTTSSLLYCIIRSKCLSHSYVFT
VITSLCVLFSSFTMIIKYELIWNTVRSLKNTLVSNLDSFDLSEQEKVNSVYNFLSTYRDIKANVDLTIKGTRTTMI
FNLFFLFVKILFLVYYCIINAENLDCLHASARVSAIVLSVAVTCIAPILVEINVYEFGRIKFALADQLLEYTDNNF
RIALHDALDYIEVHSIRCSIWKNYPMDLNLCIGFISVCATYTISILQFSY
>BmorGR38
MKPKYVTNIKLVLFLRFLCGYYYEMEIPRRLKTVAKAYCIFFLFFYLVLHHLYCSFSNHTAKWSLYLEYSIYVFMS
LYSKKMYLMDYYTSSRIIDFEPHSRIYKKLNIYLAILIPFLVVLKIVNMVTFCLSKSFNCWSWVSLLHNLLWNFTV
LGRIPPVFVFALLFCRTRIIRRTLVSITVGPGSTVLKSLFKCTRYWLTVLKRPNTHLNSCYFFQLTVFLLCSTPKL
ILETFLMLNKIKESGPIVEKLAVYAIETFYTHLFFVVSSILFDLINVDLQRIKILIVEKRMKTKNTKHRIEVEKLF
QFVKSQTIECTLWRVLSLNVRNILSFVSFAVTTIIAVLQIKNNNIY
>BmorGR39
MNVSPQYSVLKIFKPLFKVQTLFGSVRVKVGDNGITKTTKLQKFYSIFNILFATTGHFYTSFVYSVCVPCVGNSVA
ETSMALIQIYAGHLMNSFIVFSNTFLHYEKNVQMFKSLCSIDELMKIVRLEHRDLKLFIAIILLLSSTVIMNVYFL
IYMVIILPISEKWVPFANIGIMNEDLEAITFVSVLYMLYDRVKYINQTALDPVNIAKLIKESDGQNDEETVSRILK
AFKEISKAYKIVEKTFRVFFMFLVIHMFFFQMLTVEILIVLSDNLSWGTFMSKNLIGVKFIARIMLISVCVSYLER
ELQKTKGLCNLAVRNCENDIVRCHLKNIYRIIDTEIEPMTVFGLFYINNVPLDLISLTATYTVVLL
QFAFL
>BmorGR40
SMLVFCVLWSGLCYTGFIGICGMNFIWIATFMGRMVLPLIFGILLCRIRMFRLTLQKQGFDNLPYNRFSPRRYIMM
YDSIVRGLEKTDFPAKNIMFVFTICIYSKILTGLFDLISVLKREGPKLMNVMLFTLEFLPSYVLLMIYSVTLDMVS
TEMKEILKIVTEKRVFCKDKSHANIQELCQYIKNNQLKYTIWRLVSLNMQSLLRATSFCIVSTIAILQIKDWNG
>BmorGR41
MSENSLEEYIHMSFSPIYKYQKFLGSNRISLKAKNKITVANNWEKLYAFLWMLAASYSIHHFISFFYSYYYERSNI
IFLACSLGISMHYLTYILTITYDKFLTREADIDLFIDIQKIDRLLKLDRCTVLFKKFRLINIFLLILVTVPFISGF
LIHVFDYIDKPYKTFFLGLGVTITYVDVLVTAFFITKLTLRLAYINDRIAMYNKINIPHKKYSGIRRSILWIFGWR
IFKIMPKIKKNGTREKKSTFIKYPSIIFNILKCYRSITEIYSLPVFLITATVSIWTFLVIGSLVAGSRSEIKIFPV
VAMITVGLWNFYYIIQLTSLAFVNDLFLMEVKNTKQLCISVLLYTCDDSINKAANTILKNIECVPPIFSVYGIFVF
DKSIILFLFGIITSNVMTVIQFSY
>BmorGR42
MNKTKKIERLSRDILDEDFIDVFKSIFIFQRIFGLLSVNITYKYITETSKLYKLFVMSLWTVNVLCLVDYILNYRT
SFDVATDSMLKLVMSVNVTTNALIVWRNNFKLNTLKSQIYVKLQNLDRDLKTKDAVTMNKKLSALSIALMICGFIW
CTIWLFVYNAIAMNTFCVPLTIILSANVGNWLEMVLLFIIFYFVNVRAEYVNKLLRRRLNQTECPDRVFLIQNAKP
SDTVSREFICGMQSLLEIIGNIKDIYQFPIFLSTCQVMLCILVIVQNLIISVKEQMGGRAHSPPGFKWLLEPIDIY
NTSTMVDSMLCMLPALLLMLTIFFSLCVIAEALTSKLDITKKLCAMGMHSFTDDISRRSSKQIVLLLEAKRSMSVF
NIYTLGTRLPIHLLGVTASYTIVLLQFAVL
>BmorGR43
MKSPEYLSKDILDEDFVRVFRFPFLVQMALGSCRVHLKARFITIPTLGQKLYTVMSIIICSLLYFNITKLYISLYY
QHSIVYYLFLAVAGLDQLSFFANLIHVRFLNGETNTGFCIMMQRIDRKMKIDHNNIFNKTVIRANILTITLIILLY
MSLVISTIILKKYSLVTLFGLVHGQLILLVEMAYCSNLIIFFFIRVRFVNAIIKNHVHPENQNQPPKLVRYFVTNR
IMRYLAAQTHDFIVNDTDVYLKQIFEGFSMFIDIYRFQASMPLLHKANCNEPANLRILLGWDTKKFFHTEFGSLIN
SPCDFAFFFSQEKIAGSPPARQVLASKNLANYYVIVHSVIGFITALYISGRCEFFFREIRETKRLAVAVLLKYQEG
TILTIAYFPKLS
>BmorGR44
LGGSRVAICLFPFEHLALFLGVSCSYKISGMPVWTPLQIVVLSMTSGTRGASRCTTLVVLDVLRLRMNFLKRLLEK
TMQSEISNQQKYQFLEQFAEHYRYLLEALDSTRPHSAIVVTTMLMTSFVKVLIIIYFVITNSIMTIDNSRLTYMMM
TSFEVAAVSVAPALLEMANNEKDQIIMILADQLLKYTDSKLRRSVYDVVEYITVAPPTVTIWPEFTVDLGLYMDFI
GLTVCYCIMILQFEY
>BmorGR45
MKSPEYLSKDILDEDFVRVFSFPFLVQMALGSCRVHLKARFITVPTLGQKLYTVMCIIICSLMYFNMTKLYLPLYY

EHSIVYYIFVTVTGLDQLSFFANLIHLRFLNGETNTAFYIMMQRIDRNMKIDHNNIFNKTVTLANILTITLIILHY
VGLVISTIILKEYSLLSLFGLLYGQLMLMVEMALCSNLIIFFFMRVRFVNAIIKNHVHPENQNQPPKLVRYFITNR
ITRYLAAQTHDFIVNDTDVYLKQIFEGFSMFIDIYRFQVCPLCIKLVVLTLLNFEFCLVAIQRNVLGPNHIGNYYI
IVNSVMGFFTALYVSGRCELFFREIRETKRLSVAVLLQYQEGPLREKATRMLKIIEESTPQFSIYDMWQMDGYTFV
KICSLVTNLIVTLLQFAYL
>BmorGR46
MITMKSPEYLSKDILDEDFVRVFRFPFLVQMALGSCRVHLKARFITIPTLGQKLYTVMSIIICSLLYFNITKLYLP
LYYQHSIVYYLFLAVTGLDQLSFFANLIHVRFLNGETNTAFCIMMQRIDRNMKIDHNNILNKTVIRANIFTITFII
LIYVVLVISTIMLNEYSLVTLFGLLYGQLIFMVEMAHCSNLILFFFTRVRFVNAIIKNHVHPENQNQPPKLVRYFV
TNRITRYLAAQTHDFIVNDTDVYLKQIFEGFSMFTDIYRFQVCLFCIKIVVLSLLTFELCFVAVQRNLLETKNLTN
YYIMTYSVIGFFTALYVSGRCELFFREIRETKRLAVAVLLQYQEGPLREKATRMLKIIEESTPQFSVYDMWNMDGY
IFIRICSLVTNLIVTLLQFAYL
>BmorGR47
MIDFKTRNVCDINTIMKIRFLFGFYCDFPFNKRFQNILKFYCISVLVVLILGSWACSTGFRSDKKIVIYCEYIAYF
LISLSTKDRYIFDYYKQQPLIDGSTTSKVLYKKLERLLKYFVTITIVLKMLNIFVFCGWNLTKCINELDGVLFINL
LWIGLLLARLSLPVIYGLLYFRLRVLRMTLESKGFSNSPQNRFTPKKYITIYEKIMKDLLKMDYPLKYVFIIFLIG
SVPKLLQNSWQFLNSLKNYGPEISKILEFTLECLHSYIVIILPIVVALDLSEDEIKKMKIITLNKRLACLNERQKM
EIQQLFLLLKNNSLRYNLWRVVPVNLKSVLIFLSFGVTNAIAIMQAKNLN
>BmorGR48
MTVFMLKAQVDQDTILEKVVKPLNNTMFNNQEWSFQQDSAPGCSLGTSSQYLTYILTITYDKFLTREADIDLFINL
QKIDRLLKLDRCTVLFKKIRLIYIFLLILVTVPFISGFLIHVFDYIDQPYKTFFLGLGLTIIYVDVLVTAFFIANL
TLRLAYINDRIAMYYKRSLPLRKDSGIRRSRSWICGSWIFQIMPKIKNNGTRMKNYTFIKYQSLIFNILKCYRLIT
EIYSLPVFLITATVSIWTFLVIGSIVAGSRSEIKLFSIVAMITVGLWILFFIIQLTSLAFVNDLFLMEVKNTKQLC
IRVLSYTRDDSINKAVNTILKDIEYAPPIFSVYGIFVFDKSIILFLIGIITGNIMTVIQFSY
>BmorGR49
MAGIRTISFKVKPLELPDVSENNFADDGLKIVQRFKFFIYIQVITGINRLYLLKCNKFVMLFSYLYAIFLISFVAS
VYWTKEPMKNSHLVIRLFSFIEYILLICISVFLKKKKMMKFFENLSMFDQILKIDKNVNSTFCMKRVFFWVTGSIV
YNLIEFYALEFYDNTSKGLMTIICTYTIALTHDCEQIFFFTLQRVVYLRLLVVKRHIQEHFKVDEDSNRKKPNKYE
MLSKNVQLNLTALHEVYGLLHNCAEKLNKIMSIPVLLMLFTSGLTTTILLKILVRVIQLADPSNPGPAAIGVCVYL
IVHCIKYTLLVVIPCYYSSITATQVSLIRITLHDAINTIPLGKCKLQRRKVKAFYLMTKEYSFVYTLAGVIKLNMS
LPLSYISLCTTYLVIIIQFSKFLD
>BmorGR50
MAGIRTISSKVKPLELPDVSENNFADDGLKIVQPFKFFIYIQAITGINRLYLLKCNKFVLMFSYLYAIFLISFVAL
VYWTTEPKKNSHLVIRLFTFFEYTLLACISVFLKKKKMIKFFENLSLLDKMLKINKNVNSTCCMKQVFFWVTGSIV
YNLIEFYAMEFYDNTNKGLKTIICTYAIALAHDCEQIFFFTLQRVVYLRLLVVKRHIQEYFKVDEDSSRKKPNKYE
MLSNNVQLNLTALHEVYALLHNCAEKLNTVMSIPVLLMLFTSGLSTTILLKFFVRVIQLTDPSNPGSAIGVCMYLI
VRCIKYTLLVVISCYYSSITATQVSLIRITIHDAINTVPLGKLQRRKVKAFYLMTKEYSFVYALAGVIKLNMSLPL
SYISLCTTYLVIIIQFSKFLD
>BmorGR51
MAMGIRTILSKVKPLELPDVSENNFADDGLKIVQRFKFFIYIQVLTGINRLYLLKCNKFVMLFSYLYAIFLISFVA
SVYWTKEPMKNSHLVIRLFSFIEYILLICISVFLKKKKMMKFFENLSMFDQILKIDKNVNSTFCMKRVFFWVTGSI
VYNLIEFYALEFYDNTSKGLMTIICTYTIALTHDCEQIFFFTLQRVVYLRLLVVKRHIQEHFKVDEDSNRKKPNKY
EMLSKNVQLNLTALHEVYGLLHNCAEKLNKIMSIPVLLMLFTSGLTTTILLRILVRVIQLADPSNPGSAIGLCVYL
IVRCIKYTLLVVISCYYSSITATQVSLIRITINDAINTIAFGKLQRRKVKAFYLMTKEYSFVYTLAGVIKLNMSLP
LSYISLCTTYLVIIIQFSKFFD
>BmorGR52
MAGIRTISSKVKPLELPDVSENNFADDGLKIVQRFKFFIYIQVITGINRLYLLKCNKFVMLFSYLYAMFLISFVVL
VYWTTEAMKNSNLVIRNFTCLEYILLICIAMFLKKKKMIKFFENLSCLDKMLKIDKNVNSTCCMKRVSFWVAGSIV
YNLIEFYAIEFYDNTNKGLVTIICTYTFALAHDCEQIFFFTLQRVVYLRLLVVKRHIQEYFKVDEDSSRKKPNKYE
MLSNNVQLNLTALHEVYALLHNCAEKLNTVMSIPVLLILFTSGLSTTILLKILVRVIQFTDPSNPGQQSECACICI
ASNTRCLSRAITQASLQLKFLLFVSRFMMPSTLFHWVSKLQRRKVKAFYLMTKEYSFVYTLAGVIKLNMSLPLSYI
SLCTTYLVIIIQFSKFLD
>BmorGR53
MAHIKDENQSKQQQKEHETLNKNKLKKVVYTLKPALMLENWFGLSDFLLVNEDELVLLMQTEKFGVILSIFFIVMF
AVFVDFPDTETESIMELMDEVPSMVVLSQYFIASITTSSCLSAIAIRIFETFADLDSMLLITTTQDFYNKSRYQTN
KYLIIILGVSHIISSTLDLLTDDEIVWCKFFVLPIYFLQKLEVLTFCKLIVMIQCRLQIINKYLTNFIEEQEKNKAL
VFTLAESNPKKTDKFNWIGCPSPNNMKIRDLATMYDVIGTICSLINDLFNIQIFMTLVSTFTYIVIAIWSTLYFYR
APNFTFGTLTTIIIWCITIILSVVVMSFVCERLVSVRNNTKILVNKVIMNYDLPKTMRVQAKAFMELIESWPLKIM
VYDMFSVDISLMLKFISVATTYLIVIIQLSHFV
>BmorGR54
MTHAALPRSEAYFLMTLSRSTIVSKRAYGPPDGEWLPSPMDFSNARGRAKPLPTVCLRVCVQNNLSFYRPILIILQ
LCGYDFDYYNINLVLNVLTKAYCASLTCVVVYATIACCSSIQLSHIWSLIEYGTSVVIIACFRSQTKLFLKQLTTL
DVYLRISNRRFVLEKCKIFTITSVIFLLRIVYTSIYCSTHHCFNVLIYFLLSQFALVCLDVNRIWRCIVFDAIRYR
LKTLRLRMEENPDCNYYLYVKNNKSIRKNKISFCLFLYRTIADLVDLVSPELNVSRSTTNICGLKTHIIRGAPKTA
DTSCTRYSSLPEKNQQCISILFLSVACSLPKIVSNAYHLLLIIEDREPLETGGYVLMHTLQVSLLLFTPFIIVECY
TMEVEKIKLYLVHRLIDENDTTMRDNIRLFLEYMSVRTFRYRIFRIVPVNATLPLELVNLCVNYVIVLINFTHLYG
>BmorGR55
MERINLLKSFAFLENVMCIYRNFMFYNQRARFIIIGRIVAELVFYIFSAYNGFLLVYTDWFSQNFSVFFIEIISKS

SFYVITFFTMVNGILKSREYKTFIFSINKIHDYILNDTDYLKRLKCTNIFCTATIIILFVVTLIRTAIDGSNYGQL
SGINARSVIWMLTTILLECQYQTECVVYFGFILFIHAIMKYLNIRVTNTIIKIARSDMAVKRIPKYIIGRTELKDE
TDTGVDVNNVVDLEEVRYWVFIYRQLGLTTELLQKCFGMQTAFIFVTAVLNQIITVFRVIAVFIYGSLANRGAEHS
IIANFLFTLLYRLPGLLMIIVGGQMVQNQTDMLRRSMARLNNIISNNPHRETFSALSDFHRMIVKNPVKIYVLSVL
PVGAYMLPLFMTLLINHIIILLQFNHVA
>BmorGR56
MKKIRLLRSIVFLENLLCIYRNFLFFNKKARAIILIHITIELVLYVLSIVNNSFIIYSYFHSDNRSMLIVFTTICC
FYVVTFVSIVMGILRSEEFKDLVTSLELINKFFTNNKTYLKSLGRSNTMIIAITTILYCVTCIGIAVDKITLNDFY
EFTSSDVIWTVSSTLLELRYQTECVVYFGIEYLFLIFTKHLNLLVKEAIKKVSLDNNGTVKDVPISSDAVTKNEVK
RWATIYRQLMMSSKLLQACFSLQIICVFVSAVINFITTAFRMVKVSVLGSIATDMNEIIIVNLIFTLLYQNIGLVL
IIVTGQRVWNQILLLNVLLARLYNGILIQPCRDTLRTLKNLQRMVVKNPVQIKMLSVLPVGSYMLPMFMTLSVSYI
IVMLQFGHVV
>BmorGR57
MEEIKAIKLVTFIENCICVYRNYAMCTKRNKKIISLRIIVEIIIVFFVNINNILLLHKYYNGSGLLYIIYLFLVVY
YINYMFCIFYGALQGKAYRQLIFCFNKINAIAKRDKSYKKSLARLKNMCIVISIALLIISALSVFVDRSNSWNIYE
VSLRDSLLILSKIHMDFFYHFEYVVYFTHIKIFHLTLRYLNSRVKMAQFEMKMTRRDVHDEGERNIRILLTKELTT
EWAVLYKCLVFGTKTMKSLFGLQMLIAMVMSFVNFTLSLYGIILICSIEQSQTASQHNLLLILTYYTATMLLIFIV
AQSVYNEVEMLKRNLARMYNILAVDSDETQQKLVKDFLRMVYKNKVEIKMLSIFPVGMPMLTFFLSLSASYVVVMV
QFSNVF
>BmorGR58
MSSRRVLYRAEVLLSNNVDAHVQDMLKPLNFFQFILFFPKYTIRDGYITPNSLIRNIWSATGAFVFISICVFRILT
MNKIAVYDTFTTMLLISKYFDVALYCIGFIVNTYVNIAYSNVNVLLYLKLQTIKTFIPRNNEIMKNVKWYSVILII
VLFCGTLAMFSFFHLSFSYFNIFDLTTDLAVFSFDLNLVYACSVLNFLAQSLDELNKEIWRLGNAKVTVCKDGSKP
DWNGINLTYINVLDAYNYFKEAFRLLIFFHTFKTLTHMFIYIQSIIELCKKFYPGDDYDAITVGAVVGVWFFRNIT
LQCLVGVSCQNFYSATSNTESICAVQVGSIVSDEHKLFLKAVRRLNNVVFYKWSMYGMFIVDATLPRRLIELIATY
TVVFLQFAFK
>BmorGR59
MPYKKDSNRCEVLLYNNVDTDLQDMLRPLNFIQTIYLSPKYTIKDGYITPNSLFCNILSAAGAIVFFSICVYRILT
ASKIGTFEGFSTTLLITKYFDAILFSLGFVANAYVSIRLSHLNVLLYLKLQAIKTFVPCKKIMQKVKYYSIVLIIG
LIIVRLIMYIHFHWSLGYLSYLDLITDLGVISFDLNLVYASSIVKFLGYNLEELNKEILRLDEIKATMDEEGSKPD
WNGIRRTYLKFSEAYNYFKDAFRILILFHTLNTFAHVFIYVQSVIELCKAPADNYMGGRAHSPPGVKWLLEPLDIY
NSAAFSVLIAVIVWLLRNIILQSLIGISCQSFYSATSNTQSICSILVRSVLSGEYNQWFLLKKYDVETHFFIAYVV
GRAHSPSGVKWLLEPIDIYNVNSTTHLEI
>BmorGR60
MLTPRSDLCNEKLSPSFPSGKTTAADKDDTEARCQVDSSLERLLLPFNLVQHVSFIPMYSIRRGLVSPDGPLAYLY
SLLGFCLFTSVSVYRNAIMHGTRLSSLHLFTLYSDLVSFVINYSLSLICNVVNSKSNVEFVCRLQRLQTVLRRNQR
EQEQFARSNWAHLAVVTALYLAVVGLLNVVVLKQSLPDTLYLLLLFCIDVNVLYATRMLALLRCYLQLWTRKINEK
AFNPVHHNMFTAYLDILQEYEVYTTLFKKIITYYVLETFLHGLLYVQVAIQICKSIRRSGRFSEQLMMIVSIFTWT
IKNMIIMTLHNVECEKFYLAVEQAVAACQTQRASTTRCREEKRLYKNVCRVSRAAFSRERGWGLLAAGAALTLRFM
DLATTYVTVLLQFAFVSRT
>BmorGR61
MSIRFEKDLLHNYVEIELQYFLRPFNVMQSLFFQSKYRIVDNFILPNTLFKNIMSFVVSVLCALSFIYTIISVWQN
THATSFHALVTSVYLSYNIYGILIGSVLIIWLSDRNIEFVLKIQDLIKILEFNKCFLIEYAFINSIIMAAIFILNF
LLYGYFVVHLQKFALGLTFSAIVCILNQDLDIIYVIIFANILKKCASRWTVEARQKNNFNDQGKWVKLFNAFLNLT
ESYQLYQKIFEFYELLRRVGIVFLGLQLTVCRVCSNDIKSIQCTVMLHAFQLICVWIVKKFITLSILSFEMEIFYE
KLREIETVCIILVSSDNPSERELKIWKNIIRVSSCSVRKTTACGLCEVGAALPQWLLQATTAYTIVLLQFHITTFS
RATNDIYDLD
>BmorGR62
MNDLFLSKIVKWTKTTKYKLDDDFQSLFRVFNIAQAMNLCPKFLIYDKYITNNAWFIHILAISSFIVLVCLDSFFA
NFRLVLSEAMGPPFYGFSFYFISILYENIGVIIQITMNGYLTKNNVLIITKLQDTFKDFRTTDYITKSNRWTNWFI
FFIYMNFIANYSYFNFYVNTFSFHKFCFAFIKMCFDLNIVYTIFIFKMIGDSLTMFKDTAFCSKNMKLYEVSNRVY
WNKMLRLYSNILDVFELSKRTLNFFIFYFVSNILLRILSHVQLAILMNSINWLQHVAYSNIVMVLLTLAKEGIILI
VLIAKCEKIYCVIGDVQTACQLALGNAACPEKRRFCKNVRRSSSAAFSKIYICNILAVDAKLAVSLMSVTTTYTIV
MLQAILIK
>BmorGR63
MQIGNAVIHLKSTKLTTMNTISPTTKLLKIFALNSNIEEIDLKCSTKLRITMTAFVLCSLIFYSLYYKFIYVFDYV
NISIKITDCVQMVYDFCQYIVDLYFVTNYGRNISSEYFQQYKIIDKILEVVCYEIIKHRIVKLLWVFMCIWFSSSC
FDFIAWFLNYGWITPLVYSVAYIFLLIKILTTLDLSAHIMNVEIRLKMIADLIHHYYMSCEDNFQAEETLCHKNWL
NSKERAKYYELQFRIHALKQLSCNNNEIKLLSRCYLMLTEQVEIINRMYGFRILLNSLSLLIDMVRFTNISVRIMI
GSQNLAYNCGYFPAVSSIFRLLTCGAVIINLVSHCERVYYQRTRICNVIDHMIVNKNLSRESTEALQEFRNLVQNH
PIEFNMANFFQLNYSLLVSIASVVVTYTIILLQSVN
>BmorGR64
MKISLRKIVSIRNMTLIQNMFGFYHKFTDNRAIGVLLKIFCGFYSLFLSFLCINCTPRFTNDFLTYDIFFFVIEYL
TSVLVCLLYDGQYFLNYLYDLKLIDREAGIEESLEKLPISQPLFSLIFITRVIYLLSCLLMFDGIKDSLFLPAQSS
VFGANFTEFARTIGYFPRVIMFEMFYKRVNYLKSQLRNDLAHANLYPIGFVCSKVIMKYINFYKLLLRNLQQNSLQ
FKILMSMSSLYIIIKALASAYAFIYREDGVHVFIFIEFATGVFLFFVMSSIIISIFNEIEDIRQIVLAQLRYCKQG
ANTKRVQDALTILNIRCFKYALCRIYTVDFTFILRILDVSVTYVIVLVQFTHILD
>BmorGR65

MKISLRKIVSIRNMTLMQNMFGFYHKFTDNRSIGVLLKIFCGFYSLFLSFLCINCIPRFTNDFLTYNIFFYVIEYL
TSVLVCLLYDGQYFLNYLYDLKLIDREAGIEESLEKLPISQPLFSLIFITRVIYLLSCLLMFDGIKDSLFLPAQSS
VFGANFTEFARTIGYFPRVIMFEMFYKRVNYLKSQLRNDLAHANLYPIGFVCSKVIMKYINFYKLLLRNLQQNSLQ
FKILMSMSALYIIIKALATAYAFIYCEDGIHVFIFIEFAIGVFLFFVMSSIIISIFNEIEDIRQIVLAQLRYCKQG
ANTKRVQDALTILNIRSFKYALCRIYTVDFTFILRILDVSVTYVIVLVQFTHVLD
>BmorGR67
MRERKKKFNKLLNTRNYNNIVEALLPSDSIRKISGVSVVYLAVNSENRIVTKFSFIGTIFFLFWYILYFYCTYKAH
SEDQTILRTIYNTKLKRYGDDFERIASIIYVTYSMWKVPFRMSGNQVFIQRIVDIDSAIENMGEAVDYNKNAKTAL
VISIAQLGDFLVRMFCIWLSLENLSVIVPTEKLYQVVYTDALSFVITSHYCFSLIVLRGRYKYINKVLSEIKTRSA
WEYKVFVRNKVAPDLEKVQRLQDRIVCEKIKACARIYSMLYKATEAINRMYGTALVLTMLLYLVFIILYMFYFMEA
TASGLLYDIKKYVDFLICVFWQMSHALSIIYANVYFSESITREVCKF
>BmorGR66
MKRKLKKFFPNKEYNNIVEATHLWKLIRKLTGLSVLTLESKEGNRIETRFSSLGFVFFLLWFTIYFYCTYKAHNED
QTILRNIYSTKLQRYGDDFERITSIIYVLYSMWKLPFQISGNRLLLQEIVDIDKAIESVGVTIDYKKNATFALFIY
IGQIATYLFRLFCVWGCLGNLNSPVPVEKLYQDIFTDALSLLLTSQYCFSLVILRDRCRYINKILCGIENRESSRL
RLFVYSSMPGAEKDITCRKIKDCSKIYGMIYKAVESTNITYGFALVLTMLLYLIFIILYMFYFMEATAAGLFLDTK
KYIDFLICVLSELLHAMLIIFLNIYFSEETVKETRTTSFVIHGIINSDFNTQAKTEAIHFSTQLLHQIPKFTASGL
381VELNYSLLYEVGGGILVVTP401MGSGNNLATDGPRVCSPI
>BmorGR68
MRFGLKAGAAVVTILRPYNLCLKNIFKPFYVMLSLLGLFPYSIRFLGGKQFLIKPKSIYTNAVCALSLMLSMTLFL
IFHIDHIIYKSTEDNSLTEGFMTQVNYIIEMLNLEIFCVVYYFSSFLNRNKFVKVLNTVAVWSDRISISGIKTLSF
LRLKIHFSIGILMFLLISQVCVNFTRVDSLWKKVLVMFTFNIPQMIQFTAILFYYILVNMVITLLVIIQENISIST
RDTKTSSFIRVEHRMPLSLKQLELIYIKAFELKRDINKAFEAPILLTTMQCFHSIVSESHIIYHGAVMEPHMVLHS
IMNCSVWILYQLFKLYILASTGHLLQEKIQHFSNLIHFHGKGLTVYGLFPLDGTLMFKVVASAAMYLIILVQFDKR
N
>SfruGR30
MSELSFDIYENITNMDQNITHYPPEATVLAAACACIFSVVGVVGNLVTTVALLMHPKLRGHVTTMFVLSLCVSDLL
FCSINLPLTANRYIKQNWSLGPSLCQMFAFVFYGNEAVSLLSMVAITINRYTLIAYYDMYSQIYTTTKIWVQLLLI
WLVSFGLMVPPLLGVWGQLGLDPSTFSCTILPKEGRSPKKCLFVVGFALPCVVIIVSYSCIYWRVRQSKRKLEGRR
CVCGKLSGQTAKEKEEDSRLTTLMLTIFLCFLACFLPLMIMNVADDGITYPWLHIIASILAWASSVINPLIYAATN
RQYRAAYANLLKFCKNSTVKKRTTWGSRTMANSSNSPHYADKRNHMKDKPADL
>SfruGR1
MNKEHGFRVYNPNPVNKETRKREMFQRIDEKDGIKEYDAKDLYGPEITDKDGALLDAHDSFYITTKSLLVLFQIMG
VMPIMRVPKHAQTTKRTTFNWISKATLWAYLVWGLECIIVVKVGRERLANFQNSSNKRFDEVIYNIIFLSILIPHF
LLPIASWRHGPQVAIFKNMWTHYQLKYLKITGTPIVFPNLYSLTWGLCVFSWGLSFAVILSQHYLQDDFELWHSFA
YYHIIAMLDGFCSLWYINCNAFGTASRGLAMNLHKALGAEHPALKVAQYRHLWVDLSHMMQQLGRAYSNMYGIYCM
VIFFTTTISLYGALSEILEHGLSYKEMGLFVIVGYCMTLLYIICNEAYHASRKVGLEFQVRLLNVNLGAIDRSTQR
EVEMFLVAISKNPPIMNLDGFTNINRELFTANVSFMSTYLIVLMQFKLTLLRQSARKTLKSIVKAVFNTSTTMLDD
DFDEEGEE
>SfruGR12
MKVYSLSSRSPEQPCHFHGCLRHALRVARWLGFFPVQGLGEIRPEGIRFKIKSFYSIYAVATLLGQVMMSYFALLL
FFQTEVTLSSISNIVFYVTSLITNILLLKLAQQWPYLMTKAVETERGLTELTLDHKVAGRSSVLAYLVMALAMVEH
ILCTSFNIKFVMYCLQEAGVTTNVMENYVVHRMPYIFNYIPYSFFSAIFFEYLCLQSTFLWSFNDVIITCFSIYIT
AYFKTLNVVIATNVKKDKDGIPWSILRVHYSNLVKLVKEIDDHISSLILLSFFTDLFYICLQLFNSLHRNHVSFKY
CDETQTNQALSSPFYLLYYLYSFVFLVLRALMLSLFASNVHCAALEPVYSVYDVPSSVYDNEVRRFQLQLHYTEVG
LSGKFFYVTRNMILKV
>SfruGR2
MRHIQQNKTVYEKTQRDYEQEQRDLLSSQDGDTCEIHDQFYRDHKLLLVLFRALAVMPITRSRPGTITFSWKSRAT
MYAVCFYIAATAVVLIVGYERIMILRSIRRFDDYIYAILFVIFLVPHFWIPFVGWGVAHQVAIYKTNWGKFQVRYY
RVTGENLQFPNLKTTIVIISVGCLLLAVCFLLSLCILMDGFLLIHTSAYYHIITMINMNCALWYINCKGIKIASQS
LSECFRRDVEQECSAQLISRYRYLWLNLSELLQSLGNAYARTYSTYCLFMFANITIAVYGALSEIVDHGFGFSFKE
MGLFVDAAYCSTLFFIFVDCSHKSTLTVAAGVQDTLLSIDVLAVDRPTQKEIDHFIQAIEMNPAVVSLKGYAHVNR
ELLTSAISMIAIYLIVLLQFKISLPKDPQTVGT

IRs/iGluRs:
>DmelGluRIIC
MWQRILLLGCMWSAFFMCRSRGQQINIGAFFYDDELELEKEFMTVVNAINGPESEQTMRFYPLIKRLKPEDGSVTM
QEHACDLIDNGVAAIFGPSSKAASDIVALVCNSTGIPHIEFDISDEGIQAEKPNHQMTLNLYPAQAILSKAYADIV
QNFGWRKFTIVYDADDARAAARLQDLLQLREVHNDVVRVRKFHKDDDFRVMWKSIRGERRVVLDCEPNMLVELLNS
STEFGLTGQYNHIFLTNLETYTDHLEELAADNETFAVNITAARLLVNPDPPPYSLPYGYVTQRDNIVYESSDPPRT
LIHDLIHDALQLFAQSWRNASFFYPDRMVVPRITCDFAASGGRTWAMGRYLARLMKGTSGVNNTNFRTSILQFDED
GQRITFNIEVYDPLDGIGIAIWDPRGQITQLNVDVKAQKKMIYRVATRIGPPYFSYNETARELNLTGNALYQGYAV
DLIDAIARHVGFEYVFVPVADQQYGKLDKETKQWNGIIGEIINNDAHMGICDLTITQARKTAVDFTVPFMQLGVSI
LAYKSPHVEKTLDAYLAPFGGEVWIWILISVFVMTFLKTIVARISKMDWENPHPCNRDPEVLENQWRIHNTGWLTV
ASIMTAGCDILPRSPQVRMFEATWWIFAIIIANSYTANLAAFLTSSKMEGSIANLKDLSAQKKVKFGTIYGGSTYN
LLADSNETVYRLAFNLMNNDDPSAYTKDNLEGVDRVRKNRGDYMFLMETTTLEYHREQNCDLRSVGEKFGEKHYAI
AVPFGAEYRSNLSVAILKLSERGELYDLKQKWWKNPNASCFEEPDPDATPDMTFEELRGIFYTLYAGILIAFLIGI
TEFLVYVQQVALEERLTFKDAFKKEIRFVLCVWNNRKPIVAGTPISSVRTTPRRSLDKSLDRTPKSSRRVVIGRSS

EEMREMAQGSGSSSGSNNAGRGEKEARV
>DmelGluRIIA
MRLCPVVIYAFIIIIGFLEGIIALGGDDRNEITVGAIFYENEKEIELSFDQAFREVNNMKFSELRFVTIKRYMPTN
DSFLLQQITCELISNGVAAIFGPSSKAASDIVAQIANATGIPHIEYDLKLEATRQEQLNHQMSINVAPSLSVLSRA
YFEIIKSNYEWRTFTLIYETPEGLARLQDLMNIQALNSDYVKLRNLADYADDYRILWKETDETFHEQRIILDCEPK
TLKELLKVSIDFKLQGPFRNWFLTHLDTHNSGLRDIYNEDFKANITSVRLKVVDANPFERKKTRLTKVDQILGNQT
MLPILIYDAVVLFASSARNVIAAMQPFHPPNRHCGSSSPWMLGAFIVNEMKTISEDDVEPHFKTENMKLDEYGQRI
HFNLEIYKPTVNEPMMVWTPDNGIKKRLLNLELESAGTTQDFSEQRKVYTVVTHYEEPYFMMKEDHENFRGREKYE
GYAVDLISKLSELMEFDYEFMIVNGNGKYNPETKQWDGIIRKLIDHHAQIGVCDLTITQMRRSVVDFTVPFMQLGI
SILHYKSPPEPKNQFAFLEPFAVEVWIYMIFAQLIMTLAFVFIARLSYREWLPPNPAIQDPDELENIWNVNNSTWL
MVGSIMQQGCDILPRGPHMRILTGMWWFFALMMLSTYTANLAAFLTSNKWQSSIKSLQDLIEQDKVHFGSMRGGST
SLFFSESNDTDYQRAWNQMKDFNPSAFTSTNKEGVARVRKEKGGYAFLMETTSLTYNIERNCDLTQIGEQIGEKHY
GLAVPLGSDYRTNLSVSILQLSERGELQKMKNKWWKNHNVTCDSYHEVDGDELSIIELGGVFLVLAGGVLIGVILG
IFEFLWNVQNVAVEERVTPWQAFKAELIFALKFWVRKKPMRISSSSDKSSSRRSSGSRRSSKEKSRSKTVS
>DmelGluRIIB
MHGLQFLVLLALAIASGANEDTLVIKIGAIFFDTEMKLADAFSAALEEVNAINPALKLDAIKRYVTVDDSIVLQDI
SCDLIGSGVAAIFGPSSKTNSDIVEVLCNMTGIPHLQFDWHPQQSNRERMNHQLTVNVAPMELFLSAAFSDILASK
TFDWKSFTIAYERSSHLIRLQHILAWKQLHKAGIKMQEFERGDDYRILWKRINNAREKFVLLDCPSDILVDVINAS
IGYNMTGSFNHLFLTNLDTHLSGIDGFYSRDFTVAVAAVRIRTYVPPPVHDEIDVFDNSVDTRFSSLGSQLVYDSI
VLFYNALLEISQRPGFYIPNFSCGRGFWQPGPRLVEQMKQITPKMVKPPFKTQRLQINADGQREDFNLEVYNPIID
RVTHIWNKEFQLVDFEKLRENSTQALKQKRLQNKEDFSQKPIRYTVATRVGKPYFSWREEPEGVHYEGNERFEGYA
VDLIYMLAQECKFDFNFEPVRDNKYGSYDANTDEWDGIIRQLIDNNAQIGICDLTITQARRSVVDFTVPFMQLGIS
ILSYKEPPPKADIYAFLNPYNAEVWLFVMIAMMITAFALIFTGRIDQYEWDQPVENVNREMERQNIWHLSNALWLV
LGSMLNQGCDLLPRGLPMRLLTAFWWIFALLISQTYIAKLAAFITSSKIAGDIGSLHDLVDQNKVQFGTIRGGATS
VYFSESNDTDNRMAWNKMLSFKPDAFTKNNEEGVDRVKLSKGTYAFLMETTNLQYYVQRNCELTQIGESFGEKHYG
IAVPLNADFRSNLSVGILRLSERGELFKLRNKWFNSNESTCDSNVPTIDDGQFDMDSVGGLFVVLIVGVVVGLVIG
VAEFLWHVQRISVKEKIPPMLALKAEFYFVIRFWLTRKPLHTYRQSRDSTSTGYSSLEQITSASSAKKKKKTRRIE
K
>DmelClumsy
MYSLFLTHFLLIALPVLADIDRSQFMVGSIFTSDKDESEIAFRTAVDRANILERNVELVPIVVYANTDDSFIMEKM
VCNLISQGVIAIFGPSTGSSSDIIASICDTLDIPHIVYDWIPNESIPDREHSTMTLNVHPDNLLLSQGLAEIVQSF
AWRSFTVVYETDKELQQLQDILQVGEPISNPTTVKQLGPGDDHRPFLKEIKLSTDNCLILHCAPDNLLKILQQANE
LKMLGEYQSVFIPLLDTHSIDFGELSGVEANITTVRLMDPSDFHVKNVVHDWEEREKREGRYFKVDPNRVKSQMIL
LNDAVWLFSKGLTELGIFEELTAPDLECRRKKPWPFGKRIIEFIKARSEETSTGRIDFNENGQRSFFTLRFMELNS
DGFLDLATWDPVNGLDVLNDDEESEKRVGQKLSNKTFIVSSRLGAPFLTLREPQEGEILTGNSRYEGYSIDLINEI
AKMLNFKFEFRMSPDGKYGALNKVTQTWDGIVRQLIDGNADLGICDLTMTSSRRQAVDFTPPFMTLGISILFSKPP
TPPTDLFSFLSPFSLDVWIYMGSAYLFISLLLFALARMAPDDWENPHPCKEPEEVENIWSIMNTTWLSIGSLMGQG
CDILPKAASTRLVTGMWWFFALMMLNSYTANLAAFLTNSRQANSINSAEDLAAQSKIKYGAMAGGSTMGFFRDSNF
STYQKMWTAMESASPSVFTKTNDEGVERVQKGKNLYAFLMESTTLEYNVERKCDLVQIGGWLDYKSYGIAMPFNSP
YRKQISAAVLKLGELGQLAELKRKWWKEMHGGGNCEKSDEDGGDTPELGLENVGGVFLVLGLGLLSAMVLGCTEFL
WNVKSVAIEEKISLKEAFKSEALFAARIWITTKPVHTSSESGSSNSSSSSSSRSKHSFKSQGLSMKSLKSSGYQDV
EASVHSKLKKIGSMFSLKSQKTVTPPPEIGWKLDKSTQIDVVPTSDVDQELIPEVEPHLPHRHHHHHHHRHHHHHH
QPDQEHDRNPSPPE
>DmelGluRIID
MHFCWISLIILSLSRVQAQFYGGNAYEASSGQSIRLGLITDDATDRIRQTFEHAISVVNNELGVPLVGETEQVAYG
NSVQAFAQLCRLMQSGVGAVFGPAARHTASHLLNACDSKDIPFIYPHLSWGSNPDGFNLHPSPEDIANALYDIVNQ
FEWSRFIFCYESAEYLKILDHLMTRYGIKGPVIKVMRYDLNLNGNYKSVLRRIRKSEDSRIVVVGSTTGVAELLRQ
AQQVGIMNEDYTYIIGNLNLHTFDLEEYKYSEANITGIRMFSPDQEEVRDLMEKLHQELGESEPVNSGSTFITMEM
ALTYDAVRVIAETTKHLPYQPQMLNCSERHDNVQPDGSTFRNYMRSLEIKEKTITGRIYFEGNVRKGFTFDVIELQ
TSGLVKVGTWEEGKDFEFQRPPQAVNFNDIDDGSLVNKTFIVLISVATKPYASLVESIDTLIGNNQFQGYGVDLIK
ELADKLGFNFTFRDGGNDYGSFNKTTNSTSGMLKEIVEGRADLAITDLTITSEREEVIDFSIPFMNLGIAILYVKP
QKAPPALFSFMDPFSSEVWLYLGIAYLGVSLCFFIIGRLSPIEWDNPYPCIEEPEELENQFTINNSLWFTTGALLQ
QGSEIAPKALSTRTISAIWWFFTLIMVSSYTANLAAFLTIENPTSPINSVKDLADNKDDVQYGAKRTGSTRNFFST
SEEPIYIKMNEYLNAHPEMLMENNQQGVDKVKSGTKYAFLMESTSIEFNTVRECNLTKVGDPLDEKGYGIAMVKNW
PYRDKFNKALLELQEQGVLARLKNKWWNEVGAGVCSAKSDDDGPSELGVDNLSGIYVVLVIGSIISIIISILCWCY
FVYKKAKNYEVPFCDALAEEFRIVIRFSENERPLKSAQSIYSRSRNSSQSIESLKTDSEENMPVED
>DmelGluRIIE
MFFNHFVILWSLFSIHISVNWAQYENFGGYDNYQSLESVPIGLLTDQNTEQMNIVFDHAIDVANQEVGTSLTSLKE
EVNYGDAYQSYGKLCRMLETGIAGVFGPSSRHTAVHLMSICDAMDIPHIYSYMSENAEGFNLHPHPADLAKALYSL
ITEFNWTRFIFLYESAEYLNILNELTTMLGKSGTVITVLRYDMQLNGNYKQVLRRVRKSVDNRIVVVGSSETMPEF
LNQAQQVGIINEDYKYIIGNLDFHSFDLEEYKYSEANITGLRLFSPEKMAVKELLMKLGYPTDQDEFRNGSCPITV
EMALTYDAVQLFAQTLKNLPFKPMPQNCSQRTESVRDDGSSFKNYMRTLRLTDRLLTGPIYFEGNVRKGYHLDVIE
LQPSGIVKVGTWDEDRQYRPQRLAPTTAQFDSVDNSLANKTFIILLSVPNKPYAQLVETYKQLEGNSQYEGYGVDL
IKELADKLGFNFTFVNGGNDYGSYNKSTNESTGMLREIMTGRADLAITDLTITSEREQALDFTIPFMNLGIAILYL
KPQKATPELFTFMDPFSEEVWWFLGFSFLGVSLSFFILGRLSPSEWDNPYPCIEEPEELENQFTLGNSIWFTTGAL
LQQGSEIGPKALSTRTVASFWWFFTLIVVSSYTANLAAFLTIEKPQSLINSVDDLADNKDGVVYGAKKTGSTRNFF
MTSAEERYKKMNKFMSENPQYLTEDNMEGVNRVKTNTHYAFLMESTSIEYNTKRECNLKKIGDALDEKGYGIAMRK
DWPHRGKFNNALLELQEQGVLEKMKNKWWNEVGTGICATKEDAPDATPLDMNNLEGVFFVLLVGSCCALLYGIISW

VLFVMKKAHHYRVPLRDALKEEFQFVIDFNNYVRVLKNSASIYSRSRQSSMSVASVAQESQ
>DmelCG3822
MRSSGVLVLPLLLLQLILNCRKAQSLPDIIKIGGLFHPADDHQELAFRQAVDRINADRSILPRSKLVAQIERISPF
DSFHAGKRVCGLLNIGVAAIFGPQSSHTASHVQSICDNMEIPHLENRWDYRLRRESCLVNLYPHPNTLSKAYVDIV
RHWGWKTFTIIYENNDGIVRLQELLKAHGMTPFPITVRQLSDSGDYRPLLKQIKNSAEAHIVLDCSTERIHEVLKQ
AQQIGMMSDYHSYLVTSLDLHTVNLDEFRYGGTNITGFRLINEKIVSDVVRQWSIDEKGLLRSANLTTVRSETALM
YDAVHLFAKALHDLDTSQQIDIHPISCDGQSTWQHGFSLINYMKIVEMKGLTNVIKFDHQGFRTDFMLDIVELTPA
GIRKIGTWNSTLPDGINFTRTFSQKQQEIEANLKNKTLVVTTILSNPYCMRKESAIPLSGNDQFEGYAVDLIHEIS
KSLGFNYKIQLVPDGSYGSLNKLTGEWNGMIRELLEQRADLAIADLTITFEREQAVDFTTPFMNLGVSILYRKPIK
QPPNLFSFLSPLSLDVWIYMATAYLGVSVLLFILAKFTPYEWPAYTDAHGEKVESQFTLLNCMWFAIGSLMQQGCD
FLPKALSTRMVAGIWWFFTLIMISSYTANLAAFLTVERMDSPIESAEDLAKQTRIKYGALKGGSTAAFFRDSKIST
YQRMWSFMESARPSVFTASNGEGVERVAKGKGSYAFLMESTSIEYVTERNCELTQVGGMLDTKSYGIATPPNSPYR
TAINSVILKLQEEGKLHILKTKWWKEKRGGGKCRVETSKSSSAANELGLANVGGVFVVLMGGMGVACVIAVCEFVW
KSRKVAVEERLSAILNE
>DmelCG5621
MISTEASFPLGFILTSLLLAFPGCRGERTNVGLVYENTDPDLEKIFHLAISKANEENEDLQLHGVSVSIEPGNSFE
TSKKLCKMLRQNLVAVFGPTSNLAARHAMSICDAKELPFLDTRWDFGAQLPTINLHPHPATLGVALRDMVVALGWE
SFTIIYESGEYLPTVRELLQMYGTAGPTVTVRRYELDLNGNYRNVLRRIRNADDFSFVVVGSMATLPEFFKQAQQV
GLVTSDYRYIIGNLDWHTMDLEPYQHAGTNITGLRLVSPDSEQVQEVAKALYESEEPFQNVSCPLTNSMALVYDGV
QLLAETYKHVNFRPVALSCNDDSAWDKGYTLVNYMKSLTLNGLTGPIRFDYEGLRTDFKLEVIELAVSGMQKIGQW
SGEDGFQENRPAPAHSLEPDMRSLVNKSFVVITAISEPYGMLKETSEKLEGNDQFEGFGIELIDELSKKLGFSYTW
RLQEDNKYGGIDPKTGEWNGMLREIIDSRADMGITDLTMTSERESGVDFTIPFMSLGIGILFRKPMKEPPKLFSFM
SPFSGEVWLWLGLAYMGVSISMFVLGRLSPAEWDNPYPCIEEPTELENQFSFANCLWFSIGALLQQGSELAPKAYS
TRAVAASWWFFTLILVSSYTANLAAFLTVESLVTPINDADDLSKNKGGVNYGAKIGGATFNFFKESNYPTYQRMYE
FMRDNPQYMTNTNQEGVDRVENSNYAFLMESTTIEYITERRCTLTQVGALLDEKGYGIAMRKNWPYRDTLSQAVLE
MQEQGLLTKMKTKWWQEKRGGGACSDADEDSGAVALEISNLGGVFLVMGVGSFFGIFVSLLEMVLGVKERSDENQE
APDSDASSLGFANLGGVYLVMFVGSCFGSIYGLVNCVVSVYLRARENKVSFKTELLDEIRFILQCSGNTKAVKYPK
NSSRSNASSKSKGSSMSVDSLPEDTSEADASGKHNHGKK
>DmelCG9935
MLIASGFLLFQFLSYGLGVPPLVRIGAIFSNQPGMYNSELAFRYAIHRLNMDKSLLPETTVDYYVEYVNRFDSFET
VQKVCKLIRVGVQAVFSPTDSVLATHINSICDALDIPNIGRSAHDFSINVYPSKQLVNYAFNDVIQYLNWTRFGIL
HEKENGIINLHQLSRSFHGEVHMRQVSRDSYVSALNEFKGKEIHNIIIDTNSNGISILLKNILQQQMNEYKYHYLF
TSFDLETYDLEDFKYNFVNITSFRLVDTADVGVKQILKDIGLYSHHIFKKPYLNLHIKKSTILESEPALMFDSVYV
FAIGLQTLEQSHSLTLLNISCEEENSWDGGLSLINYLNAVEWKGLTGPIQFKDGQRVQFKLDLIKLKQHSIVKVGE
WTPHGHLNITEPSMFFDAGSMNVTLVVITILETPYVMMHYGKNFTGNERFYGFCVDILETISREVGFDYILDLVPD
RKYGAKDPETGEWNGMVAQLMKYKADLAVGSMTITYARESVIDFTKPFMNLGISILFKVPTSEPTRLFSFMNPLAI
EIWIYVLIAYFLVSLCIYIVGKLSPIEWKCINACDLENISIGNQFSLTDSFWFTIGTFMQQSPDIYPRAMSTRIIS
STWGFFSLIIVASYTANLAAFLTTERMINPIENAEDLASQTEISYGTLDSGSTMTFFRDSVIETYKKIWRSMDNKK
PSAFTTTYEDGIKRVNQGNYAFLMESTMLDYIVQRDCNLTQIGGLLDTKGYGIATPKGSPWRDKISLAILELQERG
DIQMLYDKWWKNTDETCTRKNTSKQSKANSLGLESIGGVFVVLIAGIIVAAVVAFFEFWYNFRYNYEATPSQSVVN
NKYNQDGILESERNYTPPDRSFWIEIAEELRYASWCMNKQKRPALTRTCSKCTIPKGQRINKL
>DmelCG11155
MVRKKREIVIKENIQGRSYLKKICCSYIILSILVISNALPPVIRVGAIFTEDERESSIESAFKYAIYRINKEKTLL
PNTQLVYDIEYVPRDDSFRTTKKVCSQLEAGVQAIFGPTDALLASHVQSICEAYDIPHIEGRIDLEYNSKEFSINL
YPSHTLLTLAYRDIMVYLNWTKVAIIYEEDYGLFNLMHSSTETKAEMYIRQASPDSYRQVLRAIRQKEIYKIIVDT
NPSHIKSFFRSILQLQMNDHRYHYMFTTFDLETYDLEDFRYNSVNITAFRLVDVDSKRYLEVINQMQKLQHNGLDT
INGSPYIQTESALMFDSVYAFANGLHFLNLDNHQNFYIKNLSCTSDQTWNDGISLYNQINAAITDGLTGTVQFVEG
RRNIFKLDILKLKQEKIQKVGYWHPDDGVNISDPTAFYDSNIANITLVVMTREERPYVMVKEDKNLTGNLRFEGFC
IDLLKAIATQVGFQYKIELVPDNMYGVYIPETNSWNGIVQELMERRADLAVASMTINYARESVIDFTKPFMNLGIG
ILFKVPTSQPTRLFSFMNPLAIEIWLYVLAAYILVSFALFVMARFSPYEWKNPHPCYKETDIVENQFSISNSFWFI
TGTFLRQGSGLNPKATSTRIVGGCWFFFCLIIISSYTANLAAFLTVERMISPIESASDLAEQTEISYGTLEGGSTM
TFFRDSKIGIYQKMWRYMENRKTAVFVKTYEDGIKRVMEGSYAFLMESTMLDYAVQRDCNLTQIGGLLDSKGYGIA
TPKGSPWRDKISLAILELQEKGIIQILYDKWWKNTGDVCNRDDKSKESKANALGVENIGGVFVVLLCGLALAVVVA
IFEFCWNSRKNLNTENQSLCSEMAEELRFAMHCHGSKSRHRPRKRSCLNCSSVPTYVPSNVSTSNVGVYYNYFN
>DmelGlu-R1
MHSRLKFLAYLHFICASSIFWPEFSSAQQQQQTVSLTEKIPLGAIFEQGTDDVQSAFKYAMLNHNLNVSSRRFELQ
AYVDVINTADAFKLSRLICNQFSRGVYSMLGAVSPDSFDTLHSYSNTFQMPFVTPWFPEKVLAPSSGLLDFAISMR
PDYHQAIIDTIQYYGWQSIIYLYDSHDGLLRLQQIYQELKPGNETFRVQMVKRIANVTMAIEFLHTLEDLGRFSKK
RIVLDCPAEMAKEIIVQHVRDIKLGRRTYHYLLSGLVMDNHWPSDVVEFGAINITGFRIVDSNRRAVRDFHDSRKR
LEPSGQSQSQNAGGPNSLPAISAQAALMYDAVFVLVEAFNRILRKKPDQFRSNHLQRRSHGGSSSSSATGTNESSA
LLDCNTSKGWVTPWEQGEKISRVLRKVEIDGLSGEIRFDEDGRRINYTLHVVEMSVNSTLQQVAEWRDDAGLLPLH
SHNYASSSRSASASTGDYDRNHTYIVSSLLEEPYLSLKQYTYGESLVGNDRFEGYCKDLADMLAAQLGIKYEIRLV
QDGNYGAENQYAPGGWDGMVGELIRKEADIAISAMTITAERERVIDFSKPFMTLGISIMIKKPVKQTPGVFSFLNP
LSQEIWISVILSYVGVSFVLYFVTRFPPYEWRIVRRPQADSTAQQPPGIIGGATLSEPQAHVPPVPPNEFTMLNSF
WYSLAAFMQQGCDITPPSIAGRIAAAVWWFFTIILISSYTANLAAFLTVERMVAPIKTPEDLTMQTDVNYGTLLYG
STWEFFRRSQIGLHNKMWEYMNANQHHSVHTYDEGIRRVRQSKGKYALLVESPKNEYVNARPPCDTMKVGRNIDTK
GFGVATPIGSPLRKRLNEAVLTLKENGELLRIRNKWWFDKTECNLDQETSTPNELSLSNVAGIYYILIGGLLLAVI
VAIMEFFCRNKTPQLKSPGSNGSAGGVPGMLASSTYQRDSLSDAIMHSQAKLAMQASSEYDERLVGVELASNVRYQ

YSM
>DmelGlu-R1B
MRFGLKLSCLWPSFLLWLTWSSGGGGGSGVGVSAQPSLTEKIPLGAIFEQGTDEVQSAFKYAMLNHNLNVSSRRFE
LQAYVDVINTADAFKLSRLICNQFSRGVYSMLGAVSPDSFDTLHSYSNTFQMPFVTPWFPEKVLTPSSGFLDFALS
MRPDYHQAIIDTIQFYGWRKIIYLYDSHDGLLRLQQIYQGLRPGNESFQVELVKRISNVSMAIEFLHTLEQIGRFE
NKHIVLDCPTEMAKQILIQHVRDLRLGRRTYHYLLSGLVMDDRWESEIIEFGAINITGFRIVDTNRRLVREFYDSW
KRLDPQMSVGAGRESISAQAALMYDAVFVLVEAFNKILRKKPDQFRNNVQRRSQTLMVAQAAASTSSDGYNYSASG
GGGGNGGAGGGFAGSDSGGSGGMASRALDCNTAKGWVNAWEHGDKISRYLRKVEIEGLTGDIKFNDDGRRVNYTLH
VVEMTVNSAMVKVAEWNDDAGLQPLNAKYVRLRPHVEFEKNRTYIVTTVLEEPYIMLKQVAFGEKLHGNNRFEGYC
KDLADLLAKELGINYELRLVKDGNYGSEKSSAHGGWDGMVGELVRKEADIAIAAMTITAERERVIDFSKPFMSLGI
SIMIKKPVKQTPGVFSFMNPLSQEIWVSVIFSYIGVSIVLFFVSRFSPHEWRLVQQQPQQSQSPDPHAHHEQLANQ
QPPGIIGGAPLPAPPGPPTPGAQTAAGAAALQAALSAGSPGSGGSSSAVVNEFSVWNSFWFSLAAFMQQGCDLSPR
SVSGRIAAASWFFFTLILISSYTANLAAFLTVERMVTPINSPEDLAMQTEVQYGTLLHGSTWDFFRRSQIGLHNKM
WEYMNSRKHVFVPTYDEGIKRVRNSKGKYALLVESPKNEYVNAREPCDTMKVGRNLDTKGFGIATPLGSALKDPIN
LAVLTLKENGELIKLRNKWWYEKAECSTHKDGETSHSELSLSNVAGIFYILIGGLLVSVFVAILEYCFRSRDSRSA
SSGSGMGLGMGLGGGMSGGSLGKANGSMMLGPSSAVPGGMPSSHQRSTLTDTMHAKAKLTIQASRDYDNGRVGYLN
CASLQYYPPAQLSATPPDAGDSLHMNAHGQV
>DmelNmdar2
MMPSRVKLKRGTDGPTPTPTPMPTTMRKHTPIATLNTASCQHNSTTSRRKRILTPPSGPISLLLLTVLTLLILDTR
SCQGLRLTNGGGSLSKGAAANKEQLNIGLIAPHTNFGKREYLRSINNAVTGLTKTRGAKLTFLKDYSFEQKNIHFD
MMSLTPSPTAILSTLCKEFLRVNVSAILYMMNNEQFGHSTASAQYFLQLAGYLGIPVISWNADNSGLERRASQSTL
QLQLAPSIEHQSAAMLSILERYKWHQFSVVTSQIAGHDDFVQAVRERVAEMQEHFKFTILNSIVVTRTSDLMELVN
SEARVMLLYATQTEAITILRAAEEMKLTGENYVWVVSQSVIEKKDAHSQFPVGMLGVHFDTSSAALMNEISNAIKI
YSYGVEAYLTDPANRDRRLTTQSLSCEDEGRGRWDNGEIFFKYLRNVSIEGDLNKPNIEFTADGDLRSAELKIMNL
RPSANNKNLVWEEIGVWKSWETQKLDIRDIAWPGNSHAPPQGVPEKFHLKITFLEEAPYINLSPADPVSGKCLMDR
GVLCRVAADHEMAADIDVGQAHRNESFYQCCSGFCIDLLEKFAEELGFTYELVRVEDGKWGTLENGKWNGLIADLV
NRKTDMVLTSLMINTEREAVVDFSEPFMETGIAIVVAKRTGIISPTAFLEPFDTASWMLVGIVAIQAATFMIFLFE
WLSPSGYDMKLYLQNTNVTPYRFSLFRTYWLVWAVLFQAAVHVDSPRGFTSRFMTNVWALFAVVFLAIYTANLAAF
MITREEFHEFSGLNDSRLVHPFSHKPSFKFGTIPYSHTDSTIHKYFNVMHNYMRQYNKTSVADGVAAVLNGNLDSF
IYDGTVLDYLVAQDEDCRLMTVGSWYAMTGYGLAFSRNSKYVQMFNKRLLEFRANGDLERLRRYWMTGTCRPGKQE
HKSSDPLALEQFLSAFLLLMAGILLAALLLLLEHVYFKYIRKRLAKKDGGHCCALISLSMGKSLTFRGAVFEATEI
LKKHRCNDPICDTHLWKVKHELDMSRLRVRQLEKVMDKHGIKAPQLRLASSSDLLNHHHLKERPPLLGNLSLAASA
QDLYRWSYKTEIAEMETVL
>DmelNmdar1
MAMAEFVFCRPLFGLAIVLLVAPIDAAQRHTASDNPSTYNIGGVLSNSDSEEHFSTTIKHLNFDQQYVPRKVTYYD
KTIRMDKNPIKTVFNVCDKLIENRVYAVVVSHEQTSGDLSPAAVSYTSGFYSIPVIGISSRDAAFSDKNIHVSFLR
TVPPYYHQADVWLEMLSHFAYTKVIIIHSSDTDGRAILGRFQTTSQTYYDDVDVRATVELIVEFEPKLESFTEHLI
DMKTAQSRVYLMYASTEDAQVIFRDAGEYNMTGEGHVWIVTEQALFSNNTPDGVLGLQLEHAHSDKGHIRDSVYVL
ASAIKEMISNETIAEAPKDCGDSAVNWESGKRLFQYLKSRNITGETGQVAFDDNGDRIYAGYDVINIREQQKKHVV
GKFSYDSMRAKMRMRINDSEIIWPGKQRRKPEGIMIPTHLRLLTIEEKPFVYVRRMGDDEFRCEPDERPCPLFNNS
DATANEFCCRGYCIDLLIELSKRINFTYDLALSPDGQFGHYILRNNTGAMTLRKEWTGLIGELVNERADMIVAPLT
INPERAEYIEFSKPFKYQGITILEKKPSRSSTLVSFLQPFSNTLWILVMVSVHVVALVLYLLDRFSPFGRFKLSHS
DSNEEKALNLSSAVWFAWGVLLNSGIGEGTPRSFSARVLGMVWAGFAMIIVASYTANLAAFLVLERPKTKLSGIND
ARLRNTMENLTCATVKGSSVDMYFRRQVELSNMYRTMEANNYATAEQAIQDVKKGKLMAFIWDSSRLEYEASKDCE
LVTAGELFGRSGYGIGLQKGSPWTDAVTLAILEFHESGFMEKLDKQWIFHGHVQQNCELFEKTPNTLGLKNMAGVF
ILVGVGIAGGVGLIIIEVIYKKHQVKKQKRLDIARHAADKWRGTIEKRKTIRASLAMQRQYNVGLNSTHAPGTISL
AVDKRRYPRLGQRLGPERAWPGDAADVLRIRRPYELGNPGQSPKVMAANQPGMPMPMLGKTRPQQSVLPPRYSPGY
TSDVSHLVV
>DmelIR8a
MELPLLVLLLALRFAGSEVLKITFWIEPVQRAEFDTDIAMVLKELDALRLDVKVDDTTLTLTRSEDGLDMQRFCEI
LSTVGASAVIDLTYSHWEEGYNLVRSLGIGYVRLERIMRPFLDMFGDFMRQKRANNVAMVFMNARDAVEAMQQMLV
GYPFRTLIMDASQTDPGQHFLERIRSLRPAPTYIALFARAAAMNGIFEKVQKADLFQRPLEWHFVFLDTRDRVFKY
RRQAELCTRFTLNPRAICRSMPMPDLYCGSGFTMQRAMLLNVLRSLINAAQVSPGYPLAIYQDCNATASSSEVSDP
LEKDDYNWLDMVHWSNFLAYAPPLPHIQDQFQSPVPGLTFAVNISAGYYSSEHEAKTDLAAWSSVGEMRLLNETIS
PARRFFRIGTAESIPWSYLRREEGTGELIRDRSGLPIWEGYCIDFIIRLSQKLNFEFEIVAPEVGHMGELNELGEW
DGVVGDLVRGETDFAIAALKMYSEREEVIDFLPPYYEQTGISIAIRKPVRRTSLFKFMTVLRLEVWLSIVAALVGT
AIMIWFMDKYSPYSSRNNRQAYPYACREFTLRESFWFALTSFTPQGGGEAPKAISGRMLVAAYWLFVVLMLATFTA
NLAAFLTVERMQTPVQSLEQLARQSRINYTVVKDSDTHQYFVNMKFAEDTLYRMWKELALNASKDFKKFRIWDYPI
KEQYGHILLAINSSQPVADAKEGFANVDAHENADYAFIHDSAEIKYEITRNCNLTEVGEVFAEQPYAVAVQQGSHL
GDELSYAILELQKDRFFEELKAKYWNQSNLPNCPLSEDQEGITLESLGGVFIATLFGLVLAMMTLGMEVLYYKKKQ
NALEITQVRPVNDSSGSGGNSSTAPPTATSTTKQAWHIPVLEAEEKPAKVSPPPSFETATFRGKKLPARITLGDGK
FKPRHGLYARRNLGASDSHSGYME
>DmelIR25a
MILMNPKTSKILWLLGFLSLLSSFSLEIAAQTTQNINVLFINEVDNEPAAKAVEVVLTYLKKNIRYGLSVQLDSIE
ANKSDAKVLLEAICNKYATSIEKKQTPHLILDTTKSGIASETVKSFTQALGLPTISASYGQQGDLRQWRDLDEAKQ
KYLLQVMPPADIIPEAIRSIVIHMNITNAAILYDDSFVMDHKYKSLLQNIQTRHVITAIAKDGKREREEQIEKLRN
LDINNFFILGTLQSIRMVLESVKPAYFERNFAWHAITQNEGEISSQRDNATIMFMKPMAYTQYRDRLGLLRTTYNL
NEEPQLSSAFYFDLALRSFLTIKEMLQSGAWPKDMEYLNCDDFQGGNTPQRNLDLRDYFTKITEPTSYGTFDLVTQ

STQPFNGHSFMKFEMDINVLQIRGGSSVNSKSIGKWISGLNSELIVKDEEQMKNLTADTVYRIFTVVQAPFIMRDE
TAPKGYKGYCIDLINEIAAIVHFDYTIQEVEDGKFGNMDENGQWNGIVKKLMDKQADIGLGSMSVMAEREIVIDFT
VPYYDLVGITIMMQRPSSPSSLFKFLTVLETNVWLCILAAYFFTSFLMWIFDRWSPYSYQNNREKYKDDEEKREFN
LKECLWFCMTSLTPQGGGEAPKNLSGRLVAATWWLFGFIIIASYTANLAAFLTVSRLDTPVESLDDLAKQYKILYA
PLNGSSAMTYFERMSNIEQMFYEIWKDLSLNDSLTAVERSKLAVWDYPVSDKYTKMWQAMQEAKLPATLDEAVARV
RNSTAATGFAFLGDATDIRYLQLTNCDLQVVGEEFSRKPYAIAVQQGSHLKDQFNNAILTLLNKRQLEKLKEKWWK
NDEALAKCDKPEDQSDGISIQNIGGVFIVIFVGIGMACITLVFEYWWYRYRKNPRIIDVAEANAERSNAADHPGKL
VDGVILGHSGEKFEKSKAALRPRFNQYPATFKPRF
>DmelIR21a
MSYYWVALVLFTAQAFSIEGDRSASYQEKCISRRLINHYQLNKEIFGVGMCDGNNENEFRQKRRIVPTFQGNPRPR
GELLASKFHVNSYNFEQTNSLVGLVNKIAQEYLNKCPPVIYYDSFVEKSDGLILENLFKTIPITFYHGEINADYEA
KNKRFTSHIDCNCKSYILFLSDPLMTRKILGPQTESRVVLVSRSTQWRLRDFLSSELSSNIVNLLVIGESLMADPM
RERPYVLYTHKLYADGLGSNTPVVLTSWIKGALSRPHINLFPSKFQFGFAGHRFQISAANQPPFIFRIRTLDSSGM
GQLRWDGVEFRLLTMISKRLNFSIDITETPTRSNTRGVVDTIQEQIIERTVDIGMSGIYITQERLMDSAMSVGHSP
DCAAFITLASKALPKYRAIMGPFQWPVWVALICVYLGGIFPIVFTDRLTLSHLMGNWGEVENMFWYVFGMFTNAFS
FTGKYSWSNTRKNSTRLLIGAYWLFTIIITSCYTGSIIAFVTLPAFPDTVDSVLDLLGLFFRVGTLNNGGWETWFQ
NSTHIPTSRLYKKMEFVGSVDEGIGNVTQSFFWNYAFLGSKAQLEYLVQSNFSDENISRRSALHLSEECFALFQIG
FLFPRESVYKIKIDSMILLAQQSGLIAKINNEVSWVMQRSSSGRLLQASSSNSLREIIQEERQLTTADTEGMFLLM
ALGYFLGATALVSEIVGGITNKCRQIIKRSRKSAASSWSSASSGSMLRTNAEQLSHDKRKANRREAAEVAQKMSFG
MRELNLTRATLREIYGSYGAPETDHGQLDIVHTEFPNSSAKLNNIEDEESREALESLQRLDEFMDQMDNDGNPSSH
TFRI
DN
>DmelIR31a
MNLLISMFILILAAGEGEIIPSMEESVVTNFVKSLVKTKQAIVFSCLFKDFKEISLALMRINQFVSVVNLNQSYSL
TSILTRENYARTSVMVNARCSGSSELLFEASENRYFNKTYQWFLWGVDLEVQSLFPLNLNYVGPNAQITYVNETAD
GYAYWDIHSKGRHLKSNLEINLIATLINDTLNIARDIFHLQSIDFRGQFNGLTLRGASVIDKEDIISNEQIESILS
RPTKDAGVAAFIKYHYELLGLLRERFNFTVNFRNSRGWAGRLGNTTFRLGLLGIVMRNEADIAASGAFNRINRFAE
FDTIHQSWKFETAFLYRYTSDLDTHGKSGNFLSPFSDRVWLFCLLTLGAFSIIWVLFEIIDYKILRIRVNSQKLEH
LNQKSSVICIKTTCIERILQTFGACCQQGLDPNPVDRSVRFLVMTLFLFSLVMYNYYTSSVVGGLLSSSDQGPSTV
DEITASPLKISFEDIGYYKVLFRESQNRSITRLIEKKLSSSRSLNELPIFSHIEDAVPYLKAGGFAFHCEVVDAYP
VISEYFDANEICDLREVSGLMEVEILNWILHKNSQYTEIFKTAMCNAQEKGFVERILRRRQIKKPACQSLYTVYPV
SLSGVLPGFVILICKSINKFS
>DmelIR40a
MHKFLALGLLPYLLGLLNSTRLTFIGNDESDTAIALTQIVRGLQQSSLAILALPSLALSDGVCQKERNVYLDDFLQ
RLHRSNYKSVVFSQTELFFQHIEENLQGANECISLILDEPNQLLNSLHDRHLGHRLSLFIFYWGARWPPSSRVIRF
REPLRVVVVTRPRKKAFRIYYNQARPCSDSQLQLVNWYDGDNLGLQRIPLLPTALSVYANFKGRTFRVPVFHSPPW
FWVTYCNNSFEEDEEFNSLDSIEKRKVRVTGGRDHRLLMLLSKHMNFRFKYIEAPGRTQGSMRSEDGKDSNDSFTG
GIGLLQSGQQADFFLGDVGLSWERRKAIEFSFFTLADSGAFATHAPRRLNEALAIMRPFKQDIWPHLILTIIFSGP
IFYGIIALPYIWRRRWANSDVEHLGELYIHMTYLKEITPRLLKLKPRTVLSAHQMPHQLFQKCIWFTLRLFLKQSC
NELHNGYRAKFLTIVYWIAATYVLADVYSAQLTSQFARPAREPPINTLQRLQAAMIHDGYRLYVEKESSSLEMLEN
GTELFRQLYALMRQQVINDPQGFFIDSVEAGIKLIAEGGEDKAVLGGRETLFFNVQQYGSNNFQLSQKLYTRYSAV
AVQIGCPFLGSLNNVLMQLFESGILDKMTAAEYAKQYQEVEATRIYKGSVQAKNSEAYSRTESYDSTVISPLNLRM
LQGAFIALGVGSLAAAALNNTINVRSLNSRDKFICGGPVKIWYYLVLLLWYYFNRGLVGIYQLWHKTSIRNTGKGM
PFLGE
>DmelIR64a
MHWWLLVFLPLSCQGLPEHELLELELDYGLAEPQRTSLLQSSLILQFSQDYKHIPRITYFTCQKPHLQTPNQIPNA
AEHRDAFAAKNFQLIKSLYESELFVRIVLLDVLAQSPTSGRPNRPGNGPTGGFSQTPSQAQSNSEWLEGVLRMEAL
RQIAVVDLACGAVSRRFLELASAKMLYSEKFHWLLIEDFAWHGRTQTAEGSGKRDDGEMEEEEPPGQQIQATDDED
LPSIESFLGGMNLYMNTELTLAKRMSEAAHYTLFDVWNPGLNYGGHVNLTEIGSFTPTEGIQLHTWFRTTSTVRRR
MDMQHARVRCMVVVTNKNMTGTLMYYLTHTMSGHIDTMNRFNFNLLMAVRDMFNWTFVLSRTTSWGYVKNGRFDGM
IGALIRNETDIGGAPIFYWLERHKWIDVAGRSWSSRPCFIFRHPRSTQKDRIVFLQPFTNDVWILIVGCGVLTVFI
LWFLTTIEWKLVPHDGSALIKPKGGAPPRHHYQQQQQQEQVEAPVRPITAVSVVVSKEKVEEKQEEYEDSTPIDAG
TLWQRCYQKLNKYIKDRKAKQKKAPERVGLFLESVLFFVGIICQQGLGFSTSFVSGRCIVITSLLFSFCIYQFYSA
SIVGTLLMEKPKTIKTLSDLVHSSLKVGMEDILYNRDYFLHTKDPVSMELYAKKITSVPTTKENEADEDEPVDPNP
VSTDPAKSYRDIVHSHETGAHAKDNAASNWLDPETGLLRVKHERFAFHVDVAAAYKIIAETFSEQDICDLTEVSMF
PPQKTVSIMQKNSPMRKVISYGLRRVTETGILTYHFNVWHSRKPPCVKKIETSDLHVDMDTVSSALLILLFSYAIT
LMILGTEILYSKWHNRIQLKWVGAT
>DmelIR75a
MQLVQLANFVLDNLVQSRIGFIVLFHCWQSDESLKFAQQFMKPIHPILVYHQFVQMRGVLNWSHLELSYMGHTQPT
LAIYVDIKCDQTQDLLEEASREQIYNQHYHWLLVGNQSKLEFYDLFGLFNISIDADVSYVKEQIQDNNDSVAYAVH
DVYNNGKIIGGQLNVTGSHEMSCDPFVCRRTRHLSSLQKRSKYGNREQLTDVVLRVATVVTQRPLTLSDDELIRFL
SQENDTHIDSLARFGFHLTLILRDLLHCKMKFIFSDSWSKSDVVGGSVGAVVDQTADLTATPSLATEGRLKYLSAI
IETGFFRSVCIFRTPHNAGLRGDVFLQPFSPLVWYLFGGVLSLIGVLLWITFYMECKRMQKRWRLDYLPSLLSTFL
ISFGAACIQSSSLIPRSAGGRLIYFALFLISFIMYNYYTSVVVSSLLSSPVKSKIKTMRQLAESSLTVGLEPLPFT
KSYLNYSRLPEIHLFIKRKIESQTQNPELWLPAEQGVLRVRDNPGYVYVFETSSGYAYVERYFTAQEICDLNEVLF
RPEQLFYTHLHRNSTYKELFRLRFLRILETGVYRKQRSYWVHMKLHCVAQNFVITVGMEYVAPLLLMLICADILVV
VILLVELAWKRFFTRHLTFHP
>DmelIR75b

MNFSVLESHFKEAQIFVDADVTYVTHDPFSKNFLLYDVYNKGRQLGGELNITADREIFCNKTNCRVERYLSELYTR
SALQHRKSFTGLTMRATAVVTALPLNVSIKEIFDFMNSKYRIQLDTYARLGYQARQPLRDMLDCKFKYIFRDRWSD
GNATGGMIGDLILDKADLAIAPFIYSFDRALFLQPITKFSVFREICMFRNPRSVSAGLSATEFLQPFSGGVWLTFA
LLLLLAGCLLWVTFILERRKQWKPSLLTSCLLSFGAGCIQGAWLTPRSMGGRMAFFALMVTSYLMYNYYTSIVVSK
LLGQPIKSNIRTLQQLADSNLDVGIEPTVYTRIYVETSEEPDVRDLYRKKVLGSKRSPDKIWIPTEAGVLSVRDQE
GFVYITGVATGYEFVRKHFLAHQICELNEIPLRDASHTHTVLAKRSPYAELIKLSELRMLETGVHFKHERSWMETK
LHCYQHNHTVAVGLEYAAPLFIILLGAIILCMGILGLEVIWHRHCTLH
>DmelIR75c
MTSWPLYRLIVFNLLEINLSNLMVFHCWSIKEAFPLVEMLNQNGIFSQYIDVQNPDNLANVHKEYLDSDLVSLNAD
VTYVSREDEERFILHDVYNKGSHLGGKLNITVDQTLQCNRSHCQVKEYLSELHLRPRLQHRMDLSSVTFRLAALVS
VLPINSSEEELLEFLNSDRDSHMDSISRIGNRLIMHTQEILGFNVQDAFGGAIGMLTNESAELCTTPFVPSWNRLH
YLHPMTEQAQFRAVCMFRTPHNAGIKAAVFLEPFMPSVWFAFAGLLIFAGVLLWMIFHLERHWMQRCLDFIPSLLS
SCLISFGAACIQGSYLMPKSAGGRLAFIAVMLTSFLMYNYYTSIVVSTLLGSPVRSNIRTIQQLADSSLDVGFDTV
PFTKTYLVSSPRPDIRSLYKQKVESKRDPNSVWLSPEEGVIRVRDQPGFVYTSEASFMYHFVEKHYLPREISDLNE
IILRPESAVYGMVHLNSTYRQLLTQLQVRMLETGITSKQSRFFSKTKLHTFSNSFVIQVGMEYAAPLFISLLVAYF
LALLILILEICWARYAKKKFSTIIPQNQ
>DmelIR75d
MKVQVAHWLPLIFFLLVSGTPRVAGSWRSEYSRQDPDPKTRWGNQLPDMLVAYYRHHGVHSLMLVVCHTDIADFRL
WKLWQHFNLNNFYVQVSTESSLRDLQHVDALDEHKDAPPPKSFHANNSTHWETSFLLPALPYKMGILLLEFSSECA
LNLLRWSAASEHNYFTTNRFWLLLTEDPGDIDLLEDPEIFIPPDSELRVLHYENVGNFSCSLIDLYKVAAWKPLKR
TLVGHNIRNSRHVIHALQHFGSAITYRQDLEGIVFNSAIVIAFPDLFTNIEDLSLRHIDTISKVNHRLMLELANRL
NMSYNTYQTVNYGWRQPNGSFDGLMGRFQRYELDLAQLAIFMRLDRIALVDFVAETYRVRAGIMFRQPPLSAVANI
FAMPFENDVWVSILMLLIITTVVLVLELFFSPHNHDMSYMDTLNFVWGAMCQQGFYVEVRNRSARIIVFTTFVAAL
FLFTSFSANIVALLQSPSDAIQSLSDLGQSPLEIGVQDTQYNKIYFTESTDPVTKNLYHKKIASKGENIYMRPLLG
MEKMRTGLFAYQVELQAGYQIVSDTFSEPEKCGLMELEPFQLPMLAIPTRKNFPYKELIRRQLRWQREVSLVNREE
RKWIPQKPKCEGGVGGFVSIGITECRYALGIFGCGAAVSFVLFLFEFIFRHFKQVYRIIKGYREVQR
>DmelIR76a
MENLLVESYYFSTVLSFFAQQFFADSHATCIFWHPAFDFRLETVHPMPLIIMDWHRWANRSDQDVYDYKIKEDEFE
GKGIPYNDWTLRLTVAIERSHCETFIAFQEQIPEFARYFYHASIYSIWRSLRNRFMFVYTKEFEDKKDSYLSGYIF
QDQPNILVITSQYLNSSTFEIKTNRFVGPRNFNKNPEPVEFYILQRFDAKGTKATWETQSAMSSKMRNLKGREVVI
GIFDYKPFMLLDYEKPPLYYDRFMNTTDVTIDGTDIQLMLIFCELYNCTIQVDTSEPYDWGDIYLNASGYGLVGMI
LDRRNDYGVGGMYLWYEAYEYMDMTHFLGRSGVTCLVPAPNRLISWTLLLRPFQFVLWMCVMLCLLLESLALGITR
RWEHSSVAAGNSWISSLRFGCISTLKLFVNQSTNYVTSSYALRTVLVASYMIDIILTTVYSGGLAAILTLPTLEEA
ADSRQRLFDHKLIWTGTSQAWITTIDERSADPVLLGLMEHYRVYDANLISAFSHTEQMGFVVERLQFGHLGNTELI
ENDALKRLKLMVDDIYFAFTVAFVPRLWPHLNAYNDFILAWHSSGFDKFWEWKIAAEYMNAHRQNRIVASEKTNLD
IGPVKLGIDNFIGLILLWCFGMICSLLTFLGELWRGQG
>DmelIR76b
MATGIELLVAAALCVACPPLNDSPPTNLIQMGENGTLSPVTELPMDVDASEAGFDADAPVETLETINRKKPKLREM
LDWIGGKHLRIATLEDFPLSYTEVLENGTRVGHGVSFQIIDFLKKKFNFTYEVVVPQDNIIGSPSDFDRSLIEMVN
SSTVDLAAAFIPSLSDQRSFVYYSTTTLDEGEWIMVMQRPRESASGSGLLAPFEFWVWILILVSLLAVGPIIYALI
ILRNRLTGDGQQTPYSLGHCAWFVYGALMKQGSTLSPIADSTRLLFATWWIFITILTSFYTANLTAFLTLSKFTLP
YNTVNDILTKNKHFVSMRGGGVEYAIRTTNESLSMLNRMIQNNYAVFSDETNDTYNLQNYVEKNGYVFVRDRPAIN
IMLYRDYLYRKTVSFSDEKVHCPFAMAKEPFLKKKRTFAYPIGSNLSQLFDPELLHLVESGIVKHLSKRNLPSAEI
CPQDLGGTERQLRNGDLMMTYYIMLAGFATALAVFSTELMFRYVNSRQEANKWARHGIGRTPNGQSVAPSRWLRGW
RRLNSGHGQLLGASTHGQNVTPPPPYQSIFNGGSHGDPLNRWRRPLANGNALGNGVLLGGDSEGGVRRLINGRDYM
VFRNPNGQSQLVPVRSPSAALFQYSYTE
>DmelIR84a
MIKLQVKVISWPLIILTAFLRVLQIESINTNFLELAAFEDFLRSEHLSHVLVVRGDDADGDWKIECHQKLLANYRV
QFYRPEMSANFEDLMFYGSPRTAVLVLNSEHVLVRRQVFGVASEAGYFNNSLAWFILGSGRESLPVEQLIDQLLSG
YRMGIDADITVALRGPDNASMLFYDVYRISRQANTPLIIEKKGLWTHSGGYQKFGNFKNTWVIRRRNFLNVTLIGS
TVLTEKPPGFGDMEYLADDKQLQQLDPMQRKTYQLFQLVERMFNLSLAISLTDKWGELLDNGSWSGVMGQVTSREA
DFAVCPIRFVLDRQPYVQYSAVLHTQNIHFLFRHPRRSHIKNIFFEPLSNQVWWCVLALVTGSTILLLFHVRLERM
LSNMENRFSFVWFTMLETYLQQGPANEIFRLFSTRLLISLSCIFSFMLMQFYGAFIVGSLLSESARSIVNLQALYD
SNLAIGMENISYNFPIFTNTSNQLVRDVYVKKICKSGEHNIMSLQQGAERIIQGRFAFHTAIDRMYRLLLELQMDE
AEFCDLQEVMFNLPYDSGSVMPKGSPWREHLAHALLHFRATGLLQYNDKKWMVRRPDCSLFKTSQAEVDLEHFAPA
LFALALAMVASALVFLLELFLHWLPDFRRRLGTMST
>DmelIR92a
MLLQPLVMHLSQLLRIIVGQYFAEFPSILIVYNNSASTTPLQLEYLSALELVLRELSKPIRLQWINVAFLKDLNDL
EDQVMGALNSSVTEGFITILSQTHHFIHARYYATRNANVRLKDKRYLFLCEDESPAELLCMDILQFYPHHLMVRPG
TETAPTGPTGPHPDPRRGGGASVSTKNKDDGEGGAGNKTTSPYRDINFELWTQKFVGAVGNLDALLLDAFLPNETF
ANRVELYPNKLLNLQRRSLLVGSITYVPYTITNYVPAGQGDVDPIHPQWPNRSLTFDGAEANVMKTFCQVHNCHLR
VEAYGADNWGGIYDNESSDGMLGDIYEQRVEMAIGCIYNWYDGITETSHTIARSSVTILGPAPAPLPSWRTNIMPF
NNRAWLVLISTLVICGTFLYFMKYVSYRLRYSGTQVKFHHSRKLEKSMLDIFALFIQQPSAPLSFDRFAPRFFLAT
ILCATITLENIYSGQLKSMLTFPFYSAPVDTIEKWAQSGWKWSAPSIIWVHTVQSSDLETEQILARNFEVHDYSYL
SNVSFMPNYGFGIERLSSGSLSVGDYVSTEALENRIVLHDDLYFDYTRAVSIRGWILMPELNKHIRTCQETGLYFH
WELEFIDKYMDKKKQEVLMDLANGHKVKGAPQALDVRNIAGALFVLAFGVAFAGCALVAELLIHRMDLSK
>DmelIR93a
MNPGEMRPSACLLLLAGLQLSILVPTEANDFSSFLSANASLAVVVDHEYMTVHGENILAHFEKILSDVIRENLRNG

GINVKYFSWNAVRLKKDFLAAITVTDCENTWNFYKNTQETSILLIAITDSDCPRLPLNRALMTVECRINAVVFVDQ
TILEENALLVKSIVHESITNHITPISLILYEINDSLRGQQKRVALRQALSQFAPKKHEEMRQQFLVISAFHEDIIE
IAETLNMFHVGNQWMIFVLDMVARDFDAGTVTINLDEGANIAFALNETDPNCQDSLNCTISEISLALVNAISKITV
EEESIYGEISDEEWEAIRFTKQEKQAEILEYMKEFLKTNAKCSSCARWRVETAITWGKSQENRKFRSTPQRDAKNR
NFEFINIGYWTPVLGFVCQELAFPHIEHHFRNITMDILTVHNPPWQILTKNSNGVIVEHKGIVMEIVKELSRALNF
SYYLHEASAWKEEDSLSTSAGGNESDELVGSMTFRIPYRVVEMVQGNQFFIAAVAATVEDPDQKPFNYTQPISVQK
YSFITRKPDEVSRIYLFTAPFTVETWFCLMGIILLTAPTLYAINRLAPLKEMRIVGLSTVKSCFWYIFGALLQQGG
MYLPTADSGRLVVGFWWIVVIVLVTTYCGNLVAFLTFPKFQPGVDYLNQLEDHKDIVQYGLRNGTFFERYVQSTTR
EDFKHYLERAKIYGSAQEEDIEAVKRGERINIDWRINLQLIVQRHFEREKECHFALGRESFVDEQIAMIVPAQSAY
LHLVNRHIKSMFRMGFIERWHQMNLPSAGKCNGKSAQRQVTNHKVNMDDMQGCFLVLLLGFTLALLIVCGEFWYRR
FRASRKRRQFTN
>DmelIR7a
MFHHLWLLMGLRSLAMGALHPPQPEAMTPLVAAALEILAEQVSPSQSTLAVMDLTQDAEHRDERQEQLMTIILRSV
GSEMALRTFQKPPAEVPASFVVFLVNSAQAFNTLGFHFTDIHSTREFNFLILLTHRMSSRAERLQVLRDISRTCVR
FHTSNVILLTEKRDGVVLVYAYRLLNMDCDLSVNLELIDIYKNGLFRHGHEARSFNRVLSLSGCPLQVSWYPLPPF
VSFIGNSSDPEERAQIWRLTGIDGELIKLLASIFDFRILLEEPCNKCLSPDIKDDCSGCFDQVIISNSSILIGAMS
GSHQHRSHFSFTSSYHQSSLVFIMHMSSQFGAVAQLAVPFTVIVWLALVVSSLLLVLVLWMRNRLVCGRSDLASHA
LQVLTTLMGNPLEARSLPRSSRLRILYAGWLLLVLVLRVVYQGKLFDSFRLPYHKPLPTEISELIRSNYTLINQEY
LDYYPRELTVLTRNGSKDRFDYIQGLGKEGKFTTTSLIATMEYYNMMHWSTSRLTHIKEHIFLYQMVIYLRRHSLL
KFAFDRKIKQLLSAGIIGYFVREFDACQYRKPFEEDYEVTPIPLDSFCGLYYISLIWLSAAVVAFILELLSQRIVW
LRRIFE
>DmelIR7b
MKYWLYILSCCSLVASTMESSSDWDLAEALAQVVANSEMGRFKTLYIYTHTNSQSTGGHLEELLDQVLMIVPNNLQ
ARRLLLQQSMEYKPYVHAVLALVDGLPSLSAIYARIRATQDLSHTLIYMSMPTDAYGEEMQATLRFLWRLSVLNVG
VVLRPPGDHILMVSYFPFSALHGCQVISANVVNRYQVGTKRWASQDYFPSKLGNFYGCLLTCATWEDMPYLVWRPD
GSGSFVGIEGALLQFMAENLNFTVGLYWMNKEEVLATFDESGRIFDEIFGHHADFSLGGFHFKPSAGSEIPYSQST
YYFMSHIMLVTNLQSAYSAYEKLSFPFTPLLWRAIGLVLILACLLLMLLVRWRHHHELPRNPYYELLVLTMGGNLE
DRWVPQRFPSRLVLLTWLFATLVLRSGYQSGMYQLLRQDTQRNPPQTISEVLAQHFTIQLAEVNEARILASLPELR
PEQLVYLEGSELQSFPALAQQSGSSARVAILTPYEYFGYFRKVHPMSRRLHLVRERIYTQQLAFYVRRHSHLVGVL
NKQIQHAHTHGFLEHWTRQYVSAVDEKDESVARIASTSYSTLDGIDGDPSLSESEEDQQVAPVRQNVLSMRELAAL
FWLILWANLGAVVVFVLELLLPRIKLRKILRKMKKSTRASATTTSTLSSPSTTKDIPFSCKDGFQDSWPKCSLLVS
>DmelIR7c
MLHSAVHNVSLVYALVWAIDNYYGMATSTPLAVVQFPTSRESRRLHNDLIDAALGRSSGTGRIQFLLEDDRVEMTE
TDTDPPPPSGLTGRPIAIWFLDSLRSYFRLEMYLNQLGSPYKRNGFFLVIYTGLEDQPMESLKIMFRRLLNMYVLN
VNVFLQRDGTVHLYTYYPYGPHHCQSSLPVYYTAFQDLAAPANGFGLTKPLFPRKLTNMHGCEMVVATFEHRPYVI
IEDDPKTPGGRSIHGIEGLIFRSLAERMNFTIKLVEQKDKNRGEILPDGNFTGILKMMVDGEVNLTFVCFMYSKAR
SDLMLPSTSYTSFPIVLVVPSGGSISPMGRLTRPFRYIIWSCILVSLIFGFVLICLLKITALPGLRNLVLGRRNRL
PFMGMWASLLGGLALYNPQRNFARYILVMWLLQTLILRAAYTGQLYLLLQDVEMRSPIKSLSEVLAKDYEFRILPA
LRTIFKDSMPTTNFHAVLSLEESLYRLRDEDDPGITVALLQPTVNQFDFRSGPNKRHLTVLPDPLMTAPLTFYMRP
HSYFKRRIDRLIMAMMSSGIVARYRKMYMDRIKRVSKRRNLEPKPLSIWRLSGIFVCCAGLYLVALIVFILEILTT
NHRRLRRAFNVINRYAA
>DmelIR7d
MDIRCVVALLLGLCKVQAVVWPHQHLLEEQLASQISATLQKIFINGLAVYNFGVFISTSYEEMDRDRVILVHQVLN
RNLYPPNFPVAVVLASKMNRKITAQVFTQLLFVQNAEQAIAIAEGVNRNGLCVIVLLTSQPERPIMTKIFTYFMQE
RYNINVVILVPRLHGVQAFNVRPYTPTSCSSLEPVEIDIKDGDLWDVFPRRLKNLHGCPLSVIVWDIPPYMRINWK
SSDPMDGLDGLDGLLLRIVARKMNFTLKLIPNEPNGLIGGSSFMNGTFTGAYKMLRERRANITIGCAACTPERSTF
LEATSPYSQMSYIIVLQARGGYSIYEVMLFPFEKYTWLLLSTILGLHWIVGSRWRMPSPILAGWMLWIFVIRASYE
ASVFNFIQNSPVKPSPRTLDQALSGGFRFITDHASYRMTLKIPSFQGKTLISAGQPVDVFDALLKAPWKTGAFTSR
AFLADHLVRHRKHRNQLVILAEKIVDNMLCMYFPHGSYFAWEINKLLFNMRSFGIFQHHSQILAWDNLPTTTDTDT
PGKRIHSSTESVATGFAESMSFVVAALNCLMGALCISIVVFGLELLSRRRHWTGLEWLFERV
>DmelIR7e
MNHINEFVARAVLHVVHHYILSVTPSLVLTLCCRSNHTCNFYNKMMSTLFREWGLAPLQIVNVLRGVPWHPVPGRR
HFNVIFTDSFAAFEEIRMEYYSREYNYNEHYFIFLQARDRLLQGEMRLIFDYCWRYRLIHCSIQVQKSNGDILFYS
YYPFGEHGCSDMEPQLINRYNGSMLVEPDLFPRKLRNFFGCPLRCALWDVPPFLTLDEDQEEVLRVNGGYEGRLLL
ALAEKMNFTIAVRKVHVNMRDEALEMLRRDEVDLTLGGIRQTVARGMVATSSHNYHQTREVFGVLASSYELSSFDI
LFYPYRLQIWMGILGVVALSALIQLIVGRMLRERMGSRFWLNLELVFVGMPLLECPRSHTARLYCVMLMMYTLIIR
TIYQGLLYHLIRTHQLNRWPQTIESLVQKNFTVVLTPIVQEVLDEIPSVQHMRFRLLEANSELDPLYFLEANHQLR
QHVTASALDIFIHFNRLSADKVHQRGEQGSGAHFEIVPEDIISMQLTMYLAKHSFLIDQLNEEIMWMRSVGLLSVW
SRWELSESYLRNEQSFQVLGTMELYAIFLMVLVGLIVGLLVFILELVSMRSIYLRKLFT
>DmelIR7f
MQGEDANLYVARALRLVIENVLAQLSTTLVVTISTRHLGTAHWFEYMMNILMDSWRMVAVQLLRIRPDLVVNPVPG
RKRVSLLMVDSYQGLLDTNITASNANFDDPDYYFIFLQARDHLIPKELQLILDHCLAHFWLHCNVMIQTAQVEVLV
YTYYPYTADACQKAYPIPVNTFDGRKWKASQMFPDKLSQMHGCPLTVLTWHQPPFVELVWDPKHNRSRGSGFEIQL
VEHLARRMNFSLELVNIALLRPNAYRLAEGSSEGPIEKLLQRNVNISMGYFRKTARRNQLLTTPMSYYSANLVAVL
QLERYRIGSLALLVFPFELSVWMLLLLALLIHLGIHLPSARRGNEEDGGGGLQVVALLLGAALARLPRSWRHRFIA
AHWLWASIPLRISYQSLLFHLIRLQLYNTPSFSLDQLLAEGFQGICTANTQRLLLEMPQLARDPDSIQSVDTPFDW
DVLNVLTRNRNRKIFAVANQDVTLSFLHSSAHPNAFHVVKQPVNVEYAGMYMPKHSFLYEKMDDDIRRLDASGFIH
AWRRASFASVHRKEQVHMTSRRYINHAKLSGIYMVMAGLYLLAGLLFAGEVLLRQRN

>DmelIR7g
MNVTSLLNFESMKYIGAQTQAASINHHVAQALRVFIEDFYQRIAPAFIVVLSCRRPSPMNFYRNIMQLLYESVDTM
IVQLVLVELGRPRRIAGPRTHNLLLVDSLDALLDIEIHTYTAQSDTSEYYFIFLQQRDALIPHDMQGVFAYCWRHQ
LINCNVMTQSSGGQVLLHTYFFPYAPGQCNDSQPTRINMFLGESWKHRDYFPSKLHNLNGCPLIVLARKVSPFLDLD
EGQRELRGLEGRLLQELSRRMNFSIQFSGLQDQLKNRTTWTEKQLLQKLVQERIAHLAIGYVRKRIQYATNLTPVF
PHYSNRVVGCLLLNAHNLTSLEIWSFPFQALTWICLVAGDRLALVLAVYAASLGLPIDPPERPSLQLLFASWLIFG
LIVRSMYSALLFFILRYHLHQRLPGNLQDLTHGDYAAVMGRTTLQDLREVPSLQDLLGLKSVIVTSEREEEVLRTL
DRCTLREGAGSHPLFFGLISQDALLHLTQRGHRAGAYHIIPQDVLEQQLAIYLQKHSHLASHLDHLVMSIRSVGLV
HHWAGQMASERYFRSRFLYREKRIRQPDLWAVYILTAGLYLLSLVVFICELLASRRAGL
>DmelIR10a
MAVLGTVFLLFMLDLKTLNLTRLNGLLVEPTRDLPQLELWLRAGSDHQDAENPYVQWFLLRTEIPLSIVTYQENRY
WMDDPFGRRNLVLVMSLDQLLTNRGAAAPIQKASTFFYILADQDKDLSADEQLRLEGSCRQLWTQHKVYNRFFLTR
DGVWIYDPFKRRDSAFGRLVRYYGSETLDKLLFRDMAGYPLRIQMFRSVYTRPEFDKETGLLTRVTGVDFLVAQML
RERLNFTMLLQQPEKKYFGERSANGSYNGAIGSIIKDGLDICLTGFFVKDYLVQQYMDFTVAVYDDELCIYVPKAS
RIPQSILPIFAVGYDIWLGFVLTAFACALIWLTLRVINLKLRIVSLGNQHIVGQALGIMVDTWVVWVRLNLSHLPA
SYAERMFIGTLCLVSVIFGAIFESSLATVYIHPLYYKDINTMQELDESGLKVVYKYSSMADDLFFSETSPXWNRDL
RADVIDEVARFRNKAGVSRYTSLILESSHFTLLRKIWVVPECPKYYTISYVMPRDSPWEDAVNALLLRFLNAGLIV
KWIQDEKSWVDIKMRSNILEADAESELVRVLTIGDLQLAFYVVIGGNLLAFLGFLAEHFRWKLQKKGV
>DmelIR11a
MRFAILWLFSGCLLPGIQVGIWVVVRAQPTGRDVLLSRLGNQQNELNTRRLANASSYLTRNYIANRINTLVVREIC
VECPYELSERQRQLVDQILASLAPELSVLLHKGTAEETTWEYTLFVVNDHTAFTGQVFIFPDELLEREFFCIVVVS
EIQSRQFVRQTVGSIVKSNLQMHFVNVVVVAQLEDGTVGTYSYKLFKANCTPGITVRQINHFDRITGKPQQSMPDL
YPVRNGHLGDCPFNVGAAHMPPHLIYKRHKDPPPASNVSIPAEDLAGIDWDLLQLLAKALKFRIQLYMPQEPSQIF
GEGNVSGCFRQLADGTVSIAIGGLSGSDKRRSLFSKSTVYHQSNFVMVVRRDRYLGRLGPLILPFRGKLWGVIIVI
LLLAVLSTCWLRSRLGLSHPIEDLLTVIVGNPIPDHRLPGKGFLRYLLASWMLLTLVLRCAYQARLFDVLRLSRHR
PLPKDLSGLIKDNYTMVANGYHDFYPLELTCRQPLDFSARFERVQRAAPDERLTTIALISNLAYWNHKHPNISRLT
FVRQPIYMYHLVIYFPRRFFLRPAIDRKIKQLLSAGVMAHIERRYMQYENKRKVASNDPVLLRRITKSIMNGAYRI
HGLVIVLATGMFILELLAGRSNGRLRRWMEWVHQ
>DmelIR20a
MLASLNRSTGLSAELLDLYGLVVHFLLSGEHTTLVYFNPAGLDCSWGVLWQRNLTAHPQIVWQRNYSYPDLYYQFN
AKLLVLACLPMDSRAAIQLEILANSLSHLRTVVRLLIEVAGPDQVTLARQYLSFCLRRSMLHVELYFRDYHHSLIL
YSFRAFPSFELVMRWISVGQGVKLFLHKLDDLRGHRLRVIPDLSPPNTFFYRDARGDNQVTGYLWDFLATFAGRLN
AGLEVVRPSWRAGSASDSSYMLEYSAKGLIDVGLTTTLITKWNLWAIHQYTYPLLVSSWCTMLPVEKPLATPDLFG
RIVCPTLAMTLLLIILVTWLVFRQLRCLTRLKNSRPARIVPHLLTLLLLTTCSAQLLSLLIFPPYHVRIASFEDLL
RGDQKILGMRNEFYNFDGAFRARYAGVFYLIDDPNELYDLRNHFNTTWAYTMPYIKWLVIKTQQRHFSKPLFRWSK
DLCFFDFMPTSVIVAPDSIYWESIKDFTFRIHQAGLMKHWIRKSFYDMIKAGKMSIKDYSDLETLKPLNIGDLEIV
WRVCGAAIAVASAIFIMELLYFYINVFFNSL
>DmelIR41a
MFIDLSWSLVLSAIVGKYLNESTICIFWNDKFEFQLLHKSDYISFVGINIKSFDDNGGHYIIDTGLKKKELQNKHL
FLDELVIKIIISIEVTHCETFVVFDKDIDRFVNAFNKASVYSIWRSLHNKFVFAHIANESPESRNHFFEDQPNILF
VVRDHSSASSFDIKTNKFVGRKAENPSQMILVDRYLASEQRFQFGKSLFADKLNNLQGREVIIAGFDYPPYTVIKH
NMSTNAQDMGVSGESDFKNVYIDGTETRIVLNFCEQFNCTIQIDSSAANDWGKVYPNMSGDGALGMLINRKADICI
GAMYSWYEDYTYLDLSMYLVRSGITCLVPAPLRLTSWYLPLEPFKETLWAAILLCLCAEATGLVLAYKSEQALYVL
PGYREGWWTCTSFGVCTTFKLFISQSGNSKAYSLTVRVLLFACFLNDLIITSIYGGGLASILTIPSMDEAADTVTR
LRFHRLQWAANSEAWVSAIRASDEALVKDILYNFHIYSDDELLRLAQDQHMRIGFTVERLPFGHFAIGNYLGPQAI
DQLVIMKDDIYFQYTVAFVPRLWPLLDKLNTLIYSWHSSGFDKYWEYRVVADNLNLKIQQQVQETMTGTKDIGPVP
LGMSNFAGFIIVWILGSAIATLTFLLLELSLTYILKQSNLK
>DmelIR47a
MRQIKLLVWLLVVGVVSSTEQLQFLKNFLEAVHKERSISTILLIQRKVHKNDFLHGLYPIFWPIICLDETKRVELV
NNFNKDFLALVYMESEADTLLLSALAADLNHIRDARIMIWLQMSPSENFLDRIVFQASKQKFLNLVVIENTLKTRR
FYPFPQPKVQVIDKPFEEKEIYPALWRNFMGKNAIAVPDLVPPRSFNSFDPKTGHRRESGSIYNVFKAFTQRYNIT
MLLKWPLIRNTTQEEIIGKSVRGEIDLPITGQLISFRHPNGSRSQPLLGMTALSIAVPCGPELPMFDRFFLFYGLA
TPITITGYYVLLNTIEIILGTLSDRIKRHPRRKKILNLVLNLRVFSCILSLPTPQGNRLRSVKGQLTMVMSITGLI
LSCIVAAQTSTILTMKPQYRHIKNFQELSDSNITVVCNHLNYLTIKQQMDPKFMAKFMQNIWIVNSIEQMKMIFDL
NTSYAYQTFSYKKDPFTLLQMHTTRKAFCRTPGLDLVSGLAYTAVLEKNSIYALALQDYTLKAFSAGLVYYWAEES
IRDLISTVGRTQFEKLPIVIGYQSLKLQDYNVCWKILLIGGALAFCVFIVEVVVGLINRRI
>DmelIR47b
MREAQIIIFLLTSAAAVTLKQYEFLXSFLKAGEQEQTITTLLMMQKHVHTKNLLQGLYPXPWPIIHFVETQRIKFI
ALLYMSSEKDIFLSSLAANLKFERLDKPFGKSNIFPVLWRNYMGXIALTLDHLVEPRSFYWTDPRTNIKRRTGYIY
MLITNFAEQHNITLQLXSPPNEDMSQMVIIERTHKGPRSTHNWADDQLETFERXQDSLLPWHGSMAIVVPCGQEMS
AYERFHAAHAFRAPIIFFGFHIFLSLIDFLLRTISDRIRCNPRRIQLLQTVLSLCVLRCILSTSLPNSNXLRSRLR
DNSPXXXVLQAXSYSALWXLTGTAXQXHNRDFQSHKLHDYXTTDGSXHSIEVPGLLKARNXXIXLFHIFSSLGTKF
DLRIGSAGSHTSGVEFRYYELLDRXSSLENNIVSQVFTILKLPYSRFRVLKLEDCRGCWQTLFVGFSIATFVFIVN
VLMGFFRNINQKK
>DmelIR48a
MHLLITETYMIIGKTLHDILNELNERLIISTNIIFCKQFDNLIHFEAQTSRFVYSSLEAFNITSLWNHVGNDNKLF
VIVGNVPPYELFAKLELSSPENCTQFILNNTVDMCADALVKNSKAFSVSRELRIAPANVIVPHGKPLLSYRYLAAP
FNTKVWIALGTYVFLISGFLCLIHWLRSGKWDFSQNLLEVYSSLLFTVFHLKATNGIERYILFGVLFISGFVYSTS

YLRLLKSMLIAETFEKQIQTFEELAESNIPLLINPYDRMIFQHHHIPKSLWTAVRTVSSETLLNHRSHGYVRLCPA
ILTASKIPSHTHRHLFSVCRFSHEQEVVPKGSSXXSLVPCIRKRNREXNHLGCLSGVSWPGISXFFHYGALGGEAF
GSILLHDANYFPSPRLFRRLAELHYGSY
>DmelIR48b
MILQQSSNLLKLLLLLAISSVRTQGLNDIIIELNQRLLISNNFLYCNQSDKLNEYEIKYLQHMPPISLMIFTSIES
MNFTQVEYNLGADNKLFLIMGNEEPPYDFLHALNLHFQFAEYIIVIDEPVDLKKSTKWLDFVNHLWQQGYVQLLIY
TSYDEKLYHKIIFPETVIEETLVEQYISIRGSFNNLYGYPVRVAAYNNAPRSMLYVNRWGKHIFAGFYMRFLRAFI
DARNGSFVPVLTPSNSPGNCTLNLVNETVDVCADALAANPAAFSLTHGFRIASANVLVTHAKPLHSYRYLTAPFQW
SVWACLVIYVLLVVNFLSFIGWLRSGKWEFSKYLLEVFSSLLFSGFYLKEIRGRERYILFGVLFIAGFVYSTEYLG
LLKSMLISEVFEKQIDTFEALVESNITLMVDPYDKILFAKYNMPEILSPIMELVSFETLLKHRNRFDQDYAYILFS
DRMALYDYAQQFLKHPKLLRIPIDFSFLYTGIPMRKRWFLKHHLGRAWYWAFESGLTRKLALDADFEAVRVGYLSF
LITEHVEAQPLNVDYFVMPAIALAIGYILALLSFVIEMTAWRIREFLGCRKATMTSTGCSEGGHVDVD
>DmelIR48c
MSLLRIILIIIFLRIVSSIPDTIISHLSAELQIKIQIYFGLGNDLYDFSRLDGNYQKIIISHNISEEFKTYHDEPV
LIIIRLERDLNLNLATLDVLRSYLTDRQYNDILLIDNDEENLNSYVDIRKAYWNAGFSQVLIYNSQQRTWSIKPYP
YLQIRPTSLKEYIENRNTRNLMGYPLRVLVTNDPPHCFVDKDELPGSPNRYKGSIVTMLKIFADQLNATFQANPFR
EFRRYSTADCVQMVSDDEIDACGSIFIRTYTYATSQPVRLNRVVIMAPFGNPIEKFYYFFRPFDLYVWIGTGIIVV
YIAVMGSLLHRWHFKEWNVGQYLLLAVQTLLNRELSLPQSSSGSKFMLLLLLFAIGFILSNLYVALLSMMLTTKLY
QRPIENLADLKAANVNILLQTHNIRPNSVYGSSEELRERFLLVEESQHLEKRNGLDPSYAYVDSEDRMDFYLYQQK
FLRRRRMKKLSNPVGYTWAVQVIKQNWVLEKHYNDHVQRFFETGLQNKLVDDVHELAVKAGFLHFFPTQTQTIEPL
RLEDIVMAAMVLGGGHALAVICFLVELFA
>DmelIR51a
MYNVLVLFLLLFTRAQMEPHRRGHNMTLLRSVLTVIRGRENWKNTPIFLGGHCNSDDLNNLMSWLQNTMEVTCHTV
DTSTSAKNENALGHFNINADNSLGLLFCQSSHELIWFNMDKRLRRLRGIRLIVILSDKRSSSSKAIMSTFKRLWHF
QFQXNFQGYVVSTPVENDIPRVFFVKDKKTGRKQIRGFGYRTFVEYLHRYNASLHVSNSQQEHAINSSVNMGRIIN
QIVDGQLEISLHPYVDVPENMGDNSYPLLIASNCLIVPVRNEISRYMYLLLPLNQSSWILLLGSVIYISGVLYYIQ
PGLLHRTWDQRIGLNILDSISRIINICSPSRIYNPSLRYFIVSVHLSILGFVVTNLYSIMLGSFFTTLVVGEQVDS
MQQLIQXQQKVLVKYYEVSTFLRHVEPDLVDGVAQLLVGVNASEQVSALLGFNRSYAYPFTLERWEFFSLQQQYAF
KPIFRFSSACLGSPIIGYPMKSDCHLQSSLNMFIMRIQAAGLLRHWVVSDFNDAMRAGYVRLLENFLGFHSLDVDS
LRLRWAVLLCGWLLSTLIFLCER
>DmelIR51b
MCKVLTLLVVILLLALTNAAYNVTLLKSVLSLISTREPWINTPIFVGHNTQGGDLNDLIIWLHQTMGVTSLTMNLF
LQPEHIRPLGHFKITRYNGIALFFCHDKHDIMWLTLDRNLRKLRRIRLIIILRNQRSGSQGAIKSIFNALWQYQFL
NVLVLQRDQLYSYTPYPAMRFFKLDIHTEPLFPHAARNFHGYVVSTPAENDIPRVFHVDPLTKSRKVLGYAYRTF
VEYLDHYNASLRLTNPDENLDPTTSVNMNHIVQLIIDGQLEISLHPYVFTPPTATKSYPLLIYPNCLIVPMRNEIP
RHMYLLRPFQLYSWYILLFAVFYITGILYCISPKLNKSSWPQRLGLNFLDAISKILFISPPITIYRPTWRHLIIFL
QLSVLGFMSTSWYNIELDSFFTTIVVGEQVNSMDQLVHQQQRVLVKEYEINTFLRHVEPRLVEKVSRLLVPVNASE
QVSALLSFNRSFAYPFTEERWQFFAMQQQYAFKPIFRFSSACLGSPHIGYPMRVDSHLETSLNHFILKIQDTGLLN
HWVVSDFNDAMRAGYVRFVDNVLGYQSIDVDTLRLGWCVLGIGWILSALVFSCEYWHLYPWRFIA
>DmelIR52a
MALGWSVIILGFIGQLSAQILNYTQSRDLELLEGSLFRVLSRLNLEEEYNTLLIYGKECVFHSLLRKLEISAVTVP
SGSTDYDWSFSTAILILSCGYDAENEENSYTLMKLQRTRRLIYLEDNSEPESVCMRYSLKEQHNIAMVKSDFDQSD
TFYSCRLFQTPNYVEGHFFKDQPIYIENFQNMRGATIRTVADSLVPRTILYRDEKSGETKMMGYLGHMINTYAQKL
NAKLHFIDTSKLGAKKPSVLDIMNWVNEDIVDIGTALASSLQFKNMDSVWYPYLLTGYCLMVPVPAKMPYNLVYSM
IVDPLVLSIIFVMLCLFSVLIIYTQHLSWKNLTLANILLNDKSLRGLLGQSFPFPPNPSKHLKLIIFVLCFASVMI
TTMYEAYLQSYFTQPPSEPYIRSFRDIGNSSLKMAISRLEVNVLTSLNNSHFREISEDHLLIFDDLSEYLVLRDSF
NTSFIFPVSVDRWNGYEEQQKLFAEPAFYLATNLCFNQFMLFSPPLRRYLPHRHLFEDHMMRQHEFGLVTFWKSQS
FIEMVRLGLASMEDLSRKRNEEVSLLLDDISWILKLYLGAMFISSFCFILEILRCGERCKRLWRCRW
>DmelIR52b
MTWLVILLCFLGYMAAHIADISVQNQSLMDNELINLLLKLRNEEFYDTLLVYGKDCEFHSVIKNVDVAVVLVSDSM
NFEWNFSSLTLILSCGPDIDNGGPNSTSIKLQRNRRLVLLKEDFQPSNICNIYTQKEQYNIALVRENFTKSKSIYT
CRYFQDPNVDEVNLSGTKPIFIEQFQNMKGKAIRIVPDLLPPRVMLYQDANDGELKMIGYVANLITNFAQKVNATL
QLDFLKPSTSITEISRMAKDDELDMGITLEASLNTSNLETSSYPYLLTSYCLMVQVPAKFPYNLVYALIVDPLVLG
IIFVLFLLLSVLLIYSQKMSWQDLSVANILLNDKSLRGLLGQSFPFPLNASKKLRLIFTILCFASIMLTTMYEAYL
QSFFTNPPSEPEICSFQDVGSYNRRIAMSALEVNGLIKTNNSHFREIRMDDLEIFDNMPECYELRDAFNLSYNYVV
TGDRWRSYAEQQTLFKEPVFYFARDLCFSRLIFLSVPLRRHLPYRHLFDEHMMQQHEFGFVNYWMSHSFFDMVRLG
LTSLKDLSRPLAYTPSLLMDDISWIMKIYLAAIVLCVFCFLLEIGVDKWKRWMKFRNLQILNTC
>DmelIR52c
MVWLIIIILFCLGNSSSQILDVTNNSHLDFDYRLFGLLQRLQVEKSYDTLLVYGEDCAIPSLFERLQVPAVLVSSGS
TNFDWNFSSLTLILSCNFQDEREENYRTLMKLQTSRRLILLKGHIKPESVCDFYSKKEQHNVAMVKENFYQLEVVY
SCRLFQDQNYEKLNLFDGKSIYKDQFRNMHGAPIRTLSDKEPPRTIPYIDSKTGEEKFKGYVGMLISQFVKKVNAT
MQIREDLIKDDEEVSFVDITNFTSNDILDIGICEARTLEMSNYDAISYPYLMSSYCFMAPLPDSLPFSDVYMAIVA
PSILIMFLIIFCICSVLIIYIQERSYRSLTIRSVLMNDICLRGFLAQPFPFPRQYNRKLKLIFMLVCFSSLISTTM
YTAYLQAFLWGPPIEPRLTSFDDVKKSRYTMAINIYEREFLEALNVSLEDVEIYDYGKFSKLRSTFNTNYLFPVTA
LQWFTINEEQKLFKYKIFYYCDAFCLNQFDILSIPLRRHLPYRDIFEEHMLLQKEFGLTKYWIDQSYRDMIRANLT
TFKDFSPLLENDYIEVHNLYWVFTMYFVGMGMGLCFFILEILRPLRYWRNCKIKCEYCYAFLKNFAK
>DmelIR52d
MVRIIIILLCLGYTKARILDATNTNHTDLEERLLSLLLRLQQEQFFNTLLIYGEDCAFSSLSRRLQVPTILVSSGS

TSFEWNYSSLALILTCEFKAEREENYQTLKKLQMNRRLILLNGNIKPDSVCDFYSKKDQYNIAMVNNNFHQVGIIY
ACRLFQERNYEKVYLSEGNPIYVDQFRNMQGALLKSITFNLIPGSMAYRDPKTGQEKHIGYVANLLNNFVEKVNAT
LDMQVKLHKAGKKTSFYNITKWASEDLVDIGMSYAAYFEMTNFDTISYPYLMTSTCFMVPLPDMMPNSEIYMGIVD
PPVLVVLIAIFCIFSVMLNYIKQRSWRSLSLVNVLLNDICLRGFLAQPFPFPRQSNRKLKLISMLVCFFSVITTTM
YTSYLQSFMWGPPIDPKMCSFADLENSRYKLAIRRYDIEMLRPFNVSMDHVVVFDESSQLEYLRDSFDDNYMYPMS
ALSWSAFKEQQKLFAFPLFYYSEKLCLKPISFFSFPIRRHLPYRDLFEEHMLQQNEFGLSTYWIDRSFSDMVRLKL
ATMNDFSPPRLEDYIEVSDLSWVFGMYFTGLGISCCCFGLELLGLPSWTRRLRLTNWLRVRN
>DmelIR54a
MWTVITGIVLWAPVLVAGSAVDFIFRAAAEHSLSVIMIRIDYCPYNWAKDIFENQTIPVVVLSDSETFINIRMFSR
PLHVACLPGHELQKDLALLENFTSSLMDFPSQKKIVYISNNFSDPTRMDYIFETCYHRRIWNIVGLLASDEHRYFY
RYHLYPSFRTEYRSLESSTIFDKDFPNMHGHPLTVMPDQWLPRSVLYVDRRTGKQILAGSVGRFFHVLSWKLNATL
QLSKKVTTGRFLNATALKELSESFSVDVPASLTIMERVEQLASTSYPMEVTHVCLMVPVARRIPIKDIYFILSSAS
NMFLAIVIVSSYGLALNLLRNMTHRDVRLVDFVLNDKALRGILGQSFNLPLSRSFSTRLIFLMLGIVGLNVSSIFG
AGLDTLMAHPPRQFQARSFAGLRRTKIPLVTTEEDFPTWMKLRVPMLVVNVSEYNHLRNGRNTSNAYFASRLYWNL
FSEQQKRFTRELFIYSTDDCLWSLALLSFQWPQNSLFTEPVSQLILEVNANGLYDFWVGMHYYDMTAAGLSGLEDP
SLQLKEREHPTSLRIVDFQWMWQAYGTFMVIAILVFLLEVSWHRITSLFVSLVY
>DmelIR56a
MGSRFFIRNLILFGLLASSNMQIPFGELEKKFELDVDFLLGVTELVGHIQGLYSITVYADCIDIHPSIQQRIMDKF
MVPVNTIGSNLSRPNYHKLDNSRIRIVLFTGLNDTILVNLNKTDVPYSDNFYMLAYASAIKNKCIELDFIEEVFTL
LWKMSIQNAILLIRGEFMMEMWSYLYMGKIHKIKLTKPNSYLESLRKYNYRFSLEVINDPPAIFWYNSSEQADVTG
GGNLSVSGPLGLIIINFLRHLNVTIDIVPIPGKQTSQYELFQQPDNLRAENGVNMVGSALLKYSPMVTQSRMCLLV
SNRRMIPFSRFLDRLVSPGVHKLTFVSSIGIFVIKYFSHRPRSFVDAIFCTIRFFFAIPLPSIILNRLPVVDRFIE
VFIIIFVQILLSSNISITTSALTTGFWEPPIINVETMRASGLHILTEDPTILQAFKENILPSSLADLVILVDEDTY
FHHVTTLNNSYVYVVQAHNWQIFRLYQQQMTNEPFEIASEELCSKWRILGIPLNPKSPLRFMFKDYFYRILESGLR
EQWVHSGFKKFCEFNNLKKLPVDSVDSWQPLSIEFYSNVIRAYIIGLVIATLAFVAELLHNGYRRKNVKKT
>DmelIR56b
MLLDTDLASGVIRSPYSFDIPHAFIFNETQFVVPKFCGPYMEIVKHFAEVYHYQLFLDSLESLPKKSVVEQDIISG
KYNLSLHGVIIRPEETSDFFNATQHSYPLELMTNCVMVPLAPELPKWMYMVWPLGKYIWTCLFLGTFYVALLLRYV
HWREPGNATRSYTRNVLHAMALLMFSANMNMSVKLKHASIRVIIFYTLLYIFGFILTNYHLSHMTAFDMKPVFLRP
IDTWSDLIHSRLRIVIHDSLLEELRWLPVEYQALLASPSRSYAYVVTQDAWLFFNRQQKVLIQPYFHLSKVCFGGL
FNALPMASNASFADSLNKFILNVWQAGLWNYWEELAFRYAEQAGYAKVFLDTYPVEPLNLEFFTTAWIVLSAGIPI
SSLAFCLELFIHRRKQRRPQYERFECYDY
>DmelIR56c
MRSSFRICLFLLTTYHPSHGWNMQHLLNLLAPFGRMNVFQEIVWFVSPHQRLDQLDEFIMRIDEAFGKSATQTVVN
NNTEMRMIYSSARRNHMSFVFTTGAEDPIMKVFSKVLLGRHFYVSMVIYVDKVGDMHPIYDLLTFAYNQQFFNSMV
HFESMEGVNQLFGVSKFPVMSFENRTDFLKYMGKIWKQVQNARSDVGGFGFTTPLRQDLPHLFQSQGHYDGSTYRI
IETFVRFINGSFKELIMPPDSLGGQVINMKDALQLIRERKMEFCAHAYALFMSDEELEKSYPLLVVQWCLMVPLYN
SVSTYFYPLQPFDWNVWFFALGALLALVLLELMWLRMFGGWSGYRGAVLNSFCYIINVPIEGQLQQPCLLRFLLLA
TVFFHGFFLSAYYTSNLGSILTVNLFHAQINTMNDIVSAQLPVMIIDYEMEFLLNLNKELPQEFLELLRPVDSAVF
SEHQTSFNSSFAYFVTEDHWEFLDEQQKHLKQRLFKLSSICFGSYHLAFPLQMDSSLWRDIEYFTFRIHSSGLLNF
YARSSFGSALHAGLVQRMPDTQEYTSAGLQHLAIAFILLLVMSFLAGIVFVLETLSR
>DmelIR56d
MDNRAAELILRERNIFPTNGSDNITLLNNMFVLEMFYRITQLYHFKNFIFYISERLDLNNKDSQEFFHNFWTYFPM
APNLIITREHHLGIPMMQFISTPSLVMVFTTGKDDPIMELASHNQQGIHWLKTIFVLFPSLQSRDFETNPESLAQF
TAEIKDVYDWVWRKQFINTFLITIKDNVFILDPYPTPSIVNKTGVWQAEEFFHKYAKNMKGYLVRTPILYDMPRVF
KSDRPTNRYEKNFIHGTSGNLFLGFLEFVNATLMDTSANVTADYLNMTNLLDLVSQGVYETLIHSFTEITTKFVVS
YSYPIGINDCCIMVPYRNQSPADQYMHEALQENVWVLISLFTLYITVAIYLCSPLRPRDLSAAFLQSICTLTYSVP
TFIIRTPTLRMRYLYILLAIWGIVTSNLYISRMTSYFTTAPPVRQINTVQDVVEANLRIKMLAIEYERMAKSPLQY
PESYLNQVDLVDKHMLDLHRDPFNTSFGYTVSSDRWRFLNLQQLHLRKPIFRLTEICEGPFYHVFPLHKDSHMRSV
MTEYIMIAQQAGLMNHWERETFWEAVHLHRIHVHLFDDEPMALSLDFFSSLLRTWTLGLILAGLAFAAEMKWHEHV
TFKRRPVIRITRKPRSFLRRFMKL
>DmelIR56e
ERXAFRNQWAFCFPRTXAIEVVLSAWSPXCPGQRSKPQPISXPHHXGSCWRKRKWKXKPRLLVVDKRTLVEHLNSL
NDGYAYCIIAGHWQVGMM
>DmelIR60a
MWCNNPGLIIIIFLGQILNLCQGIVNLSNETANTVIFMLPEKDLGPDVWKAGVGCLDSFAQIFFFRNPKERFTRAY
NLMLVHAFHLSSPADQIQEGFSKLINEAVTNPGPPDREELFQMRVASDYNITNGTEDKGELILADNYVIVVDSVDR
LKELMKKKIVEMRSWNPGARFLVLFHNATCRNRPLGVASNIFKDLMEMFYVHRVALLYANSTMNYNLLVNDYYSNV
NCRILNVQSVGQCHDGKLYPNNAVVKASMQDYVSGFSPRNCTFFACSSISAPFVEADCILGLEMRILGFMKNRLKF
DVNQTCSLESRGEMDGPANWTGLLGKVQNNECDFVFGGYYPDNEVADHFWGSDTYLQDAHTWYIKMADRRPAWQAL
VGIFEAYTWIGFILILIISWLFWFTLVMILPEPKYYQQLSLTAINALAVTISIAVQERPICETTRLFFMALTLYGL
NVVATYTSKMIATFQDPGYLHQLDELTEVVAAGIPFGGHEESRDWFENDDDMWIFNGYNISPEFIPQSKNLEAVKW
GQRCILSNRMYTMQSPLADVIYAFPNNVFSSPVQMIMKAGFPFLFEMNSIIRLMRDVGIFQKIDADFRYNNTYLNR
INKMRPQFPETAIVLTTEHLKGPFFILVVGSCWAALTFIGELIIHRWRTQLVSTSEQQDRRSDKRRRRRRRRKPEK
DNRWQRQVQVAPVVRFTPVKRRKVFQGQTSQK
>DmelIR60b
MRRSLYLIIAIGLVDVHCVSLRYILNALENELQYRAILLVESASEIESCWEQKYIQGAVPILNFNANQSLYLKDAL
NTNILALVCLNENVESTMQALYENLEDMRDTPTILFVLSDSKVQDVFLECLRRKMLNVLAFKGLDRGFVYSFRAFP

TFRVIERNVMDILQYFEQQLEDLGGHTLTTLPDNIIPRTVVYKSPDGSRQLAGYLYPFLRNYVSTINATLKVCWHL
VPEDGMIQLGEVVRLSEIHDVDFPLGMHGIEHGSTSQNVPLEVSSWFLMLPMEPSLSRAQFFIMLGFEKVTPVLLL
LTILLSTAHRIEMGLRPSWRCYVLGDRVLQGTLGQAFFLPRRLSVKLMLVYSLILLNGFTFSNYSITSLETWLVHP
PSGHPIHSWEQMRTLNLKVLIVPSELDSMTKALGKQFTESNSDLFELSKSGNFQDKRLAMDQSYAYPVTCTLWPLL
EHAQIRLPKPEFRRSREMVLIPLLIMAMPLPKNSMFHKSLNRYRALTHQSGLYEFWFKRSFNELVALRKIHYKVNG
DHQIYRDFEWQDFSYVWLGFVGGTIASILVLLAEIGYHRWQLNQN
>DmelIR60c
MEMRLALFFTFACLAGAHDGSLRNMLKSLEDELGYRTILLLEGFVYSFKAFPTLRVVKRRVKDVRRYFEPQLEDLG
GCVLKVVPDGIMPRTMVYQGEDGELQMGGYLSHFIRNYVSTINASLQIRWDLFPEDGDFDMDSLTGSNHVDFPLGL
GSLSFQTLHKDVAMEISSWFLMLPMEPSLPRARFFIRFGISLYLIPLIILLAIVLSNAHRFEAGLTPSWRCCSMGN
TVLRGVLAQAFVLPKGLSPKLMFVYWLLLVSGFFVSNYVIVYLTAWLIQPPTSDPVTDFDQMRRAKLKILMVPTDM
DYLKSIRGAEYVDAHSDVFQTADSTDFQTQRMSMELHFAFSVTGTLWPLLRQAQVKLHRPIFRRSKEMVFLPFVIM
GMTMPNNSIFLSSLKQYRLRTSEAGLYLLWFKKSFSELVAIHKISYKEDWVHDSYSDLKWEDFLFAWLGFLGGTTV
SCLALLAEIGYHRWLWKRTHQ
>DmelIR60d
MRLAIYVAFLSSIGNRSGFLSSLLMSLGKELHYKTILLVGGSSTCWSLEPFETGVPILNLRGENNAYPQDTFNSQM
LALACLQTESEDAVKLLYRSLKDMRDTPTLLFASSEEHIHDTLFLGCFRENMLNVLALTASSKEFIYSYQAFPTFR
VIKRKLVEIHRYFEPQLKDLGGHIVSALPGNIMPRTMCYRNAEGERQLAGYLNTFIRNYVESINGTLRISWGLVPE
DDMRHLTISRLSKIQHVDFPLGIIPLYNKTDKQHVYMEISSWFLMLPMETSVPRAHLFVKLGLERLLPIIVVVGAV
LGNAHRIEVGLGPSWRCYYLADKVLRGALAQPIVLPRRLSPKLMLIYSLLLLSGFFLSNYYMASLTTWLVHPPASD
RILEWDQLRYLHLKVLTIPEEFKYMSLILGTDFMTAYGSIFQLTNSTDFQRRRISMDPSYAYPVTTSLWPFLELSQ
VRLRRPLFRRSYDMVLQPFQVMSLPLPRNSIFHKSLLRYAALTRETGLYYYWFRRSYYELVALGKISYKEEEGNPY
CDLKWNDFRIVWLAFLGGTIISCLALLLEVAHYRWHLGNSSL
>DmelIR60e
MVIKMISFLLVSVLLCLVGASDSESMQVQVLQDLNLALQTELNVFIDFECCATSEILHKLDSPRILLSSNSREARD
LRIRGNFTESTLIIVSVMDSDLNPLVASLLPRLLDELHELHIVFLSNEEPGFPKQDLYTYCFKEGFVNVILMSGKG
LYSYLPYPSIQPISLSNVSEYFDRARIIRNFQGFPVRILRSTLAPRDFEYSNEQGGLVRAGYLFTAVKELTYRYNA
TIESVPIPDLPEYDVYLAVAEMLHTKKIDIVCYFKDFSLEVAYTAPLSIIREYFMAPHARPISSYLYYSKPFGWTL
WAVVISTVLYGTVMLHLAARGARVEIGKCLLYSLSHILYNCHQKIRVAGWRDVAIHGILTIGGFILTNVYLATLSS
ILTSGLYDEEYNTLEDLARAPYPSLHDEYYRSQMKAKTFLPERLRRNSLSLNATLLKAYRDGLNQSYIYILYEDRL
ELILMQQYLLKTPRFNMIRQAVGFTLESYCVSNSLPYLAMTSEFMRRLQEHGISIKMKADTFRELIHQGIYTLMRD
DEPPAKAFDLDYYFFAFVLXTVGLISSLLVFFAELVSGHL
>DmelIR60f
MRFHLNIANSGLLGLHLCPTRSALPEQNPCFSKAGAVIXNLTLPWRRWRERCLLGALRPXTLPTPELQCXSKYLPX
RKSQQENASSGLPGFCXGDXQTELHRGSRAIALPRSPYHDLYYVWIAYLGGTMIGIGMLAVEIACFKWDLLRRPPI
XMY
>DmelIR62a
MYLQFLFALFLSRYQIVATENFDRAFELALFLDRIGRVHRLHAITIVNSLGSVDPSYLDDLHRGLMCNSSNHFYML
PQMTATDKDSSHVHFSSLQDEETIYLVFARDSKDAVIYLQAERARGRRYTRTMFLLRKQESQKDIKYFFELLWKLQ
FRSALVVVAARNFYQMDPYPTVRVIRMRRLSSYDPHHVFPPANRKNFRGYRMRLPVQQDVPNTFWYKNRRTKAWEL
AGLGGILINQLMMHLNVTMDLFRFEVNGSSLLNMAALTDLIVKGKVELSPHLYDTLQSNTSVDYSYPTQVAPRCFM
IPLDNEISRSLYVFLPFSLTMWLCLLFVLLVVHFVYVRRLIPDGHFWAILGVPGAGQVRYGNRKPVRRFSTFLILF
GIFILGQTYSTKLTSSLTVTLIRRPDNSLEELFLLPYRILVLPTDVYAIVDSLGHAEQFSTKFSCTDAENFSQKRI
SMHPEYIYPISTIRWRFFDMQQRFLRKKRFYFSKICHGSFPYQYQLRVDSHLKDALHRFLLHVQQAGLHDLWLDTC
YRKAHRMGYLKDFSTLAELEEKLRLRPLALNLLVPAFSLFLCGMLGSGIAFLVEIRHSFGCRQKPPSINRNPGD
>DmelIR67a
MLPILVPVLLLFNETSWINPILTSIYKDRHHETVLLLQHSQHGNASGLERFPWPVFSFNEQMDFYVRGKYNSEMLV
LIWQTGNSDWDLDLWQALDRSLLNMRKVRVLLLRKWEKIPTADVAATAEHLLFLHVAVIGQGNRIYRLQPYAPQSW
LQVDPIESPIFIKIRNYFGRYIVTLPDQFPPRSIVYRNPKTDEIQMTGYVYKFLLEFIRIYNFTFRWQRPIVQGER
MNLILLRNMTLNGTINLAISLCGFETPSXLGVFSDVYDMEEWYIMVPRAQEISIADVYVVMVSGNFLIVLIIFYFI
FTILDTCFGPLLLKERVDWSNLMLNERMISGIMGQSFNMSARNTISSKVTNATLFLLGLVLSTLYAAHLKTLLTKR
PTSQQISNFKQLRDSPVTVFFEEAERFYLKHAWDRPIRYIKDQLNFRETIEYNALRMGLNRSNAFSALTSEWMIVA
KRQELFKQPIFTVQPELRVIQTSVLLSLVMQSNSIYEDHINDLIHRVQSAGIVEYWKHQTLREMITMGMISQKDPF
PYVAFREFKVGDLFWIWLLWVSFLFMSFVIFLCELLVDCFISKTLIRNKRPH
>DmelIR67b
MELLYLNTLQSLSLLEGNRLVQTVQELNNIYQTELNVFLEFGNGADILESAQGTFVPTLWIKNPQNQKVMKGNFTS
CTLTILYLEDEHLDRGLYYLANWLWEYHHLEVLIFFNGGSYDKLIQIFSRCFNEGFVNVLVMLPGSDELYTFMPYQ
DLKILNLKSIKEFYSLSRKKMDLNGYNITSGLVIAGAPRWFSFRDRQNRLILTGYMLRMIVDFTNHFNGSVRLMNV
LTVNDGLELLANRTIDFFPFLIRPLKSFSMSNILYLENCGLIVPTSRPLPNWVYLLRPYAFDTWIAWLIMLIYCSL
ALRILSKGQISISAAFLKVLRLVMYLSGSRDMGTRPTTRRLFLFVILTTSGFILTNLYVAQLSSNSAAGLYEKQIN
TWEDLDKSDSIWPLIDVDIKTMEKLIPDRTKLLKKIVPTLEADVDTYRRNLNTSCIHSGFFDRIDFALYQQKFLRF
PIFRKFPHLLYQQPLQISAAFGRPYLQLFNWFVRKIFESGIYLKMKDDAYRHGIQSGLLNLAFRDRHLEVKSNDVE
YYYLIAGLWFGGLTLATVCFLLELLIGYAKIKVTISCKMNIM
>DmelIR67c
MFCWLIFLNIILLSDRSESWSAREVIHQFNHDQQLQLNIYLDCNDVELQIGQEVSNLFVNSTADKMKILGRFSSHS
LIIACFKDSTRNRTLNGVKELLWGLQYLPILFVVDSNMDFYFQQALRHGFIHVLALNFMNGSLYTYKPYPKVEVHQ
IKDMQKFYKLTKLRNLQGQAVRTTVETMTPRCFRYRNRHGQLVYAGYMYRMVKEFISTYNGTEEHVFGNVDTVPYK
EGLAALKNGEIDMMPRIIHALEWYYFYRSHILYNIKTYIMVPWAEPLPKSLYFIQPFRGTVWITIMVSFVYASIVI

WWIRYRQQGNSSLTQSFMDVLQLLFQLPLSKIWHFNMGTHQVVSFIVLFVFGFMLTNLYTAQLSSYLTTGLFKSQI
NTFDDLFREKRTLLVESFDAEVLHNMTKEKIIQKEFESIILITSIEEVFKHRKSLNTSYAYEAYEDRIAFELSQQR
YLRVPIFKILKEVYDQRPVFVALRHGLPYVELFNNYLRRIFESGIWIKLQEDSFLEGIASGEISFRKSKSREIKIF
DKDFYFFAYILLGMGWCVSTIALFLELWSFKYSVTNVLHEG
>DmelIR68a
MRCLWILIVAFISLAMATSIPIPIANPAPLSGYEMQLKILLQKILWVANVKRCFAVITDDLHYPIYDRIFFESVGR
RVIPFFVMRTNESDDLQRPSRQVELFVKAIKSSDCELNVITILNGWQVQRFLGYIYDNRSLNMQKKFVLLHDLRLF
ESDMIHLWSVFIDAIFLKRQLDNKYTISTIAFPGILSGVLVMKNIANWELGKGLNGRILFADKTSNLFGTSLPVAI
SEHVPMVLWANATKSFQGVEVEIMNALGKALNFKPVYYKPNQTENMDWTELDGGASVAYGSGNPDGYAQNGTHIDS
MLVDEVAAHSARFAIGDLHLFQVYLKLVELSAPHNFECLTFLTPESSTDNSWQTFILPFSAGMWVGVLLSLFVVGT
VFYAISFLNAIINGNVSSEFFRCLRPNRNVPMDPKIYRRISFRIAISRYRSSKGDRMPRDLFDGYTNCILLTYSML
LYVALPRMPRNWPLRVLTGWYWIYCILLVATYRASFTAILANPAARVTIDTLEDLLRSHIPPSTGATENRQFFLEA
NDEVARKVGEKMEVFGYSDDLTSRIAKGQCAYYDNEFYLRYLRVADESGSALHIMKECVLYMPVVLAMEKNSALKP
RVDASIQHLAEGGLIAKWLKDAIEHLPAEALAQQEALMNIQKFWSSFVALLIGYVISMLTLLAERWHFKHIVMKHP
MYDVYNPSLYYNFKRIYPQH
>DmelIR68b
MKFLVGLLLQWYLPGIYALAEIACRIAVEQNVQVTYLYRCASCPASFDADYSALELDLYRCVGSRLPVITRNMEAH
ELEPFRRTDSLSIFQIPAAEKGDSLVRRILDMLNPHQRRKHMHKYLFVWPNAGRHQLLRLFRGSWAKKLLYGLAIT
GRENGTFDFDPFAWGGLQVIQRLDGEVPYARKVKDLRGYPLRFSMFTDPLMAMPRSPVETAGYQAVDGVAARVVGE
MLNASVTYVFPEDNESYGRCLPNGNYTGVVSDIVGGHTHFAPNSRFVLDCIWPAVEVLYPYTRRNLHLVVPASAIQ
PEYLIFVRVFRRTVWYLLLVTLLVVVLVFWVMQRLQRRIPRRGVIQFQATWYEILEMFGKTHVGEPAGRLSSFSSM
RTFLMGWILFSYVLSTIYFAKLESGFVRPSYEEQVDRVDDLVHLDVHIYAVTTMYDAVRSALTEHQYGLLENRSRQ
LPLGIATSYYQPVVRRRDRRAAFIMRDFHARDFLAITYDSQAERPAYHIAREYLRSMICTYILPRGSPFLHRLESL
YSGFLEHGFFEHWRQMDLITRVGASPDAEEFLEDLGDQTDTDSGSNELAIRNKKVVLTLDILQGAFYLWSVGIGIS
CLGFAVEHAHWFWRRQTLRNAVEARTS
>DmelIR85a
MSIQWLKHILLLAILVNLAGTRENHIPLDLKKSSIVMVKMSQILCKARIKVLFVYFENQTSHEHTGQILKEVTKCD
ISNQNTPLEAVKDDGILMYMVMITTNISQPLELSLIRKKSAAKHRSHVFLLVRDADTVSDAWMRASFRQFWKIWLL
NIVILYWRDGRLNAYRYNPFMDNYLIPVDNKPNEVPTLEQLFPKTIPNMQRKPLRMCIYKDDVRAIFWRQGTILGT
DGLLAAYVAERLNATMMITRPHSYNNHNLSSDICFLEVAKEYVDVAMNIRFLVPDTFRKQAESTVSHTRDDLCVIV
PKAKTAPTFWNIFRSFGSLVWALILVSVLVANVFCYILKSEVGRVPMQLFAGALTMPMTQIPPNHSIRLFLIFWLY
FGLLICSAFKGNLTSMMVFQPYLPDINQLGALARSHYHIIIRPRHVKHIQHFLTLGHKHESRIREQMLEVSDTQMY
EMMRNNDIRFAYLEKYHIARFQVNSRVHMHLGRPLFHLMNSCLVPFHAVYIVPYGSPYLGFLDSLIRSSHEFGFER
YWDRIMNSAFIKSGVKVVNRRRGSGNDEPVVLKLQHFHAVFALWLVGIGMACIVLAWEHLTHNYNLAVTKRRD
>DmelIR87a
MSTPEQRFWLAALLFLLSQHSEVRGFGINLMKVQTEDKGQEACILALLRKYFDSGDGLSGSVLCINRNYQLPNIEE
QLLRGVNNYENYPWSLLITNSREGPSPAKFLMNEKPQCYFLIVDNLEDEDLDEVFEHWKGMVNWNPLAQFVVYLAS
LEETDEEMNDLMVELLLTFINKKIFNVNVIGQSEENQFYYGKTVFPYHPDNNCGNRVISVELLDACDYPSEETDSE
DENDEDEGDGAQEEDDGPQEEGDGEQEEEDGPQEQEDGDQAKGDEGQENDDGGLENKVENEFRIGASDDDELENDL
SSNSSEPEAIIEEFFRAKFEDKFPRDLSGCPLTASFRPWEPYIFRNSEEQPVDDYYYGLQGDEDDYNDTSPNYGES
DDESYADPGEDGDGAIPDTETQSGGKLKLSGIEYEMVQTIAERLHVSIEMQGENSNLYHLFQQLIDGEIEMIVGGI
DEDPSISQFVSSSIPYHQDELTWCVARAKRRHGFFNFVATFNADAGFLIGIFVVTCSLVVWLAQRVSGFQLRNLNG
YFPTCLRVLGILLNQAIPAQDFPITLRQLFALSFLMGFFFSNTYQSFLISTLTTPRSSYQIHTLQEIYSNKMTVMG
TSEHVRHLNKDGEIFKYIREKFQMCYNLVDCLNDAAQNEHIAVAVSRQHSFYNPRIQRDRLYCFDRRESLYVYLVT
MLLPKKYHLLHQINPVIQHIIESGHMQKWARDLDMRRMIHEEITRVREDPFKALTFDQFRGAIAFSGGLLLVASCV
FAFELCYVKYVYRTEKRERKTKKITKKVHNIKIQHD
>DmelIR94a
MALPKQLKFINIFLVLLIIYGSSDGTENQHEIFLNRLLQAVHNERSVETLFLLHHSNLANCSLQDWNPPRIPTIRS
NELTVFNVEKTFNHNALALVCLMKNSYREILNTLAKSFDCMRQERIILMIHRKSDSKFIEDITHEVKNLQFLHLIV
LIVQEKYNGQVFASTLRLQSFPEPHFKRIRNVFAIQRIFYRPINFHGKVLNAIPNDIPILFVALNEMFTEYARRYN
STLRIQNRTIKEDIEITEDNYDIDMKIQLHNSQNFLHHMNIAMDIGSNSLIILVPCATELRGLDIFKELGVRTLTW
LALLFYIIFVLVEMLFVFISNRFNGRNFTMRYTNPLINLRAVRAILGQTSPISNRYSLSIQHFFVFMSLFGTLFGG
FFDCKLRSFLTKRPYYSQIENFSELRKSGVTVVVDHTTRQFIEQEINANFFRDEVPNVRTTTIQELINHVYSYDRK
FAFVANSIPWRTFREEMKSINQKILCDSKNLTILENVPLTFSIRRNAIFSHHLRNFIINAADSGMITCWFKMAGKV
IRKHIKTTLRESEQQPSHLPLSFDHFKWLWAVLCIAYVMSFMVFVMEILWSKYQRRTRSVSIV
>DmelIR94b
MSLIFNLLFILILSQAVSQETEFLQLKYLNNIVRSMIKLHKMETLVIVKHHLDNNCSLQNWNAHGMGIIRTNDQGK
LIMKDTFNSRTLAIICIGQNSHITLLRNVFETFGKVQQKKIILWTQMELKEKFFQEISKKSRDLKLLNLLVLKAVT
KDKLLIYRLNPFPSPHFKRIENIWTPNDTLFMDTKFNFHGMTAVVKHDYNWTIQMGNIRKFPISRIEDKEVIEFAL
KYNLTLQFFNDVERFDIELRKRIILKSNSTQPIDSGIPMVFSSLLIVVPCGNYLSIQDVIKVSGIEKWIFYIILVY
VIFVLIEITFLGVTILISRQSRHQMIPNTLVNLCAFRAILGLPFPETRRTSLSLRQLFLAIALFGMIFSIFINCKL
SSMLTNPCPRPQVNNFEELKTSGLTVVMDHDAENFIEKEIGVDFFNQYMPRKVTLTFTERAKLLFSLKGNHAFTLF
SESFAIIESYQRSKGLRAHCTSEDLIVAERVPRIYILENNSILDRPLRRFIRQMQESGITNHWLKNIPSSLEKNLM
QITIPYDRERVHPLSIEHLTWLWCILILGYSISMIVFFVEMSLKRRKKNLENRAPNICIC
>DmelIR94c
MSKVFKLLVLPLIYLSLTKGSKNPQLKFLRELINVIEEGREIRTIMVIKHSRDEYCHLDQWNPRGSPILRTNEMGS
IRISGYFNDQAVILACMGENSDYGLLKSLANAMDNMRQERIILWSEREPTKMLMDYISQQADRYNFAQIIIVTMNE
DVDAVPSLHQLNPYPTPRFRQITNISNIRRTSFFGCGLSFQGKTAILKESVVSNIRFKVWSPSGPIPLSELKDYEI

VQFAVKYNLSLKLYDQNESKSDHFDIQLGPLFITKDFPTQMAFVSPNTACSLIVIVPCSPKWRFMDVLHKLGVLKL
IGCLLIAYAVFVLIETLILWLTHRISGREVRLTSLNQLLNPRAFRGILGLPFPEFRRSSISLRQLFLVISVFGLVY
SNFVSCTLSALLTKPAQNPQVRNFKELRDSGLITIMDKYTHSFIEKHIDPEFFDHVLPHYLILQKKEALRMIWNFN
DSYSYVMYTTTWKSLNTVQKSFDERVFCESESLTIAWNLPRMYVLGNNSVLKWMLSRYITYMPQTGIPDSWTEQLP
KVLKLLYNVTSPRRIKEGAVPLSIQHLSWIWHLLFIGESIATLVFIVEILLQKSNQHTSNMRERSSED
DDFV
>DmelIR94d
MGQLHLLLVALVLLSPGGDSFYHSLIHHLNRELKIEYVLLLGNFDTTWLDILWQLPVSVLQIKEHSRETYSLLENP
SHNVLTIAFVNDSPEDILEILYRNLRMLNTQPVLLVIRKSTIRVNSLLEWCWHHQLLKVVAIAQDFMESLIVYSYN
PFPVLQFIERRLDNSTVIFEKRLENLHGYEVPIALGGSSPRLIVYRDLEGKLIFSGPVGNFMKSFEQRYNCRLVQP
YPFDESAISPARDLIASVQNGSVQIALGAIYPQVPYTGYSYPIELMSWCLMMPVPEEVPHSQLYSMVFSPMAFGIT
IVAMVLISLTLSMALRLHGYRVSFSEYFLHDSCLRGVLSQSFYEVLRAPALIKAMYLVICLLGLLITSWYNSYFST
FVTSAPRFPQLTSYESIRHSNIKIVIWKPEYEMLLFFSENMEKYSSIFQLQEDYKEFLHLRDSFDTRYGYMMPMEK
WSLMKEQQRVFSSPLFSLQDDLCVFHTVPIVFPMVKNSIFKEPFDRLILDVTATGLLSRWRDMSFTEMIKAGQLGL
EDRGHPKEFRAMKVGDLIQIWRFVGWMLGLATIVFLLELICFWRHKMWQNMKYMFCRNKNI
>DmelIR94e
MDCPKWILSGLCLISLVSGATVIELLGTLKLELDFEYVLLMKNRNFSLSDQVWNGTSLTKDVMDEVQVPVLQFNEN
VSYFLHNSISRRLVTLGFMSDANLDEHRGLLTALVANLRHMTTSRVIFLVQSKASTDFLYELFRNCWRKKLLNVIV
IFQDFETTSTFYSYSNFPILQIEERIYETSLQTLPIFPDRLRNLHGYEMPVILGGTAPRMIAYRNKKGNVVYDGTV
GHFMTAFQQKYNVKFVQPLQAKNPLDFAPSMQTVGAVRNETVEISISLTFPTIPPFGFSYPYEQMNWCVMLPVEAD
VPPFEYYTRVFELAAFLLTLGTLVLISCLLASALSLHGYATNISEFLLHDSCLRGVLGQSFVEVFRAPTLVRGIYL
EICVLGILITAWYNSYFSSYVTSAPKQPPFRTYDDILASKLKVVAWKPEYAELVGRLLEFRKYETMFLVEPDFNRY
LALRDTLDTRYGYMITTNRWVLINEQQKVFSRPLFQKRDDFCFFNNIPFGFPLHENSVFMEPVQKLIMELAETGLY
YHWITTGFSELIDAGEMHFVDLSPHREFRAMQIQDLQYVWYGYAFMVVLSSLVWLLENLAYTVKSKTIFPTHFMQR
NKK
>DmelIR94f
MSGMWQQVLLAETSNWFRSDVLQRFWTHLRVEIRFRTMLNYRLESCDCWFDNVLGSDNSTALLWNDQTYPHYLRRR
QDTDILVVSCLRFHQYQEVLLALSLMLDQMRSMPVVLQLCGDEDSMQELNSARLLLKHSQDLKMPNVVLLSSTFFT
SATLYSYEMFPEFNVQKLVYQAYLTLFPYKLGNLKGHPIRTVPDNSEPLTIVRKTLNGSIAIDGLVWQFMIEFAKH
INATLQLPIEPHPEKSIKLVQILDLVRNQTVDIAASLRPYSLNVQRSSTHIYGSPMMVGNWCMMLPTERVIGSHEA
LTRLMKSPWTWLILLLFYSVHRFLAQKTRLRSSLIHLIKLLINLSLICFLQAQLSAYFIGPQKVNHISNMQQVEES
GLKIRGMRGEFMEYPIDMRSRYASSFLLHDLFFDLAQYRNSLNTSYGYTVTSVKWELYKEAQRHFRRPLFRYSEEI
CVQKLSLFSLIQQSNCIYCYRSRIFILRMHEAGLIRLWYRRSYYVMVTAGRFPIGDLSTVHRAQPIRWTEWQNVVL
LHGVGLLFSVVVFVIELTVHYANVCLNNL
>DmelIR94g
MSTAVNSVHSKLVSLISRGQELTSIFFYAPAKEKCHLEDTISSATWGLPLVIWRTDRTVILNGFIGEGLLVLACLP
GFHWRALLGSLARSLKYLRQARILIELMQDRDEFLVSEVLQFCLSQDMINVNAIFDDFPETENLSSFEAYPSFEVV
NQTFTPDTQVSDLYPNKMLNLRGGVIRTMPDYSEPNTILYQDKEGNKEILGYLWDLLEAYAHKHNAQLQVVNKYAD
DRPLNFIELLDAAQSGIIDVGASIQPMSMGSLSRMHEMSYPVNQASWCTMLPVERQLHVSELLTRVIPYPTLALLL
LLWIFYEVLRGRWRRHSRLQSIGWLVLATLVSSNYVGKLLNLFTDPPSLPPVNSLAALMESPVRIISIRSEYSAIE
FTQRTKYSAAFHLALHASILIGLRNAFNTSYGYTITSEKWKIYEEQQKRSSKPVFRYSKDLCFYEMIPFGLVIPEN
SPHRAPLHSYTLLLRQAGLHDFWVNRGFSYMVKAGKINFTAVGERYEAKTLTITDLRNVFIIYVSVLLISLILFTC
ELFVSWVN
YWLGF
>DmelIR94h
MLSNISFSSAPELVDLYGLVLKFLVSSETTLFYFNPTGQKCSWETLPRTILSNHPQIIWFREETYPGLYKRHSSNL
FVMACLSSTSYDGQLQLLAESLTRYRSVRVLIEVQDKEGSFLASQILLLCQQHSMLNVVLYFSRWTRTLNVFSYLA
FPYFKLLKQRLSGSLRPKIFINQLKDLQGYKIRVQPDLSPPNSFSYRDRHGECQVGGFLWRIVENFSKSLKGDTQV
LYPTWAKAKVSAAEYMIQFTRNGSSDIGVTTTMITFKHEERYRDYSYPMYDISWCTMLPVEKPLSVEILFSH
VLSPGSALLLILAFILFFLIVPQLIKCLGITFRGRLIGMASRIFALVMLCSSSAQLLSLLMSPPLHTRIKSFDDLL
TSGLKIFGIRSELYFLDGGFRAKYASAFHLTENPNELYDNRNYFNTSWAYTITSVKWNVIEAQQRHFAHPVFRYST
DLCFSSETPWGLLIAPESFYREPLQHFTLKINQAGLITQWMTQSFHEMVRAGRMTIKDYSRTNLMKPLRIQDLRKC
WVIFAVGLGTSTVVFTIELLLIYTNVFLNSL
>DmelIR100a
MATTLQLIMLALVGGTLGQANNTDHKQVLTSIVKQLEGGLELHLRTSEDGGNDLVQFLMQEKSSIIISAKQEEVPS
RAKIMRHHFFIFDGVHQMQEIRTSLFNTDGFYILALENNTIEDDVLLMEFAADVWLQHGHSRIYYVQLSKKSVLLF
NPFLQRLVVVQDSKTYSRIYKDLEGYHLRIYIFDSVYSSVIGDGENKVLSVTGADAKLAKTVARQLNFTADFVWPD
DEFFGGRLANGEYSGGVGRAHRGEVDIIFAGFFIKDYLTTHIQFSAAVYMDELCLYVKKAQRIPQSILPLFAVHMD
VWLCFLLVGLLGALVWLILRAVNLILGIEGVPDGSRATRISYFGAARRIFVDTWVIWVRVNVGRFPPFHSERIFVA
SLCLVSVIFGALLESSLATVYIRPLYYRDVNTLRELDESGQPIYIKHPAFKDDLFYGHNSEVYRRLDAKMMLVAEG
EERLIEMVSKRGGFAGVTRSASLQLSDIRYVMTKKVHKIPECPKNYHIAYVLPRPSPYLEEVNRIVLRLVAGGIVG
LWTGEAKERAKWSIQRFPEYLAELDVGRWKVLTLSDVQLAFYALTIGCLLSAIVCMAEILLGRQRRLHSPK
>HarmiGluR1
MVFNTFYATRYTGVLLLNAVILSAQPFAVEKIAVGAIFDQNTEEIQNVFKYAMTIHNQNISSRRLELQAYVDVINT
ADAFKLSRLICNQFARGVFAMLGAVTPESFDTLHSYTNTFQMPFVTPWFPEKVIPPSSGLIDHAVSMRPDYHKAIV
DTILYYGWKEIIYMYDSHDGLLRLQQLYQTMQPGRTAFRIALVKRINNASDAIEFLLALEQYDRWGNKRIVLDCNA
KNAKSILVEHVRKVQLGRRTYHYMLSGLVMDDHWENEVTEYGAVNITGFRIVDHSRKIVRDFMDGLRRMDPRFKGT
ISAETALMYDGVQVLMDALGRLWRKKPDAFRSALRRAAGQANSTKVIDCNPGKSWVVPFEHGDKISRLIKKTDIEG

LTGNISFNEEGHRHNFTLHVVEMTVQSAMLKVATWSDAHGLQVATPRYVQLRSPASYDTNKTYIVTSFLEEPYLMQ
KPIEFGQKEELFGFCKDLMDVIAKKMGIKYKLKLSNDANYASDALPDIHSGVVGEIVRKEADIAIAPFAVTPERER
LVDFSEPFLTLDTPIAYTRTPRQLSDTFSFLRPLSKEIWLCVLFSFFAVSIVLFLVSRFSPHEWKSVSISDTQLDH
TMSSTSEIILHNEFSIWNSFWFSLGSFMQQGSDVVPRSLSGRIVGTVWWFFALILVCSYTANLAAYLIVERIAEPA
LSTVSYSPNIAHTESSSLNFRNNFVKDPVLNEEAISAYSDDGEACGPSRVCRYKHVNFAFATAKGSPLREAINLAI
VNLKKEDFITKLWRKWATYNKKPDCEMIKDEETTITEMTLSQVAGIFYVLVGGLALALGVALVEFCQHGRAEAARA
NVPLRAALRAKARLASRTERKTPPQRTQGDHERLGWNGAAFAGYFTSGTQISQEDAVHASFTHV
>HarmiGluR2
MSRLLLKWLAATLCLLRVHGDRTLGAIFDDGTFLLEAAFNVAIAAASEDQENPFVANVIKTSPSDITEAENAMCTL
LESNVFGVFGPTKKGSLQHIQSIADYLEIPHIITDPVETQNRNWSVINLFPHHLAYSQLFADLIELKGWTDFTIIY
EGAELLPFFDSILSMQDLDTGQKILIKIVQLPDGDDFRSQLKFIKKSGSVNYIINCRRETLPLVLEQAQQVGIMSD
EHSYLIMNPDFQTIDIDPFKHGGSSITGIRMFDPSLESIQNFITSLNEKVAELSENEIENAIAENGLTLDLALVYD
AVTLFVSTLNAMSLEEGSNVTCDDAESWGFGSSIVNYARTMEVDGLTGIIKFDEDGFRSEIEIDVLEIMSYGLDKV
GTWTLEDGFVETKDNVSPAEQEGSESMKGKHFVVLTALSAPYGMLKESLKKLEGNDRYEGFGIELIDELAKINEFN
YTFDIQEDGVYGSYDKKTGKWNGMMEKIMDGRADFAITDLTITAARQKAVDFTSPFMNLGITILYKKPTKEPPDLF
SFISPFSMGVWGWLAGAFVGVSCLLFILGRLAPEEWQNPYPCIEEPETLDNQFTLANSFWFTLGSVLTQGSEIAPI
AVSTRMAGSMWWFFTLIMVSSYTANLAAFLTVESKFYAIKSVNDLASNPYGMTYGAKKGGATFSFFKESDNLLYQK
MYHYMEDHPELQTATNDQGLDRVKSDSENYAFLMESTSIEYMVERNCDVAQVGGLLDSKGYGIAMKKNSPYRQPMS
ESILQLQEEGKLTRMKDKWWKEKRGGGACADDDAGGGEAQPLVLANVGGVFIVLAAGSGMAVVCAFVEMVFDVWMI
SRKMKVSFREELKAELKFILSFSGDTKPVRHRESTGSGSGGSKDDGEKNADAESPDDDDRADPSPTPRSERSGHSH
HTLHSRRQSNAVQMAKMRKYSLRSAM
>HarmiGluR3
MWPGWRWVVLMVNAVAALSPVVKIGAVFTEDARGGSTELAFKYAVYRINKERLLLPNSTLVYDIQYTPSRDTFKTY
KKACAQIQSGAVALVSGVGPLLGNTLQHMSASLHAPHLTVGPFAPENLNNTFTINLYPPKDLLTKAFAEFLSYLNW
TRMGVIYEDYGYGELNILDIAKDGRDMYAVRCRDAKEYRRGLALLKAQQIEHIVVDTDPKRVRQLARAILQLQMNN
ENYHYVFTSFDFELFDMEDFYYNRVNMSGWRLVDRHSDKVKESLQVMEKFHPIGASIISGGHIKTEPALLYDAVQI
LCQALAITEDIHPGNVSCDKDTPSLHGKAIYDNINTIQAHGLTGPLEFKQGIRKNFHLQLMRLTGGEKGGMVVSGT
WSPAEGLAITDPAAYTRDPPPNVTLTVVTVEEKPYVMVKEGWNLQGNARFEGFCIDLLARVAARAGFHYRLRLVPD
NMYGARDPDTGHWNGIVRELMDRKADIAVASMTINYAREAVIDFTKPFMNLGIGILFKVPTSQPTRLFSFLNPLAI
EIWLYVLAAYILVSFTLFVMARFSPYEWSTSTHVCGHETKLLTNQFSVCNSFWFITGTFLRQGSGLNPKATSTRIV
GGIWWFFTLIILSSYTANLAAFLTVERTVLPIQSAADLAAQHHIHYGTLNGGSTMSFFRDSNIDIYQKMWEHMSSA
SPPALVSSYEEGVRRVLAGNYAFLMESTMLDHRVQRDCNLTQIGGLLDSKGYGIATWKGSPWRDKISLAILELQEK
GVIQILYDKWWKNTGDVCNRDGKDSKANPLGVQNIGGVFVTLLCGLALAIVVAILEFCWNTKKNASQGRQSLCSEM
GQELRTAMRGGSSSRTVLRPGCSRCSPATHVPPATSRYQHSRSSSVELKELRWS
>HarmiGluR4
MRGANAIFLILFFGHLSALPDTIRIGGLFHPEDEKQEVAFRYAVERVNADRAVLPRAKLLAQVETISPQDSFHASK
RVCHLLRSGVAAIFGPQSAPAAAHVQSICDTMELPHLETRWDYRTRRESCLVNLYPHPAALSRAYVDLVRAWGWRS
FTIVYENSDGLVRLQELLKAHGPSELPVAVRQLPDSHDYRPLLKQIKNSAESHIVLDCTTERIRDVLQQAQQIGMM
SDYHSYLITSLDLHSVDLEEFKYGGTNITALRLLDPERADVQRVVRDWVYDEARKGRKLQLGHTTAKENMTFIKTE
TALMYDAVHLFAKALHDLDTSQQIDVRPLSCEAEDTWPHGYSLINYMKIVEMKGLTGVIKFDHQGFRSDFTLDIIE
LTRDGLQKAGTWNSSEGVNYTRSYGENQKQIVEILQNKTLIVTTILSSPYCMRREASEKLTGNAQFEGYAIDLIHE
ISKILGFNYTFKLAPDGRYGSYNRETKEWDGMIRELLEQRADLAIADLTITYDREQVVDFTMPFMNLGISVLYRKP
IKQPPNLFSFLSPLSLDVWIYMATAYLGVSVLLFILARFSPYEWDSPRNCLDEPPVLENQFTLLNSLWFTIGSLMQ
QGSDIAPKAVSTRMVAGMWWFFTLIMISSYTANLAAFLTVERMDSPIESAEDLAKQTKIKYGALKGGSTAAFFRDS
NFSTYQRMWSFMESARPSVFATSNKEGEERVVRGKGAYAYLMESTTIEYVVERNCDLTQVGGMLDSKGYGIAMPPN
SPYRTAISGAVLKLQEEGKLHILKTKWWKEKRGGGSCRDETSKSSSTANELGLANVGGVFVVLMGGMGVACVIAVC
EFVWKSRKVAVDERKEEASLCSEMASELRSALKCPGGGAGGGGGGPGGARDGAGSPYLHYGFSTKSQLH
>HarmiGluR5
MAKNKGTRMWSYHVFLAVVYCGGQLVTADIDRRRFSNPTYYNVGGVLSSNESIAFFKDTISNLNFKDQYVPRGVTY
HDYSMLMDPNPIKTALNVCKDLIAHRVYAVVVSHPLTGDLSPAAVSYTSGFYHIPVIGISSRDSAFSDKNIHVSFL
RTVPPYSHQADVWVDVLKHFNYMKVIFIHSSDTDGRAILGRFQTTSQSVDEDVDRKVVVEQVIEFEPGLDSFSDRL
MDVKGAQARVFLMYASKTDAEIIFRDATFLNMTTVGYVWMVTEQALDAANAPEGLLGLRLVNATNEHAHIQDSIYV
LASAIRDMNTSEEIHAPPSDCDNSGSIWTTGRLLFDYVRKQRLENGATGHVAFDDHGDRVHAEYDMVNVRAQGEHV
AVGKYFYSKETQKMRLELKEQEIIWMGRSTSKPEGFMIPTHLKVLTIEEKPFVYSRRIDDGSECTPEEIPCPHYNA
SEDTDQLYCCKGFCMDLLKHLSKAINFTYSLALSPDGQFGNYIIRNFSQPGAKKEWTGLIGELVYERADMIVAPLT
INPERAEFIEFSKPFKYQGITILEKKPSRSSTLVSFLQPFSNTLWILVMVSVHVVALVLYLLDRFSPFGRFKLANI
DGTEEDALNLSSAIWFAWGVLLNSGIGEGTPRSFSARVLGMVWAGFAMIIVASYTANLAAFLVLERPKTKLTGIND
ARLRNTMENLTCATVKGSAVDMYFRRQVELSNMYRTMEANNYDNAEQAIEDVKNGKLMAFIWDSSRLEFEAAQDCE
LVTAGELFGRSGYGVGLQKGSPWADLVTLAILDFHESGIMESLDNLWILRNNMLNCEENEKTPNTLGLKNMAGVFI
LVLAGIIGGIVLIVIEVVYKRHQIRKQKRMEIARHAADRWRGAVEKRKTLRAAILPSQRRAKSNGVKEAGSISLAV
ERGARRRDEPRVPRYLPAYTPDVSHLVV
>HarmiGluR6
MHVLVPLLLVCVSQCVSAQEGPPIGGIFYKDSEDMKAALEVSAKSFNFTASIKEVSTRGEVLEISKYVCQLAEEGV
IGIIDGTGGRSSEIIQGLCDALELPHISIEHNDLYSDDWFVLNMYPSPTAYNMVLQKLILHKEWKNFTLLYTKGHS
LIRVSELLQMGNDTLVVSLRELSGSDYRDVLIDAKHNGYKNFVVDSPSRYLEQVLLHAQQVGMMAEEHSYIFVSPD
LFTLDMSRFKYGGVNMTGFRLVELQDKDNEKLWNFTSTLNLETGKTFKPEQLKTQVLLIHDAVEVFAAAFKKVKVQ
PEALSCDNYQAWSFGSTLLNFMKTNKVEGLTRSLIFDGVGQRTDITFNILELTSAGNQSIGNWTNNELKINRPLVA
DAEITQESALRNKSLRVLISLAPPYGYMRKSDKKLEGNDQYEGFTIDLIDKLSEILGFSYEFAVEDDYGTKTESGE

WTGMALQLREERADLAICDLTITAVRQSGIDFSTPFMTLGIGILYKEPSKQPPEMFSFMAVFSKEVWYYMMLIQLA
LGATMIFVGRISNKEWQNPVPCIESPEELNNQFSFANSVWLIIGSVMQQGSEIAPIAIGPRMITSVWWFFTMVMVA
SYVGTLVAFLTVEKNVLPFETVQELYESKSITYGAKEKGSTKQFFENSTNPIYQAMFKKMKAHNWLAKENDIGVYW
AETQNYAFFMESTSLEYYKERHCDLLQVGGLLDSKSYGIGMKKKSPYKKYIDDALLKLKENGEIEKLRNIWWKEKR
GGGKCGEKRDADQKQLGMKNMLGAFVVLGVGCLIGLFISIIDMLWGVFKRSVKYSTTFKYELIEELKFALKFSGHI
KPVKRPQKAIDGSFEALAKAEGKDDIRSLHSIRSCDTHRTHHSHSSRHSSRSLSVAFAKRRSYS
>HarmiGluR7
MCWYLLVLLLCVQKCTPQFVYTEMTEISYQIVGIFEKDATIQMAAFNDSLNHVHVQEVTIRPATLQPRRTDSYSVW
RELCSNNAIQAVAVFGPQNPITDGAIRDQCAIANIPHIQATWQSMDPDLELNEETTAQEEGEDEESEIPFKKISIN
FYPDSEEIALAYGKLLQYYKWGGFAALYEDNFGLLRVQKILAEVSLQSQIFMYKLDPKGDNRRIFKALRKFQVSRF
LLDCHSDHILRYLGEADNAKLVSEYQHFVLVSMDTSTVAESLIKMPSNITWLSITQYDKLKDGGHYLATRVGNWRS
NEESPSVVQFKLDSLIMDDVASHLVKALDVKDIAQPPVFSCTGDEEPWAHGAAYQQKILKTQSYGVTGNVEFDQRG
RRINYVLYINEIHIRDRQTIGRWESATGMINETKRLDSSAANQQSSKEFVVISRRAKPYFSFKEKCEKTECKDDDR
FEGFSVDLVDNIFRILREEKYNYTYRFIHDYDMEYGKVDPTTHTWTGLVGYLLDKKADLAVCDLTITEERKKVVDF
SVPFMSLGISILYTQDRKVQPGMFSFLNPYTFEVWMHTATAYCVVSIVLFICARISPADWENPEPCEKDPEELENI
WTFKNCAWLTMGSIMTQGCDILPKAIGTRWVCGMWWFFAVIVCQTYIAQLSASMTSALENEPINSVDDLAKQTKIR
YGAIVGGSTLEFFKASKDKTYRHMYETMAANPAVLVTSNDEGEERVLKSKNTYAFFMESSTIEYKLKRNCKLKKVG
GELDSKDYGIAMPANSPFRTDINRAILRLKELTTLDKIKNKWWHEKYGAQKCEPTVDENDIEGDLEMENLMGAFVV
LVVGLVFCLFITAIEFMNEVRNIVVREQVTHKEVFIKELKASLNFFQLQKPVLRNPSRAPSIASSDSDERRENQAK
AIENFMNLEKAV
>HarmiGluR8
RTSPAPPPPRAPSSITAALVVPHKAFGTRDYTKAEKAALSKLPRKLKLFSHVRLNITLSTQGLTPSPMSILDSLCK
EFLAVNVSAILYLMNHEQYGRSTASAQYFLQLAGYLGIPVIAWNADNSGLEKRASHASLRLQLAPSIEHQTSAMLS
ILERYKWHQFSVVTSAIAGHDDFIQAVRERVTALQDRFKFTILNAIVVKRSSDLNELVTSEARVMLLYATREEAAE
ILSAAGDLHLTGENFVWIVTQSVLGSMQQPNKFPVGMLGVHFDTSSSSLIAEIATAVKVFAYGVESYVSEPENIRY
PLGTRLSCSGAGAGEARWSTGERFYRHLRNVSVEGEAGRPSIEFTPDGELKAAELKIMNLRPALGEQLVWEEIGTW
NSYPRERLIIKDIVWPGGLHTPPQGVPEKFHMRITFLEEPPYINLAPPDPVSGRCSLDRGVICRVAPEVEVAGLEA
GAAHGNSSLYQCCSGFCIDLLQQLAEQLGFTYELVRVEDGRWGTLHHGKWNGLIADLVNKKTDMVLTSLIINSDRE
AVVDFSVPFMETGVAIVVAKRTGIISPTAFLEPFDTASWMLVGAVAIQAATFSIFFFEWLSPSGFDCSTGNNSKRV
PQNRFSLCRTYWIVWAVLFQASVHVDSPRGFTARFMTNMWAMFAVVFLAIYTANLAAFMITREEYHELSGLDDPRI
ARPLTQRPPLKFGTVPWSHTDATLAKYFTEPHAYMARYNRSTVSAGVTCVLTGELDAFIYDGTVLDYLVSQDEDCR
LLTVGAWYAMSGYGLAFTRNSKYVSMFNKRLLDLRANGDLERLRRYWMTGTCKPNKQEHKSSDPLALEQFLSAFLL
LMAGILLAALLLLLEHVYFRYLRSHLAASSAGSCCALVSLSMGQSLTFHGAVVEAAARGFGERSHCRSAVCAAQVW
RARHERDMAVARARQLA
>HarmiGluR9
MATRDGTRDLKLLSKNTSDSGEAAGAPHAAQTTYCIHKPTPASTMITLKAFIIAISTVCLFIHNSHALRLTAFPIG
GLFNRETLPSSLQVFSNMIEANRMTTYHGRSLVSKVVDSYSTSLELCPFTSEDRGIVALIDARPTYGICDITCLLC
NRLNITHLSLGWEPTDTQSEDFFSFAYYPPPEMISKAYATLIKDLGFDKFTILYEDDGSFIRLQQVINTWPTTMEP
ILFRKLDPYGDNRETFKYIFKVARMSYHVLDCNITNIHKYMNEIVQVENATEFQSFILTNLDAYSLDFKAIDDLMA
NVSTLHLTTPSEATWKDKGMLGARAFGLETALAADALSHLEKAIRSLQLTTDNLPDPPPLCQKSTRSEYEESAWPL
GYDLREALIKTTTKGFSGHIEFDSEGRRTNFMLHYSKLDKESQFVYAGDWDYKTNVITKKDHVDDRSLALKPGSKI
RVVTKTGSPYFSVVDTPEGKVYRGYCVDLIDAIFKYIKETRKEHFEYEFYIAPGNEYGNQIEGTNKWTGIIGELMD
HKAHLGICDLTITSERNSVLDFSIPFMTLGISLLFREEDPEAPDRFSFIKPLSLDVWLYLATTYVIVSFVLLVCAR
MSQDDWVNPHPCNQNPENLENIWSLYNCMWLTMGSIMTQGCDILPRAAGSRWIAGVWWFFALIVTASYTANMSTFL
SNSRRSNVINDVKELSEQNKISYGAVYNGSTYRFFQTSNDTVYSKIWSVMNAAKPTVFTTSNLEGKDRVTRSNGKY
AFFMESTSIEYYIKRNCTLKMLGSKLDSKEYGIAMPKNYGFKGKIDHAILSLQELGELEKLKKKWWEDEDNQEHCE
KIVKEEDDNGSLQMKNTSGIFLVLGTGGILGLIVAIIDFMFHARQISVKEKVTFKEALSSEWHASLNPRELHKPTA
PPRSAPPSTASPSPQRERSQSRAVSVLRAASSFINFDEIY
>HarmiGluR10
MLLRGVYVTVVTIILILGNVGAVLRKFETLKTTVNIGAIFPPNTVAEVAFASALARASMESEHYHFVMKAVYSPYG
DSFAASKAACELLSGGVIAVFGPTDPVSASAVEAHCRAARVPHIQAVWRPPPVRGEQQPSPPGINLYPEAVALSKA
VALFIDDSDWRSYTLLYDDDHGLIRLQEVLKHTNPEVKWLVRRLVPGEDNRPLLKSLKGTGETRVILDCPADRVLE
YLRQANEVKFFEDYMSYVLMSLDAHTLDLEELRYGLSNVTCLRIFDHSDSRTRAYLADWKVRGSDDVKIPRQSYEI
TVEAALASDAARLITDAVENAPEEFKLEAQEISCDSEDQWESGEDFTNHLLTNPIVGITNKINLDNTTGERMNFSV
EIMELSNSGFNSIAKWNPEVGFEYGRSADETSDRLAEKWQNKTFKVVSRIGAPFLVEVVPKEGETLTGNDRYEGYS
KDLIHEILKELLHLNYEIEIVPGNGYGSYNKETKKWDGLVGHLLERKADLAICDLTITYERRSSVDFTTPFMTLGI
SILYLKPTPPEPELFSFLKPFSVDVWIYMAAAYLMVSLLLHILARLAPNDWENPHPCDKSPEELENIWHIKNSCWL
TMGSIMTQGSDILPKGYSTRWVCGMWWFFGLIMCSSYTANLAAFLTNAAMDDSIKSAEDLAMQSKIKYGTLIGGST
YSFFKRSNVSIYQRMYGAMESARPSVYVKNNDEGLERVLKGKRDYAYFMESTAIEYQLERHCELMQVGGLLDSKGY
GIAMPFDSSYRTAVDNALLKLAESGKLVEIKNRWWKAPPEKACVTEEASEEGAAGELGVENVGGVFVVLGTGCGMA
AAMGIFEFLWNVREVAVEQKMTQSEAFWAELTFALSFWETEKPVKHSRPSSSASESQGASRASSVLRSAADLFHLD
VFK
>HarmiGluR11
MMALKFILNTFKYILLLSYLAIYCNNIEVSSQIPMADKIPIGVVFDQNTEEIQNAFKFAMLQHSNANKSSLDFQLY
VDIINTADAFKLSRLICNQFARSVVAMLGAVAPDSFDTLHSYANTFQMPFVTPWFPEKVIPPSSGLNDYAVSMRPD
YHRAVMATITHYGWKNVIYIYDSHDGLLRLQQLYQSLQPGNATFRISNVKRVSNASDVVEYLRAIEKLDRWSNKYV
VLDSTTQLAKATLILHVRDVQLGRRNYHYFLSGLVLDDRWEKEVTEFGAINITGFRLLDFSRKIVRDFIDVWRRET
ISAQAALTYDAVQVLIDAVLRLMRKKPDILRATLRRASQNNSKIMDCNPKNKLIPFEHGEKISRMIKKTEVDGITG

SIRFNEQGHRKNFTLQVMEMTVDGDIVKVATWCENKGLIPVIPKLDHPAVPGSYDRNKTYIVTTIEEPPYIIREDP
EDPEFRPDEPFKGFCADLTKMISEKLEINYEIKIVRDGKYGNEDPKVLGGWDGMIGELIRKEADIAIAPLTVTLER
ESVIDFSKPFLSFNLKPNVASNSTGAIFSFLQPLSMEIWLSIVCSLFAVSVVLFIVSRFSPYEWRVVSFTDSHITE
HSDLATTKTTVVNEFSFWNSMWFSLGSFMQQGSDINPRSVSGRIVGCVWWFFALIVISSYTANLASYLTLSRISEP
SQTYSKVAMCPEDTVDGPKPTIEVPQEPPVDEHGWLAFLMDRSAEPEDKPCEMIVTLTNSGYKDFAVGVPKGSQLR
DGVNMALQSLKEEGEIPRLVRKWFTKSECDAPDTDIKGSELTLSQVAGLFYVLVGGLTLAMAVALFEFCKHGRAEA
ARANVPLRAALQAKTSLSSNTERKMTQSQRGPQREHDRLGWNGGAFGGQYYSPATQIGQEETALHSSFTQV
>HarmiGluR12
LPLLLVDAAPADAAWEALELYPHPQVLAQACAELCEAKGWTRAVLLHTGDARAAALLTARARPLALLARRLPPADD
DALLRNLLLVLKKSGLTNFIVWCDAACAVRVLDAAQRVGLLAERHSYIVLSLDLHTQPLHDYSHGGANVTGLRLFD
PEAPEVVEVMEQWRKMYAERLGVAADDEGDGDSPEAEEAAAIAAAPPTALLLAHLGTELVAEAWRRLQLPPAAPAD
CAMGAGAFHADTLLNYLRSEEWSSEDGAGRLVGGAVAWEVDGARREVQLQVVELARGGRLARAGLWAPRAGLSWQR
RDPPASDPPPDSMTNRTFTVLIAKSDPYVMMQESTERLTGNDRYEGFCIELIDQLARLLQFNYTFVQQEDGDYGSK
DNVTGKWSGMLGRLMEDKNIDFAVTDLTITAERERAVDFTTPFMNLGISILFRTPKQPEPKIFAFLLPFSNGVWLC
LGFAYLGTSLLLYVVGRLSHEEWQNPYPCIEEPPALENQFTLANALWFNLGAVLLQGSEIAPVAYSTRAVASVWWL
FALVITSSYTANLATLLAKKSSDQVITNVQELADNQLGIDYGAKHGGSTYTFFEHSQSELYQRMFEHMRTRKMPAS
NVEGIAKVESGKYAFLMESTSIDYLTERNCGVTKVGSLLDSKGYGIAMKKNSPYRQAMNLGLLNLQEAGTLREMQH
TWWKEKHGGGACKVSPDSPPPHTPSQEPVLMLLCVQVDEEHESEELNMSNFLGLWLVLVVGSALGIVLSCCDLAWA
AARRARASGRRFHTHFWEELRFVFRFEQSVKPLQGPLSGTPSSSARSEAAEAEAEAEAEAEAEPEETPEPRARSGS
GAAGARRRRSSMHAASLRLARHTNRDSATPARR
>HarmIR1.1
MHQARLRKMILIGVLFGLMQAVSGFNLDVANLAADYVKHKDLRHVCYFTCQSRYYNTILVHKLTKQSVRVSVRRID
ESVNRDVVRAASRSTGAVGLLLDAHCRGTPLVLLEASKNKLFDAMHPWLILTNIEDADNCTDYIQQSFQQLNLSVD
ADIAVASYNGGDNYTLTDVYNFGTIQGNNLEVNHLGSWRPETGLEIKLKGYKYYNRWNFQNLTLRAISVIVDQPEM
FYPEMLSEMTYTAGVAAMTKITSQMLNTLKEQHNFRFNYSIAGRWIGSPKRNSTLAVTNALFWEEQDLSSTCARIF
PKWLDWVDIIHPPTTNLQTKFYYLIPETGVGQYENRFLTPMSHGVWGCAFIAGIACTLVLTGAAWMESRPKPGLYA
FFSVFAAVCQQGYEDGVQLLESYSSQGRRLTLLVIGLTSMLLYNYYTSSVVSWLLNAAAPSIGNLDGLINSDFELI
FEDIGYTRGWLDNSGFFYYSGFKNVKEDELRDKKVTKAKRTVPVLQTVNTGVELLRTGKYAFHTEPYTAAQVISKT
YEDEELCNLGALQMMLPAHVYIMAQKRSPYKEFFDWSLLRLLERGHVKAIRARFAGTMPACSGARPRALALGQAAP
AFLMLLLCVLLSWIILAFEVLWSRVQLKKRGP
>HarmIR1.2
MISYQILLVLILPCIIFGLNQNTLKLGIEYFKYRDVKFVCLLTCEKYSSWALQYSKSASTVSIAVSETSILRSKVN
YNRVENCLRRKTYGLGVIIDTNCEMAADALYFASQNMWLDSHHKWLLIDDDKAKLEVYNDTEIENVIYEDRNSTLI
DILSNLNISVDADIVVAEKGNSSYNLYEVYNYGKIQGGNLIVNEIGFWSHENGFNLNINLNGYKYYRRWDFQNISM
RMILVAQRASKNFDLESLTGPEPVPGVAIITQTPTDILYIVAKIHNIRYVSTITDRWIGSYEKNSSKVVSTSLYFR
EQDVSPVIRGLSTVYERIDVINPPLTSIETRYYYRIPTMGPGKFENQFLRPLSTTAWWSVIGVSTLCAGLLLLSAL
LEQRPSSVQYAVFSVVASLCQQFFQDIDDSGTKRISTARKVTILVTGLSCVLLYNYYTSSVVSWLLNGPPPSINSL
KELLESPLELIYEDIGYTRSWLQSPSYYFNKRNAPIEDELRQKKVFNKKKNAPLLEPLVQGIKMVQKGGYAYHTEV
NSANALISRTFSQSELCELGSLQSMEKTLLHPCLQKHSPYKEFMTWSLMRLSEQGIVSCIQIRRSSFEVKCEGSSP
RALALGGAAPAFILLLGGYMLATVIMLVERLVFKMKHENQVK
>HarmIR2
MRTANVVKSSLDTIFIIIIHKILLLSAYDNADICKLVSIDLLSGITKYFYTSKKFQYKEQFKIGFLRIKESGLLNR
LVSTDFEEPKCTKSHIIQISMQHIAIPLTILAVTSVLSTIIMVAEKIHYNRNMRWPYFN
>HarmIR2.1
MALVNMTNVTLRVATQYNCDSLRNRARTSDPHTFDPMWRETILGCMILDLLRDMYGFKIPWIAATTAACGLRQSSA
ARDNSQPMKMYGAAAEARDSRARPTNFGCCIFFSMCYVFDNPERNVTVIGGLNNASQSYRDSVDVYSRSMTLTQDL
AEQIYPIHALHTWKLGFLLSRPGNKIFSTFYSKPFSRPVWNCLYCFGLLIIVFFYILKRWEFSVIGGWQICFVYEA
LLVVGAYCQHIPPIDPRLPSRRIAYLIFFTFVYIVYTYYTSNLLSNLVNDKDHGIDLPMLADSDYVFLAVNHMMMA
IFERSQIYHYNRNISLVVKKLMRIHTVSIPDGLEAVKTGKYALLSDFITVYPYMKKIRTLNVTQARPLVAFPVKQH
FGALGRVVVVSAASAELSLDRVFVESSCCTHLETALRHV
>HarmIR7d.1
MEVRDTEVNHTLEHFVTEDPASELGILAAKVAYYNFEWRFLTIVMYNTVQAIGLNTFLMHYEKSVIVKLGRFLPAR
RSAVPQMIIFGEDASEISSTIRWTVRAKYDSNGKFIIICAHHEQECDELKIFQTLQSLYMFNAVVLKTSNKTKESL
AYSYDFLSEGKCKNSVPYKVNLTTDCFNDNCFKNLYPERLSNFRKCPLIMSTIEQPPFMYLHNLTSKPTGIDGDIM
RLVADMLNATLHLKPPYDGADSGHFANNNWTGSLGDIYNNHSHASVCSAPITSGKYGNFQISFTYYSMDIVWATRL
PAQQAPWQKLLHPLNIYIRIILLLMFICIIFMNTFCKTNLFKVISKIRDVFKIAPPKYSLLFYSWVVFLGVPILRT
PERRSFVVTVYTWIWFCFIMRSAYQAALMNSLKNPAYLDNLKTFQEVLKENYPFGGLDSLKEYYIDDRAIYDKWKV
VELKNLDKTLDDILEGSTDFVLASNKEFIKHHIMKYNGTKQLQIIPQKIVNSPTVVYFKKFSPLVPPMNFALRIAF
EAGFIQRTYVRYLDHDKKLLQRLRSKQAEPLSMEHFAGCFVLLVLGWFVSLTYFAVEYICGNLDDE
>HarmIR7d.2
MSNQMLYGHLTANMAISELNHFFDTNVTAGSQLGDMAAKVAIQNFDLRHATMLFFNSTLCYGVEVFLQFYHYNIVI
NRAKVLARKTTRQFVLFASDTADIELLLDSIISFEMDNTGKFIIICESSTPKECDEQDIMVLCWNYRIVNMVFIRQ
EETEAVGFTYYPVADGICNNLKPIKLNSHNHYTKTTYGEIFRKKFRNLNFCPIIASTFIQPPYMYIKNGIPTGIDG
DLLRMLIHGMNASLKMMTPSRGTGWGFREKNGTWMGSLADVYDDLANFSMTSAAITLTRFTDFQISSGYSTSKVVW
VSESAQVQNVALKLLHPFEKNTRFLLIASFLLVICCAFVLKSSCWTAMCNEENQSSRSVVFYSWMICMGQAVEKLP
TKSAYVQMTLFFIWYCFLVRTAYQVYLISSLKGRFYDSQFESIDEAINAQYPFGGGPALKDYYVDYPFVYKNWVNI
DTQQIAPTAVNISKGMNFVLAMNIDAARVVMKTRKAKLHILPEPIIISPTVLFFKKYSPLSETVNTILHRLIAAGF
PDKLYKIYSSTFDIVDQGSDEGETLKMSHFTACYVVLILGWIVSAIFFSLEVYFGKIYKHPS

>HarmIR7d.3
MIWVLRILVILQLMLRSCSASSNLIETAVNISTTTFNLHFTTTVIWKQNESECVTGYLQSYPGSVVLSPWATYNDS
KVREINETIGFKQTIYFATNLLEYEIIMELINEVIRFPIRFILVLENPVKSISELSAFIEVTARNDQADLILISEN
DAGEVSLSTFFPYSEGLCGNYTPVFLKYGDDLWPKKFSNFYQCPIRTALLEYFPYVTVHFEKGKITSVGGYDGKIL
MIILKKLNASLEVTSAYNNVFGTYVNGTATGSIGDLATEKADILIPADILTEKRYTVTLPSHTYHTVDIRWVGPKQ
REVYDWLKFIIPDKTNFTYLHLLVYILFVIVAMLVRKCKPHLTSATNRILYQSFIILLGQSARFVTKSWLLNSLFV
LWIWFCFFFRIDYQADLVDALQTLDLEPPFESIEEAVTKVDGYGGVEVVVDYYKDTPLERNYKVIPMNELKNYIRR
IVEGENFILATDIALVKLLEPYVQILKKRISATGACFYMRPGWPAAKDVDDVIFSLVEAGFIENLLSDNNNHRWIV
NRMNADDVLQPKSLSVEKLSTCFYGLGIMWLICFIILLIEIVHHNKHHNK
>HarmIR7d.4
MFVDFSKTLYFAIFFLKIFSCSALNDITQCAVEVANYNFFYRLPAVILYRDVNDKLVTDFLKAYKGTVMIESRGVE
PAKQVVIIVDNYQSFIRLLSMLKPDLRGRNLLNGRVKLLIVINSNRRKLDHINGILWSYYVTDVVIVTKDKQGRIA
LYTYYPYKNHLNCQNTEPVLIGYWSRNMSYSNMYPDKMTNMHECPLYISTNKIYDQATERKIPLQTIKKAIVRLLR
DIMNFTPIVSARDYISIDSDRAKNWSDSLNDVISGFANISTCTIPLGVDRLGLLDYSMPYFRIRIAWLAPPVAPGP
VWWRLLSPLNGYLWLILLVVTFLVISLPFVLKLKRIKYFCHRYFKNFDKVQGAAFRVWGALLGQTIRVAPRRFRDF
YIVGLWLWFTFVVRNAYQSVLIGALKTDTLTGNFANLKETVDNGYNFGGRAGIYSHFEFDPLIRDGFEIIPEVKFE
EVFRDLIDGKKKFVLAASLEYAYAYCLAQGKKENECGHVLPDSILTVPLVVWMKMYSPFVRPLTTWLPRMIESGLL
EKDAVLKTSYVTTITSDPSPLTRHQTLSCFLCLGFGCLISLVILLLEIVRKKTSEYTVVKKSQLVELDRESKFVID
F
>HarmIR8a
MSFHYLFLLIFLINLGCVLSELSLRFVFIIESQEQDLTHEISKSLKLAESVRTDVKLDDAIVVLDRETEDESYRML
CSSLSKGVSMIIDLSWTPWEMAEDLAAETGVPVVRTLLGSQQLVKALDEYLESRNATDAAIILESESDVDRTLYEL
LGASNIRVWVHAGLTRDSAKALKTMRPEPSFYVIVGENGFIMDTYRRAVKEKLVRRDYRWNLVLTDYSTLELSQLV
LPTVTLQPDPGECCKLMKREDCSCPNDFQRKQYILNALIQYIAEVYSKLDRDLPLVTSSISCEEPEAVMNSTRDRL
YRQFAEDAEISNETLFYWDMDRSGLFLRSRFILSTYKPTAGQQTIATWSADEEYKLLPGVELEPLKMFFRIGTAPA
VPWTLMKLDPETGEQMYDDDGQPLYEGYCIDLIARLSETMSFDYEIVSPKSGGFGKKLPNGTWDGVVGDLMRGETD
IAISALTMTAEREEVIDFVAPYFEQTGILIVIRKPIRKTSLFKFMTVLRTEVWLSIVAALVLTGFMIWLLDKHSPY
SARNNPHAYPYPCREFTLKESFWFALTSFTPQGGGEAPKALSGRTLVAAYWLFVVLMLATFTANLAAFLTVERMQT
PVSSLEQLARQSRINYTVVEGSSVHQYFINMKFAEDTLYRVWKEITLNATSDQAQYRVWDYPIREQYGHILLAINA
SEPVPDAKTGFQQVNEHTDADFAFIHDSAEIKYEVTRNCNLTEVGEVFAEQPYAIAVQQGSRLQEELSRALLELQK
ERFLEQLAGKYWNESARQACPDADESEGITLESLGGVFIATLFGLGLAMITLAWEVFYYKRKEKNKVQTLNTKPEK
VAFESKSTLETKVAESVAKLKKRGKKGNLAKNVTFGDTFKPVAEKGVSYISVFPKDYRP
>HarmIR21a
MAVSWFIVIFLLFYPVYGEEVLVEYYPSQSILDTQHKVKNKREIKSNAAAIALDNSNNVSTREINWRKFNQEKTDD
DAKIKKRALDPVFRGHPKTREELWNEHFLNETTKFDQTPSLVHLLHNITLTYLKDCTPVILYDSQVKSKESYLVQN
LLKGFPMSFVHGYINDGGELVERKLLHASTDCQNYILFLTDIKASAKILGKQPKNKIIIVARSSQWAVQEFLASVV
SRMFVNLLVIGQSFKEGDDANLESPYILYTHKLYTDGLGASQPVVLNSWNHGKFSRNVNLFPPKMTEGYAGHRFLV
AAANQPPYVFRRIKADLDGGNPRVAWDGVEIRLVKLLAERNNFSIEIIEPLELHLGSGNAVAKEITSGRADIGIAG
MYLTTDRIRDLDVSQAHSQDCAVFITLMSTALPRYRAILGPFHWHVWVALTFTYLFGMFPLAFSDKHTLRHLLHNS
GEIENMFWYVFGTFTNCFTFLGKNSWSKTDKITTRLLIGWYWIFTIIITSCYTGSIIAFVTLPVFPETVDTIKQLL
AGFYRVGTLDRGGWEKWFLNSSDPQTKKLLNKLEFVPNVEAGIRNTTKAFFWPYAFLGSKAELEYIVQANFTATKS
KRAALHISNECFAPFGITIGFPNNSVYSEKMSLDISRMIQSGIIDKIADEVRFEMQRSVTGKLLAAGSGTIKIPSA
EEKGLTLEDTQGMFLLLGAGFIIAATALVSEWMGGFTRRCRFQRKVDTPISVNSREHLIPTPKTDIGSEIKIIGDT
ESRLHFDSRPSTAASRDTLEGQIINVTEDNIDVHNSFNVDRFDSRRSSSLDLDREVREIFEKDQKRRRIVSQDMES
VDEHGPTVSRVAFGDPIKHEK
>HarmIR25a
MSTVTVLLLFNLVHIAFGQTTQNINVLLINEESNALAEKAFEVAKEYVRRNPSLGLAVDPVIVVGNRTDAKSFLEN
VCRKYNDMLLAKKTPHVVLDFTMTGVGSETIKSFTEALGLPTISGSFGQVGDLRQWRTLNANQTRFLLQVMPPADI
LPEAIRAVVTKQDITNAAIIFDEFFVMDHKYKSLLQNIPTRHVITPVKSFEANEIKTQLESLRNLDIVNFFIVGSL
RTIKNVLDAADKNQYFGRKTAWFALSLSLEKGDISCGCKNATIVHMRPTPDANSRDRLGKIKTTYSMNGEPEITSAFY
FDLSLRTFLSIKSLLDSGKWPNDMKYITCDDYDGKNTPNRTLDLKTAFQEIKETPTYAPFFIPQDDPMNGRSYMEF
STDLLAITVKDGASISSHSLGSWKAGLSSNLTLTDPNNMSNYSAQLVYRIVTVEQKPFIIRDDKAPKGFKGYCIDL
IEEIRQIVKFDYEIILAPDGNFGTMDENGNWNGIIKELVDKKADIGLASLSVMAERENVVDFTVPYYDLVGITIMM
KLPRTPTSLFKFLTVLENDVWLSILAAYFFTSFLMWVFDKWSPYSYQNNREKYKEDEEKREFTLKECLWFCMTSLT
PQGGGEAPKNLSGRLLAATWWLFGFIIIASYTANLAAFLTVSRLDTPIESLDDLSKQYKIQYAPLNGSAAMTYFQR
MANIEEKFYEIWKEMSLNDSLKEVERAKLAVWDYPVSDKYSKMWQAMEEAVLPNTIEEAIQRVRDSKSSSEGFAWL
GDATDVKYHVMTSCDLQSVGDEFSRKPYAIAVQQGSPLKDQFNNAILQLLNKRKLEKLKEIWWNNNPETMKCEKQD
DQSDGISIQNIGGVFIVIFMGIGLACVTLGVEYWWYKWRKRPVVGDVTQVEPAKSTRNNVDKQGEGFTFRGRNLGL
TFKPKF
>HarmIR31a
MLAQAVVEFFKNKVVSSIIVLACWPAVDQMKFTRQLSQYGMSATFSCEPAILDKIHPQYLQGVLYMVRDDDNLAFF
KKVKRVHFTMKYKWLLLGDHVPEALRDIRYDSDVTFLKWGRNNRNETKKSTNLALLPEPPTSPIYFYDVYVHLREG
ISIHYWAHWTKESGFVVTHDREKILRRLDLQRYPLRIATPVGHYSSDKYDGTFVDYLKDETMLDQDPGIRSAHSAS
MLLTEAVNAQDVLIENELWATVVNNNSMFLMVSNGQADLSGGVLRLLLDRSFKLDYVTPLWPFRVGFTYLAERESS
SNMYLEPFSPGVWWSCLGIMAILAMVEWITAKTPKEREGALYTVLTTWLQQDASAVPEGASGRWAFTVLSISAMLV
HAYYTSAIVSALMSTGRGGPETLKALGDSKYAIASEDYDMYRYLFFDVETTWDDLEYLKKKKKTSKFYQELERGVE
LIQQGNTAFHSEYNQIYPHFKTFSDDHICKLQHIDTIPEILTWVTTTKHGQWTEVLRIAGSWLQETGLRKRLVNRL
RIPQPPCRASLLAERVKLGDIAPLLALTMLGAILSVVLLGIEIMYAKAKGRKLQEGDAKHALEDSDDSSEVTDVVY

NYLA
>HarmIR40a
MELLLLFLLINGVQSLLDIQDVTSDVMTSLPKDFSIAVKDIAEGMPTKAITIVRGNSTNIRSQDIFELLCLLSQHK
VLTTNLDIATKENKQKYYEFLGKALDVSDQRTSLILCEPYECENILFELTENNLIHSMILYFFYWSYGPVSDTFLM
TMKEAMRVAVITNPRESVFRIYYNQGTPDRLNHLTLVNWWAGTLYKSPVLPPTDKVYHNFRGRVFEVPVLHAPPWH
FVKYNNDNTITVTGGRDDKLLALMSKKLNFKYRYYDPPDRSQGSSISSNGTFKGTLGLIWKRKADFFLGDVTMTWE
RLQAVEFSFMTLADSGAFLTHAPAKLSETLAIIRPFQWEVWPLVFATLLVTGPALWIVIAAPSLWQRRECDQLGLF
SSCCWFTTTLFLRQSSSKEPSSTHKARLVSVLISLGATYVIGDMYSANLTSLIAKPSRERPIGTLQALEEAMRDYG
YELVVESHSSSLAILENGTGVYGRLANLMRRQRVQRVRNVEVGVRLVLSHKRVAVLGGRETLYYDTERFGSHNFHL
SEKLYTRYSAIALQIGCPYLETFNNVLMTLFEAGIIAKMTTDEYKNLPEQARRSDPVTESDKQGNELMGDSTTASQ
APQSESAKGLQPVSLRMLRGAFCLLGIGHLLAAISLAVEIQLHRRSKRRHEPPPSEHRKTQKLLVLGKSVMLFKRG
YKRVCATVYTGIDKALGPEVKD
>HarmIR41a
MLVSTPALAPLEILLNTIINRYLQSAYCITVFSETPFTFILPTSFISLIPNETILVEQIFNVSETGCSDYIVRMRD
PQIFMEAFERVVHIANVRRSDRKIIFLPYDEEYNEENDVNLPSLVFAMKGSKYLANMLMIVNHASVNQDCKIFNLV
THQFVGRSEEATHLPKYLDSWDSCTQKFENEANLFPHDLTNLYGKVVRVACFTYKPYALLDIDPAIEPLGRDGVEI
RIVDELCRWINCTVEIVKEDVDQWGEIYANESGGIGVIGSVVEDRADIAITALYSWYEEYRVMDFSVAGVRTAITC
IAPAPRLLSSWEMPLMPFTWYMWLAVVFTYFYASTGLLTAQGCSTTTYPFFNVFGMMIGQSQYESRASWRIRGVTG
WLLIAGLILSCAYGAGLASTFTVPRYEPSIDTVQDIVDREMEWGATHDAWIFSLTLSTEPLVKQLVSQFRIHSFDE
LKRKSYTRSMAYSIEKLPAGNFAIGEYITQEAVLDMMLMLEDFYYEQCVVMMRKSSPYTEKVSQLVGRLHQSGLLL
AWETQVALKHLNYKVQVEVRLSRSKNDVGITKPLNLDNVVGIFIVYAIGLTISFAIFLGEIYVHQRKKKNEVLHID
>HarmIR60a
MSVDKIFLVNVVIFIQVHISDGVVNPFGPTVVNDYTNCISKVIDNEFHEPGLLIFANTNNVSTSVTRIRTKLLKRL
HKEIKFSIEIMSPNNEVEICDLDNYNLGVLHVDVYVAIPFANYFVIIIDSYTDFSFLASKLIRSRSWNPFAKFIIL
LFNFVQDDKVNIDYVERVLSCLFKYNAINVIIAVPKANNFRNAIIYSWRPYDPPKYCGYFNETAKDRLVVQNMCES
GVLKHDRKVFDNKIPHDMEGCVIEVLALQRHPFISDDEYDANIEKLMIDAMLKRFKMKARYNFIDGYRGERENVGE
WNGGLKKLASKSGHLLLGGIFPDFDVHEDFETSVTYLADAYTWVVPRAHKSAAWVALVIIFKSLVWYSVIAGFFLC
GITWKIIAELSEDSDYNRSFRHCFLNTWITVLGFVSYLHPVKESLRVFFVFLNIYCMLFSTAYQTKLFEVLTNPSY
EYQIQTVEELVESGLKFGGFEELHDLFYNSTDPFDYRIGDQWTDITNITEAMIDVAVHRNFSLLCSRLELAHISGI
TPELSDSVGNYKYYTFTDNVFSVPIETIALRGFPFMMEFSTTITIFKQSGLNEGLRQHFAHFNERRRARQLRALLK
EKSDVNPLSSEHLQGGFLALALGYVSGTLALIVEVIVNCDYVQNKFANFKRRVNLLS
>HarmIR64a
MNLFCNLLLTLSVTEVPLVIDILKHKNIKNAVLFQCYNNHFVSGVHKIFNENNVLIASSKIISNGTYIIPLDRKKT
GIVVDTSCEGWNSVLDSRTVSFKDYSFIIIAEHLSPILEALSRYPILVDSDVIVAHKLNQSYNLYEVYNTGFKLKG
KYVIRLLGHWNSSLYIEDLNRWDLGGAFVKTAVVVINPTKLTNQTTEQYMEKPIRMQVKVDTVHRMKFFILLKYMR
DMYNFRFDMHRVHSWGYKRNGTFDGMVHALYCGQAELGGAPIFYRIDRWELVQYVSEVWTSRHSFIFRHPKYPGGF
YTIYTRPLSGIVWYCVIAMLVVTAVILWAMLLVQNTWGDNEDSSLSLAGIIIWGAICQQGIAINRESTSTKLLIFI
TFMYAVTLYQYYNATIVSSLLLEAPRNIRTLKDLLDSDLKAGAHDIVYNYDYFKRTTDPVAIELYHKKVVTATQHN
YFPAEKCMDLVRRGGYAIHIDTSVAFPLIKATFNEREICDTTLVQMYPQQRMGVVMRKNTQYREHVAYAIRRFSEA
GLPQRLRSDVDEPMPECAHTPDSSVFCVGIREFSTPLLVLALGMLVSVLLLICEIVLHRVVQRAGLRDFVH
>HarmIR68a
MFKIIVLFCFFTAVKLDTFPILKDLHERKDLEYVLIDLINVLMREYEVTCIAIICDEVYLNVFSGPLFKRTSAIPY
VMTVVEDYEDLLSPNFVTLESLRAARKEGCNVYVILLANGLQASRLLRFGDRHRILDTRAKFIMLHDFRLFHSELH
YIWRRIVNIIFIKHHNKMTGTAKSRPWFELSTVPFPNPIKGVFVPRRVDIWKNENFHYKRPLFADKTSNLNGEVLN
VVYLDHVPSVVVMKNNVSTKLGGVEVEILHTLAEKMNFKPRAYQAANAEIHKWGQKQPNGSFSGLLGEMVNGRADV
ALGNLQYTPYHLGLTDLSIPYTSQCWTFLTPEALTDNSWKTLILPFKLYMWIAVLLVLLITGTIFYGLAKNHMNLQ
EYRRKSKSKRSNIDDENTKPGLYLFGEIINSILYTYGMLLVVSLPRLPMGWSIRLLTGWYWLYCILLVVSYRASMT
AILANPAPRVTIDTLKELVDSKVTCGGWGTQSKKFFEQSLDEYSQRIGDKFETIDDPMEAANKVAQGVYAYYDNSD
FLKYISVVRKNSFMDPKQNNTVNNTEVTGRKDTQRNLHIMTDCVVNIPISIGFHKNSPLKPLADIYMWRVVEVGLV
EKWLNDAMYQIRTLETSEDEVKALMNLKKLYGAFIALAIGYSLSAICLIGELIHWHIIVKRDPNFDKYALHLYYLH
KNKKH
>HarmIR75d
MELISFILSYFITKDLSMMTAFICWTPERTSELCRSASGAGVRLQLATDFHQPPPMTPRGRFREAMLLDLTCPHAA
LIMEAASSSRGFNYRHSWLLLHNSSERGLIADALSPYEILPDADVVWSAPDALLDVYKIKPATPYLLTDLGLTRNC
SRHQLRALWGALPSAVTRRRDLKNVTMKGISVVTEPYNFKGWSDLRNRQIDTFPKFTYPLMMLAAQDMHFRFDLRQ
MDVYGVSHNGSFDGLVGRLQRNDAEVGLASIFIRPDRMQVADYISETCVLLCAFIFRQPARSAVSNVFLAPFSAGV
WAASAGVAAAAALLLVALRAVLQRTRQRDDLALFTLPETLTFALGTLCQQGFHTTPGVTSVRLVMFSTLLASLFVF
TAYSAKIVAILQTPSDALRTIDDLTRSPITIGVQDTTYKKVYFLESPDESTQQLYRRKILPQGERAYHSVVDGIAR
VRTGLFAFQVESSSGYDIIRQTFTEREKCSLKEIEAFKLPLVAVPMRKNSGYRELFATRLRWQREVGLMSRERRVW
LVSRPRCDAAGGGFVSIGIGDVLPALQVLGLGALISLTLLAAELAAHAIRNSRRWNHI
>HarmIR75p
MNIFTFIFLLLIHKSVYAKDANTINFIKLFIQNDQKPTHLIYGGLCWKKNIINKLVVELSTIGVRTSASFKPKSKY
QDHAILYLTDLDCAQSKAIISYALSKELFQFTYRWLVLVTSKQLQQSKISLLMNGSVLVDSDLVLAERAGNHFKMV
EMHRPGLNGSMITTPRGFYNGSFVDVRPHRELYRRRRNMMGHPITMSNVIQDSNTTREHLPKEDRLELQYDSITKA
CWSAAKIGFEMINATAKYIFSYRYGYKVDGQWSGMIADLYSNKADVGTNCVIFRDRFDVVTYTDLVAPMRMLFIFR
QPPLAYVANVFYLPFSTRVWVTIAVCTAIATVTLYLASKVELVLTKASTQQQLDGGICDALLLTMSAVTQQGCYLE
PRRAPGRMMVFVLFTALMALYAAYSANIVVLLQAPSDSIRSLPQLANAKITLAANDVDYNHFVFNQSKEPLHTSIR
DRVFPENGKARLYSLADGVERIRKGLFALHSVAEPVYRQIEATFLESEKCDIATVDYLVTFDSFTPVRKGSPYLEL

IRVVHKQIRESGIQSAIRKRFLVSKPHCTTKMSSFSSVGLMDMKPVLILMLYGVCLSVTIAAAEILVFKLSEHRKK
YKVESTEPPSP
>HarmIR75p.1
MNLKILYLLSFLYVFMVKCDGNFNSDVIMSFVTLEERPTSLLIPHLCWSQHALTSLVRSMTSVAVTTATSLQYNRT
EYHLQYVLIMADLACPGTDRFLIKASKEGYFKAPYRWLLLNYDDDKTVLKDVHMLVDSDVVVTKRISDQEYWFIEA
YKISENSEVIYTLRSKWGRMEDHRSSNVLSSRRHDLRGHILTMSNVITDSNETRVHMNDRLNLHQDSITKMSYAVV
KICFEMLNATERLMFTHTWGYKDKNGQWQGIVDQLLKKEADLGTLTIFTQERMMAVDYIAMVGSTAVRFVFREPPL
SYISNIFALPFSGAVWLAIFICVLGCSIFLYIASKWEASMGMHPLQLDGSWADVLILMIGAVLQQGCTLEPRYAAG
RCVTLILFLALTILYAAYSANIVVLLRAPSSSVRSLPDLLNSPLKLGASDFEYNRYFFKKLNDPIRKSIYEKKIAP
KGKKANYYSMKEGVERIRKGLFAFHMELNPGYRLIQETYQEDEKCDLVEIDYINEIDPWVPGQKRSPFKDLFKINF
LKIRESGVQANIHQRLTVPRPRCSGHVSTFSSVGITDMYPAMLMTLYGMLLAPAVLLMEIMYHRLMIARQQKRGTS
DYDHIPFRH
>HarmIR75p.2
MLSMRIILQAFILVLILVERKAFARDHTLVHFIKSYVQNEEKPTILIMNNLCWEKKVVVSLANEMSKIGSRSSTSM
GVDSRYYYHDLLYLLDLDCPGAEDIIALATARNLFRSPYRWLVITAWSKNANIAALWNSPLLADSDLVLAAGSGGV
LKLVELHKPSPNGTMISTLRGFYNGSLYDVRPHRELFRRRRDVMGHTITMSNVIQDSNTTVYHLPREDGMEPQYDS
ISKICWMNVKLAFQMLNATPGYVFSYRWGYKVNGKWSGMIDDIHSGRAELGTNCVVSDIERLDVVAYTDRLAPFRV
RFVFRQPPLPYVANIFSMPFSKNVWIAMSVCAVLSTATVYLAAKWEAKEGKGPTQLDSIGDAMLLTFSAIGQQGCV
MEPRRLSGRMMVFVLFTALMALYAAYSANIVVLLQAPSDSIRSLPQLANAKITLAANDVDYNHFVFKLHKDPVREI
VYKRIDPEKGKKHFYDLNEGVERIRQGLFAFHSIVEPVYMRIEQTFLETEKCDLMEVDFLNSYDTFVPVRKESPYL
ELLRVVFKQIRESGIQSALSKRLQVPKPHCTSKMSSFSSVGLMDMKPVLILMLYGVCLSVTIAAAEILVFKLSEHR
KKYKVDSTEPPSP
>HarmIR75q.1
MKYFTLFLNILCLKWCVSILPNNDLQMIVDLANSYEKPTAIVANVCWNSKADEVKLSKMLTNLERPITVRYLRKNK
TFANSYPNNHLLLLLDKNCVDAEFFLKQANANKMFSKSYRWLVLGNPIQKESIVPPEFRGLNISVDSEVIMAQKTA
NNDTLLHTIYKIRPKSEWNIEYYGVWSADYGLNKSDKTIQSNVMRRKDFKGEPLTASSVVEDKRTTLSDLITLRHI
LVDTVAKSTFRSINPLFDFLNASRRILFNDTWGYKVNGTWNGMIGEINTGKAELCGMVTFMSLERLKILEYFTNPT
PVTVKIVFRKPPLSYQNNLFLLPFTTGVWICLGAFVLIMIVVLYINTKWDIKKYEQFNEQNMDQTCLPPTWSDITI
FVLSAISQQGSSNELKGTLGRLVMFIVFLAFLFLYTSYSANIVALLQSTSNQIRTLSDLLNSKLELGVEDVPYNRY
YFSPAQSASDPIKKAIYETKVAPRGKPNFLTLEEGVKAMQKRPFAFNMNTGTGYRIVSALFQEHEKCGLHEIEYYQ
NAKAWLCSGKNSPFGEMFKVGYIRIQEHGLTDRENRLAYAKKPVCSVMGSSFDSVNMVDFYPVCLMLLYGMILAFV
LLVIEILAHRHQMKKHNQEPDVTQLQ
>HarmIR75q.2
MKTIFFIAALCSLSLCNGQDSQNMQVMLSDVIEASGRPSSVIAKLCWTPSKIIQLHTHLTNKYIQFSANDVIKTDN
PEFYDEEQHIVFMADLDCPDIDTYFEKNSARNVFRAPFRWILFGNSSTNDDDIVPRAISNIDVLLDSEVLVLRSVD
DVYEMHFIYKISPNNTWQTEFYGTWNAKHRFQKSPRFFEPTSLRRLDIDGYEISICYVLTNNESVNHLSDGLEDHI
DTITKVNFPTTNHLLDFLNAKRKYIFANTWGYRVNGSWNGMTGYLVRGEVEVGGSPMFFTFERVSIVDYIASPTPT
RSKFVFQQPKLSYENNLFLLPFNTTVWYSTIALVFIIYLVLLLVTKWEWKKTQDLLETREKDAGVLRANVVDIIVL
IFGAACQQGSPSELKGSLGRVVMLVLFLALMFLYTSYSANIVALLQSSSSHIKTLEDLLHSRIKFGVHDTVFNRYY
FSTATEPVRKAIYEKKVAPPGTTPRFMTMDEGVIQMRKGLFAFHMETGVGYKFVGKYFNEGEKCGLREIQYLQVID
PWLAVRKNTPFREMFKIGTKRIQEHGLQYRENRLMYEKRPKCTGGGSNFVSVSMVDCYPAVLILSYGAIVALFLLA
LEILAHKKENILRKLNCRKDEMN
>HarmIR76b
MAGIELIISSICNATFCDVPYGGADKGSEVLTPEVINFKSLMQDVNGKNLKVTTYNNTPLSWTEHHNGTVVGKGVA
FTVMEILRKKFNFTYDVIEPKRNYELGGRVTDDSIIGLLNSSKVDMAAAFMPTLIAYRKQVSFSIDLDEGVWVMML
KRPKESAAGSGLLAPFNDLVWYLVLAAVLTFGPCITFFTRVRTKLIADGEGVLPLKPSFWFVYSAFLKQGTNLSPE
ANTTRVLFVTWWLFMILLSAFYTANLTAFLTLSKFTLAIENPRDLYQKNFRWVASAGSSVEHIVKTEGEDLYYLSA
MINNGKARFLSVLSDKDFLDPVKRGAVLVKEQTVVDHLMYNDYTSKKDVEESDKCTYVVAPNAFMKKQRAFAYPVG
SKLKSLFDPVLTQIFQAGILDFLKRSDLPSTKICPLDLQSKDRKLRNSDLIMTYLVMVAGSATAVAVFAAEIFIKR
YLSVKVNKTKKTKDRNKKSKIGKKSTRYDDSRPPPYDSLFGKNPRFNVETTRTKIINGREYYVFETGNGDRKLIPA
RAPSSFLYRSDK
>HarmIR87a
MFAPLPTLLFFIHFSSTMSENPLLMTTGNSGQTTKTAECVLKLSAKYFVEKKALSGSIVIININSYTSTTQGLLLQ
TVHSGIKYSIMVKDSFYPHANASHFPEKAKNYMLILEEKSELERNILQLNKLPTWNPLAKAIVFYQLKHNETAEET
SIEFINELRDYKLFKTIVFIYDEYNDVVISYTWRPYSDTNCGGRCDSVYVLDRCTNNTIYEFEKQHDMFPSDMKGC
PLVAYAVIAEPYVMPPVGKITNSSFDDAYEFAKGGEINLVKIISQFTNMSLITRTSDILENWGVVYQNGTATGAFE
VLRNESADLVIGNVEVTRILRKWFHPTVNYLQDEMTFCLPKAGQAPTWDNLVIIFQWTTWVATFFSLVIMGLVFHV
FYYREHTNATKWPTNSLLMTFSMLLGWGASFEPKSPTFRILIFAWLCFSINMGISYESFLRSFLMHPRFEKQISTE
ADLIQSRIPLGGREIYRSYFETNNASSFYLYREYNSTTFSEGVRRAALERNFAVVSSRRQAVYQDQKLGKGAPLIY
CFPESNNMYKYGVAILTRRWFPMLERFNNIIRSVSENGLIDKWMNELLIHSVSSEEASTIMPLSIQNLLGAFMFIG
FMYGASIVIFIGEVVIGFIEKRKQTKKVMNKRLR
>HarmIR93a
MKVWILSFVCLFVSVSGEDFPSLITANASIAVVLDRQYLGEKYQSILDELKDYIKELARVDLKHGGVIVYYYSWTA
ISLKKGFLAVFSIASCEDTWDLFSRTEEEELLLFALTEVDCPRLPSHSAITATFTEPGEELPQLLLDLRTSNAFQW
KSAIILHDDTLSRDMVSRVVKSLTSQIDEESASPVSVTVFKMKHEINEYLRRKEMHRVLSRLPVKYIGENFIAVVT
SDVMTTMAETARDLLMSHTQAQWLYVISDTNAQNGNLSSFINDLYEGENVAYIYNMTDNNPDCKNGIMCFCQELMD
AFISALDAATQDEFDVAAQVSDEEWEAIRPSKIQRRNMLLKHMQQHLAAKSRCGNCSTWRALAADTWGATYRGLSD
ASDLSNVNTNGSSGVIDKIDLLKVGFWRPIDAVRFDDVLFPHIHHGFRGKELPIITFHNPPWTILERNESGAIVKY

SGLIFDIVNQLAINKNFTLKIILASVLKKELANDTLADTMHGMDAKLTIAAISKGQGALAAASFTVLADPMPGINY
TMPVSIQPYAFMIARPRELSRALLFLLPFTTDTWLCLGLAVILMGPTLYIIHRMSPYYEAMEITRQGGLATIHNCL
WYIYGALLQQGGMYLPRADSGRLVVGTWWLVVLVVVTTYSGNLVAFLTFPKQEVPVTTVAELLENRALYTWSITKG
SYMEMELKNSDEPKYIALLKGAEMVTTSVGMGGTMTSGSALLQRVRFHRHVIIDWKLRLSYLMRADRLESDNCDFA
LSAEEFFDEKVAMIVPAGSPYLPVINKELDRMHKAGLITRWLEAYLPKKDRCWKASSMMQEVNNHTVNLSDMQGSF
FVLFMGFFSASTVLVLEFLYHRRKRRSELTVIKPYVE
>HarmIR100b.1
MKLLAFLILTTLLAISSSKPGKKLVPSVAIDCPLIPITKHSQTILFDVDVNSDPSIRTCILKSVSEQSVLILTDYD
RRPEVNFTNKRIMTNEFPNVIITTNHLSFKAVRCNKSPLLELFMKSNWSWLYIIVTGDSVFSCKNGTMTSKHFRLL
EKFMNTIWHRFEVMRVGLAFPIACKQKMIIYHRKRPSTQKLYDRSIKLINATSYKDLLAAINYSGTGLCAHYPIKA
NIFERYPTSITQCKNLHYYDIHFNLNLTFGYCGLDGMVMHDLLTHFRFNLSSPKNEDCNIYGFAIPGNISGSLGCI
VRNELDLSFNSRFMTLYSDEHIYYLHYITTDKLCALVRKTGVVPLWHGPYNVFRPPLWFIIMGVLVLISGIMWVYA
IINRTITGQKVMAYWYYLLNAVMTTMIGCSPMKNRSMIIIRSACLSGSILFLAVYQGHTSRVYTTLKHFERISTLD
DLYAFGAILYTTPGMRQFTRQLQRPGNKLEEDFFNRSRLILNERIGAGITLEIPRATTLDRKSDAEMKILEHFSDR
EGRPLIDIVDECFMNYFLSYITRSGFPFFEEIQIFTQRLLEAGLPTKYYKWTQQMLNIPTSLPETRSEPRPFSKIK
LKDQRVAFFVLFVGSALSIIVFAVEIFKGPPVEF
>HarmIR100b
MHRIKYFVFVVCIASSNCELRTKLMPAVLPNCPLLPVEKYSQTVVIDINTNTNPELKSCLVKTCGHSVVIINVCGK
RYTINMDIDRKSIKTTDYPNIILITKHFTKKTADSGLLKYVVKSLNWSLLYFIVTGQQQYQCLNGTMSTSDMLLLD
NIMNSIWHKFKIMRVIVAFPYTCEDKMLVYHGKRPSTDGDCLYDRPVKLINATNKEELLKAIRKSGEKLSENYPIK
ASIFERYPTSIKDCSNLHYYGHFNLSRSHGYCGLDGIVMNDLITHFNFNLSFPENETCNTYGFAVPGNLSGSLGCI
ARNELDISFNSRFMTLYSDEHIYYLHYVITDKLCAFVKRTGVIPIWHGAFNVYSPPSWMFIIGVIMIISVIIWGSA
IVNKKLTGVKNKSCWYYLYHTLTMTMTGSSPMKQRTLLLMRGSCLAGSVLFLAVYQVSWSGHISRVYTTLRRIEQI
STLEELYRSGATLYTSPSFRELSKQLLNKKNKLQVEFFNRSLLTPHETLDIVLQQPFATSLERKSDAEMEILTKYS
NEQGPLIDMVAECLHNYYLSYIARSGFPFFEDLQVFAARLKEAGLPEAYYRWTQKMLNIPTSMPEDHSIPRCFRPI
RLRDQRVPFGVLFVGTILSLIVFVAEIRKGKSRNVK
>HarmIR85a
MLVIFVILLMCHPSLCFDESSVLFQEVKEYHRDAWLKAEYAYEVVNIMYNSFRQWYFTVTFCEFTYFENRILKYTE
QYGYGYNVMLLSGCPYSNNSFVKPRHNRHGETAYLVTSNDLSLDVSETVIAALKRTGIFKPRSAVIFVIKNVLELD
NYFYHALSNHFQMLWSSSVTNSVLILKTDRLRMYSYNPFFQEIKDITNVRDVSKLLSKQYNNLYGYGLRLSVFRKV
YVSDRTGPVRCDSYLAQTVMKFLNASCYPLPPRDGSTVGDLLENGTATGVTSDLIDGYTDLELNSRILKNTYYGYI
DTTYPLDQDELCFLVKKSDTQSTFKTTINLISMEMLLLFFFTFTVFIIIITILVRKAENNLLNLNDERRAEDTLIDL
IKCFIRQTMDFDFMGPVFRSLVLLIIIYSLIIDCAIDGIITSAITYPRYKPDIETMAQLGASNLTFGIHNRDLKIF
NSSLSTDYYELIKNRIVPFSDKKIKEVLEKREYQYATLLRKSDSQYVSRKVSNMRKGKPLFYTVPDCPLPCFIVYG
LRYGSPYLNRLNYIIHHLFQAGILQYWSKTEELNADRSRLGAIENKDRKPLNIKNLQEMFYMLAIGELISTLVFIF
EILYHKYHKKQ
>HarmIR100f
MTVVLFLTLLLLANGTSKSLMKPENPDDQLLTTCVIDILNKYFIEQKQLTIVLNSTADMKLQDIYSKTNTTIFIRR
PFCNFCHYKRVHASYVFFANNSQDFSENFQYLVQEPFWNPYGKFLIVISSLQGDLRVIFDVLLMFHVNNVVLLNGT
GPGHLYTYNPFANYACGRYYDEIIGFGTCLNNRANLFPDKLVSGLRNCTFRVSFPHKPPFTVNPSIVKHLIRQQPL
IGFEEKLLKVLAEQEHFNITYNDRYGKPFYSVILPNMTAVGPLSLIQRNKTDIIIGGMMLSPARYSALSHVSGHFD
YLDELIFAVRKADLVTNSKKIFLEFHPTVWLLLILVFIIYFILLIIVLRPKDKSLIMLKMLSSLVLHGCGMSCRYT
VKWLVLIWLVFAVIINIFYQTSLYSLTNNPIREHQINEEHELNNLKACIEPALVAYLRAEHIPNNITLSEEDINDC
DNIARNVHKVGQNRKLYTVLRKMMFEFSKDHFTDPWGHSPIHTFTKPLSKTISAILLYKGFPTTHQLKVTLLRLIE
SGLVNKFLGEHMHLKKIKFVFPREPFRAHVIIPWNLFAVGCILALITFFLETLHHRKYKIYFRGDY
>HarmIR100e
MLTVHFLLTLSLASCRSDNIHIIEPQTKMPLIMCITHVIEKYFSEQKVLTYVDMDSDDNVLLKAIHSLKATAVVFR
QPFIRSPFRHRGYLIAAKTSNIFADHFKKLRQEPTWNPAARFLIVIKILKEDELKKIFDVLLESHVFNSLLINATD
EAQIYTYNPFDKYACGHYYSEVIYYGECSQTKTDLYPNKLVTGFKNCSFRASVPHRPPYSVDRAQLNNITQTDILG
TEQLVLKLLGEKEQFQVNYTYDYDKFIYSTISPNMTVSGPMKTLTNNESDIIFGGIWLVASRSDAFTYLYGHLDFE
DDLLIIVKRASLVAIWKTTFLEFQPTVWGLLVMTFLLYSVMMIRILGADDKFGVMMTLFKNLLANSVHIPNRSEVK
RIFLTWIWFSFLINSIYQSSLFSLTSHPAKEHQVYKIREIFVYKMKPCINPSMLIYIETEMNVSTPIDNNCLDTPK
NLYRVCQSDKIFTLAQKFVYQYYKKLYCDRFGQPRVYYFNAPYAQLMFGIFFYKGFPITDRMQTHLLRFKENGLVS
KCLKDHYYSRKIKHHFHEKEFEARFILPWVMHLIGCVLATIVFIFELLSKRFKWFQR
>HarmIR100c
MTKRIVVLFYLTFHGSFIQLTDAENIPELYNFAVNKKYNTVGCINSILTKCFPCGSLTTFVNPNANMDQLIKTTNA
ENICHSIIVRSFDDCDWYIWTNVYVITAPDLLAFWFGMLDLSRDLFWNPRAKFIIQVDYLGANGEGIEDVFKIVIK
HRMYNVVLLQNLKDDAVIYTYHPFENNSCGKRFDKIITLGKCENEDDIVDYFPNKIPSEMKNCTFNVVATDDVPNF
ISKSSNYTVYGKYVSGLEQFVLDTIAEREGSFVEYEVITGDATFGVVLPNRTTTGLLNYIDRNKADIAAGGFILMQ
NRVELFDYIWGFNYAAFYLYTPAAGNQVWRRVYREFGLRTWTLTGAALVLMIVVGLVIKRLINDKSFSVLYIWGYF
FGNSNGGFSTHKKFKLLMLMWSFFAFCISSFYNTALVGLVSVHVQEQPHSLNVGNLKTLSYEPCISDNSRLFFQYA
YNQTLPVGKDIHNCTNTDSSLELVASTKKYYAVEMEYSYKLKEYQYMDKQGKPKLESWLFSNTNVIVVYLVRGFPF
IEKFQDYAHRLYESGLTLNHFKLISLRSYSVLQRHPKPFSVTKLLDLKMHFGILVAGWVLSFICFLLEIWYNNINI
SKKIQRVYVN
>HarmIR100d
MFFLVMFYSIVSAGAFLTTDHENIESLTNCVVKIIKKVDYNRYTKVVDVTLMNIKNDIKLSALHNLTGVRFVSRRF
FWNTNDLSNKYYLVMSEDFQELEEGLKEVTSDIFWNPLAGFIIVLKSHKHHSSHDITDLLHTYNIFFASLILHKDD
KYFIYKYNFTASNRCYKAGHLTLWASCSDFCSEKQLPILIEGNIRNCQYKFISRNLWPFTNFDTAFKGTEQWFVAL

FEKQYGVKIDLKKFGKIDKCETITYDTKYIMLKKVENNEVEGAVGGYSITEEYSGNVSHSYPISIDYMYYILPHKK
YVGPLVAVLHGSTGTFVLIGLLFVIFCIAAKFLSIFAAQKDFSQDVLMVFGYLLNKCSANRISAGWPQAIVFSSLL
FTSFIFPYAIQANLYSVTTEPVRGQEPKTSQDLKNYKAVLYTDFQYRHNKEFSGYLDCGNRINCLMLVKNCPDKSC
YTVMTSAHYFANLWQLTDDECKMTTYILREPYITSLRTIYLRRGSVLIRPIKEFLLRIINSGVLSKYSRDLYIREW
LKRKCHHRSEHVPLSMSSTYYVFMMLIAGYCLSVITFICEFMARSQIIIRRRT
>HarmIR100h
MLVAYILFFLLYEANSYKIPTPCPEYNKKLVKCVTDIVREHYSEKKVITYVGDKFEDEELLKAINNAGTVSIVSLK
STKRMIIPHEAYLLSGKNATFIAKHFPKVRRETSWNPLARFLVLVKNLTESDLKIVFDMFLQLHARHVIIVNATED
AHLYGYNPFDNYGCGKRYDYIFSYGKCAKAFYKELYVNKIITKLRNCTFNVVITQWPPYTILPTNDSNDLSPLRHG
AEPYLFQLIGRMLNFKINITYDYTAVEEFPTVSTDMEAVGSLKKLQDNQADAAIGGMLLTPSRALAFSFVYGHLAY
TDEIRFIVKRASDVPPWKNVYLEFHWTVWVILLLTLMFYSILVILLLQTNDKSYVVLVLLSNLLLHGHSFRSRSTV
KTVLILWMWFAYLVNTFYQSTLFSLTTNPAQEYQISNEEDLARFRLKPCFSKVMENYYRESVQSNDGYQRIKGCDG
LVESVHTVANSEDLYTILLNGQFRYNMQEFRDKYGTSRVVALPKPYSKVMYAIFLYKGFPMINYFLHKSLRLRELG
LVDKVLKKLTYRRLIKYRFHEKEFQTRFAIPWIIYVFGCSIAIITFIIEINMPRH
>HarmIR100i
MLVAWVFTILLNFGQCQNILPPEAEDNQELRQCVTDILDKYFNESIELTYVNMETDNEDLLQTVYKSQKFSLVTRN
SMYQSVLPNYGYLIITRNVNTFIEYFKYLQTDTTWNPYARFLIIIETLEDEDIKSVFDILLRKHVNNVVVMNGTTD
AHLFTYNPFDNYACGKYYTDIIDYGPCKENPNDLYPNKLVTGLKNCTFRAAIVHRPPFTVNPLKAPKTILGTEEYI
LKALAELEHFEVTFNYSVNPAVFSSAFPNMTAFGPMEMLQNNETDVIFGGNMMVLTRGQAFSFLNGYHDYNDELRF
VVKRASLVPLWKTVYIEFDTTVWMLILLALVVYSVMVIYLLQTKDKGFVFMELLDNLLTHSRDIRCSMTIKCILMI
WVMFAYLINTCYQSSLFSLTTNPSKEHQVASEEDIIQYKYKPCFSVALKTFLSLEITEGMLSTRSSPADIDQNACS
TTIQAITTVSRTKGVFSLIPNYIYLYNKPSFNDKFGNPLIYYFDKPFAKFLYCFYFYKGFPISNRMRMNAIRMREN
GLADKSMKDHFFKRALKQRFSQQEFETRFVLPWGLYIIGCTISIITFLVEYMSQSQLKIQEQHVL
>HarmIR100g
MSAAILFSILCLNYCTSANGFYLGEELRNENLIVCVTGIIEKYFSEYKGLTHVQFETNSEHVRLMRAIHSSAITAL
VTQDPNQQLRVSHQGYLITASTAKEFTDNFSTFVDDLSWNPFARFLIVVRRVHEEELYQIFDVLLRWHVNNIILVN
GTNDAQLYSYNPFDNYGCGKIYKDVIRHGFCLETTSNLYPNKVVTGLRNCTLRAAIPHRPPFTLRPYMIEQNDRMK
MGSEQYLFCLLAETEQFKVEFYYEGDYYPLSKLYDNISIPGPMENLRNNKYDVMFGSVMLVASREARFTYLHGHLD
YHDEIRFIVKKANDTANWKNAYFEFHARVWWTVLAVFIVHSTLLNIHLRSKDKTDTMLKMFDLLLSHGCKMPHRMS
ARCLFLIWVWFAFLINNLYQSALVSLVRTPVKEYQVKDEEDIVRMQLKPCISPPLLQYMYTESNITNSFNSNCTNP
FSSINFVAKSKHYYSVVQKTVYFYNKKQFCDEEGENTVYFFKKPYAKLIFGIFFHKGFPISDRMRTNAIRLRENGF
VKKKLKDHIFAREIKIKYHDKGFKCRITVPWFIYVGGGSLAVIVLIVEIIWSRYKE
>HarmIR100j
MRAVYLLVFLIYSGNCVKIPIAGPKFSEKLIQCVIGIIREYFTGSKVITYVGNSYQNEELLKALNNANIMSVITRR
STIKRTSKHQAYLITASNATFFAQRFIKTTKEPSWNPNARFLIIVTDLDGDLKLMFNTFLKLHANNVIVVNATDDA
HLYSYNPFDNYNCGKRYDEIIEYGKCSQAHAYDLYPKKLVTGLKNCTFNILITQWPPYTIINTNDSNNDDPLKSGA
EPYLFQLIGEKLGFDINILTNTNDSEEFPTVSSEMTAVGSLKRVQDNEADVVLGGMLLTPSRALAFSYLYGHFVYT
DEIRFVVKRASDVANWKYIYLEFESTVWLLLLLALVIYSMLAMILLRTNDKSYVALILLGNLVLHGRSLRTRWSVK
YVLIIWVVFAYLVNTFYQSSLVSLVTHPVRDYQISTEEDITKYQLKPCFSSIMGKYYVESVQSGTGFDVTHGCYGL
MESVTTVSQSNNMFTILLYGVFQYHEQKFFDEYGNPLIISLPKPYSKVIYSMYMYKGFPMMDQLRHRALQLRENGL
VEKVMRDMIFMKRIKHSYHKREYVPRFAIPWLLYFIGCLASIIAFVIEIISKRNIHTEA
>HarmIR100a
MNYHSIFILCIFARTAHGNSTNGLSLLKTGPDLEDQRITMIIDIISLNLKQNGWDTIMCVGKLPDSFYVKIQKIPY
SFVVMNLDDPDDYFIEDTVPYHANFIIISCQDYEELEELRQKLVASPYFHPLANILTYYHRREDKATMAKLFFSAW
YYKAINSILVQFSDEEETLLVSDFTPYVNEDYKIQPENKFGCWTARNLGMPVVGFDTGYVCVEKCHNVSIHTRLRA
NNLGTCIGFNTNSVSYNDFTHLRNLKLFEDRTKDLHGFVFRAYAVQVKPFFLIKNHGNGTYTLYARDGMIWNTMAE
LFNFGIDLSPSVDVMMKPFNFEISIDQIFAFARRKGDLCLFPIYQFDVIVVGLDFTYPFKDSGICILSARAGFETS
LFRIKTLRANISTIILFLACFACTWATFTVYKAAEKRHLSFDQIGKDFMNTCRQILMINLYKPPTHHFFRIFLAIA
LWCFFVINFTSQATIISFFTAVKRGKEVDTFDDVVAKGYPLEAMASPDLILPDTEEKFRIINSRLVNEVDIYGCVD
RLKTDPRRFCLMDCSVGRYIKRNRLNHRGEQYLHIAEQDRIHSHYLAMVFSEHSPMTERFSRYMMILCEAGLIRKW
EQYRYTDIKDEVTTKPLAFDDLSGIFQVFCFMVGNTLMIFFLELVASRIKTCCQNIKIPNRLPKRNTE
>HarmIR143
MWKYLVYSTLIIVNSVHGKTDTMKLLPKNNEKNASEFNSITDCIISISDKELFFMPTVAILKQAENNIKENLFINE
LIDKLTLFNIPQIIVEEIDNEENHELYINSLTIAFIETCDDINKIDFNKVDRAMRFLITISDSTKDKCIENLQEIG
ESISHDAVTFIFRSNEADINEMFTIFPKIDGNTCKEVVDEPKHINTCINGTLDNDDIFPIKNPGNLNKCPFKVGMS
TLFPFSTMKNKEKLKLYDRVDDIKGSDFEIMKIINEYFNATLEIYYIFKTEENPYSDVEFIPFVINGSLDACAGGI
YRIYGDIVEYSGIYVSQGIFWVYYVEREDRSWQNLMHKLNDIYIFMIFYISYSIIWCLIRLFDGEAVSLMKTLLYC
WGALVGASSLQDPRSRKQKFLTLMYLIMCIYLSAYVSMQFYAFLTITAPPHTFKTNSDVMESGRTAYLKDITKYFI
SDERYTRFANKSADCVSFLDCSEKTLLYNGLTVILQGFFYNFQAATAVNDEARILRATENILTVYNEMIIRKDSPL
VEKFQKVMQRLFEAGITRRLFTEAIGISVVAKAKSANTNMISSSYSCQAGCSITLKQFAGVFYAWIFGCIISSVVF
ILEIFLKREKRLLTKIE
>DpunIR25a
MLSAKKTPHVVLDFTMAGVGSETIKSFTAALGLPTISGSFGQAGDLRQWRSLNLNQTKFLLQVMPPADILPESIRA
IVVKQDITNAAIIFDEFFVMDHKYKSLLQNVPTRHVITPVKSFQKDEIKNQLRGLRELDIVNFFIIGSLRTIKNVL
EAADENQFFGSKTAWFALTLDKGDISCGCKEATIIYMKPTPDAKSRDRLGKIKTTYSMNGEPEITSAFYFDLSLRT
FLAVKSLLDSGKWPNDMKYITCDDYDGKNTPNRTLDLKAAFQEVKEIATYAPFLIPEDDPMNGRSYMEFTTDVAAV
TVKDGASIGSRSLGTWKAGLNSPLSLSDPDSMIEYSAKLIYRIVTVEQHPFIIKDDTAPKGFKGFCIDLIDEIKEI
VKFDYEITVAPDGNFGSMDDNGNWNGIIKELIEKRADIGLSSLSVMAERENVVDFTVPYYDLVGTTILMKLPRTPT

SLFKFLTVLENDVWLSILAAYFFTSFLMWVFDKWSPYSYQNNREKYKDDEEKREFTLKECLWFCMTSLTPQGGGEA
PKNLSGRLLAATWWLFGFIIIASYTANLAAFLTVSRLDTPIESLDDLSKQYKIQYAPLNGSSAMTYFKRMANIESK
FYDIWKDMSLNESLSDVERAKLAVWDYPVSEKYNKMWEAMREAGLPNSIEEAIQRVRDSKTSSEGFAWLGDATDVR
YHVLTSCDLQMVGDEFSRKPYAIAVQQGSPLKDQFNNALLQLLNKRILEKIKENWWINNPNAMKCDKQDDQSDGIS
IQNIGGVFIVIFMGIGLACITLGVEYWWYKWRKRPTVGDVTQVEPVKTTRLDPDGRVTKDEGFNFRSKNLGLSTLK
SKF
>DpunIR8a
MDMNLYIIFLIVNLGCVVSELSLKFVFIIEVHEKDLTHKIGKALKATEENNSELKLKDSIILLDRENEEDAYGKLC
SSVFKGVSMMVDLSWSSWHYAEEISSATGVPLIRMELGSQQLVKAIDDYLESRNATDAAIIVESEADVDKTLYKLL
GNSNIRIWVNPGLSRDSSKMLKSMRPEPSFHVLVGESGFVMETYKRAVKEKLVRRSYRWNLVLTDYRIIDTTQLVL
PTVILQADPSECCRIMGVKECTCPGDLQRTQELLNSLLQYITEVYLKLSSDLTFTIKINCDNLQSSDNMNNTKEVI
YKQFAEDCESSNETLFFWEEDKQTLYLRSRFILSIYKPEQVLEKIASWNADEDYSLLPGVTLEPLRMFFRIGTSAA
VPWTMHKLDPNTGEPMITEDGQPVYEGYCIDLIDKIAEALEFDYEILTPKSGGFGQKLPNGSWDGVVGDLMHGETD
MAVAALTMTAEREEVIDFVAPYFEQTGILIVLRK
>DpunIR40a
MKRMFVIYLRVNIVFCFFDIRDITSDSLKSLPKDFPIAVKDIAEGLPTKALTIVRGNSTSIRTQDIYELLCLLSEH
RIQVINLDIATKDNKDKYYTFLKTALDVSDERTSIILCNPHECENVLEELTETNLIHRTILYIFYWPYGDVSKKFL
NTMREAMRVAVITNPRESVFRIYYNQATPDRLHHLALVNWWSGRLYKSPVLPPSNKIYRNFNGRVLNVPVLHAPPW
HFVSYNNDSSVNVKGGRDDKLLALLANKLNFRYRYYDPPDRSQGSSISGNGTFKGTLGLIWKRKADIFLGDVTMTW
ERLQAVEFSFLTLADSGAFLTHAPAKLSETLAIIRPFRWEVWPLVCATLLITGPALWIVIAAPSLWQKKRTNQRKL
LTNCCWFTTTLFFLRQSSSKEPSSTHKARLVSVLISLGATYVIGDMYSANLTSLLARPAREQPIGTLQALEEAMRY
HGYELVVESHSSSLTILENGTGVYGRLAKLMKRQRVQRVRNVEQGVRLVLSRRRVAVLGGRETLYYDTERFGSHNF
HLSEKLYTRYSAIALQIGCPYLETFNDVVMTLFEAGILAKMTTDEYKNLPEQSRRSEPVTETERPSADFTGESLQS
SQVQNESTIGLEPVSLRMLRGAFCLLGIGYSLAGLTFIIEIQTHRRSELSKSSPAGTKQMRSVTLVTRTKRFISEH
NRRIKMTICTYLDRVLGPTPPIRKTSLFKFMTVLRTEVWLSIVAALILTGFMIWLLEKYSPYSARNSPESYPYPCR
DFTLKESFWFVLTSFTPQGGGEAPKALSGRTLVAAYWLFVVLMLATFTANLAAFLTVERMQTPVSSLEQLARQSRI
NYTVVEGSSIHQFFINMKFAEDTLYRVWKEITLNATSDQAQYRVWDYPIREQYGHILLAINASGPVLDAAAGFQQV
NEHTDADFAFIHDSAEIKYEVTRNCNLTEVGEVFAEQPYAIAVQQGSRLQEDLARALLQLQKERFLEQLASKYWNE
TARQGCPDADESEGITLESLGGVFIATLFGLGLAMITLAWEVFYYKRKERNKVQAIDSTIEKSGFEGPKKLNLEKR
KFDIKEKLSKVSKLRKRDVGKRNITFGDSFKPVAKKPGVSYISVFPKNEYRP
>DpunIR75q.1
MNMSVFILILNVIFSNATNNELTMISDVIKSFIKPTVVTAKVCWTLSNKIKLLSYVMYADFPTTIRFVEDNFDMSD
RSPAQHRLFIMDLNCNNSRVILKQASAADKFVKPHRWFLFGITSINRENVLTTLDDLNTFIDSEVIVSEKTDTNKY
MLKLIYKIKRNSEWIEEHFGSWTEDNKLVKSWNILYNSITMRRKNIQQEPIVISVVVTHNRTKSKLLDLTDMTTDS
ASKSTYRHILPLYDFMNASKGLIYNPEWGVFKNNSWTGMIGQLVRGEAEIGGTVTFVTQSRIGVIEYITCPSTSTV
KFVFREPSLSYQNNLFILPFNPMVWYCMFSFVMLLIFILYINARWEVNKTTKYYGKALDSTTLRPNVSDITVLIVS
AMSQQGSCFELKGTLGRLVMFILFLTFLFLYTAYSASIVALLQSSSNQIRTLSDLLHSRLELGLENTVYNEYYFRT
ATEPVRKAIYDTKIVPKGQKAFMSVEDGVKKMQNEPFAFNMYLGIGYRMVDKYFYEHEKCGLHEIAYIQESNPYIA
CRKNTPFMEIYKVGLFRIREHGIGRREESLLISKKPICTARGGSFRSVNMIDCYPILLMLLYGMLISVSILALEKM
MYYRRRLGVTTNPDAVAELDS
>DpunIR7d.3
MNLTYGFITETFVDILTIINILQYKNINNVNIIHCDKSFDIITAHKMFAQNKMRTGFLNIGDDVTRNINKHNPTIG
IVLNTACESWKDTFDNIDTTNSFRYPFVWLIITNDVSATVNVLSQYPIQVDADVTIMYKSNDTKETFELYETFNTG
FNSNGTFQVQYKGPWNSTKNKTNIFLTRMDLTGVLIKVTVVVTRNIINETIEESLVNGAKDGMDSLHKLKFFMILK
YIQDMYNFTMELRSTHSWGYIRNGSFDGMVGALINGQADIGGSPIFFRIDRSKFIDYAVETWPSSFSFVFRHPKHS
SGFHTIYTRPLDHSVWYCIFGLLLITGVSMFLILKVKIRNQSNDAEESSISLAFLFSFGSLCQQGMPISRKTASVK
MLIFITFVYSVTLYQYYNAVIVATLLREPPTTIKSLRDLLESNLKIGVEDVLYNKDYFQRTTDPVAIELYKKKIAY
SKHYNFFEPDKGMALVKQGGFAFHLDNIIAYRFMQKQFTEREICELRDVILYPPQKMAAAVAKRSPYKEHMATGIR
KIFESGLMRRWKTSLDAPMPKCTHTPDSSIFSVNIREFSSPLIALTFGMIIALIILCCEIFIKNVTKD
>DpunIR87a
MHSKTLQAFLVTHFLAIVNSMVMTIGNSNQIAKTAECVLKLSAKYFVEKKALSGSIVIININSYESNTEGLLLQTI
HGGLKYSVMVKDSFYPHANASHFPEKAKNYMLILEEKSELQRNMLQLNKLPTWNPLAKAVVFYQIKNNETAEGTAK
ELINELRDYKLYRTIIFIYDENNDEVISYTWKPYSETNCGGICQTVYILDICRDSKLIQVEAQKEVFPSDMKGCPL
VVYAVVSEPYVMPPIRKLQSASYNDVYEFQSGGEVNLVKIISNFTNMSLVMRMSDREEDWGMIYQNGTSTGAYSVL
RNEFADLMIGNIEVSKTLRKYFHPTVSYIQDEMTWCVPKATPASTWDNLVIIFQWTTWVATFVCLFIMGLLFHYLY
YREHNKNVTKWPTNSLLMTFSMLLGWGASFSPKSSTFRILIFAWLCFSINMEVSYESFLRIFLMHPRFNKQISSQA
ELIESGISLGGRDVYHKYFEDKNFSTFYLYRKYDTITFTEGVKRSALKRDFAVVSSRRQAAYQDQKLGKGAQLIFC
FPESDNLYKYGVVLLARKWFPLLERFNGIIRSVSENGLISKWNQEFLYQTKNDGTNTIVPLSTQHLLGAFMLIGLM
YCMSLIIFLVELISVYLVRRNRWNKVYIKRFGYVP
>DpunIR68a-2
MLELTLLIIMLPALQADKSTILKDVNEGKDLEYLMIDLLNDLTKSQEVTCVAIISDTVYLNVFDGEFFGWTYSVPM
VMTVVEEYEDLLSPNFYTLESLRAARKQGCNVYVILLANGFQAARLLRFGDRHRILDTRAKYIMLHDHRLFHSDLH
YLWKRIVNVIFLKYHRKITGVQKSKAWFDLSTVPFPNPIQGVFVPRRVDIWSKGKFHYNRSLFKDKTNNLNGETLN
VVYLDHVPSVVVTTNNETKKVGGVEIEILNTLSEKMNFHPKLYQPINADLHKWGQKQPNGSFSGLLGEMINGKSDI
ALGNLQYNPYHLEHTDLSIPYTAQCWTFVTPEALSDNSWKTLILPFKLYMWIAVLLVLLITGSIFYGLARYYINLQ
EFRKDRREIASKFVESEKSGHMINDHDAKPVGLYLFGEIINSILYTYGMLLVVSLPKLPSGWSIRILTGCYWLYCI
LLVVSYRASMTAILANPAPRVTIDTLKELADSKVTCGGWGSETKQFFQDSLDDIGQKIGQKFETIDSPLQAANKVA
KGIFAYYDNKDFLKYLIVERKNDFMMPNDNVNSTRNEIVENEKNLHIMSDCVVNIPISIGFHKNSPLKPLADVYMR

RIVEVGLVEKWLNDAMYSIKSLEKNEVEIKALMNLKKLYGAFIALAIGCFVSGVCLIGELLYWNFVVKRDPDFDKY
ALNLYYLKKKK
>DpunIR75q2-2
MNLTCLLVMLLYFSSTICNANIDQQSKLIASVIRTFGRPATVISTVCWPKHRIVELFSYLSEENVIQTSMVHIDEV
NEIPRTNTQEHYTVFLVDLTCVNSIEFINMGNVQNYFRSPYRWIIIDKNNLASDIIPQALTNVNILIDGEVLLVRN
TNQLYLVYKINLISNWTIEPFNIRDDKNSLQISNQFVDSVALRRMNLKGNEIKICYVLTDDDSINHLTDKVKDYVD
TITKVNFPTTNHLLDFLNAQRKYYFANTWGYRVNGTWNGMTGYLVRREVDIGGSPMFFTSERIPFVDYIASPTPTL
SKFVFQQPKLSYENNLFLLSFRTSVWYSIIALIFLVFLVLLIVTIWEWKCMEYNKTETDFSIGVLRANIADIVVLM
FGAACQQGSPVELKGVLGRVVMLILFLTLMFLYTSYSANIVSLLQSSSTQIKTLEDLLNSRIKLGVHDTVFNKYYF
TTATEPIRKAIYEKKIAPPGAVPRFMSMEEGIKKMEKGLFAFHMEIGVGYKFVGKYFKEGEKCGLREIQYLQVMDP
YLAVQKDTPYKEMFKIGLKRIQEHGLQNRENRFLYEKRPKCSGRESNFVSVSMVDCYPALLVLSYGTIFALVILAF
ESLWFYRHNIRNKIRCLLHEHKVRYH
>DpunIR75p.1-2
MNIKLISISFLLLFTTFTIRAYDNDVIKMIISFLIYNERPTSMLVPYLCWDPYQIKILVKSLCDEGIGTAYSFQYN
RTEYHLQYSVIIADLDCPRTSKFMLMANQQGYFKSPYRWLLLSSNETNPILEDIEMSVDSDVVIARRINNERFIFT
EAYKISEKSEIIYTKRLVWWNNDNTKANQETINKLPSKLNVISKALIPGNLLVNGKDSSKNIFLSNYGVIEDYRET
KVLSARRNNLKRHTLTMANVITDSNETRKHMDDRLHLHQDSITKMSYGVVKVCFEMLNATEKLIFTHTWGYRDKNG
KWQGIVDHLIKKEADLGTLTIFTQERMDAVDYIAMVGSTAVRFVFREPPLAYISNIFTLPFTGNVWLAILICVVGC
AVFLYFTSKWEASMSMHPLQLDGSWADVLILIIGAVLQQGCTQEPRYAAGRCVTLILFVALTILYAAYSANIVVLL
RAPSSSVRSLPDLLNSPLKLGASDFEYNRYFFKKLNDPIRKAIYDKKIAPKGKKPNYYTMEEGVEKVRKGLFAFHM
ELNPGYRLIQETYQEDEKCDLVEIDYINEIDPWVPGQKRSPFKDLFKINFLKIRESGLQMCLHRRLQVGKPKCSGQ
VATFSSVGIQDMGPAMLASLYGVLLAPAILALEITYKKLITAREMRKSLIEDQFINPYLD
>DpunIR75p-3
MKLIILILHFLSLSTAKDITTIKFIKSFIVNDNKPSTLVFSLCWTKSIQVSLAKEMSEMGIKASSSKYLESAENFQ
EHTVLFLIDLECPDAAEVIVMAGSKQLFRLGYRWLVLTNDTDDEVSSSLLHRSTLYNSPVLADSDLVLAKRSGNQF
VVVELYKPGLNFTMQTYSRGYFDDSFIDVRPHRELFKRRRNLLGYGITMANVIQDSNSTKYHLPKENGLEPQHDAI
AKICWMNAKLAFEMLNATPRYIFSYRWGYKVNGQWSGMINDLHSERADLGTNCVVSDIERLNVVTYTDMLAPFRVR
FIFRQPPLPYVSNIFSLPFRTSVWLAVFICCIISTVTIYFATKWEARIVRVSSPTQLDGTVSDAMLLTMSAVSQQG
CAMEPKKASGRIMLWVLFTALMALYAAYSANIVVLLQAPSNSIRTLHQLAYSKMTLAANDVDYNHFIFSLFKDPDH
VSIYKIIQPEKGREQFYELNEGVEKIRQGLFAFHSIVEPVYRRVEETFLETEKCDLMEIDFLGSFDPFVPVKKDSP
FLELLRVV
>DpunIR75d-2
MSVRLQASDNRAFVSRFSWLLLHNGTYETNIIESFFYINILPDSDVKISGPDYLLDIYKIKPNHPLVITSLDLNRN
SSLKELSSFWNNFPTPVKRRKDLSNVYLTAATIVTQPQYFKGWSDLTNREIDTYPKLTYPLMMLCSEDLNFRYNLR
QVDLYGDELNGSFNGLAGLLQRNEIEVGITSMFMRADRFGVLHYSSETVSLTGAFIFRQPSQSAVSNIFLLPFSRD
VWLASAVVFLSSGLVLALLSRQLFHMDPILSQLTLSEAFTFALGTVCQQGFHVTPVMASARVVMFFTLTTALFAFT
AYSAKIVAILQTPSDAIQTIDDLTNSPMGMGIQETTYKKVYIAESNEPATQRLYRHKLLPLGERAYLSVVDGISRV
RSGLFAYQVEQSSGYDVISKTFEEHEKCGLKEIEAFSLPMVAIPIKKHSGYRELLASRLRWQREVGLIDRASSIWL
AAKPRCDSAASGFVSVGLLDILPAIQVLAAGAFVAVLILLAEITFAKSSRD
>DpunIR41a-2
MLLLPNSTLLPIENLFYALFDHYLYTSICLTFISEIPLYVQVSISFTFLVPNNTTEGLMYQILPISEMGCSDYIVL
MRHPEDFMVAFEKVVHMGNSRRSDRKIIFLPYLEGSSNSNCSTDLLEVLSMKESSFVANILLALPVEDHLEGISFD
LVTHKYVGLDSEVYQPLYLDRWNSITKTFEKNVNLFPNDIRNLQGKTVRVGCFTYKPYVLLNLDTAISPLDRDGME
MRIIDEFCRWVNCTVEVVNEEEGQWGELYDNQTGIGVIGSVVEDRADFGISALYSWYEEYRMLDFSVAGIRTAITC
LAPSPRLLPSWELPFLPFSWYMWYAVILTFIFASIGLIIAKRCSADQVFLTVFGILISQSHYSADSTWKVRRVTGW
LLITGLILASAYGGGLASSFTIPKYEPSIDTVQDLVDRKLEWGATHDAWIFSLTLSKQPLVKQLVSQFKTYTSDQL
KKKSFTRSMAYSIEKLPAGYFAIGEYLTREALLDMTIMLEDFYYENCVVMLRKSSVYTERISELIGRLHQSGLIHA
WETQVALKHLDYKVQLEVKLSRSKGDVGTFHGLSLQDLLVFYI
>DpunIR93a-2
MKVGSLLLLLLCIFVSGEDFPSLITANASIAVVLDRQYLGEKYQAVLDELKDYIKELARVELKHGGVVVHYYSWTAI
SLRKGFLAVFSVASCEDTWSLFDRTQEEELLLFALTEVDCPRLPVNSAITVTYSEPGGELPQLLLDLRSSNAVKWK
SAVILHDDTLSRDMVSRVVQALTTQKDEDSAPPVSVTVFKMKHEINEYLRRKETHRVLSKLPVKYIGENFISIVTS
DVMTTMADTARELLMSHTTAQWLYIISDTDKYNGNLSSLVNVLYEGENVAYIYNVSDNHPSCKNGIMCYCEEMMDA
FIAALDAAIQDEFDVAAQVSDEEWEAIRPTKAQRRDTLLKHMQLHILSKSSCGNCTRWRILSADTWGATYRDFTDT
PDPKAKDTNDTFTGAIENINLLDVGYWRPIDAIKFKDVLFPHVAHGFRGKELPIITYHNPPWTILKTNESGAVVSY
SGLVFDIVNQLAKNKNFTIRIILPANVKYEFSNSSVDMMHSESAMLTLAAVAKRQAALAAASFTVLPDPMPGINYT
VAISTQSYSFMIVRPRELSRALLFLLPFTTDTWLCLGLAVVLMGPALFVVHRWSPYYEAMEITREGGLSTIHNCLW
YIYGALLQQGGMYLPRADSGRLVVGTWWLVVLVVVTTYSGNLVAFLTFPKQEVPVNTVSELLENSATYTWSLSKGG
YLEMELKNSDEPKYKTLLKGAQLTRGIGGMEGNSEVMVNEILNRVRNERHALIDWKLRLSYLLHADHLATGTCDFV
LSSEEFMDERVAMIVPSGSPYLPVINKEINRMQKAGLITKWLSAYLPKRNRCWKTSTVAQEVDNHTVNLSDMQGSF
FVLFLGFFSASSVLFLEWLYNRRKRRSEQVVIKPYTE
>DpunIR21a-2
MASFIQCCVISFCIMNVTFSEEIEYYASESVLDSKKSLILERARKRVKRNNILSIKNNTNAAKQWRLDLNIEIKEF
KGAKIKRAVDSTFHGYPKTREELWNERFLNESTAFDQTPSLIKLLHNITVTYLKDCTPVILYDNLVKDKESFIFQN
LLKGFPVTFVHGYIGDNDQPKEPKLLRTVKECLHFMIFLTDIKRSAKVLGKQSDSKVVIVARSSQWAVEEFLAGPL
SRMFTNLLVIGQSFNEDVDNTLEAPYILYTHKLYTDGLGASQPVVLNSWTHGKFSRYVNLFPPKMTDGYAGHRFVV
AASNQPPFVFRRIKTDLDGGNPRVVWDGIELRVLALLAERNNFSIEIIEPHEPNLGPGDAVAKEITMGRADIAVAG
MYLTSERIRDMDMSFAHSQDCAVFITLMSTALPRYRAILGPFHWHVWVTLILTYLFGMFPLAFSDKHTLRHLINNS

GEIENMFWYVFGTFTNCFTFLGKNSWSKTTKITTRLLIGWYWIFTIIITSCYTGSIIAFVTLPIFPETVDTIDQLL
SGFYRIGTLDRGGWERWFLNSSDPKTNKLFRKIEFVPNVESGIRNTTKAFFWPYAFLGSQSELEYIVQANFSKTKS
KRAVLHISNECFAPFSVVMGFPNNSLHGKKLSNDIRIMFQTGLIDKIADEVRWEMQRSSTGGFLSAGKGSLKIASA
EEKGLTLEDTQGMFLLLAAGFLMGATALISEWMGGFSRKCCFRKTTPPKSISEEELAGTPDVEALNVLCNGTDSRL
DFGTRSASTDSRDTLDGKVINVTEENILVHGDFNTNQWVSRRSSSIDLDKEVKDIFEKDEKRRQRMNTPFLDSAKS
ESTASKDAFGDRVK
>DpunIR76b
MTGIDLIISTICNATFCEVIYDNPKELPQTRTQSELYYLAKEINGKHLKIATYDNYPISWVEKGENGTNVGRGTAF
VIVEILQKRFNFTYELVVPERNYESGGVKPEDSVIGLLNASKVDMVAAFLPKLTKYQKMVSFSHTLDEGVWRMMLK
RPKESAAGSGLLAPFDSLVWYLILAVVFFYGPCISLLTHIRSKIINDEEHNLPISPSFWFVYSAFIKQGTSLAPEA
NSTRILFATWWIFIIFLSAFYTANLTAFLTLSKFTLEIENTIDLYKKNYRWVAQKGGSVECTVTDPSNDLYYLNKM
LLNGRAEFRDVQADIDYLTLVQGGAVLVKEQIAIDHLMYDDYLSKSRDGVEEANRCTYVVAPNPFMKQLRAFGYPY
DSKMKSLFDSTLVYIQQSGIINFLINQDLPSTKICPLDLQSKDRQLRNTDLMMTYMIIVAGVCAAIAVFVVELVTK
RYVYLKIRKGDKSRRKKLRFSRDISQHFNDTRPPPYDSLFGNETENAVKKIINGREYYVFNKGNGVSKLVPVRAPS
ALLYR
>DpunIR68a
LHDHRLFHSDLHYLWKRIVNVIFLKYHRKITGVQKSKAWFDLSTVPFPNPIQGVFVPRRVDIWSKGKFHYNRSLFK
DKTNNLNGETLNVVYLDHVPSVVVTTNNETKKVGGVEIEILNTLSEKMNFHPKLYQPINADLHKWGQKQPNGSFSG
LLGEMINGKSDIALGNLQYNPYHLEHTDLSIPYTAQCWTFVTPEALSDNSWKTLILPFKLYMWIAVLLVLLITGSI
FYGLARYYINLQEFRKDRREIASKFVESEKSGHMINDHDAKPVGLYLFGEIINSILYTYGMLLVVSLPKLPSGWSI
RILTGCYWLYCILLVVSYRASMTAILANPAPRVTIDTLKELADSKVTCGGWGSETKQFFQDSLDDIGQKIGQKFET
IDSPLQAANKVAKGIFAYYDNKDFLKYLIVERKNDFMMPNDNVNSTRNEIVENEKNLHIMSDCVVNIPISIGFHKN
SPLKPLADVYMRRIVEVGLVEKWLNDAMYSIKSLEKNEVEIKALMNLKKLYGAFIALAIGCFVSGVCLIGELLYWN
FVVKRDPDFDKYALNLYYLKKKK
>DpunIR93a
MMHSESAMLTLAAVAKRQAALAAASFTVLPDPMPGINYTVAISTQSYSFMIVRPRELSRALLFLLPFTTDTWLCLG
LAVVLMGPALFVVHRWSPYYEAMEITREGGLSTIHNCLWYIYGALLQQGGMYLPRADSGRLVVGTWWLVVLVVVTT
YSGNLVAFLTFPKQEVPVNTVSELLENSATYTWSLSKGGYLEMELKNSDEPKYKTLLKGAQLTRGIGGMEGNSEVM
VNEILNRVRNERHALIDWKLRLSYLLHADHLATGTCDFVLSSEEFMDERVAMIVPSGSPYLPVINKEINRMQKAGL
ITKWLSAYLPKRNRCWKTSTVAQEVDNHTVNLSDMQGSFFVLFLGFFSASSVLSGVVVQ
>DpunIR75d
LSNVYLTAATIVTQPQYFKGWSDLTNREIDTYPKLTYPLMMLCSEDLNFRYNLRQVDLYGDELNGSFNGLAGLLQR
NEIEVGITSMFMRADRFGVLHYSSETVSLTGAFIFRQPSQSAVSNIFLLPFSRDVWLASAVVFLSSGLVLALLSRQ
LFHMDPILSQLTLSEAFTFALGTVCQQGFHVTPVMASARVVMFFTLTTALFAFTAYSAKIVAILQTPSDAIQTIDD
LTNSPMGMGIQETTYKKVYIAESNEPATQRLYRHKLLPLGERAYLSVVDGISRVRSGLFAYQVEQSSGYDVISKTF
EEHEKCGLKEIEAFSLPMVAIPIKKHSGYRELLASRLRWQREVGLIDRASSIWLAAKPRCDSAASGFVSVGLLDIL
PAIQVLAAGAFVAVLILLAEITFAKSSRDLLRCRK
>SfruIR25a
MSSLTVFLLFYFIRNTFGQTTQNINVLLINEESNALAEKAFEVAKEYVRRNPSLGLAVDPVIVVGNRTDAKVFLEN
VCRKYNDMLSAKKTPHVVLDFTMTGVGSETIKSFTEALSLPTISGSFGQVGDLRQWRSLNANQTRFLLQVMPPADI
LPEAIRAIVTKQDITNAAIIFDEFFVMDHKYKSLLQNIPTRHVITPVKSFEANEIKTQLESLRNLDIVNFFIVGSL
RTIKNVLDAADKNQYFGRKTAWFALSLEKGDISCGCKNATIVHIRPTPDANSRDRLGKIKTTYSMNGEPEITSAFY
FDLSLRTFLTIKSLLDSGKWPNDMKYITCDDYDGKNTPNRTLDLKTAFQEIKETPTYAPFYIPPDDPMNGRSYMEF
STDLLAITVKDGASISSHSLGSWKAGLSSNLTLTDPNNMSNYSAQLVYRIVTVEQKPFIIRDEQAPKGFKGYCIDL
IEEIRAIVKFDYEITLAPDGNFGTMDENGNWNGIIKELVDKKADIGLSSLSVMAERENVVDFTVPYYDLVGITIMM
KLPRTPTSLFKFLTVLENDVWLSILAAYFFTSFLMWVFDKWSPYSYQNNREKYKEDEEKREFTLKECLWFCMTSLT
PQGGGEAPKNLSGRLLAATWWLFGFIIIASYTANLAAFLTVSRLDTPIESLDDLSKQYKIQYAPLNGSAAMTYFQR
MANIEEKFYEIWKEMSLNDSLKEVERAKLAVWDYPVSDKYSKMWQAMEEAVLPNTIEEAIQRVRDSKSSSEGFAWL
GDATDVKYHVMTSCDLQSVGDEFSRKPYAIAVQQGSPLKDQFNNAILQLLNKRKLEKLKEIWWNNNPESMKCEKQD
DQSDGISIQNIGGVFIVIFMGIGLACVTLGVEYWWYKWRKRPAVGDVTQVEPAKFTRNNVDKQGEGFNFRGRNLGL
NFKPKF
>SfruIR60a
MNFSVEIMSPGKEVEICDKDNYNLGVLHVDTFVAIPSANYFIIIIDSYTDFSYLASKLIRSRSWNPFAKFIILLFN
YVRNDKINIEYVEKVLSCLFKYNAINIVIAVPQASNVRNAIIYSWRPYDPPKYCGYFNETAKDRLVAVNTCERGRL
KYNNSVFEDKVPYDMNGCVMEILALQRQPFISDDEQYTSIEKIMIDRMLKRFKMKAHYHFLEGFRGERENVGEWNG
ALKKLSSKTGQLLLGGIFPDFDVHEDFETTTTYLADVYTWVVPRAPKSAPWVALVFVFKRLVWYSVIVCFVLCGIA
WTVIGRLSGESPYNKSLFHCFLNTWITTMGFVTYLHPKKDSLRVYFVFLNMYCILFSAAYQTQLFDVLTRTSYDHQ
MNTVQELVDSGIKFGGYEELHDLFYNSTDPFDNLIGEKWVDIENITDALINVAVYRNFSLLCSKLELKHISAVTPA
LSDNAGNYNYHTFSDNVFNVPIETIALRGFPFMLKFSTTITIFKQSGLNEGLRHQFTEFTERRRARQLRDLLKEKS
DVSPLTAKHLQGGFFALFLGYVSGIFTLIAEVLVNTGSFKKKFAQCKRKLNFM
>SfruIR75d
KYVGGRFDLRQVDFYGVSHNGSFDGLVGHLQRREAEVGLASLFMRHDRMQVADFFSETCVLACAFIFRQPSRSAVS
NVFLAPFSAGVWGASACVAASAALLLVALRRLRQRTRASTDLRLFTLLEAITFALGSLCQQGCKDSFNLTFRVVRL
PHVHRVFRRVPQDAAGDVSARGDVQHAADVTVRVHGVLRQDRGHLADSKQRAADHRRPGAVSHDHRRPGHHLQDGL
LSREPGEVDAAAVPAQDPAAGRARVPQRRGRHRARAHRLLRLPGGEELWLRHNQADVHGAREVQPQRDRGVQAAAR
GRAHEETLRI
>SfruIR64a
MNLLCNLLLALSVTDVRLVIDIFKLKNLKNGVIFHCYDNYVVSNVHKVLNEHGILVASANIDYNVTYNVATSYPKV

GLVIDAACERWTSVLDSDTISFQGYSFIIITEDVTGTTEMLSQYPIEVDSDVIVAHKINQTFNLYEVFNTGTKYRG
TYNVRKVGHWNTSLFINSPNRWNLQGIFVKTAVIILTTPRIVNQTIEQYMEKPIKSQIDVDTVHRMKYFIMLKFMR
DMYNISYDIHRVSTWGYQRNGSFDGMVNALYQGMAEIGGAPIFYRIDRGERVQYISEVWMSRHSFLFRHPKYPGGF
YTIYTRPLSDVVWYCVVAMLAVTAVTLWVMLVVQNHKGDNEDSSLSLAGLVIWGAICQQGISINRESTSTKLVIFT
TFVYAVTLYQYYNATIVSSLLLEPPRNIRTLKDILDSDLKAGSHDIVYDRDYFKRTTDPVAIELYHKKVATSAQYN
FFTPEEGIALVKKGGFAFHIDTTFAFPLIKATFTEREICETTLVQMYPLQRMGVVVRKHSPYKEHIAYAIRKMYEV
GLPPRIQSEIDEPMPECAHTPDSSIFCVGIREFSTPLLALTFGMATSIVVLLCEIIIDRVVQLGRVRDFRH
>SfruIR40a
MLLLKLLRVILFVCGVSVHGFFDVRDVTSDIMTSLPKDFSVAVKDIAEALPSKSITIVRGNSTNIRSQDVFELFCM
LSEHNVLTTNLDIATKENKQKYYELLKKALDVSDQRTSLILCEPYECENILYELTDNNLIHSMILYIFYWSYGQVS
ETFLMTIKEAMRVAVITNPRESVFRIYYNQGTSDRLNHLTLVNWWSGRLYKSPVLPPPDKVYHDFKGRVFEVPVLH
APPWHFVKYNNDNTITVTGGRDDKLLALIAKKLNFKYKYYDPPDRSQGSSISGNGTFKGTLGLIWKRKADFFLGDV
TMTWERLQAVEFSFMTLADSGAFLTHAPAKLSETLAIIRPFQWEVWPLVFATLLVTGPALWIVIAAQSLWQKRKCD
QLSLFSSCCWFTTTLFLRQSSSKEPSSTHKARLVSVLISLGATYYVIGDMYSANLTSLIAKPSRERPIGTLSALEEA
MRDYGYQLVVESHSSSLAILENGTGVYGRLANLMRRQRVQRVRNVEVGVRLVLSHKRVAVLGGRETLYYDTERFGS
HNFHLSEKLYTRYSAIALQIGCPYLETFNNVVMTLFEAGIIAKMTTDEYKNLPEHARRSDPVTESDKQGGEVLGDS
AAASSQTPQGESTKGLQPVSLRMLRGAFCLLGIGHLLAAISLAVEIQLHRRSKRRKKPAQNEHRKAQKLLVLGKSV
LLFKRGCKKVCTSMFTSIDKALGSDVKD
>SfruIR100
MRALHLVVLFLHTVNCLEIEIPQRVYTDKLLKCSLDIIKKYFAETKVMTYVGSKVHDKELLQAIHDMNLVSMVSKR
ADSKVPVPHQGYLICAKNFTHFVGHFTNIIKEATWNPNARFLFIVRELTYEKLKTIFDITLKLHVNDVVVVNSTDD
ADLYTYNPFDNYNCGKRYDDIISYGKCSEADSINLYPNKFVTGLRNCTFRVWTTQWPPYTMVPEVNSTDPFLLKHG
VEPYLLSLMGEMLGFDLDIIGHKDITDDFPTVSKDMEAIGSLKRIQDNEVDIVVGGMLLVPSRAAAFFYVYGHQVY
TDEIRFVVRRATEEPAWKNVYLEFSTTVWVLLLLTLVLYSFILIIILLRAADKGYIVLILLDILVLHGRNIRSRWMV
KMVLLSWVLFAYLVNVFYQSNLVSLTTNPVLQYQISNEEDIFKYNLKPCLSLIMGRYYIESVQSDREYDMDSGCYG
LMESVETVSKSDNLFALLLYAIYQYYEDDYFDEYGDPRVVTVSKPYSKVIYTFYLYKGFPLIHDLCQKCLQLRENG
LVDKAMRDLNFLKKIKHTFRQSQFQPRFAVPWIVYVFGCTISIVTFAIELLSKY
>SfruIR41a
MLLPNITFPLELLLNAIITQYMESAYCVTVFSDKPLSLTFTNSFIYLVPDEDNLVEQIFNVSEMGCSDYIVCMRDP
QNFMTAFERVVHIGNVRRSDRKVIILPYNEEYNENSDENLPSLIFSMKGSEYLANMLMVVNRNSNNSDCKAFDLVT
HQFVGPDDMSNLPIYLDRWDSCTQQFENDANLFPQDMTNLYGKTVKVACFTYKPYVLLDIDTAIEPLGRDGVEIRI
VDEFCRWLNCTVELVREDVDQWGEIYKNESGGVGVIGSVVEDRAHLGITALYSWYEEYRVMDFSVAGVRTAITCIA
PAPRLLSSWEMPLMPFTWYMWLAVVFTYFYASTGILTAQGCDSSSYPFLNAFGMMIGQSQYEGKPSWKIRSVTGWL
LIAGLILSSAYGAGLASTFTVPRYEPSIDTVQDIVDRKMEWGATHDAWIFSLTLSSEPLVKELVKQFRIYSFDELK
RKSFTRSMAYSIEKLPAGNFAIGEYVTQEAILDMMLMLEDFYFEQCVVMMRKSSPYTEKVSQLIGRLHQSGLLLAW
ETQVR
>SfruIR75p
MSAARLCIPQRLTAPSVGDSRDPRQSFMSVRRTGLQGSGIFMVVSIEDPGLLELQWFWEVVAGFSHKKGDEYLLIE
ELGIECRKTSRNMDAHILVPTLAVFIFLVKYVDSNDFDLDVISSFVTLDERPVSLLIPFVCWKQHDLKSLARRMCE
VGVSTATSLQYNRTEYDLQYVLIIADLSCEGTDRFLVKASKEGYFKAPYRWLLLNYEDDDTALLHADILVDSDVVL
VNKIHDQEYWFVEAYKISENSEIIHTKRLIWRRNNTKLDKNITFMDQNNVTDTYNTTSPVIYQYNDIKNNRLGVPT
NTDVLKVTVTKYGRVEDYRSSNVLSSRRYDLRGHTLTMVNVITDSNETRLHMNDRLYLHQDCITKMSYAVVSICFH
MLNATERLLFTHTWGYKDKNGQWQGIVDHLLKKDADLGTLTIFTQERMDVVDYIAMVGTTAVRFVFREPPLSYISN
IFTLPFSGTVWLAIFICVLGCSIFLYIASKWEASMGMHPLQLDGSWADVLILIIGAVLQQGCTLEPRYGAGRCVTL
ILFIALTVLYAAYSANIVVLLRAPSSSVRSLPDLLNSPLKLGASDFEYNRYFFKKLNDPIRKAIYEKKIAPKGKQA
NFYSMKDGVERIRKGLFAFHMELNPGYRLIQETYQEDEKCDLVEIDYINEIDPWVPGQKRSPFKDLFKINFLKIRE
SGVQANVHQRLTVARPRCSGHVSTFSSVGITDMYPAMLMTLYGMLLAPAVLLLEITYKRLMTIRNQKRRLVDPDHI
PFRH
>SfruIR76b
MAGIELIISSICNATFCEVPYNETYQAPDALAEKDINFMSLMKEVNGKHIKVTTYNNTPLSSTEFENGTVVGKGVA
FTIMNILRKKFNFTYEVILPTKNFELGDKISDDSIIGLLNTSKVDMAVAFIPTLLPYREKVSFSIDLDEGVWVMML
KRPKESAAGSGLLAPFNDLVWYLVLAAVLTFGPCITFFTRVRSKLITDDEGVLPLKPSFWFVYSAFLKQGTNLSPE
ANTTRVLFVTWWLFMILLSAFYTANLTAFLTLSKFTLAIESPRDLYQKNNRWVASAGSSVEHVVKTEGEDLYFLNT
MINSGKARFLSVLGDKDFLEHVKKGEVLVKEQTVVDHLMYNDYISKKDVEESEKCTYVVAPSAFMKKQRAFAYPVG
SKLKGLFDPVLTQIFQAGILDFLKRSDLPSTKICPLDLQSKDRKLRNSDLIMTYMVMVAGSATAVAVFGAEIFIKR
YVSGKLNKNKKSKRKKSKTGKPSRSHDDSRPPPYDSLFGKNPKFNVENTRTKMINGREYYVFETSSGDKKLIPARA
PSSFLYRSDK
>SfruIR75q.1
MIVAHTSYVIFTVTSQTVVAFTMKYLTFFLNIICLNFCVSLNTNSKLQIIVDIAKSFNKPTSVVAKMCWESNKRSK
YAPVEAKLAKMLANLDRPMNIRYLRANETIENDNYPNNDLLFIMNRTCEDANAFLRWASANHKFRKSYRWLILGKS
LIIKDETFKVSPEFDNIRISVDSEVIIIEENNTDEVSLHTFYKLKPHTQWIIEDYGNWSPITGFIKSRTRIESNVM
RRKDFMGETLITSVAISDNSTKTDLLGLGNIFIDTPAKSSFRTIVPLFDFLNATKVVIFSDTWGYFINGSWNGMIG
DISRGEADLCGIVTFITKERMTILEYLTHPTPITLKFVFRQPPLSYQNNLFLLPFSTGVWLCTGAFIVILIAILYI
NTRWDAKKYKYYNKQKIDQTCLPPTWSDITIFVLSAISQQGSSNELKGTLGRLVMFLVFLAFVFLYTSYSANIVVL
LQSTSNQIRTLSDLLHSRLELGLEHAPFNKFYFSSAYTADDPIKKALVDTKIAPKGVLTNVMNIEQGVRIMQKRPF
AFNMNTGTGYRIVSAIFQEHEKCGLQEIEYIPNSNPWLCSRRLSPYGELFKVGYIRIQEHGLSDRENRLIYAKKPA
CTVMGGSFGSVNMVDFHPVCLVLLYGMILAFLLLGVEILVHRKQMKIRNQAVVE
