# Supplementary material for: Identification and Characterization of Chemosensory Receptors in the Pheromone Gland-Ovipositor of Spodoptera frugiperda (J. E. Smith)
Source: Insects. 2022 May 21;13(5):481. doi: 10.3390/insects13050481 (PMC9146910; doi:10.3390/insects13050481)
Supplement: Supplementary file 1 [file insects-13-00481-s001.zip › insects-1715090-supplementary-Table S3.pdf]

Table S3: Evaluation of sequencing data of *S. frugiperda* samples.

PG-OV	Read Number	Base Number	GC Content	⩾Q30 (%)
Repeat 1	44,017,476	6,602,621,400	47.01%	89.61%
Repeat 2	41,405,096	5,854,668,456	46.24%	89.47%
Repeat 3	36,785,916	6,649,778,988	46.43%	89.94%
