# Supplementary material for: Identification and Characterization of Chemosensory Receptors in the Pheromone Gland-Ovipositor of Spodoptera frugiperda (J. E. Smith)
Source: Insects. 2022 May 21;13(5):481. doi: 10.3390/insects13050481 (PMC9146910; doi:10.3390/insects13050481)
Supplement: Supplementary file 1 [file insects-13-00481-s001.zip › insects-1715090-supplementary-Table S4.pdf]

Table S4. Assembly summary of pheromone gland-ovipositor (PG-OV) transcriptome in *S. frugiperda*.

Length Range	Transcript	Unigene
200–300	94,890 (33.57%)	53,990 (45.01%)
300–500	77,422 (27.39%)	31,919 (26.62%)
500–1000	62,528 (22.12%)	19,457 (16.22%)
1000–2000	33,155 (11.73%)	9,592 (7.99%)
2000+	14,636 (5.18%)	4,970 (4.14%)
Total Number	282,631	119,928
Total Length	188,846,354	68,151,470
N50 Length	969	785
Mean Length	669	569
