# Supplementary figures and images for: Identification and Characterization of Chemosensory Receptors in the Pheromone Gland-Ovipositor of Spodoptera frugiperda (J. E. Smith)
Source: Insects. 2022 May 21;13(5):481. doi: 10.3390/insects13050481 (PMC9146910; doi:10.3390/insects13050481)

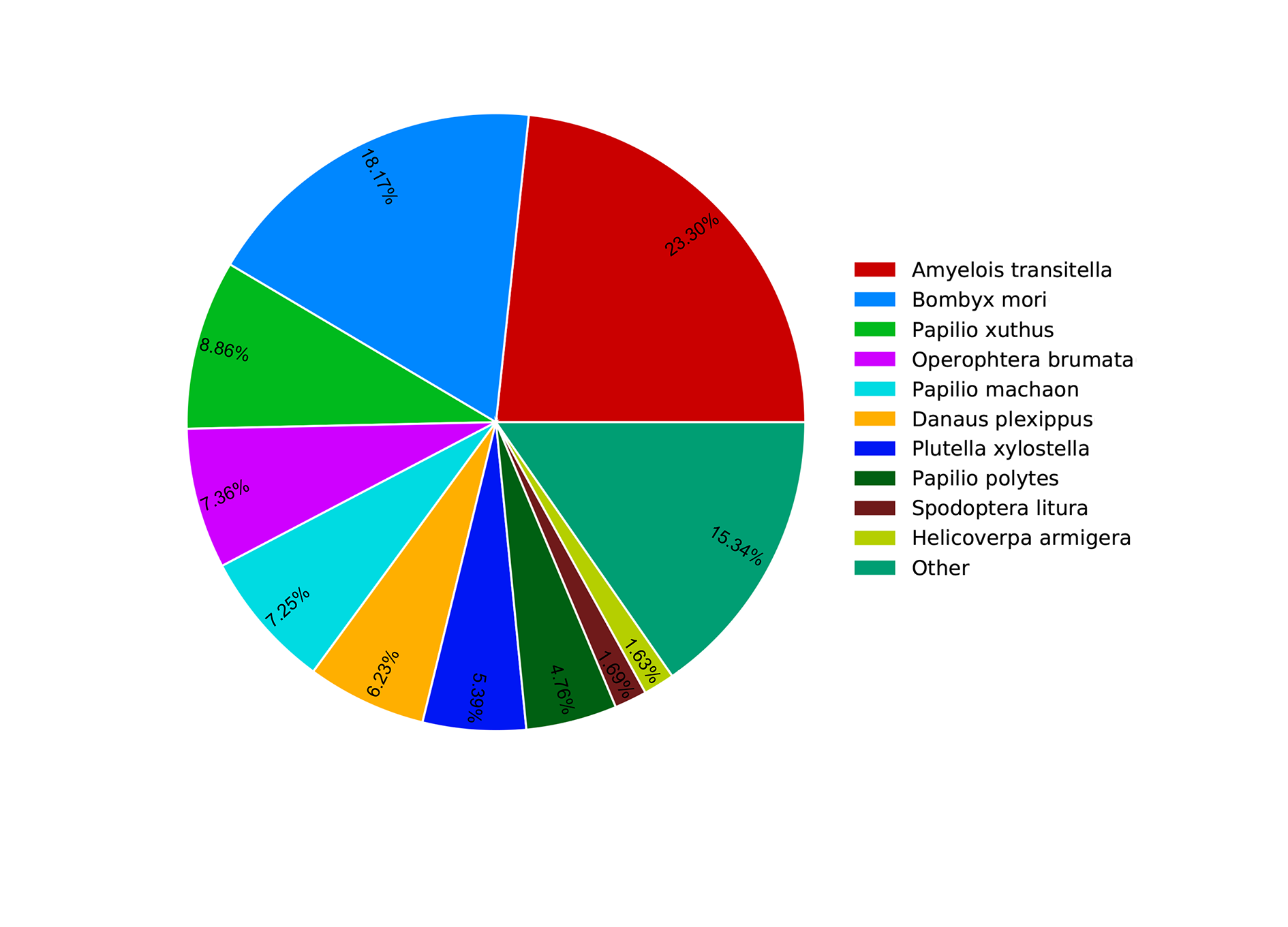

Supplement: Supplementary file 1 [file insects-13-00481-s001.zip › insects-1715090-supplementary-Figure S1.tif]

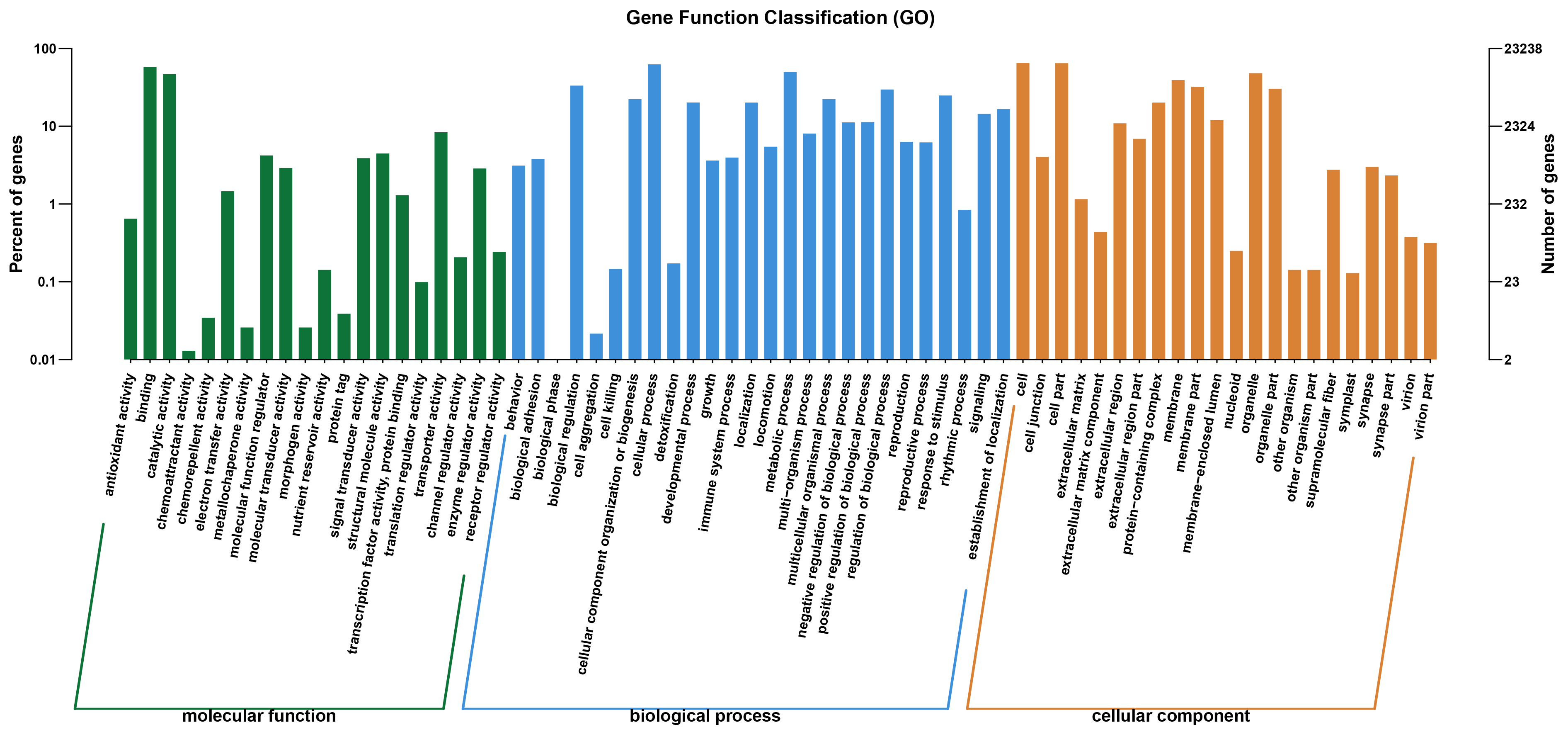

Supplement: Supplementary file 1 [file insects-13-00481-s001.zip › insects-1715090-supplementary-Figure S2.tif]
